# Measuring the Direct Medical Costs of Hospital-Onset Infections Using an Analogy Costing Framework

**DOI:** 10.1007/s40273-024-01400-z

**Published:** 2024-07-05

**Authors:** R. Douglas Scott, Steven D. Culler, James Baggs, Sujan C. Reddy, Kara Jacobs Slifka, Shelley S. Magill, Sophia V. Kazakova, John A. Jernigan, Richard E. Nelson, Robert E. Rosenman, Philip R. Wandschneider

**Affiliations:** 1 Division of Healthcare Quality Promotion, US Centers for Disease Control and Prevention, 1600 Clifton Road, MS H16-3, Atlanta, GA 30329-4027 USA; 2 Department of Health Policy and Management, Rollins School of Public Health, Emory University, Atlanta, GA USA; 3 IDEAS Center, Veterans Affairs Salt Lake City Health Care System, Salt Lake City, UT USA; 4 Department of Internal Medicine, University of Utah School of Medicine, Salt Lake City, UT USA; 5 Emeritus professor, The School of Economic Sciences, Washington State University, Pullman, WA USA; 6 The Institute for Research and Education to Advance Community Health, Elson S. Floyd College of Medicine, Washington State University, Spokane, WA USA

## Abstract

**Background:**

The majority of recent estimates on the direct medical cost attributable to hospital-onset infections (HOIs) has focused on device- or procedure-associated HOIs. The attributable costs of HOIs that are not associated with device use or procedures have not been extensively studied.

**Objective:**

We developed simulation models of attributable cost for 16 HOIs and estimated the total direct medical cost, including nondevice-related HOIs in the USA for 2011 and 2015.

**Data and Methods:**

We used total discharge costs associated with HOI-related hospitalization from the National Inpatient Sample and applied an analogy costing methodology to develop simulation models of the costs attributable to HOIs. The mean attributable cost estimate from the simulation analysis was then multiplied by previously published estimates of the number of HOIs for 2011 and 2015 to generate national estimates of direct medical costs.

**Results:**

After adjusting all estimates to 2017 US dollars, attributable cost estimates for select nondevice-related infections attributable cost estimates ranged from $7661 for ear, eye, nose, throat, and mouth (EENTM) infections to $27,709 for cardiovascular system infections in 2011; and from $8394 for EENTM to $26,445 for central nervous system infections in 2016 (based on 2015 incidence data). The national direct medical costs for all HOIs were $14.6 billion in 2011 and $12.1 billion in 2016. Nondevice- and nonprocedure-associated HOIs comprise approximately 26−28% of total HOI costs.

**Conclusion:**

Results suggest that nondevice- and nonprocedure-related HOIs result in considerable costs to the healthcare system.

**Supplementary Information:**

The online version contains supplementary material available at 10.1007/s40273-024-01400-z.

## Key Points for Decision Makers

**Table:** 

Traditionally, hospital infection control and surveillance programs have focused on device- and procedure-related hospital-onset infections (HOIs), along with *Clostridioides difficile* infections, as these place a substantial disease and cost burden on the healthcare system.
This analysis develops estimates of the direct medical costs of HOIs that are not associated with a device or procedure (i.e., skin and soft tissue infections, pneumonias, central nervous system infections, lower respiratory tract infections, cardiovascular system infections) to produce a more comprehensive national estimate of the direct medical costs of HOIs.
Our analysis demonstrates the significant direct medical cost savings that can be achieved from enhanced investments in hospital infection control and prevention programs.

## Introduction

Healthcare-associated infections are infections patients can get while receiving medical or surgical treatment in a healthcare setting [1]. Hospital-onset infections (HOIs) represent a subset of healthcare-associated infections that occurred subsequent to an inpatient hospital admission, where there is “a lack of evidence that the infection was present or incubating at the time of entry” into the hospital [2, 3]. The number of infections that occur in US hospital settings has been estimated to be 721,800 [95% confidence interval (CI) 214,700–2,489,400] in 2011 and 687,200 (95% CI 181,400–2,691,200) in 2015 [4, 5]. Approximately 28.5% of infections in 2011 and 23.6% in 2015 were device-associated infections (i.e., central line-associated bloodstream infection, catheter-associated urinary tract infection, and ventilator-associated pneumonia). A major focus of infection prevention efforts in recent decades has been reducing the risk of device-related and surgical site infections [4, 6]. Hence, most published estimates of the economic impact of HOIs have focused on device or procedure-related infections along with *Clostridium difficile* (now *Clostridioides difficile*) and select surgical-site infections [7–10]. A 2013 meta-analysis of published estimates of the attributable direct medical cost associated with these infections found that their cost to the US healthcare system was approximately $9.8 billion (95% CI, $8.3–11.5 billion) annually (2012 US dollars) [6]. Still largely unknown is the magnitude of the attributable direct medical costs associated with a host of other infections that make up approximately 45% of total HOI burden in the USA (nonventilator-associated pneumonias; gastrointestinal infections excluding *Clostridioides difficile*; urinary tract infections not associated with a catheter; primary bloodstream infections not associated with a central line; eye, ear, nose, throat, or mouth infections; lower respiratory tract infections; skin and soft-tissue infections; cardiovascular system infections; bone and joint infections; central nervous system infections; reproductive tract infections; and systemic infections) [4, 5].

While retrospective cohort study designs are commonly used for measuring attributable costs of device- or procedure-related infections, literature reviews of studies on measuring attributable costs point out the difficulties in developing accurate estimates given the variation in study designs, patient settings (intensive or specialty care units versus general medical wards), and statistical analyses [11, 12]. Previous studies used various study settings (ranging from specific patient care units to the general patient population) and usually employed retrospective cohort study designs where patients are grouped by their infection status (patients with a HOI versus similar noninfected patients) where differences in treatment costs between groups provide an estimate of attributable HOI costs [11]. More robust analyses employ matching, multivariate regression analyses, or multi-state modeling to account for other variables affecting patient treatment costs, including (1) severity of underlying disease, (2) health status, (3) treatment intensity, (4) the length of time from infection to the end of hospital stay (time-dependent bias), (5) post-discharge patient follow-up to assess whether a subsequent hospital admission is the result of a HOI acquired during a previous stay, and (6) other factors related to the hospital environment and infection transmission [13, 14]. However, there are still knowledge gaps related to the pathogenesis, epidemiology, and prevention of HOIs, along with the need for improved approaches to the design and conduct of healthcare epidemiology studies [15]. This raises the issue of whether conventional methods of attributable HOI cost measurement can appropriately disentangle the confounding of cost relationships between the patient’s underlying disease, the impacts of other comorbidities and patient characteristics, the impacts on the intensity of patient treatment, and acquiring a HOI.

There exist additional complications associated with the measurement of hospital patient costs. Rules for allocating fixed costs to patients can differ across hospital accounting systems, adding another confounding factor for multicenter studies.^1^ Added to this is the challenge of measuring patient cost in multiproduct firms, such as hospitals where the magnitude of benefits and costs associated with patient treatment are also influenced by jointness in the production of multiple disease treatment programs [17].

Given the conceptual and empirical limitations with using retrospective cohort study designs, there has been interest in using large administrative datasets for prevention effectiveness studies or HOI surveillance. Although these datasets are readily available and less expensive to acquire compared with conducting a systematic review of patient medical records, they lack clinical details that can be used to identify patients with a HOI (with the exception of *Clostridioides difficile* infections) [18–26]. Estimates of the attributable impact of certain HOIs on Medicare reimbursements have been done but with data linked to the National Healthcare Safety Network (NHSN) to determine patients’ infection status [27–30].

Our primary objective was to calculate the total direct medical costs (subsequently referred to as costs) to US hospitals resulting from HOIs in inpatient populations for both 2011 and 2016 (based on 2015 prevalence data) by combining previously published estimates of HOI incidence for 16 different sites of infection (for each year) with our estimates of the attributable costs derived for each HOI type.^2^ To overcome the challenges associated with measuring attributable costs, we employed an analog cost method to develop separate simulation models for generating nationally representative estimates of attributable costs for nondevice- and nonprocedure-associated HOIs, along with device- and procedure-associated HOIs using a single administrative hospital discharge dataset.

## Methods

### Overview

Our overall objective was to estimate of the total cost of HOIs for 2011 and 2016 that can be used to assess the potential impacts of investments in infection prevention interventions and programs (see Supplementary Appendix A, Section I for the formal model). For each study year, our analytical strategy for estimating total costs involved deriving simulation models to estimate the attributable cost associated with 16 types of HOIs using a publicly available hospital discharge dataset, which included the following steps: (1) construct 16 statistical cost models of the attributable costs associated with patients infected with a similar infection type as the primary or principal reason (diagnosis) for the hospital stay, which serves as an analog for the attributable cost of HOI; (2) construct 16 attributable cost simulation models that are parameterized with parameters from the statistical cost models from step 1; (3) generate estimates of attributable HOI costs from simulation models (from step 2) using data for patients that have the same infection type as a secondary diagnosis; and (4) construct national cost estimates for 16 types of HOI using previously published burden estimates from 2011 and 2015, and then multiply them with attributable cost estimates from step 3 [4, 5].

### Analogy Cost Methodology and Analogue Identification

Analogy costing is a methodology used, particularly by the US Department of Defense and the National Aeronautics and Space Administration, to estimate cost on the basis of historical data for one or occasionally two analogous system(s) [31–33]. It takes a currently fielded system, similar in design and operation to the proposed system, and uses it as a basis for costing the analogy. The Environmental Protection Agency uses a similar methodology known as benefit transfer to derive economic values of ecosystems on the basis of existing data from comparable, but previously studied, ecosystems [34]. The cost of the proposed system is then estimated by adjusting the historical cost of the current system to account for the differences between them. Adjustments were made using factors (i.e., scaling parameters) that represent differences in size, performance, technology, reliability, maintainability, and/or complexity [31]. The accuracy of the analogy costing will depend on how similar the costing projects (or objects) are.

Given the difficulties in extracting the attributable cost of an infection from the total cost of a patient with an HOI, we searched for an appropriate analog that could approximate the value of the resources used to treat HOIs. After reviewing various infectious disease treatment guidelines [35–44], we concluded that the resources used to treat infectious diseases were similar, whether the onset of the infection was in the hospital or in the community. Several guidelines recommend the same diagnostic and treatment protocols for HOIs and community-onset infections (COIs) in hospitalized patients (i.e., specimen collection, laboratory testing, radiology, and antibiotic selection) [37, 39–41]. For example, acute bronchitis is the result of a non-pneumonia lower respiratory tract infection (LRTI) that can be either a COI or a HOI. Similarly, there are other diseases associated with a LRTI (such as abscess of the lung or empyema) that can also occur. Given that hospitalization costs (diagnostic and treatment cost) are initially similar for infections that are COI and infections that are HOI, the chances of producing accurate estimates of the costs estimates for HOI are improved.

Applying analogy costing, we developed 32 simulation models of the attributable costs of HOIs (16 infection types for each study year) using hospital cost data from the National Inpatient Sample (NIS). The NIS is a national hospital discharge dataset containing information on inpatient “resource utilization, access, quality, outcomes and cost” [45]. While the NIS reports inpatient hospitalization charges, these were converted to costs using cost-to-charge (CCR) ratio records provided in the NIS [46]. Each attributable cost model consists of two stages. In stage one, we estimated inpatient cost models using median regression for inpatient hospitalizations that had an infection as a principal diagnosis.^3^ In stage two, we parameterized attributable cost simulation models using parameters from the stage 1 statistical cost models. Data from hospitalizations with an infection indicated as a secondary diagnosis (reflecting hospitalizations with an HOI) were then used in the stage two simulation cost models to generate estimates of the attributable cost owing to each infection. The attributable cost estimates were multiplied by the incidence estimates and then summed to produce a national estimate of the attributable cost of HOIs.

Two assumptions regarding HOI costs underlie our approach. First (mentioned above) is that costs associated with resources used to treat a HOI are not significantly different from treating an infection that is the principal diagnosis for the hospitalization. The second uses the same assumption in the opposite direction; the cost associated with additional treatments for an underlying disease that results from an HOI can be approximated using the cost of additional treatments for comorbidities when the infection is the principal reason for hospitalization. Our assumptions imply that COIs resulting in hospitalization require similar levels of resources to treat HOIs. However, there are also cases where hospitalizations with an infection as the principal diagnosis are not a COI, but an actual HOI that has emerged post discharge and results in readmission to the hospital. Published research has shown that the number of post-discharge HOIs can be substantial [48–51]. Thus, our analogy group contains cases of post-discharge HOI. Similarly, our group of hospitalizations with an infection coded as a secondary diagnosis may contain patients in whom the infection may not meet the clinical and/or surveillance criteria to classify it as a HOI. As NIS lacks the clinical information to identify these cases, we assumed that their treatment costs are also similar to the treatment costs for HOIs, regardless of the lack of clinical confirmation of infection.

### Attributable Cost Models and Data Sources

The analysis to develop attributable cost estimates used data from the National Inpatient Sample (NIS) for 2011 and 2016.^4^ The year 2016 was chosen, as opposed to using 2015 data that more directly matched with the 2015 incidence estimate, because of the transition to the International Classification of Disease Codes, version 10 (ICD-10) from the International Classification of Disease Codes, version 9 (ICD-9) that took place in October 2015 [54, 55]. Attempting to use ICD-9 and ICD-10 codes to identify cases of infection within the same year could confound both the identification of cases and the measurement of costs. As producer prices remained flat between 2015 and 2016, the use of 2016 prices should not bias the results [56]. We also adjusted all attributable cost estimates to 2017 prices using the producer price index for general medical and surgical hospitals (PUC #62211) [56] to facilitate comparison of results with other studies.^5^

To identify inpatients hospitalized with infections in the NIS, two infectious disease physicians (Reddy and Jacobs Slifka) developed catalogs of all relevant diagnostic ICD-9-CM codes (for 2011) and ICD-10-CM codes (for 2016) for each of the 16 infection types. These codes were then used to identify all hospitalizations within each infection type as either having a principal diagnosis (recorded as the first diagnosis in the data) or a secondary diagnosis as indicated in the other 24 diagnosis code fields in the 2011 NIS or 29 fields in the 2016 NIS (a table with the codes used for identifying each infection type is presented in Supplementary Appendix B Table 1) [57].^6^ Hospitalizations in the secondary group were restricted to those with hospital stays of three or more days to be partly consistent with definitions used to identify hospitalizations with a HOI. Table 1 contains the catalog of ICD-9 and ICD-10 codes that we used to identify hospitalizations with a LRTI as the principal diagnosis or a secondary diagnosis. In this way, we capture the distribution of total costs for hospitalizations with a non-pneumonia LRTI as the principal diagnosis so it can be used as an analog measure for the attributable costs of non-pneumonia LRTI when it is a HOI.

**Table 1:** ICD-9 and ICD-10 codes for identifying non-pneumonia lower respiratory tract infections (LTRI)

Diagnosis	LRTI ICD-9 codes	LRTI ICD-10 codes
Acute bronchitis	466.0, 466.1, 466.11, 466.19	J20.0, J20.1, J20.2, J20.3, J20.4, J20.5, J20.6, J20.7, J20.8, J20.9
Acute bronchiolitis	466.2	J21.0, J21.1, J21.8, J21.9
Empyema	510, 510.0, 510.9	J86.0, J86.9
Pleurisy with effusion	511.1	J90, J94.2
Abscess of lung and mediastinum	513.0, 513.1	J85.0, J85.1, J85.2, J85.3
Pulmonary disease caused by Mycobacterium	31	A31.0
Acute laryngotracheitis and tracheitis	464.10, 464.11, 464.2, 464.21	J04.10, J04.11, J05.0

*LRTI* lower respiratory tract infection, *ICD-*9 International Classification of Diseases, ninth revision, *ICD-10* International Classification of Diseases, tenth revision

The identification of cases proceeded in a similar fashion for each of the remaining infections, except for device-related infections. Previous research has indicated poor accuracy in coding for central line insertion when trying to identify central-line-associated bloodstream infections [23, 58, 59]. Accordingly, we did not attempt to develop a separate attributable cost estimate for central-line-associated bloodstream infections and only generated one overall estimate for both primary bloodstream and central-line-associated bloodstream infections, particularly because 75–85% of bloodstream infections were central-line-associated in the prevalence studies by Magill et al. [4, 5]. However, procedure codes were used for urinary catheter insertion and invasive mechanical ventilation to identify cases of urinary tract infections associated with a catheter insertion and cases of pneumonia associated with a mechanical ventilator, respectively.

The 2011 and 2015 estimates of HOI incidence used in the costs analysis were taken from two studies by Magill et al., one with a HOI incidence estimate for 2011 and the other for 2015 [4, 5].^7^ It is important to note that these burden estimates are based on definitions of “healthcare-associated infections” from the CDC’s National Healthcare Safety Network that were in place at the time of data collection and data from CDC prevalence surveys of HOIs and the National Inpatient Sample [4, 5, 45, 60, 61].

### Patient Group Comparisons and Model Development

We selected a minimal set of predictor variables for the statistical and simulation models that could best reflect resource use and the treatment of infections in hospitals. Candidates for model inclusion are those that can influence the length of patient stay and relevant risk factors for infection [62, 63]. Variables selected included cost (charges adjusted to cost using cost-to-charge ratios for each facility), variables reflecting patient resource use [length of stay, number of diagnoses, number of procedures, age, and all patient refined diagnosis related groups (APR-DRG)] indexes for patient severity of illness and the risk of mortality (used as continuous variables in the analysis), and variables accounting for cost structure differences related to facilities [variables for urban teaching hospitals, small hospitals with fewer than 250 beds, and a wage index that measures the relative hospital wage level in their area compared with the national average hospital wage level (see Supplementary Appendix A Table 1 for descriptions of study variables taken from NIS)] [64].^8^

To assess the credibility of our analogy hypothesis, we calculated individual univariate statistics on our study variables for each infection type. We evaluated the differences in costs, length of hospital stay (LOS), and the other study variables by creating groups of hospitalizations with an infection as the principal diagnosis (principal group), a secondary diagnosis (secondary group), and a matched group (5:1) of hospitalizations with a similar distribution of principal diagnosis codes as the secondary group but with no infection codes as part of their discharge record (no infection group). Comparisons were performed for each infection type for 2011 and 2016.

The presumption underlying our strategy for the cost relationships of hospitalizations is that treating an HOI adds to the overall direct inpatient costs above the treatment of the underlying disease. If so, the mean cost and mean LOS for hospitalizations in the secondary group must be greater when compared with the principal group and the no infection group.^9^ However, economies of scope are present when treating an HOI that lowers overall patient costs, thus making our analogy unworkable.

An example of a univariate analysis using non-pneumonia LRTI is presented in the results, along with a table of means for all the other infection types by each patient group. A detailed discussion of the two stage simulation models and their specifications are presented in Supplementary Appendix A, Section II. All statistical analyses were performed using SAS version 9.4 (SAS Institute).

## Results

We illustrate the results of our patient group comparisons, stage 1 median regression results, and the stage 2 simulation model calculations using the 2011 and 2016 non-pneumonia LRTI univariate statistics as an example.

### Univariate Analysis

The univariate statistics for the three patient groups (secondary diagnosis group, principal diagnosis group, and no infection group) were as follows (Table 2): the mean total patient costs for the principal diagnosis group of $7415 (2011) and $9179 (2016) were lower than those for the secondary patient group of $26,839 (2011) and $29,367 (2016). Likewise, the mean total costs for the no infection patient group of $13,671 (2011) and $14,398 (2016) were lower than the mean for the secondary group, but higher than the principal diagnosis group. The same pattern was observed for LOS. The means for the principal group (3.83 days in 2011 and 4.24 days in 2016) were also smaller compared with the no infection group (5.63 in 2011 and 5.41 in 2016) and the secondary group (10.35 days in 2011 and 10.37 days in 2016). This pattern was generally observed for all other infections (Table 3). The means of costs and LOS for the principal group were always lower than those of the secondary group while the means of costs and LOS (see Supplementary Appendix B Table 2 for univariate statistics for LOS) for the no infection group were also always smaller than those of the secondary group. However, there was no general pattern in the magnitudes of the differences in costs and LOS between the no infection and principal groups (Table 3 and Supplementary Appendix B Table 2).

**Table 2:** Univariate statistics for patient group comparisons for lower respiratory tract infections (LRTI) 2011 and 2016

Patient characteristics	LRTI—Group comparisons 2011			LRTI—Group comparisons 2016		
	Cases with secondary Dx, LOS > 2 days	Cases with principal Dx	Matched cases with no LRTI Dx*	Cases with secondary Dx, LOS > 2 days	Cases with principal Dx	Matched cases with no LRTI Dx*
Number of cases =	31,266	40,735	156,330	167,961	43,213	839,601
Variables:						
Cost						
Mean	$26,839	$7415	$13,671	$29,367	$9179	$14,398
Median	$11,335	$3833	$7784	$14,237	$5325	$8504
Mode	$3186	$360	$78	$9445	$1465	$3148
Range	$101–1,620,419	$78–766,034	$31–1,089,791	$23–2,850,000	$63–1,142,474	$19–1,636,440
LOS (length of hospital stay)						
Mean	10.35	3.83	5.63	10.37	4.24	5.41
Median	6	3	4	7	3	4
Mode	3	2	2	3	2	2
Range	3–365	1–234	1–364	3–365	1–188	1–354
Number of diagnosis codes, mean (SD)	13.10 (6.45)	6.26 (5.26)	11.05 (6.10)	17.59 (6.37)	8.94 (6.69)	13.58 (6.53)
Number of procedure codes, mean (SD)	2.30 (3.50)	0.55 (1.49)	1.43 (2.26)	2.83 (3.63)	0.90 (1.75)	1.53 (2.35)
Age (years), mean (range)	54 (0–111)	27 (0–108)	59 (0–109)	64 (0–90)	35 (0–90)	62 (0–90)
APRDRG_Severity_Index, mean (SD)	2.86 (0.92)	1.89 (0.87)	2.49 (0.95)	3.14 (0.78)	2.13 (0.90)	2.53 (0.90)
APRDRG_Risk_Mortality_Index, mean (SD)	2.34 (1.09)	1.40 (0.74)	2.12 (1.03)	2.84 (0.96)	1.69 (0.90)	2.29 (1.01)
Wage_Index, mean (SD)	0.9900 (0.156)	0.9871 (0.155)	0.9969 (0.159)	0.9950 (0.191)	1.0014 (0.194)	0.9960 (0.194)

*Dx* diagnosis, *SD* standard deviation

**Table 3:** Means of total patient costs by patient group and infection type

Type of hospital-onset infection	Mean of total costs 2011			Mean of total costs 2016		
	Infection as secondary Dx	Infection as principal Dx	No infection Dx	Infection as secondary Dx	Infection as principal Dx	No infection Dx
Bone and joint infection	$26,375	$17,650	$14,452	$25,355	$16,768	$14,721
*n* =	42,552	21,776	212,760	42,549	22,409	212,745
Cardiovascular system infection	$43,249	$26,555	$18,144	$34,174	$22,195	$16,131
*n* =	9125	5015	45,625	22,336	5654	111,680
Catheter-associated urinary tract infections	$23,879	$9852	$18,756	$24,168	$9601	$20,145
*n* =	9064	2230	26,320	6253	1163	17,253
Central nervous system infection	$44,180	$21,285	$15,927	$48,930	$20,256	$16,737
*n* =	10,790	12,877	53,950	9759	8339	48,795
*Clostridium difficile*	$37,454	$11,378	$15,804	$33,358	$9442	$15,423
*n* =	45,608	23,829	228,040	45,591	20,983	227,955
Eye, ear, nose, throat, or mouth infection	$17,616	$5601	$12,127	$21,168	$6899	$13,028
*n* =	69,880	25,409	349,395	70,703	22,131	353,349
Gastrointestinal infection	$28,311	$13,226	$13,606	$28,039	$13,237	$13,980
*n* =	116,457	101,290	553,289	113,501	94,606	556,692
Pneumonia	$29,712	$10,424	$13,876	$27,139	$9887	$14,186
*n* =	276,384	211,745	1,158,662	285,522	156,343	1,122,370
Primary bloodstream infection	$41,308	$21,419	$13,909	$40,143	$18,267	$14,258
*n* =	146,190	217,950	728,674	132,732	377,953	656,091
Reproductive tract infection	$14,045	$5599	$9012	$16,733	$7008	$9830
*n* =	8825	9495	44,125	9799	8578	48,995
Skin and soft-tissue infection	$21,929	$7675	$14,443	$21,519	$8763	$14,483
*n* =	191,787	134,747	859,036	198,195	131,301	915,776
Surgical-site infection	$49,746	$18,877	$15,789	$51,902	$22,733	$16,528
*n* =	34,975	64,052	174,875	16,337	19,873	81,685
Systemic infection	$25,888	$6718	$12,679	$26,446	$14,989	$12,787
*n* =	447,047	8478	1,590,801	456,813	11,746	1,497,653
Urinary tract infection	$21,202	$7259	$13,270	$19,467	$6888	$13,937
*n* =	465,732	130,217	1,961,233	452,591	103,923	1,868,330
Ventilator-associated pneumonia	$68,967	$43,211	$41,913	$66,083	$38,786	$41,871
*n* =	57,311	7189	128,400	54,664	4570	132,297

*Dx* diagnosis, *n* number of observations

### Median Regression Analysis and Attributable Costs

**Fig. 1:**
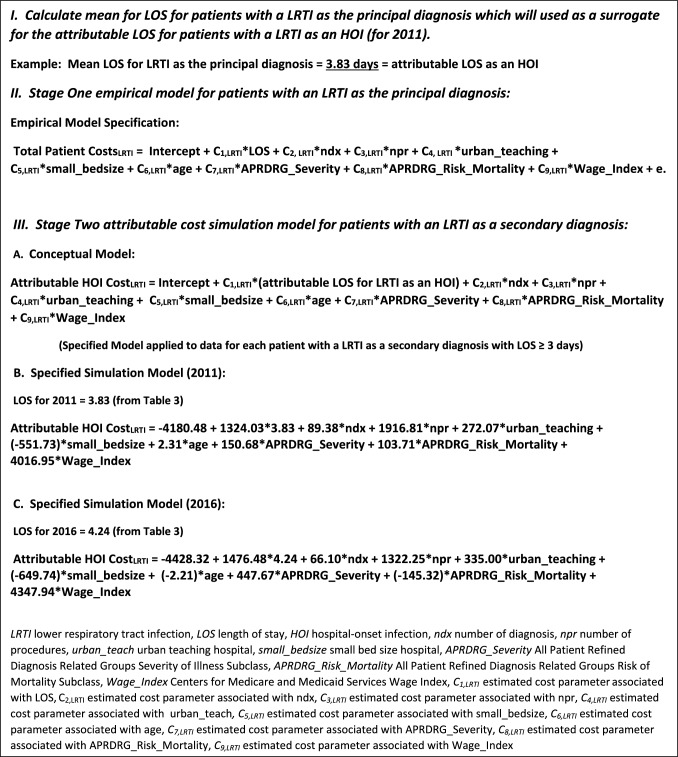
Attributable HAI simulation cost model for lower respiratory tract infections. *LRTI* lower respiratory tract infection, *LOS* length of stay, *HOI* hospital-onset infection, *ndx* number of diagnosis, *npr* number of procedures, *urban_teach* urban teaching hospital, *small_bedsize* small bed size hospital, *APRDRG_Severity* All Patient Refined Diagnosis Related Groups Severity of Illness Subclass, *APRDRG_Risk_Mortality* All Patient Refined Diagnosis Related Groups Risk of Mortality Subclass, *Wage_Index* Centers for Medicare and Medicaid Services Wage Index, *C*_*1,LRTI*_ estimated cost parameter associated with LOS, C_2,LRTI_ estimated cost parameter associated with ndx, *C*_*3,LRTI*_ estimated cost parameter associated with npr, *C*_*4,LRTI*_ estimated cost parameter associated with urban_teach*, C*_*5,LRTI*_ estimated cost parameter associated with small_bedsize, *C*_*6,LRTI*_ estimated cost parameter associated with age, *C*_*7,LRTI*_ estimated cost parameter associated with APRDRG_Severity, *C*_*8,LRTI*_ estimated cost parameter associated with APRDRG_RISK_MORTALITY, *C*_*9,LRTI*_ estimated cost parameter associated with Wage_Index

Table 4 contains the results of the median regression analysis for 2011 and 2016 for the LRTIs (stage 1 of the cost model). The stage 1 regression parameters were used to construct our stage 2 attributable LRTI cost simulation model, along with the mean LOS for each year (from Table 2), which reflects the attributable length of stay owing to a LRTI. The fully specified LRTI simulation models for 2011 and 2016 are shown in Fig. 1 (the coefficients C_1,LRTI_, C_2,LRTI_, C_3,LRTI_, C_4,LRTI_, C_5,LRTI_, C_6,LRTI_, C_7,LRTI_, C_8,LRTI_, and C_9,LRTI_ are the cost analogs for the attributable cost of these infections).

**Table 4:** Median regression results for lower respiratory tract infections (LRTI)

2011 Median regression estimates for cost of LRTI as the principal diagnosis (*n* = 40,643)						
Parameter	Estimate	Standard error	95% Confidence limits		*t*-Value	Pr > |t|
Intercept	−4180.48	91.1787	−4359.192	−4001.767	−45.85	< 0.0001
Length of hospital stay	1324.030	12.2049	1300.1083	1347.9519	108.48	< 0.0001
Number of diagnosis codes	89.3800	5.9700	77.6787	101.0814	14.97	< 0.0001
Number of procedure codes	1916.808	41.1644	1836.1245	1997.4906	46.56	< 0.0001
URBAN_TEACHING_HOSPITAL	272.0654	22.1886	228.5752	315.5556	12.26	< 0.0001
URBAN_TEACHING_HOSPITAL	0.0000	0.0000	0.0000	0.0000	–	–
SMALL_BEDSIZE_HOSPITAL	−551.727	33.9330	−618.2360	−485.2170	−16.26	< 0.0001
SMALL_BEDSIZE_HOSPITAL	0.0000	0.0000	0.0000	0.0000	–	–
AGE	2.3119	0.5740	1.1868	3.4370	4.03	< 0.0001
APRDRG_Severity_Index	150.6755	27.3487	97.0713	204.2796	5.51	< 0.0001
APRDRG_Risk_Mortality_Index	103.7112	39.6886	25.9206	181.5019	2.61	0.0090
Wage_Index	4016.947	78.7623	3862.5711	4171.3228	51.00	< 0.0001

2016 Median regression estimates for cost of LRTI as the principal diagnosis (*n* = 43,111)						
Parameter	Estimate	Standard error	95% Confidence limits		*t*-Value	Pr > |t|
Intercept	−4428.32	92.3444	−4609.313	−4247.32	−47.95	< 0.0001
Length of hospital stay	1476.475	10.8909	1455.129	1497.8214	135.57	< 0.0001
Number of diagnosis codes	66.0973	4.87	56.552	75.6426	13.57	< 0.0001
Number of procedure codes	1322.245	25.6503	1271.97	1372.5205	51.55	< 0.0001
URBAN_TEACHING_HOSPITAL	334.9984	28.5321	279.075	390.9218	11.74	< 0.0001
URBAN_TEACHING_HOSPITAL	0	0	0	0	–	–
SMALL_BEDSIZE_HOSPITAL	−649.742	37.0221	−722.3062	−577.1779	−17.55	< 0.0001
SMALL_BEDSIZE_HOSPITAL	0	0	0	0	–	–
AGE	−2.2123	0.6268	−3.4408	−0.9838	−3.53	0.0004
APRDRG_Severity_Index	447.6694	26.6918	395.3529	499.9858	16.77	< 0.0001
APRDRG_Risk_Mortality_Index	−145.316	29.054	−202.2622	−88.3695	−5	< 0.0001
Wage_Index	4347.944	71.203	4208.385	4487.5038	61.06	< 0.0001

Using the 2011 NIS data for hospitalizations with LRTI as a secondary diagnosis (31,266 hospitalizations, Table 2), the average of the attributable cost estimates from the stage 2 model for these hospitalizations was $11,301 in 2011 dollars (Table 5), where the attributable costs associated with LOS equaled $5071 ($1324 × 3.83) while the remaining cost of $6230 stemmed from the impact of infection treatment intensity (number of extra diagnosis codes, extra procedure), patient’s disease severity and mortality risk, and the changes in costs given the hospital type and relative wage costs where the infections occurred (in 2011 dollars). Similarly, the 2016 simulation model for LRTI costs produced a mean attributable cost estimate of $12,013 in 2016 dollars (Table 6), where costs associated with attributable LOS equaled $6258 ($1476 × 4.24) and all other cost impacts equaling $5755. Supplementary Appendix B Table 3 contains the 2011 and 2016 median regression results for all other HOIs.

**Table 5:** Burden, length of stay, attributable cost, and total direct medical cost estimates for 2011^a^

HAIs under traditional surveillance	Central burden 2011	Low burden 2011	High burden 2011	2011 LOS (days)	2011 Attributable costs	2011 Attributable costs (2017$)	Total cost central 2011 (2017$)	Total cost low 2011 (2017$)	Total cost high 2011 (2017$)
Ventilator-associated pneumonia^b^	61,600	19,900	194,100	13.92	$44,449	$48,543	$2,990,257,629	$966,008,552	$9,422,224,120
Surgical-site infection	157,500	50,800	496,500	7.68	$22,451	$24,519	$3,861,785,995	$1,245,579,229	$12,173,820,614
*Clostridium difficile*^b^	87,300	27,200	281,600	6.39	$14,826	$16,191	$1,413,481,782	$440,397,531	$4,559,409,734
Catheter-associated urinary tract infections^b^	63,200	19,000	211,100	5.24	$11,724	$12,804	$809,201,758	$243,272,680	$2,702,887,518
Primary bloodstream infection	71,900	20,700	247,400	8.23	$23,306	$25,452	$1,830,017,661	$526,861,830	$6,296,889,699
Subtotal	441,500	137,600	1,430,700				$10,904,744,825	$3,422,119,823	$35,155,231,684
Non-device and non-procedure related HAIs									
Pneumonia^b^	95,900	30,900	302,400	5.07	$13,384	$14,617	$1,401,729,500	$451,652,154	$4,420,052,145
Gastrointestinal infection^b^	35,800	11,200	115,600	4.33	$14,687	$16,040	$574,235,374	$179,649,056	$1,854,234,895
Urinary tract infection^b^	30,100	9100	100,700	4.14	$8782	$9591	$288,675,713	$87,274,053	$965,768,914
Eye, ear, nose, throat, or mouth infection	40,200	10,400	151,500	2.77	$7015	$7661	$307,970,309	$79,673,911	$1,160,634,373
Lower respiratory tract infection	28,500	6900	115,000	3.83	$11,301	$12,342	$351,750,222	$85,160,580	$1,419,342,999
Skin and soft-tissue infection	22,700	5200	97,500	4.40	$9815	$10,719	$243,316,506	$55,737,702	$1,045,081,910
Cardiovascular system infection	8400	1200	47,000	9.00	$25,372	$27,709	$232,754,584	$33,250,655	$1,302,317,315
Bone and joint infection	7100	1000	41,800	7.74	$18,133	$19,804	$140,604,924	$19,803,510	$827,786,734
Central nervous system infection	5800	700	36,600	7.49	$24,133	$26,356	$152,862,645	$18,448,940	$964,616,001
Reproductive tract infection	4500	500	31,400	3.19	$7621	$8323	$37,453,067	$4,161,452	$261,339,177
Systemic infection	1300	0	19,200	3.21	$8703	$9504	$12,355,642	$0	$182,483,326
Subtotal	280,300	77,100	1,058,700				$3,743,708,485	$1,014,812,012	$14,403,657,790
All infections	721,800	214,700	2,489,400				$14,648,453,310	$4,436,931,835	$49,558,889,474

*LOS* length of stay

^a^All estimates have been rounded to the nearest 100th unit to be consistent with estimates reported in the study by Magill et al. [4]

^b^While the Magill studies do not directly report burden estimates for ventilator-associated pneumonia, *Clostridium difficile* infections, and catheter-associated urinary tract infection, we used the reported proportions for ventilator-associated pneumonias to all pneumonias, catheter-associated urinary tract infections to all urinary tract infections, and *Clostridium difficile* infections to all gastrointestinal infections to obtain separate burden estimates for these infections. These respective proportions were: (1) 39.1% in 2011 for ventilator-associated infections to all pneumonias, (2) 67.7% in 2011 for catheter-associated urinary tract infections to all urinary tract infections, and (3) 70.9% in 2011 for *Clostridium difficile* infections to all gastrointestinal infections

*Note*: the 2011 *Clostridium difficile* burden estimates reported in Table 4 of Magill et al. [4] were based on a case definition similar to the NHSN laboratory-identified event definition for *Clostridium difficile* cases. This results in a smaller number of identified cases than the prevalence survey case definition for Clostridium difficile infections. As the 2015 *Clostridium difficile* burden estimates found in Magill et al. [5] were based on the prevalence survey case definitions, we calculated *Clostridium difficile* burden for 2011 on the basis of the prevalence survey case definition to be consistent with the 2015 results. On the basis of this definition, the proportion of *Clostridium difficile* infections to gastrointestinal infections is 70.9%, which results in central, low, and high estimates of 87,300, 27,200, and 281,600, respectively

### Estimation of the National Direct Medical Costs of Hospital-Onset Infections

Tables 5 and 6 include the 2011 and 2015 incidence estimates (including a low, central, and high estimates), LOS, the unadjusted (2011 and 2016) and inflation adjusted (2017) attributable costs, the calculation of the total attributable cost associated with each infection, and the total sum of all attributable direct medical costs. In 2011, the total cost of HOI based on the sum of the central incidence estimates was $14.6 billion and ranged from $4.4 billion (based on the low estimates) to $49.6 billion (based on the high estimates) when adjusted to 2017 dollars. The economic cost of HOI decreased to $12.1 billion (with a low of $3.3 billion and a high of $46.9 billion) mostly because of overall decline in HOIs from 2011 to 2015.^10^ Nondevice- and nonprocedure-associated HOIs comprised approximately 26–28% of total HOI costs using the central estimates ($3.7 billion in 2011 and $3.4 billion in 2016, both in 2017 dollars).

**Table 6:** Burden, LOS, attributable cost, and total direct medical cost estimates for 2016^a^

HAIs under traditional surveillance	Central burden 2015	Low burden 2015	High burden 2015	2015 LOS	2016 Attributable costs	2016 Attributable costs (2017$)	Total cost central 2016 (2017$)	Total cost low 2016 (2017$)	Total cost high 2016 (2017$)
Ventilator-associated pneumonia^b^	62,700	18,200	220,700	12.47	$39,124	$40,009	$2,508,545,823	$728,158,437	$8,829,921,263
Surgical-site infection	110,800	30,200	411,200	8.42	$23,174	$23,698	$2,625,749,064	$715,682,506	$9,744,657,174
*Clostridium difficile*^b^	106,100	29,900	381,400	5.52	$12,339	$12,618	$1,338,764,859	$377,276,808	$4,812,487,440
Catheter-associated urinary tract infections^b^	38,600	9,600	155,300	5.26	$12,060	$12,333	$476,044,398	$118,394,462	$1,915,277,074
Primary bloodstream infection	83,600	21,800	321,300	7.36	$20,607	$21,073	$1,761,680,926	$459,385,696	$6,770,670,830
Subtotal	401,800	109,700	1,489,900				$8,710,785,070	$2,398,897,909	$32,073,013,781
Non-device and non-procedure related HAIs									
Pneumonia^b^	114,000	33,000	400,900	4.73	$11,599	$11,861	$1,352,166,271	$391,416,552	$4,755,118,054
Gastrointestinal infection^b^	40,200	11,400	144,600	4.63	$15,643	$15,996	$643,042,318	$182,355,284	$2,313,032,815
Urinary tract infection^b^	24,100	6000	97,200	3.89	$8688	$8884	$214,105,420	$53,304,254	$863,528,915
Eye, ear, nose, throat, or mouth infection	33,600	7300	151,100	2.99	$8208	$8394	$282,029,528	$61,274,272	$1,268,293,503
Lower respiratory tract infection	29,100	6000	133,900	4.24	$12,013	$12,285	$357,486,033	$73,708,460	$1,644,927,143
Skin and soft-tissue infection	35,500	7800	156,800	4.61	$11,113	$11,364	$403,438,960	$88,642,927	$1,781,950,113
Cardiovascular system infection	1900	0	23,000	7.92	$22,413	$22,920	$43,547,605	$0	$527,155,221
Bone and joint infection	3200	200	32,500	6.77	$16,934	$17,317	$55,414,465	$3,463,404	$562,803,163
Central nervous system infection	1900	0	23,000	7.58	$25,861	$26,445	$50,246,030	$0	$608,241,411
Reproductive tract infection	1900	0	23,000	3.61	$9647	$9865	$18,743,644	$0	$226,896,744
Systemic infection	0	0	15,300	6.01	$15,456	$15,805	$0	$0	$241,817,008
Subtotal	285,400	71,700	1,201,300				$3,420,220,276	$854,165,154	$14,793,764,090
All infections	687,200	181,400	2,691,200				$12,131,005,346	$3,253,063,063	$46,866,777,870

*LOS* length of stay

^a^All estimates have been rounded to the nearest 100th unit to be consistent with estimates reported in the studies by Magill et al. [5]

^b^While the Magill studies do not directly report burden estimates for ventilator-associated pneumonia, *Clostridium difficile* infections, and catheter-associated urinary tract infection, we used the reported proportions for ventilator-associated pneumonias to all pneumonias, catheter-associated urinary tract infections to all urinary tract infections, and *Clostridium difficile* infections to all gastrointestinal infections to obtain separate burden estimates for these infections. These respective proportions were: (1) 35.5% in 2015 for ventilator-associated infections to all pneumonias, (2) 61.5% in 2015 for catheter-associated urinary tract infections to all urinary tract infections, and (3) 72.5% in 2015 for *Clostridium difficile* infections to all gastrointestinal infections

To evaluate the drivers of the cost differences between 2011 and 2016, we compared LOS estimates across infections. The estimates for LOS showed minimal or modest declines for pneumonia, ventilator-associated pneumonia, *Clostridioides difficile*, urinary tract infections, primary bloodstream infections, skin and soft-tissue infections, cardiovascular system infections, and bone and joint infections. The estimates increased for gastrointestinal infections; surgical-site infections; ear, eye, nose mouth, and throat infections; central nervous system infections; lower respiratory tract infections; reproductive tract infections; and systemic infections. For catheter-associated urinary tract infections, LOS estimates were essentially the same. The two infection types for which LOS estimates significantly changed included ventilator-associated pneumonia (a decrease from 14 days in 2011 to 12.5 days in 2016) and systemic infections (an increase from 3 days in 2011 to 6 days in 2016).

The inflation adjusted estimates of attributable costs showed a similar pattern. Cost estimates between 2011 and 2016 decreased for pneumonia, ventilator-associated pneumonia, *Clostridioides difficile,* primary bloodstream infections, cardiovascular system infections, urinary tract infections, and bone and joint infections. Attributable cost estimates have increased for gastrointestinal infections, including eye, ear, nose, throat, and mouth infections; skin and soft tissue infections; lower respiratory tract infections; surgical site infections; systematic infections; central nervous system infections; and reproductive tract infections. The cost estimates with the largest changes included ventilator-associated pneumonia (a decrease from $48,500 in 2011 to $40,000 in 2016) and systemic infections (an increase from $9500 in 2011 to $16,000 in 2016).

## Discussion

### Summary

We found that the total annual direct medical costs owing to HOIs were approximately $14.6 billion in 2011 and $12.1 billion in 2016 (in 2017 dollars) for the 16 infection types assessed in the simulation. While the majority of the estimated total costs comprised *Clostridioides difficile* infections along with device-associated and procedure-associated infections, the costs associated with nondevice and nonprocedure related infections comprised approximately 28% of the total costs, with nonventilator associated pneumonia accounting for 40% of non-device and nonprocedure costs alone.

To assess the credibility of our attributable cost estimates, we compared four of our estimates with those derived from two published meta-analyses conducted by Zimlichman et al. and Tyler et al. (ventilator-associated pneumonia, surgical-site infections, *Clostridioides difficile* infections, and catheter-associated urinary tract infections) [6, 70]. To make a valid comparison, we adjusted all attributable cost estimates from both sources to 2017 dollars, using the producer price index for general medical and surgical hospitals (PUC #62211) [56]. These adjusted estimates were then applied to the central incidence estimates reported by Magill et al. for 2011 (from Table 5) [4]. Supplementary Appendix A Table 2 presents the differences in the adjusted attributable cost estimates and total attributable costs across studies. Our estimates for each infection were between the Zimlichman study estimates (lower) and the Tyler study estimates (higher). Using only the mean (center) attributable cost estimates from each study, the total direct medial cost estimates were $10.1 billion (Tyler et al.), $7.2 billion (Zimlichman et al.), and $8.7 billion from this study. In percentage terms, our total cost estimate was approximately 86% of the Tyler study estimate and approximately 21% higher than the cost estimate based on the Zimlichman study. While there are differences in the attributable cost estimates for the four infections compared with other studies, the total economic costs for this subgroup of infections, as estimated by our study, produced an estimate that is not substantially different from cost estimates produced by other methods.

### Advantages

Our analogy costing approach has several advantages over traditional approaches in measuring the attributable costs of HOIs. First, as we relied on principal diagnosis codes to identify hospitalizations with an infection, there is greater certainty that the costs associated with these hospitalizations reflect the value of resources used for treatment, as the accuracy of coding for the principal diagnosis in administrative data has been shown to be 80–85% (high specificity) [71, 72]. Second, the cost-to-charge ratio records for converting charges to costs are based on the cost accounting framework used by the Centers for Medicare and Medicaid Services in their hospital cost reporting system, thereby avoiding potential measurement bias of patients costs that can result from using data generated from single or multicenter hospital studies with differing cost accounting systems [46]. With our case definitions and the use of NIS, we identified thousands of cases for each infection, which reflected the range of variability in patient treatment and demographics and enhanced the statistical precision in our regression models. Third, the cost models can be adjusted for geographic cost differences using wage indexes that Medicare calculated for each core-based statistical area (CBSA) [73]. Using the 2016 LRTI simulation model from Fig. 1 and wage index data for FY2016, setting Wage_Index to 1.127 (the wage index associated with the CBSA for Washington State), then running the remaining data through the model results in attributable costs of $12,587 for LRTI for ($574 greater than the 2016 estimate of $12,013; from Table 6). This kind of analysis presumes that hospitalizations with LRTIs in NIS can be used to reflect LRTI patients in Washington State.

### Limitations

Our study has potential limitations. An overarching limitation on all the results stems from the inherent biases that underly the use of administrative datasets and the presence of upcoding [74–81]. Given that the cost/charge data in NIS suffers from measurement error, we used median regression to estimate all cost models to minimize the effect of outliers in the cost data. However, some cost outliers are actually accurate. To the extent that this is true, our attributable costs estimates are biased downward.

Second, we relied on expert opinion in creating catalogs of diagnosis codes to identify hospitalizations with infection, which may lead to misclassification of hospitalizations with either a primary or secondary diagnosis. Further research is needed to validate the coding to assess the specificity and sensitivity of patients with an infection. Our cohort of hospitalizations with infection as a secondary diagnosis contained hospitalizations that may not have been an actual HOI, resulting in possible misclassification bias. We have excluded hospitalizations that had a LOS of less than 3 days to help mitigate this bias.

Third, our use of parameters from multivariate median regression model parameters as cost analogs to predict attributable HOI costs may be problematic if multicollinearity between independent variables is present. While multicollinearity has been shown to not affect model predictions or overall fit in linear models, little is known on how multicollinearity affects predictions from median regression models [82, 83]. However, the diagnostics that we perform on our models did not detect the presence of multicollinearity. Additionally, administrative datasets, such as NIS, lack detailed clinical information that can be used to better identify and track the resources used to treat both the HOI and the underlying disease [21, 84]. Thus, our estimated median regression cost models (stage 1) may not accurately capture any economies or diseconomies of scope that potentially exist in a multiproduct producing, firm such as a hospital.

Fourth, there are additional cost to HOIs that are not captured in the NIS dataset. The cost data in NIS does not include professional fees of physicians and more broadly excludes follow-up outpatient visits bill to Medicare Part B [85]. Including those fees would appreciably increase the costs of HOIs [85]. While there is not a published adjustment ratio for 2016, the published adjustment ratios for commercially insured patients and for Medicaid patients for 2011 were 1.269 and 1.143, respectively. Applying a crude upward adjustment of 15% for all hospitalizations, our central estimates of total HOI direct medical cost would increase to $16.8 billion (2011) and $13.9 billion (2016). Additional healthcare resources are also needed to treat any short-term or long-term morbidities owing to a HOI that involves a range of healthcare providers after hospital discharge, including additional inpatient care, skilled facility care, outpatient visits, home health assistance, and other noninstitutional services [29].

Finally, our method attempts to disentangle the confounding impacts between acquiring an infection, other comorbidities, and patient severity of illness on attributable cost of HOIs. Studies comparing hospital costs for patients with COIs and HOIs have found that attributable infection costs were larger for HOIs [86–88]. Hospitalized patients with greater disease severity, particularly those requiring admission to an intensive care unit, have been shown to have a greater risk of acquiring a HOI during their stay [89, 90]. Resource use is usually greater for these patients as they require greater utilization of medical devices and/or procedures that increases their infection risks, particularly when patients are immunocompromised, have diabetes mellitus, and need mechanical ventilation [89, 90]. While our models contain NIS variables reflecting the degree that severity of illness and the risk of mortality affect the model, further research is needed to see whether our cost analogues may potentially overstate/understate the impact of the severity of patients’ underlying disease and on infections and attributable costs. This implies that our cost estimates can potentially have an upward or downward bias on our attributable cost estimates.

Despite these issues, the design of our study attempts to minimize the impact of bias on our results. First, research on the quality of LOS data in administrative dataset has found these data to be highly reliable [91, 92]. While we used median regression to obtain cost coefficients that dampens the impact of outliers and possible upcoding, their combined use in the simulation probably produced results that were more centered and less biased, either upward or downward. Second, our statistical and simulation cost models were specified with variables available in the NIS that could represent economies (cost savings) or diseconomies (higher costs) of scope that may result from the joint cost of production in hospitals. A comparison of the univariate statistics between the three patient groups for cost suggests that using costs associated with patients with infections as the principal diagnosis may understate HOI costs, as there are diseconomies of scope for treating patients with an infection as a secondary diagnosis in addition to their underlying disease or principal diagnosis (see Supplementary Appendix A, Section III for more discussion).

Although the direct medical costs of HOIs are substantial, they represent a small proportion of the overall social cost of HOIs. Additional costs faced by patients include lost wages and/or diminished worker productivity, particularly if patients suffer long-term morbidities, and the additional economic value society would pay to reduce the risk of morbidity and mortality [93, 94]. The societal willingness to pay to avoid the annual risk of disease and death owing to *Clostridioides difficile* infections alone has been estimated to be $166 billion [95].

## Conclusions

We applied an analogy costing methodology to estimate the direct medical cost of HOIs as an alternative to more typical analytical methods, such as meta-analysis and retrospective cohort studies. We have generated more comprehensive national estimates of the direct medical cost of HOI, which will be of interest to policy makers. Our methodology may be of interest to health services researchers, as our analysis raises the question of whether epidemiological methods designed to assess disease risk can be used to assess differences in resource use and cost. Lacking disease classifications codes to identify HOIs, we used costs associated with patients who had an infection as the principal diagnosis, as the treatment costs and the associated cost relationships to other patient characteristics more accurately reflect the direct medical cost to treat infections. Using these costs relationships in the simulation models, we can estimate the attributable costs associated with HOIs. Our method also generated cost estimates for infections that are not associated with a device or procedure that have rarely been measured.

**CDC Disclaimer** The findings and conclusions in this report are those of the authors and do not necessarily represent the official position of the Centers for Disease Control and Prevention.

## Supplementary Information

Below is the link to the electronic supplementary material.

Supplementary file1 (DOCX 483 KB)

## Footnotes

1 Various cost systems include traditional costing, activity-based costing, time-driven activity-based costing, and performance-focused activity-based costing [16].

2 The types of infection include: ventilator-associated pneumonia; surgical-site infection; *Clostridium difficile*; catheter-associated urinary trat infections; primary bloodstream infection; pneumonia; gastrointestinal infection; urinary tract infection; eye, ear, nose, throat, or mouth infection; lower respiratory tract infection; skin and soft-tissue infection; cardiovascular system infection; bone and joint infection; central nervous system infection; reproductive tract infection; and systemic infection.

3 The observations in the NIS represent hospitalization events, as opposed to unique patients, and thus contain observations of patients with multiple hospitalizations [47].

4 In 2011, NIS consisted of 100% of the discharges from 20% of US hospitals. However, in 2016, the NIS consisted of a 20% national patient-level sample, with nonrepresentative sampling across hospitals. While there are different discharge trend weights that have been developed for the different sample designs, our analysis does not involve discharges or assessing trends, only (1) the charges/costs associated with discharges with infections as a principal diagnosis and (2) the data from discharges with infections as a secondary diagnosis for use in our 2011 and 2016 attributable cost simulation models [52, 53].

5 In addition to the 2011 and 2015 prevalence surveys, another survey was scheduled to take place in 2020. However, this study was delayed to 2023 owing to the coronavirus disease 2019 (COVID-19) pandemic. Because of the substantial impact of the pandemic on the incidence of selected HOIs, we have chosen not to adjust cost estimates to 2023 prices pending an updated economic analysis using the 2023 incidence estimates.

6 In NIS, the principal diagnosis is defined as “the condition established after study to be chiefly responsible for the patient’s admission to the hospital. Secondary diagnoses are conditions that coexist at the time of admission that require or affect patient care treatment or management, or that develop during the inpatient stay” [57].

7 The incidence estimates for 2015 [5] can be found in the paper’s supplementary appendix (Table S10) located at: https://www.nejm.org/doi/suppl/10.1056/NEJMoa1801550/suppl_file/nejmoa1801550_appendix.pdf.

8 The APR-DRG severity of illness and APR-DRG risk of mortality indexes are part of NIS and were created using propriety software belonging to the 3M Health Information Systems [65]. The severity of illness index refers to overall patient illness severity to the extent of physiological decomposition or loss of organ system function, while risk of mortality estimates the likelihood of dying [66]. The two measures were designed to be independent of each other [67]. These measures are calculated separately in the software mostly on the basis of secondary diagnoses and their interaction principal diagnosis and age. While these indexes were created as categorical variables, other studies have carried out analyzes using the indexes as a continuous scales [68, 69].

9 The hypothesized relationship can be expressed as: (Cost_patients with infection as a principal diagnosis_ ⋚ Cost_patients with no infection_ < Cost_patients with infection as secondary diagnosis_).

10 We evaluated two alternative median regression and attributable costs model specifications to assess any impact on our total cost predictions in 2016. First, we substituted a categorical variable for age and a dummy variable for sex. Second, we used the APR-DRG severity of illness and mortality risk indexes as categorical variables (as they were originally conceived). The revised specifications produced only minor changes in our total cost predictions (+0.43% for the former, −2,5% for the latter).

## Declarations

### Availability of Data and Material

The National Inpatient Sample is a publicly provided dataset available from the Healthcare Cost Utilization Project (HCUP) sponsored by the Agency for Healthcare Research and Quality. Our disease coding algorithms are available in the Supplementary Appendix B.

### Code Availability

Data were analyzed using SAS version 9.4 software.
