# Supplementary material for: Measuring the Direct Medical Costs of Hospital-Onset Infections Using an Analogy Costing Framework
Source: Pharmacoeconomics. 2024 Jul 5;42(10):1127–44. doi: 10.1007/s40273-024-01400-z (PMC11405445; doi:10.1007/s40273-024-01400-z)
Supplement: Supplementary file 1 — Supplementary file1 (DOCX 483 KB) [file 40273_2024_1400_MOESM1_ESM.docx]

**Supplementary Information**

**Article title**

Measuring the Direct Medical Costs of Hospital-Onset Infections Using an Analogy Costing Framework

**Journal name**

PharmacoEconomics - Open

**Author names**

R. Douglas Scott II, Steve D. Culler, James Baggs, Sujan C. Reddy, Kara Jacobs Slifka, Shelley S. Magill

Sophia V. Kazakova, John A. Jernigan, Richard E. Nelson, Robert E. Rosenman, Philip R. Wandschneider

**Corresponding author**

Doug Scott, US Centers for Disease Control and Prevention, 1600 Clifton Road,

MS H16-3, Atlanta, GA 30329-4027.

ORCID ID: 0000-0001-5364-3059

E-mail address: DScott1@cdc.gov

**Appendix A**

**Section I. Formal Total Cost Model**

Our overall objective is to produces estimates of the total cost of HOIs to the U.S. healthcare system for 2011 and 2015. The specified total cost model (TDMC) is given as:

(1) $Total Cost=\sum_{i=1}^{16} {(HAI}_{i}* {Attributable HAI Cost}_{i})$ $\mathrm{TDMCj}=\sum_{i=1}^{16} {(HOI}_{i}* {Attributable HOI Cost}_{i})$;

where:

j = (2011,2015)

i = (1,2, ……,16),

HOI_i_ = the estimated incidence of each hospital-onset infections,

Attributable HOI Cost_i_ = attributable costs of patient care for each infection type.

**Section II. Simulation Model Measuring the Attributable Costs of HOI**

The variables taken from the National Inpatient Sample dataset for 2011 and 2015 used in this analysis are presented in Appendix A Table 1 [1]. Development of the simulation models proceeded in two stages. For the first stage, we estimated median regression models, using total costs of patients with an infection as a primary diagnosis as the dependent variable, for each type of HOI resulting in 32 separate models from the two study years. The specified form for all stage 1 regression cost models included the same set of variables for each infection type, that serves as predictors of patient cost and resource use consistent with the economic theory of quality control and the cost of quality analysis. These variables included LOS, number of diagnoses, number of procedures, patient age (to control for the impact of aging on resource use) , assessment of severity of underlying and the risk of mortality; and data on hospital characteristics including small hospital bed size indicator (250 beds or less), an urban teaching hospital indicator, and the Centers for Medicare and Medicaid Services’ wage index (see Appendix A Table 1 for NIS variable descriptions) [2]. We used the same minimal set of variables across the 16 infection types as they best represented information related to resouse use and their valuation, run less risk of model overfitting, and were available in both study years.

To identify an appropriate statistical approach to model this data, we tested whether the distribution of cost and LOS were skewed. Employing White’s test for heteroscedasticity, we found that the distribution of cost and LOS was highly skewed with a number of cost outliers that represented hospital stays for over a million

**Appendix A Table 1; Description of NIS Variables and Variable Transformations for Attribtable Cost Simulation Models**

| **Variables** | **Description** | **Type / Category** | **Response Category** |
| --- | --- | --- | --- |
| **Dependent Variable:** |  |  |  |
| **Total Patient Costs (Costs)** | **Charges adjusted by Cost to Charge Ratio** | **Numeric** |  |
| **Explanatory Variables:** |  |  |  |
| **Numeric Variables (NIS)** |  |  |  |
| **LOS** | **Length of Hospital Stay** | **Numeric** |  |
| **NDX (I10_NDX for 2016 NIS)** | **Number of Diagnosis Codes** | **Numeric** |  |
| **NPR (I10_NPR for 2016 NIS)** | **Number of Procedures Codes** | **Numeric** |  |
| **AGE** | **Age** | **Numeric** |  |
| **Wage_Index** | **CMS Wage Index [2]** | **Numeric** | **Developed by CMS to measure the relative hospital wage level in a core-based statistical areas**  **(CBSA) compared with the**  **national average hospital**  **wage level** |
| **Categorical Variables** |  |  |  |
| **HOSP_BEDZ (NIS)** | **Bed size of hospital** | **1** | **Small** |
|  |  | **2** | **Medium** |
|  |  | **3** | **Large** |
|  |  | **.** | **Missing** |
| **SMALL_BEDSIZE_HOSPITAL (New)** | **Collapsed dummy in model** | **1** | **Small** |
|  |  | **0** | **Medium+Large** |
| **HOSP_LOCTEACH (NIS)** | **Location/teaching status of hospital** | **1** | **Rural** |
|  |  | **2** | **Urban nonteaching** |
|  |  | **3** | **Urban teaching** |
|  |  | **.** | **Missing** |
| **URBAN_TEACHING_HOSPITAL (New)** | **Collapsed dummy in model** | **1** | **Urban_teaching** |
|  |  | **0** | **Urban nonteaching + Rural** |
| **APRDRG_Severity_Index (NIS)^1^** | **All Patient Refined DRG: Severity of Illness Subclass** | **0** | **No class specified** |
|  |  | **1** | **Minor loss of function (includes cases with no comorbidity or complications)** |
|  |  | **2** | **Moderate loss of function** |
|  |  | **3** | **Major loss of function** |
|  |  | **4** | **Extreme loss of function** |
|  |  | **.** | **Missing** |
| **APRDRG_Risk_Mortality_Index (NIS)^1^** | **All Patient Refined DRG: Risk of Mortality Subclass** | **0** | **No class specified** |
|  |  | **1** | **Minor likelihood of dying** |
|  |  | **2** | **Moderate likelihood of dying** |
|  |  | **3** | **Major likelihood of dying** |
|  |  | **4** | **Extreme likelihood of dying** |
|  |  | **.** | **Missing** |

**NIS, National Inpatient Sample; CMS, Centers for Medicare and Medicaid Services; APRDRG, All Patient Refined Diagnosis Group**

^1^ **APRDRG_Severity_Index and APRDRG_Risk_Mortality_Index are used as continuous variables in the median regression analysis.**

Dollars [3,4]. Given the potential for measurement bias from inappropriate ICD-9 or ICD-10 coding of secondary diagnoses (upcoding) and the presence of outliers in the data, we employed median regression (as opposed to ordinary or generalized linear regression) to model each infection with the realization that the generated estimates produced from this model will result in lower, and thus conservative, attributable cost estimates [5-13]. An example of the 2011 and 2016 median regression estimations for LRTI is presented in the results section in the paper while all results for all other models are found in Appendix B of this document.

Figure 1 (in paper) illustrates the steps to develop the conceptual simulation model using LRTIs in 2011 as an example. The estimated parameters from the stage 1 regression are used to parameterize the coefficients on the same variables in the stage 2 simulation model. As these stage 1 parameters capture the relationships between costs and model variables for patients that are more likely to have an actual infection (accuracy of primary diagnosis codes being high), the more accurately these parameters disentangle the attributable cost of infection from total costs of patients that have an infection as a secondary diagnosis.

To calculate the attributable costs of LRTIs, we entered the data for patients with a LRTI as a secondary diagnosis into the stage 2 equation, with one exception. Instead of using the recorded LOS observation for each of the patients in the secondary groups (2011 and 2015), we replaced it with the average LOS for patients in the principal groups (3.83 days for 2011 and 4.24 days for 2015) for each patient in the secondary group. These values serve as the analog for the attributable LOS due to the HOI in the simulation model. The other model parameters also served as analogues (surrogates) for the attributable costs of a hospital-onset LRTI that resulted from the changes in the remaining variables (attributable impact on the number of diagnosis, number of procedures, etc.) which were calculated by multiplying the associated parameters (from the median regressions) with the associated NIS data for the patients with an LRTI diagnosis code as a secondary diagnosis. The average of the attributable cost estimates from each patient was then calculated to be used with the LRTI disease estimates to calculate the direct medical cost associated with LRTI (formula 1). Simulations models were similarly run for each of the other HOI types for 2011 and 2016, resulting in a total of 32 separate attributable HOI cost estimates. The HOI attributable cost estimates were multiplied by the corresponding incidence estimate to calculate the total costs of HOIs to the healthcare system for both 2011 and 2016 (see equation 1). The simulation models for each of the remaining 15 infections in each year were developed using the same approach.

**Section III. Justification For Empirical Model**

The analytical challenge to measuring the attributable cost associated with a HOI is being able to separate the costs for those resources used to treat the underlying disease from the cost of the resources used to treat the HOI. An implicit cost function for the total hospital cost of treatment for patients having a specific condition and one (or more) of the HOIs can be expressed as:

1. C(U_1_,U_2_,…..,U_n_,HOI_1_,HOI_2_,…..HOI_16_;X_1_,X_2_,…..,X_n_)

where

U_1_,U_2_,…..,U_n_ = treatment process for the 1 through n underlying conditions (or reasons for hospitalization),

HOI_1_,HOI_2_,…..,HOI_16_ = prevention and treatment process for each of the 16 hospital onset infections, and

X_1_,X_2_,…..,X_n_ = the unit prices for the 1 through n inputs used across all processes.

This cost function is comprised of multiple production processes, one for each U and one for each HOI. If the production process for treating any U involves no interaction between the individual inputs and the output levels of any of the prevented HOIs (non-joint production), then the cost function is “separable” (assuming that there is sufficient clinical data to distinguish the amount of resources used in each process) [14]. In this case, equation 1 can be expressed as the sum of two separate cost functions as shown by

1. C(U_1_,U_2_,…..,U_n_,HOI_1_,HOI_2_,…..HOI_16_;X_1_,X_2_,…..,X_n_) = C(U_1_,X_1_,X_2_,…..,X_n_) +…..+ C(U_n_;X_1_,X_2_,…..,X_n_)+ C(HOI_1_;X_1_,X_2_,…..,X_n_) + ….. + C(HOI_16_;X_1_,X_2_,…..,X_n_)

As we are only interested in developing estimates of the attributable costs for each of the separate HOIs [C(HOI_1;_X_1_,X_2_,…..,X_n_) + ….. + C(HOI_16;_X_1_,X_2_,…..,X_n_)], we used the total cost associated with patients who had an infection, not as an HOI, but as their principal diagnosis, as a surrogate (analogue) based on our analogy costing measurement strategy. The empirical attributable cost model for LRTI to be estimated from the NIS datasets is:

1. C(HOI_LRTI_;X_1_,X_2_,…..,X_n_) = Intercept + β_1,LRTI_*LOS + β_2, LRTI_*ndx + β_3,LRTI_*npr + β_4, LRTI_ *urban_teaching + β_4,LRTI_*small_bedsize + β_5,LRTI_*age + β_6,LRTI_*APRDRG_Severity + β_7,LRTI_*APRDRG_Risk_Mortality + β_8,LRTI_*Wage_Index + e.

This same specification is used for all other infection types.

The formulation of total patient costs in equation 2 may overstate actual costs as multi-product firms like hospitals can achieve cost savings by treating a number of conditions (or diseases) together (joint production). As there are common inputs used in both processes (physician consults, nursing care, laboratory test, beds, etc.) and the firm’s fixed cost can be spread out across multiple products (treatments), this gives rise to economies of scope (costs savings associated with treating a diverse group of conditions). In this case, the cost function is not separable and the cost of joint production is less.

1. C(U_1_,U_2_,…..,U_n_,HOI_1_,HOI_2_,…..HOI_16_;X_1_,X_2_,…..,X_n_) < C(U_1_,X_1_,X_2_,…..,X_n_) +…..+ C(U_n_;X_1_,X_2_,…..,X_n_)+ C(HOI_1_;X_1_,X_2_,…..,X_n_) + ….. + C(HOI_16_;X_1_,X_2_,…..,X_n_)

However, HOIs are considered medical errors and can result in diseconomies of scope (extra costs associated with preventing or treating infections across a diverse group of patient conditions and severity of disease) resulting in higher costs for treating both the underlying disease and the infection. An examination of the univariate statistics for LRTI in Table 3 (in main paper) provide evidence that these diseconomies exist for potential cases with HOI. When looking at costs across the three patient groups, we see the cost (secondary group) is similar or slightly greater to the cost (principal group) + cost (no infection group) at the median, but for the mode and at the mean, cost (secondary group) tend to be greater than cost (principal group) + cost (no infection group). It appears to be the case for most infection types, where on average, there are scope diseconomies between HOI and other ailments, especially for severe cases, although it disappears at the median.

If we look at LOS for production returns to scope, LOS (secondary) exceeds LOS (principal)+LOS (no infection) only at the mean (and only for a small number of infection types) indicating that this difference is being driven by some outliers at the upper tail of the LOS distribution. For the other infection types, there are very slight increasing production returns to scope in terms of LOS (more ailments, less additional time added). In terms of overall care, this would indicate that those with a HOI on top of their principal diagnosis have a confounding treatment plan that requires more medical intervention that is greater than what is given for those who have the infection as the principal diagnosis, but which can be done over some of the same time period as the care for the principal diagnosis. To the extent room costs are a share of total hospital cost, we over-estimate the cost of HOI. However, the evidence from the cost comparisons indicates that those with HOI must need more medical care (over and above treatment for the principal diagnosis) than those with infection as the principal diagnosis.

While it is highly unlikely that the diseconomies of scope due to HOIs outweigh the economies of scope from the joint production of the treatment of multiple diseases, evidence from our group comparisons suggests that using the cost of infection as a principal diagnosis to estimate the cost of treating HOI is a strong lower bound, and thus a conservative estimate of the attributable costs of HOIs.

**IV. Comparison of Estimates From Previous Studies**

Appendix A Table 2 presents attributable costs and total costs estimates from studies by Tyler at al., Zimlickman et al, and 2011 attributable cost estimates from the present study for four infection types.

**Appendix A Table 2: Comparison of Cost Estimates to Previous Estimates**

|  | **Magill et al. (2011)^4^** | **Tyler et al. (2017)^55^** | | **Zimlichman et al. (2012)^6^** | | **Present Study** | |
| --- | --- | --- | --- | --- | --- | --- | --- |
| **Infection Type** | **2011 burden** | **Attributable Cost (2017$)** | **Direct medical Cost Estimates** | **Attributable Cost (2017$)** | **Direct medical Cost Estimates** | **Attributable Costs for 2011 (2017$)** | **Direct medical Cost Estimates for 2011** |
| **Ventilator-Associated Pneumonia** | **61,600** | **$49,100** | **$3,024,560,000** | **$42,758** | **$2,633,903,128** | **$48,543** | **$2,633,892,800** |
| **Surgical-Site Infections** | **157,500** | **$29,331** | **$4,619,632,500** | **$22,139** | **$3,486,815,990** | **$24,519** | **$3,861,785,995** |
|  |  |  |  |  |  |  |  |
| ***Clostridium difficile*** | **87,300** | **$17,940** | **$1,566,162,000** | **$12,040** | **$1,051,062,318** | **$16,191** | **$1,413,481,782** |
| **Catheter-associated urinary tract infection** | **63,200** | **$14,337** | **$906,098,400** | **$954** | **$60,314,750** | **$12,804** | **$809,201,758** |
| **Total** | **369,600** |  | **$10,116,452,900** |  | **$7,232,096,186** |  | **$8,718,362,335** |

**Appendix B: ICD-9 and ICD-10 Infection Code Tables, Univariate Statistics, and Cost Model Specifications**

**Bone and Joint Infections (BJI)**

| **Appendix B Table 1-BJI: IDC-9 and ICD-10 Codes for Bone and Joint Infections** | | | |  |
| --- | --- | --- | --- | --- |
| **CD-9 Description** | **ICD-9_bji** | **ICD-10_bji** | | **ICD-10 Description** |
| Intraspinal abscess | 3241 | G061 | | Intraspinal abscess and granuloma |
| Inflammatory conditions of jaw (includes abscess of hard palate, jaw, mandible…) | 5264 | M272 | | Inflammatory conditions of jaws |
| Pyogenic arthritis | 71100 | M0000 | | Staphylococcal arthritis, unspecified joint |
| Pyogenic arthritis | 71100 | M0010 | | Pneumococcal arthritis, unspecified joint |
| Pyogenic arthritis | 71100 | M0020 | | Other streptococcal arthritis, unspecified joint |
| Pyogenic arthritis | 71100 | M0080 | | Arthritis due to other bacteria, unspecified joint |
| Pyogenic arthritis | 71100 | M009 | | Pyogenic arthritis, unspecified |
| pyogenic arthritis, shoulder region | 71101 | M00011 | | Staphylococcal arthritis, right shoulder |
| pyogenic arthritis, shoulder region | 71101 | M00012 | | Staphylococcal arthritis, left shoulder |
| pyogenic arthritis, shoulder region | 71101 | M00019 | | Staphylococcal arthritis, unspecified shoulder |
| pyogenic arthritis, shoulder region | 71101 | M00111 | | Pneumococcal arthritis, right shoulder |
| pyogenic arthritis, shoulder region | 71101 | M00112 | | Pneumococcal arthritis, left shoulder |
| pyogenic arthritis, shoulder region | 71101 | M00119 | | Pneumococcal arthritis, unspecified shoulder |
| pyogenic arthritis, shoulder region | 71101 | M00211 | | Other streptococcal arthritis, right shoulder |
| pyogenic arthritis, shoulder region | 71101 | M00212 | | Other streptococcal arthritis, left shoulder |
| pyogenic arthritis, shoulder region | 71101 | M00811 | | Arthritis due to other bacteria, right shoulder |
| pyogenic arthritis, shoulder region | 71101 | M00812 | | Arthritis due to other bacteria, left shoulder |
| pyogenic arthritis, shoulder region | 71101 | M00819 | | Arthritis due to other bacteria, unspecified shoulder |
| pyogenic arthritis, shoulder region | 71101 | M009 | | Pyogenic arthritis, unspecified |
| pyogenic arthritis, upper arm | 71102 | M00021 | | Staphylococcal arthritis, right elbow |
| pyogenic arthritis, upper arm | 71102 | M00022 | | Staphylococcal arthritis, left elbow |
| pyogenic arthritis, upper arm | 71102 | M00029 | | Staphylococcal arthritis, unspecified elbow |
| pyogenic arthritis, upper arm | 71102 | M00121 | | Pneumococcal arthritis, right elbow |
| pyogenic arthritis, upper arm | 71102 | M00122 | | Pneumococcal arthritis, left elbow |
| pyogenic arthritis, upper arm | 71102 | M00129 | | Pneumococcal arthritis, unspecified elbow |
| pyogenic arthritis, upper arm | 71102 | M00221 | | Other streptococcal arthritis, right elbow |
| pyogenic arthritis, upper arm | 71102 | M00222 | | Other streptococcal arthritis, left elbow |
| pyogenic arthritis, upper arm | 71102 | M00229 | | Other streptococcal arthritis, unspecified elbow |
| pyogenic arthritis, upper arm | 71102 | M00821 | | Arthritis due to other bacteria, right elbow |
| pyogenic arthritis, upper arm | 71102 | M00822 | | Arthritis due to other bacteria, left elbow |
| pyogenic arthritis, upper arm | 71102 | M00829 | | Arthritis due to other bacteria, unspecified elbow |
| **Appendix B Table 1-BJI: IDC-9 and ICD-10 Codes for Bone and Joint Infections cont.** | | | | |
| **CD-9 Description** | **ICD-9_bji** | **ICD-10_bji** | | **ICD-10 Description** |
| pyogenic arthritis, forearm | 71103 | M00031 | | Staphylococcal arthritis, right wrist |
| pyogenic arthritis, forearm | 71103 | M00032 | | Staphylococcal arthritis, left wrist |
| pyogenic arthritis, forearm | 71103 | M00039 | | Staphylococcal arthritis, unspecified wrist |
| pyogenic arthritis, forearm | 71103 | M00131 | | Pneumococcal arthritis, right wrist |
| pyogenic arthritis, forearm | 71103 | M00132 | | Pneumococcal arthritis, left wrist |
| pyogenic arthritis, forearm | 71103 | M00139 | | Pneumococcal arthritis, unspecified wrist |
| pyogenic arthritis, forearm | 71103 | M00231 | | Other streptococcal arthritis, right wrist |
| pyogenic arthritis, forearm | 71103 | M00232 | | Other streptococcal arthritis, left wrist |
| pyogenic arthritis, forearm | 71103 | M00239 | | Other streptococcal arthritis, unspecified wrist |
| pyogenic arthritis, forearm | 71103 | M00831 | | Arthritis due to other bacteria, right wrist |
| pyogenic arthritis, forearm | 71103 | M00832 | | Arthritis due to other bacteria, left wrist |
| pyogenic arthritis, forearm | 71103 | M00839 | | Arthritis due to other bacteria, unspecified wrist |
| pyogenic arthritis, hand | 71104 | M00041 | | Staphylococcal arthritis, right hand |
| pyogenic arthritis, hand | 71104 | M00042 | | Staphylococcal arthritis, left hand |
| pyogenic arthritis, hand | 71104 | M00049 | | Staphylococcal arthritis, unspecified hand |
| pyogenic arthritis, hand | 71104 | M00141 | | Pneumococcal arthritis, right hand |
| pyogenic arthritis, hand | 71104 | M00142 | | Pneumococcal arthritis, left hand |
| pyogenic arthritis, hand | 71104 | M00149 | | Pneumococcal arthritis, unspecified hand |
| pyogenic arthritis, hand | 71104 | M00241 | | Other streptococcal arthritis, right hand |
| pyogenic arthritis, hand | 71104 | M00242 | | Other streptococcal arthritis, left hand |
| pyogenic arthritis, hand | 71104 | M00249 | | Other streptococcal arthritis, unspecified hand |
| pyogenic arthritis, hand | 71104 | M00841 | | Arthritis due to other bacteria, right hand |
| pyogenic arthritis, hand | 71104 | M00842 | | Arthritis due to other bacteria, left hand |
| pyogenic arthritis, hand | 71104 | M00849 | | Arthritis due to other bacteria, unspecified hand |
| pyogenic arthritis, pelvic region and thigh | 71105 | M00051 | | Staphylococcal arthritis, right hip |
| pyogenic arthritis, pelvic region and thigh | 71105 | M00052 | | Staphylococcal arthritis, left hip |
| pyogenic arthritis, pelvic region and thigh | 71105 | M00059 | | Staphylococcal arthritis, unspecified hip |
| pyogenic arthritis, pelvic region and thigh | 71105 | M00151 | | Pneumococcal arthritis, right hip |
| pyogenic arthritis, pelvic region and thigh | 71105 | M00152 | | Pneumococcal arthritis, left hip |
| pyogenic arthritis, pelvic region and thigh | 71105 | M00159 | | Pneumococcal arthritis, unspecified hip |
| pyogenic arthritis, pelvic region and thigh | 71105 | M00251 | | Other streptococcal arthritis, right hip |
| pyogenic arthritis, pelvic region and thigh | 71105 | M00252 | | Other streptococcal arthritis, left hip |
| pyogenic arthritis, pelvic region and thigh | 71105 | M00259 | | Other streptococcal arthritis, unspecified hip |
| pyogenic arthritis, pelvic region and thigh | 71105 | M00851 | | Arthritis due to other bacteria, right hip |
| pyogenic arthritis, pelvic region and thigh | 71105 | M00852 | | Arthritis due to other bacteria, left hip |
| pyogenic arthritis, pelvic region and thigh | 71105 | M00859 | | Arthritis due to other bacteria, unspecified hip |
| pyogenic arthritis, lower leg | 71106 | M00061 | | Staphylococcal arthritis, right knee |
| pyogenic arthritis, lower leg | 71106 | M00062 | | Staphylococcal arthritis, left knee |
| **Appendix B Table 1-BJI: IDC-9 and ICD-10 Codes for Bone and Joint Infections cont.** | | | | |
| **ICD-9 Description** | **ICD-9_bji** | **ICD-10_bji** | | **ICD-10 Description** |
| pyogenic arthritis, lower leg | 71106 | M00069 | | Staphylococcal arthritis, unspecified knee |
| pyogenic arthritis, lower leg | 71106 | M00161 | | Pneumococcal arthritis, right knee |
| pyogenic arthritis, lower leg | 71106 | M00169 | | Pneumococcal arthritis, unspecified knee |
| pyogenic arthritis, lower leg | 71106 | M00261 | | Other streptococcal arthritis, right knee |
| pyogenic arthritis, lower leg | 71106 | M00262 | | Other streptococcal arthritis, left knee |
| pyogenic arthritis, lower leg | 71106 | M00269 | | Other streptococcal arthritis, unspecified knee |
| pyogenic arthritis, lower leg | 71106 | M00861 | | Arthritis due to other bacteria, right knee |
| pyogenic arthritis, lower leg | 71106 | M00862 | | Arthritis due to other bacteria, left knee |
| pyogenic arthritis, lower leg | 71106 | M00869 | | Arthritis due to other bacteria, unspecified knee |
| pyogenic arthritis, ankle and foot | 71107 | M00071 | | Staphylococcal arthritis, right ankle and foot |
| pyogenic arthritis, ankle and foot | 71107 | M00072 | | Staphylococcal arthritis, left ankle and foot |
| pyogenic arthritis, ankle and foot | 71107 | M00079 | | Staphylococcal arthritis, unspecified ankle and foot |
| pyogenic arthritis, ankle and foot | 71107 | M00171 | | Pneumococcal arthritis, right ankle and foot |
| pyogenic arthritis, ankle and foot | 71107 | M00172 | | Pneumococcal arthritis, left ankle and foot |
| pyogenic arthritis, ankle and foot | 71107 | M00179 | | Pneumococcal arthritis, unspecified ankle and foot |
| pyogenic arthritis, ankle and foot | 71107 | M00271 | | Other streptococcal arthritis, right ankle and foot |
| pyogenic arthritis, ankle and foot | 71107 | M00272 | | Other streptococcal arthritis, left ankle and foot |
| pyogenic arthritis, ankle and foot | 71107 | M00279 | | Other streptococcal arthritis, unspecified ankle and foot |
| pyogenic arthritis, ankle and foot | 71107 | M00871 | | Arthritis due to other bacteria, right ankle and foot |
| pyogenic arthritis, ankle and foot | 71107 | M00872 | | Arthritis due to other bacteria, left ankle and foot |
| pyogenic arthritis, ankle and foot | 71107 | M00879 | | Arthritis due to other bacteria, unspecified ankle and foot |
| pyogenic arthritis, other specified sites | 71108 | M0008 | | Staphylococcal arthritis, vertebrae |
| pyogenic arthritis, other specified sites | 71108 | M0009 | | Staphylococcal polyarthritis |
| pyogenic arthritis, other specified sites | 71108 | M0018 | | Pneumococcal arthritis, vertebrae |
| pyogenic arthritis, other specified sites | 71108 | M0028 | | Other streptococcal arthritis, vertebrae |
| pyogenic arthritis, other specified sites | 71108 | M0088 | | Arthritis due to other bacteria, vertebrae |
| pyogenic arthritis, multiple sites | 71109 | M0009 | | Staphylococcal polyarthritis |
| pyogenic arthritis, multiple sites | 71109 | M0019 | | Pneumococcal polyarthritis |
| pyogenic arthritis, multiple sites | 71109 | M0029 | | Other streptococcal polyarthritis |
| pyogenic arthritis, multiple sites | 71109 | M0089 | | Polyarthritis due to other bacteria |
| Arthropathy associated with other bacterial diseases, site unspecified | 71140 | M01X0 | | Direct infection of unspecified joint in infectious and parasitic diseases classified elsewhere |
| Arthropathy associated with other bacterial diseases, site unspecified | 71140 | M0280 | | Other reactive arthropathies, unspecified site |
| Arthropathy associated with other bacterial diseases, upper arm | 71142 | M01X21 | | Direct infection of right elbow in infectious and parasitic diseases classified elsewhere |
| Arthropathy associated with other bacterial diseases, upper arm | 71142 | M01X22 | | Direct infection of left elbow in infectious and parasitic diseases classified elsewhere |
|  |  |  | |  |
| **Appendix B Table 1-BJI: IDC-9 and ICD-10 Codes for Bone and Joint Infections cont.** | | | | |
| **ICD-9 Description** | **ICD-9_bji** | **ICD-10_bji** | | **ICD-10 Description** |
| Arthropathy associated with other bacterial diseases, upper arm | 71142 | M01X29 | | Direct infection of unspecified elbow in infectious and parasitic diseases classified elsewhere |
| Arthropathy associated with other bacterial diseases, upper arm | 71142 | M02829 | | Other reactive arthropathies, unspecified elbow |
| [Arthropathy associated with other bacterial diseases, forearm](http://www.icd10data.com/Convert/370.31) | 71143 | M01X31 | | Direct infection of right wrist in infectious and parasitic diseases classified elsewhere |
| [Arthropathy associated with other bacterial diseases, forearm](http://www.icd10data.com/Convert/370.31) | 71143 | M01X32 | | Direct infection of left wrist in infectious and parasitic diseases classified elsewhere |
| [Arthropathy associated with other bacterial diseases, forearm](http://www.icd10data.com/Convert/370.31) | 71143 | M01X39 | | Direct infection of unspecified wrist in infectious and parasitic diseases classified elsewhere |
| [Arthropathy associated with other bacterial diseases, forearm](http://www.icd10data.com/Convert/370.31) | 71143 | M02831 | | Other reactive arthropathies, right wrist |
| [Arthropathy associated with other bacterial diseases, forearm](http://www.icd10data.com/Convert/370.31) | 71143 | M02832 | | Other reactive arthropathies, left wrist |
| [Arthropathy associated with other bacterial diseases, forearm](http://www.icd10data.com/Convert/370.31) | 71143 | M02839 | | Other reactive arthropathies, unspecified wrist |
| Arthropathy associated with other bacterial diseases, hand | 71144 | M01X41 | | Direct infection of right hand in infectious and parasitic diseases classified elsewhere |
| Arthropathy associated with other bacterial diseases, hand | 71144 | M01X42 | | Direct infection of left hand in infectious and parasitic diseases classified elsewhere |
| Arthropathy associated with other bacterial diseases, hand | 71144 | M01X49 | | Direct infection of unspecified hand in infectious and parasitic diseases classified elsewhere |
| Arthropathy associated with other bacterial diseases, hand | 71144 | M02841 | | Other reactive arthropathies, right hand |
| Arthropathy associated with other bacterial diseases, hand | 71144 | M02842 | | Other reactive arthropathies, left hand |
| Arthropathy associated with other bacterial diseases, hand | 71144 | M02849 | | Other reactive arthropathies, unspecified hand |
| Arthropathy associated with other bacterial diseases, pelvic region and thigh | 71145 | A1802 | | Tuberculous arthritis of other joints |
| Arthropathy associated with other bacterial diseases, pelvic region and thigh | 71145 | M01X51 | | Direct infection of right hip in infectious and parasitic diseases classified elsewhere |
| Arthropathy associated with other bacterial diseases, pelvic region and thigh | 71145 | M01X52 | | Direct infection of left hip in infectious and parasitic diseases classified elsewhere |
| Arthropathy associated with other bacterial diseases, pelvic region and thigh | 71145 | M01X52 | | Direct infection of left hip in infectious and parasitic diseases classified elsewhere |
| Arthropathy associated with other bacterial diseases, pelvic region and thigh | 71145 | M01X59 | | Direct infection of unspecified hip in infectious and parasitic diseases classified elsewhere |
| Arthropathy associated with other bacterial diseases, pelvic region and thigh | 71145 | M02851 | | Other reactive arthropathies, right hip |
| Arthropathy associated with other bacterial diseases, pelvic region and thigh | 71145 | M02852 | | Other reactive arthropathies, left hip |
| Arthropathy associated with other bacterial diseases, pelvic region and thigh | 71145 | M02859 | | Other reactive arthropathies, unspecified hip |
| **Appendix B Table 1-BJI: IDC-9 and ICD-10 Codes for Bone and Joint Infections cont.** | | | | |
| **ICD-9 Description** | **ICD-9_bji** | **ICD-10_bji** | | **ICD-10 Description** |
| Arthropathy associated with other bacterial diseases, ankle and foot | 71147 | M01X71 | | Direct infection of right ankle and foot in infectious and parasitic diseases classified elsewhere |
| Arthropathy associated with other bacterial diseases, ankle and foot | 71147 | M01X72 | | Direct infection of left ankle and foot in infectious and parasitic diseases classified elsewhere |
| Arthropathy associated with other bacterial diseases, ankle and foot | 71147 | M01X79 | | Direct infection of unspecified ankle and foot in infectious and parasitic diseases classified elsewhere |
| Arthropathy associated with other bacterial diseases, ankle and foot | 71147 | M02871 | | Other reactive arthropathies, right ankle and foot |
| Arthropathy associated with other bacterial diseases, ankle and foot | 71147 | M02872 | | Other reactive arthropathies, left ankle and foot |
| Arthropathy associated with other bacterial diseases, ankle and foot | 71147 | M02879 | | Other reactive arthropathies, unspecified ankle and foot |
| Arthropathy associated with other bacterial diseases, other specified sites | 71148 | A1801 | | Tuberculosis of spine |
| Arthropathy associated with other bacterial diseases, other specified sites | 71148 | A1802 | | Tuberculous arthritis of other joints |
| Arthropathy associated with other bacterial diseases, other specified sites | 71148 | M01X8 | | Direct infection of vertebrae in infectious and parasitic diseases classified elsewhere |
| Arthropathy associated with other bacterial diseases, other specified sites | 71148 | M0288 | | Other reactive arthropathies, vertebrae |
| Arthropathy associated with other bacterial diseases, multiple sites | 71149 | M01X9 | | Direct infection of multiple joints in infectious and parasitic diseases classified elsewhere |
| Arthropathy associated with other bacterial diseases, multiple sites | 71149 | M0289 | | Other reactive arthropathies, multiple sites |
| Arthropathy associated with other Viral diseases, site unspecified | 71150 | M01X0 | | Direct infection of unspecified joint in infectious and parasitic diseases classified elsewhere |
| Arthropathy associated with other Viral diseases, shoulder region | 71151 | M01X11 | | Direct infection of right shoulder in infectious and parasitic diseases classified elsewhere |
| Arthropathy associated with other Viral diseases, shoulder region | 71151 | M01X12 | | Direct infection of left shoulder in infectious and parasitic diseases classified elsewhere |
| Arthropathy associated with other Viral diseases, shoulder region | 71151 | M01X19 | | Direct infection of unspecified shoulder in infectious and parasitic diseases classified elsewhere |
| Arthropathy associated with other Viral diseases, shoulder region | 71151 | M02811 | | Other reactive arthropathies, right shoulder |
| Arthropathy associated with other Viral diseases, shoulder region | 71151 | M02812 | | Other reactive arthropathies, left shoulder |
| Arthropathy associated with other Viral diseases, shoulder region | 71151 | M02819 | | Other reactive arthropathies, unspecified shoulder |
| Arthropathy associated with other Viral diseases, upper arm | 71152 | M01X21 | | Direct infection of right elbow in infectious and parasitic diseases classified elsewhere |
| Arthropathy associated with other Viral diseases, upper arm | 71152 | M01X22 | | Direct infection of left elbow in infectious and parasitic diseases classified elsewhere |
| Arthropathy associated with other Viral diseases, upper arm | 71152 | M01X29 | | Direct infection of unspecified elbow in infectious and parasitic diseases classified elsewhere |
| Arthropathy associated with other Viral diseases, upper arm | 71152 | M02829 | | Other reactive arthropathies, unspecified elbow |
| Arthropathy associated with other Viral diseases, forearm | 71153 | M01X31 | | Direct infection of right wrist in infectious and parasitic diseases classified elsewhere |
| **Appendix B Table 1-BJI: IDC-9 and ICD-10 Codes for Bone and Joint Infections cont.** | | | | |
| **ICD-9 Description** | **ICD-9_bji** | **ICD-10_bji** | | **ICD-10 Description** |
| Arthropathy associated with other Viral diseases, forearm | 71153 | M01X32 | | Direct infection of left wrist in infectious and parasitic diseases classified elsewhere |
| Arthropathy associated with other Viral diseases, forearm | 71153 | M01X39 | | Direct infection of unspecified wrist in infectious and parasitic diseases classified elsewhere |
| Arthropathy associated with other Viral diseases, forearm | 71153 | M02831 | | Other reactive arthropathies, right wrist |
| Arthropathy associated with other Viral diseases, forearm | 71153 | M02832 | | Other reactive arthropathies, left wrist |
| Arthropathy associated with other Viral diseases, forearm | 71153 | M02839 | | Other reactive arthropathies, unspecified wrist |
| Arthropathy associated with other Viral diseases, hand | 71154 | M01X41 | | Direct infection of right hand in infectious and parasitic diseases classified elsewhere |
| Arthropathy associated with other Viral diseases, hand | 71154 | M01X42 | | Direct infection of left hand in infectious and parasitic diseases classified elsewhere |
| Arthropathy associated with other Viral diseases, hand | 71154 | M01X49 | | Direct infection of unspecified hand in infectious and parasitic diseases classified elsewhere |
| Arthropathy associated with other Viral diseases, hand | 71154 | M02841 | | Other reactive arthropathies, right hand |
| Arthropathy associated with other Viral diseases, hand | 71154 | M02842 | | Other reactive arthropathies, left hand |
| Arthropathy associated with other Viral diseases, hand | 71154 | M02849 | | Other reactive arthropathies, unspecified hand |
| Arthropathy associated with other Viral diseases, pelvic region and thigh | 71155 | M01X51 | | Direct infection of right hip in infectious and parasitic diseases classified elsewhere |
| Arthropathy associated with other Viral diseases, pelvic region and thigh | 71155 | M01X52 | | Direct infection of left hip in infectious and parasitic diseases classified elsewhere |
| Arthropathy associated with other Viral diseases, pelvic region and thigh | 71155 | M01X59 | | Direct infection of unspecified hip in infectious and parasitic diseases classified elsewhere |
| Arthropathy associated with other Viral diseases, pelvic region and thigh | 71155 | M02851 | | Other reactive arthropathies, right hip |
| Arthropathy associated with other Viral diseases, pelvic region and thigh | 71155 | M02852 | | Other reactive arthropathies, left hip |
| Arthropathy associated with other Viral diseases, pelvic region and thigh | 71155 | M02859 | | Other reactive arthropathies, unspecified hip |
| Arthropathy associated with other Viral diseases, lower leg | 71156 | M01X61 | | Direct infection of right knee in infectious and parasitic diseases classified elsewhere |
| Arthropathy associated with other Viral diseases, lower leg | 71156 | M01X62 | | Direct infection of left knee in infectious and parasitic diseases classified elsewhere |
| Arthropathy associated with other Viral diseases, lower leg | 71156 | M01X69 | | Direct infection of unspecified knee in infectious and parasitic diseases classified elsewhere |
| Arthropathy associated with other Viral diseases, lower leg | 71156 | M02861 | | Other reactive arthropathies, right knee |
| Arthropathy associated with other Viral diseases, lower leg | 71156 | M02862 | | Other reactive arthropathies, left knee |
| Arthropathy associated with other Viral diseases, lower leg | 71156 | M02869 | | Other reactive arthropathies, unspecified knee |
| Arthropathy associated with other Viral diseases, ankle and foot | 71157 | M01X71 | | Direct infection of right ankle and foot in infectious and parasitic diseases classified elsewhere |
| Arthropathy associated with other Viral diseases, ankle and foot | 71157 | M01X72 | | Direct infection of left ankle and foot in infectious and parasitic diseases classified elsewhere |
| **Appendix B Table 1-BJI: IDC-9 and ICD-10 Codes for Bone and Joint Infections cont.** | | | | |
| **ICD-9 Description** | **ICD-9_bji** | **ICD-10_bji** | | **ICD-10 Description** |
| Arthropathy associated with other Viral diseases, ankle and foot | 71157 | M01X79 | | Direct infection of unspecified ankle and foot in infectious and parasitic diseases classified elsewhere |
| Arthropathy associated with other Viral diseases, ankle and foot | 71157 | M02871 | | Other reactive arthropathies, right ankle and foot |
| Arthropathy associated with other Viral diseases, ankle and foot | 71157 | M02872 | | Other reactive arthropathies, left ankle and foot |
| Arthropathy associated with other Viral diseases, ankle and foot | 71157 | M02879 | | Other reactive arthropathies, unspecified ankle and foot |
| Arthropathy associated with other Viral diseases, other specified sites | 71158 | M01X8 | | Direct infection of vertebrae in infectious and parasitic diseases classified elsewhere |
| Arthropathy associated with other Viral diseases, other specified sites | 71158 | M0288 | | Other reactive arthropathies, vertebrae |
| Arthropathy associated with other Viral diseases, multiple sites | 71159 | M01X9 | | Direct infection of multiple joints in infectious and parasitic diseases classified elsewhere |
| Arthropathy associated with other Viral diseases, multiple sites | 71159 | M0289 | | Other reactive arthropathies, multiple sites |
| Arthropathy associated with other infectious and parasitic diseases, site unspecified | 71180 | M01X0 | | Direct infection of unspecified joint in infectious and parasitic diseases classified elsewhere |
| Arthropathy associated with other infectious and parasitic diseases, site unspecified | 71180 | M0280 | | Other reactive arthropathies, unspecified site |
| Arthropathy associated with other infectious and parasitic diseases, shoulder region | 71181 | M01X11 | | Direct infection of right shoulder in infectious and parasitic diseases classified elsewhere |
| Arthropathy associated with other infectious and parasitic diseases, shoulder region | 71181 | M01X12 | | Direct infection of left shoulder in infectious and parasitic diseases classified elsewhere |
| Arthropathy associated with other infectious and parasitic diseases, shoulder region | 71181 | M01X19 | | Direct infection of unspecified shoulder in infectious and parasitic diseases classified elsewhere |
| Arthropathy associated with other infectious and parasitic diseases, shoulder region | 71181 | M02811 | | Other reactive arthropathies, right shoulder |
| Arthropathy associated with other infectious and parasitic diseases, shoulder region | 71181 | M02812 | | Other reactive arthropathies, left shoulder |
| Arthropathy associated with other infectious and parasitic diseases, shoulder region | 71181 | M02819 | | Other reactive arthropathies, unspecified shoulder |
| Arthropathy associated with other infectious and parasitic diseases, upper arm | 71182 | M01X21 | | Direct infection of right elbow in infectious and parasitic diseases classified elsewhere |
| Arthropathy associated with other infectious and parasitic diseases, upper arm | 71182 | M01X22 | | Direct infection of left elbow in infectious and parasitic diseases classified elsewhere |
| Arthropathy associated with other infectious and parasitic diseases, upper arm | 71182 | M01X29 | | Direct infection of unspecified elbow in infectious and parasitic diseases classified elsewhere |
| Arthropathy associated with other infectious and parasitic diseases, upper arm | 71182 | M02821 | | Other reactive arthropathies, right elbow |
| Arthropathy associated with other infectious and parasitic diseases, upper arm | 71182 | M02822 | | Other reactive arthropathies, left elbow |
| Arthropathy associated with other infectious and parasitic diseases, upper arm | 71182 | M02829 | | Other reactive arthropathies, unspecified elbow |
| **Appendix B Table 1-BJI: IDC-9 and ICD-10 Codes for Bone and Joint Infections cont.** | | | | |
| **ICD-9 Description** | **ICD-9_bji** | **ICD-10_bji** | | **ICD-10 Description** |
| Arthropathy associated with other infectious and parasitic diseases, forearm | 71183 | M01X31 | | Direct infection of right wrist in infectious and parasitic diseases classified elsewhere |
| Arthropathy associated with other infectious and parasitic diseases, forearm | 71183 | M01X32 | | Direct infection of left wrist in infectious and parasitic diseases classified elsewhere |
| Arthropathy associated with other infectious and parasitic diseases, forearm | 71183 | M01X39 | | Direct infection of unspecified wrist in infectious and parasitic diseases classified elsewhere |
| Arthropathy associated with other infectious and parasitic diseases, forearm | 71183 | M02831 | | Other reactive arthropathies, right wrist |
| Arthropathy associated with other infectious and parasitic diseases, forearm | 71183 | M02832 | | Other reactive arthropathies, left wrist |
| Arthropathy associated with other infectious and parasitic diseases, forearm | 71183 | M02839 | | Other reactive arthropathies, unspecified wrist |
| Arthropathy associated with other infectious and parasitic diseases, hand | 71184 | M01X41 | | Direct infection of right hand in infectious and parasitic diseases classified elsewhere |
| Arthropathy associated with other infectious and parasitic diseases, hand | 71184 | M01X42 | | Direct infection of left hand in infectious and parasitic diseases classified elsewhere |
| Arthropathy associated with other infectious and parasitic diseases, hand | 71184 | M01X49 | | Direct infection of unspecified hand in infectious and parasitic diseases classified elsewhere |
| Arthropathy associated with other infectious and parasitic diseases, hand | 71184 | M02841 | | Other reactive arthropathies, right hand |
| Arthropathy associated with other infectious and parasitic diseases, hand | 71184 | M02842 | | Other reactive arthropathies, left hand |
| Arthropathy associated with other infectious and parasitic diseases, hand | 71184 | M02849 | | Other reactive arthropathies, unspecified hand |
| Arthropathy associated with other infectious and parasitic diseases, pelvic region and thigh | 71185 | M01X51 | | Direct infection of right hip in infectious and parasitic diseases classified elsewhere |
| Arthropathy associated with other infectious and parasitic diseases, pelvic region and thigh | 71185 | M01X52 | | Direct infection of left hip in infectious and parasitic diseases classified elsewhere |
| Arthropathy associated with other infectious and parasitic diseases, pelvic region and thigh | 71185 | M01X59 | | Direct infection of unspecified hip in infectious and parasitic diseases classified elsewhere |
| Arthropathy associated with other infectious and parasitic diseases, pelvic region and thigh | 71185 | M02851 | | Other reactive arthropathies, right hip |
| Arthropathy associated with other infectious and parasitic diseases, pelvic region and thigh | 71185 | M02852 | | Other reactive arthropathies, left hip |
| Arthropathy associated with other infectious and parasitic diseases, pelvic region and thigh | 71185 | M02859 | | Other reactive arthropathies, unspecified hip |
| Arthropathy associated with other infectious and parasitic diseases, lower leg | 71186 | M01X61 | | Direct infection of right knee in infectious and parasitic diseases classified elsewhere |
| Arthropathy associated with other infectious and parasitic diseases, lower leg | 71186 | M01X62 | | Direct infection of left knee in infectious and parasitic diseases classified elsewhere |
| Arthropathy associated with other infectious and parasitic diseases, lower leg | 71186 | M01X69 | | Direct infection of unspecified knee in infectious and parasitic diseases classified elsewhere |
| Arthropathy associated with other infectious and parasitic diseases, lower leg | 71186 | M02861 | | Other reactive arthropathies, right knee |
|  |  |  | |  |
| **Appendix B Table 1-BJI: IDC-9 and ICD-10 Codes for Bone and Joint Infections cont.** | | | | |
| **ICD-9 Description** | **ICD-9_bji** | **ICD-10_bji** | | **ICD-10 Description** |
| Arthropathy associated with other infectious and parasitic diseases, lower leg | 71186 | M02862 | | Other reactive arthropathies, left knee |
| Arthropathy associated with other infectious and parasitic diseases, lower leg | 71186 | M02869 | | Other reactive arthropathies, unspecified knee |
| Arthropathy associated with other infectious and parasitic diseases, ankle and foot | 71187 | M01X71 | | Direct infection of right ankle and foot in infectious and parasitic diseases classified elsewhere |
| Arthropathy associated with other infectious and parasitic diseases, ankle and foot | 71187 | M01X72 | | Direct infection of left ankle and foot in infectious and parasitic diseases classified elsewhere |
| Arthropathy associated with other infectious and parasitic diseases, ankle and foot | 71187 | M01X79 | | Direct infection of unspecified ankle and foot in infectious and parasitic diseases classified elsewhere |
| Arthropathy associated with other infectious and parasitic diseases, ankle and foot | 71187 | M02871 | | Other reactive arthropathies, right ankle and foot |
| Arthropathy associated with other infectious and parasitic diseases, ankle and foot | 71187 | M02872 | | Other reactive arthropathies, left ankle and foot |
| Arthropathy associated with other infectious and parasitic diseases, ankle and foot | 71187 | M02879 | | Other reactive arthropathies, unspecified ankle and foot |
| Arthropathy associated with other infectious and parasitic diseases, other specified sites | 71188 | M01X8 | | Direct infection of vertebrae in infectious and parasitic diseases classified elsewhere |
| Arthropathy associated with other infectious and parasitic diseases, other specified sites | 71188 | M0288 | | Other reactive arthropathies, vertebrae |
| Arthropathy associated with other infectious and parasitic diseases, multiple sites | 71189 | M01X9 | | Direct infection of multiple joints in infectious and parasitic diseases classified elsewhere |
| Arthropathy associated with other infectious and parasitic diseases, multiple sites | 71189 | M0289 | | Other reactive arthropathies, multiple sites |
| Unspecified infective arthritis, site unspecified | 71190 | M01X0 | | Direct infection of unspecified joint in infectious and parasitic diseases classified elsewhere |
| Unspecified infective arthritis, shoulder region | 71191 | M01X19 | | Direct infection of unspecified shoulder in infectious and parasitic diseases classified elsewhere |
| Unspecified infective arthritis, upper arm | 71192 | M01X29 | | Direct infection of unspecified elbow in infectious and parasitic diseases classified elsewhere |
| Unspecified infective arthritis, forearm | 71193 | M01X39 | | Direct infection of unspecified wrist in infectious and parasitic diseases classified elsewhere |
| Unspecified infective arthritis, hand | 71194 | M01X49 | | Direct infection of unspecified hand in infectious and parasitic diseases classified elsewhere |
| Unspecified infective arthritis, pelvic region and thigh | 71195 | M01X59 | | Direct infection of unspecified hip in infectious and parasitic diseases classified elsewhere |
| Unspecified infective arthritis, lower leg | 71196 | M01X69 | | Direct infection of unspecified knee in infectious and parasitic diseases classified elsewhere |
| Unspecified infective arthritis, ankle and foot | 71197 | M01X79 | | Direct infection of unspecified ankle and foot in infectious and parasitic diseases classified elsewhere |
| **Appendix B Table 1-BJI: IDC-9 and ICD-10 Codes for Bone and Joint Infections cont.** | | | | |
| **ICD-9 Description** | **ICD-9_bji** | **ICD-10_bji** | | **ICD-10 Description** |
| Unspecified infective arthritis, other specified sites | 71198 | M01X8 | | Direct infection of vertebrae in infectious and parasitic diseases classified elsewhere |
| Unspecified infective arthritis, multiple sites | 71199 | M01X9 | | Direct infection of multiple joints in infectious and parasitic diseases classified elsewhere |
| Fibular collateral ligament bursitis | 72663 | M76899 | | Other specified enthesopathies of unspecified lower limb, excluding foot |
| Olecranon bursitis | 72633 | M7020 | | Olecranon bursitis, unspecified elbow |
| Olecranon bursitis | 72633 | M7021 | | Olecranon bursitis, right elbow |
| Olecranon bursitis | 72633 | M7022 | | Olecranon bursitis, left elbow |
| Pes anserinus tendinitis or bursitis | 72661 | M76899 | | Other specified enthesopathies of unspecified lower limb, excluding foot |
| Tibila collateral ligament bursitis | 72662 | M7640 | | Tibial collateral bursitis [Pellegrini-Stieda], unspecified leg |
| Tibila collateral ligament bursitis | 72662 | M7641 | | Tibial collateral bursitis [Pellegrini-Stieda], right leg |
| Tibila collateral ligament bursitis | 72662 | M7642 | | Tibial collateral bursitis [Pellegrini-Stieda], left leg |
| Prepatella bursitis | 72665 | M7040 | | Prepatellar bursitis, unspecified knee |
| Prepatella bursitis | 72665 | M7041 | | Prepatellar bursitis, right knee |
| Prepatella bursitis | 72665 | M7042 | | Prepatellar bursitis, left knee |
| Other disorders of synovium, tendon, and bursa | 72789 | M6500 | | Abscess of tendon sheath, unspecified site |
| Other disorders of synovium, tendon, and bursa | 72789 | M65011 | | Abscess of tendon sheath, right shoulder |
| Other disorders of synovium, tendon, and bursa | 72789 | M65012 | | Abscess of tendon sheath, left shoulder |
| Other disorders of synovium, tendon, and bursa | 72789 | M65019 | | Abscess of tendon sheath, unspecified shoulder |
| Other disorders of synovium, tendon, and bursa | 72789 | M65021 | | Abscess of tendon sheath, right upper arm |
| Other disorders of synovium, tendon, and bursa | 72789 | M65022 | | Abscess of tendon sheath, left upper arm |
| Other disorders of synovium, tendon, and bursa | 72789 | M65029 | | Abscess of tendon sheath, unspecified upper arm |
| Other disorders of synovium, tendon, and bursa | 72789 | M65031 | | Abscess of tendon sheath, right forearm |
| Other disorders of synovium, tendon, and bursa | 72789 | M65032 | | Abscess of tendon sheath, left forearm |
| Other disorders of synovium, tendon, and bursa | 72789 | M65039 | | Abscess of tendon sheath, unspecified forearm |
| Other disorders of synovium, tendon, and bursa | 72789 | M65041 | | Abscess of tendon sheath, right hand |
| Other disorders of synovium, tendon, and bursa | 72789 | M65042 | | Abscess of tendon sheath, left hand |
| Other disorders of synovium, tendon, and bursa | 72789 | M65049 | | Abscess of tendon sheath, unspecified hand |
| Other disorders of synovium, tendon, and bursa | 72789 | M65051 | | Abscess of tendon sheath, right thigh |
| Other disorders of synovium, tendon, and bursa | 72789 | M65052 | | Abscess of tendon sheath, left thigh |
| Other disorders of synovium, tendon, and bursa | 72789 | M65059 | | Abscess of tendon sheath, unspecified thigh |
| Other disorders of synovium, tendon, and bursa | 72789 | M65061 | | Abscess of tendon sheath, right lower leg |
| Other disorders of synovium, tendon, and bursa | 72789 | M65062 | | Abscess of tendon sheath, left lower leg |
| Other disorders of synovium, tendon, and bursa | 72789 | M65069 | | Abscess of tendon sheath, unspecified lower leg |
| Other disorders of synovium, tendon, and bursa | 72789 | M65071 | | Abscess of tendon sheath, right ankle and foot |
| Other disorders of synovium, tendon, and bursa | 72789 | M65072 | | Abscess of tendon sheath, left ankle and foot |
| **Appendix B Table 1-BJI: IDC-9 and ICD-10 Codes for Bone and Joint Infections cont.** | | | | |
| **ICD-9 Description** | **ICD-9_bji** | **ICD-10_bji** | | **ICD-10 Description** |
| Other disorders of synovium, tendon, and bursa | 72789 | M65079 | | Abscess of tendon sheath, unspecified ankle and foot |
| Other disorders of synovium, tendon, and bursa | 72789 | M6508 | | Abscess of tendon sheath, other site |
| Other disorders of synovium, tendon, and bursa | 72789 | M6720 | | Synovial hypertrophy, not elsewhere classified, unspecified site |
| Other disorders of synovium, tendon, and bursa | 72789 | M67211 | | Synovial hypertrophy, not elsewhere classified, right shoulder |
| Other disorders of synovium, tendon, and bursa | 72789 | M67212 | | Synovial hypertrophy, not elsewhere classified, left shoulder |
| Other disorders of synovium, tendon, and bursa | 72789 | M67219 | | Synovial hypertrophy, not elsewhere classified, unspecified shoulder |
| Other disorders of synovium, tendon, and bursa | 72789 | M67221 | | Synovial hypertrophy, not elsewhere classified, right upper arm |
| Other disorders of synovium, tendon, and bursa | 72789 | M67222 | | Synovial hypertrophy, not elsewhere classified, left upper arm |
| Other disorders of synovium, tendon, and bursa | 72789 | M67229 | | Synovial hypertrophy, not elsewhere classified, unspecified upper arm |
| Other disorders of synovium, tendon, and bursa | 72789 | M67231 | | Synovial hypertrophy, not elsewhere classified, right forearm |
| Other disorders of synovium, tendon, and bursa | 72789 | M67232 | | Synovial hypertrophy, not elsewhere classified, left forearm |
| Other disorders of synovium, tendon, and bursa | 72789 | M67239 | | Synovial hypertrophy, not elsewhere classified, unspecified forearm |
| Other disorders of synovium, tendon, and bursa | 72789 | M67241 | | Synovial hypertrophy, not elsewhere classified, right hand |
| Other disorders of synovium, tendon, and bursa | 72789 | M67242 | | Synovial hypertrophy, not elsewhere classified, left hand |
| Other disorders of synovium, tendon, and bursa | 72789 | M67249 | | Synovial hypertrophy, not elsewhere classified, unspecified hand |
| Other disorders of synovium, tendon, and bursa | 72789 | M67251 | | Synovial hypertrophy, not elsewhere classified, right thigh |
| Other disorders of synovium, tendon, and bursa | 72789 | M67252 | | Synovial hypertrophy, not elsewhere classified, left thigh |
| Other disorders of synovium, tendon, and bursa | 72789 | M67259 | | Synovial hypertrophy, not elsewhere classified, unspecified thigh |
| Other disorders of synovium, tendon, and bursa | 72789 | M67261 | | Synovial hypertrophy, not elsewhere classified, right lower leg |
| Other disorders of synovium, tendon, and bursa | 72789 | M67262 | | Synovial hypertrophy, not elsewhere classified, left lower leg |
| Other disorders of synovium, tendon, and bursa | 72789 | M67269 | | Synovial hypertrophy, not elsewhere classified, unspecified lower leg |
| Other disorders of synovium, tendon, and bursa | 72789 | M67271 | | Synovial hypertrophy, not elsewhere classified, right ankle and foot |
| Other disorders of synovium, tendon, and bursa | 72789 | M67279 | | Synovial hypertrophy, not elsewhere classified, unspecified ankle and foot |
| Other disorders of synovium, tendon, and bursa | 72789 | M6728 | | Synovial hypertrophy, not elsewhere classified, other site |
| Other disorders of synovium, tendon, and bursa | 72789 | M6729 | | Synovial hypertrophy, not elsewhere classified, multiple sites |
| Other disorders of synovium, tendon, and bursa | 72789 | M6780 | | Other specified disorders of synovium and tendon, unspecified site |
| Other disorders of synovium, tendon, and bursa | 72789 | M67811 | | Other specified disorders of synovium, right shoulder |
| Other disorders of synovium, tendon, and bursa | 72789 | M67812 | | Other specified disorders of synovium, left shoulder |
| Acute osteomyelitis, site unspecified | 73000 | M8600 | | Acute hematogenous osteomyelitis, unspecified site |
| Acute osteomyelitis, site unspecified | 73000 | M8610 | | Other acute osteomyelitis, unspecified site |
| Acute osteomyelitis, site unspecified | 73000 | M8620 | | Subacute osteomyelitis, unspecified site |
| Acute osteomyelitis, shoulder region | 73001 | M86011 | | Acute hematogenous osteomyelitis, right shoulder |
| Acute osteomyelitis, shoulder region | 73001 | M86012 | | Acute hematogenous osteomyelitis, left shoulder |
| Acute osteomyelitis, shoulder region | 73001 | M86019 | | Acute hematogenous osteomyelitis, unspecified shoulder |
| Acute osteomyelitis, shoulder region | 73001 | M86111 | | Other acute osteomyelitis, right shoulder |
| Acute osteomyelitis, shoulder region | 73001 | M86112 | | Other acute osteomyelitis, left shoulder |
| **Appendix B Table 1-BJI: IDC-9 and ICD-10 Codes for Bone and Joint Infections cont.** | | | | |
| **ICD-9 Description** | **ICD-9_bji** | | **ICD-10_bji** | **ICD-10 Description** |
| Acute osteomyelitis, shoulder region | 73001 | M86119 | | Other acute osteomyelitis, unspecified shoulder |
| Acute osteomyelitis, shoulder region | 73001 | M86211 | | Subacute osteomyelitis, right shoulder |
| Acute osteomyelitis, shoulder region | 73001 | M86212 | | Subacute osteomyelitis, left shoulder |
| Acute osteomyelitis, shoulder region | 73001 | M86219 | | Subacute osteomyelitis, unspecified shoulder |
| Acute osteomyelitis, upper arm | 73002 | M86021 | | Acute hematogenous osteomyelitis, right humerus |
| Acute osteomyelitis, upper arm | 73002 | M86022 | | Acute hematogenous osteomyelitis, left humerus |
| Acute osteomyelitis, upper arm | 73002 | M86029 | | Acute hematogenous osteomyelitis, unspecified humerus |
| Acute osteomyelitis, upper arm | 73002 | M86121 | | Other acute osteomyelitis, right humerus |
| Acute osteomyelitis, upper arm | 73002 | M86122 | | Other acute osteomyelitis, left humerus |
| Acute osteomyelitis, upper arm | 73002 | M86129 | | Other acute osteomyelitis, unspecified humerus |
| Acute osteomyelitis, upper arm | 73002 | M86221 | | Subacute osteomyelitis, right humerus |
| Acute osteomyelitis, upper arm | 73002 | M86222 | | Subacute osteomyelitis, left humerus |
| Acute osteomyelitis, upper arm | 73002 | M86229 | | Subacute osteomyelitis, unspecified humerus |
| Acute osteomyelitis, forarm | 73003 | M86031 | | Acute hematogenous osteomyelitis, right radius and ulna |
| Acute osteomyelitis, forarm | 73003 | M86032 | | Acute hematogenous osteomyelitis, left radius and ulna |
| Acute osteomyelitis, forarm | 73003 | M86039 | | Acute hematogenous osteomyelitis, unspecified radius and ulna |
| Acute osteomyelitis, forarm | 73003 | M86131 | | Other acute osteomyelitis, right radius and ulna |
| Acute osteomyelitis, forarm | 73003 | M86132 | | Other acute osteomyelitis, left radius and ulna |
| Acute osteomyelitis, forarm | 73003 | M86139 | | Other acute osteomyelitis, unspecified radius and ulna |
| Acute osteomyelitis, forarm | 73003 | M86231 | | Subacute osteomyelitis, right radius and ulna |
| Acute osteomyelitis, forarm | 73003 | M86232 | | Subacute osteomyelitis, left radius and ulna |
| Acute osteomyelitis, forarm | 73003 | M86239 | | Subacute osteomyelitis, unspecified radius and ulna |
| Acute osteomyelitis, hand | 73004 | M86041 | | Acute hematogenous osteomyelitis, right hand |
| Acute osteomyelitis, hand | 73004 | M86042 | | Acute hematogenous osteomyelitis, left hand |
| Acute osteomyelitis, hand | 73004 | M86049 | | Acute hematogenous osteomyelitis, unspecified hand |
| Acute osteomyelitis, hand | 73004 | M86141 | | Other acute osteomyelitis, right hand |
| Acute osteomyelitis, hand | 73004 | M86142 | | Other acute osteomyelitis, left hand |
| Acute osteomyelitis, hand | 73004 | M86149 | | Other acute osteomyelitis, unspecified hand |
| Acute osteomyelitis, hand | 73004 | M86241 | | Subacute osteomyelitis, right hand |
| Acute osteomyelitis, hand | 73004 | M86242 | | Subacute osteomyelitis, left hand |
| Acute osteomyelitis, hand | 73004 | M86249 | | Subacute osteomyelitis, unspecified hand |
| Acute osteomyelitis, pelvic region and thigh | 73005 | M86051 | | Acute hematogenous osteomyelitis, right femur |
| Acute osteomyelitis, pelvic region and thigh | 73005 | M86052 | | Acute hematogenous osteomyelitis, left femur |
| Acute osteomyelitis, pelvic region and thigh | 73005 | M86059 | | Acute hematogenous osteomyelitis, unspecified femur |
| Acute osteomyelitis, pelvic region and thigh | 73005 | M86151 | | Other acute osteomyelitis, right femur |
| Acute osteomyelitis, pelvic region and thigh | 73005 | M86152 | | Other acute osteomyelitis, left femur |
| Acute osteomyelitis, pelvic region and thigh | 73005 | M86159 | | Other acute osteomyelitis, unspecified femur |
| Acute osteomyelitis, pelvic region and thigh | 73005 | M86251 | | Subacute osteomyelitis, right femur |
| **Appendix B Table 1-BJI: IDC-9 and ICD-10 Codes for Bone and Joint Infections cont.** | | | | |
| **ICD-9 Description** | **ICD-9_bji** | **ICD-10_bji** | | **ICD-10 Description** |
| Acute osteomyelitis, pelvic region and thigh | 73005 | M86252 | | Subacute osteomyelitis, left femur |
| Acute osteomyelitis, pelvic region and thigh | 73005 | M86259 | | Subacute osteomyelitis, unspecified femur |
| Acute osteomyelitis, lower leg | 73006 | M86061 | | Acute hematogenous osteomyelitis, right tibia and fibula |
| Acute osteomyelitis, lower leg | 73006 | M86062 | | Acute hematogenous osteomyelitis, left tibia and fibula |
| Acute osteomyelitis, lower leg | 73006 | M86069 | | Acute hematogenous osteomyelitis, unspecified tibia and fibula |
| Acute osteomyelitis, lower leg | 73006 | M86161 | | Other acute osteomyelitis, right tibia and fibula |
| Acute osteomyelitis, lower leg | 73006 | M86162 | | Other acute osteomyelitis, left tibia and fibula |
| Acute osteomyelitis, lower leg | 73006 | M86169 | | Other acute osteomyelitis, unspecified tibia and fibula |
| Acute osteomyelitis, lower leg | 73006 | M86261 | | Subacute osteomyelitis, right tibia and fibula |
| Acute osteomyelitis, lower leg | 73006 | M86262 | | Subacute osteomyelitis, left tibia and fibula |
| Acute osteomyelitis, lower leg | 73006 | M86269 | | Subacute osteomyelitis, unspecified tibia and fibula |
| Acute osteomyelitis, ankle and foot | 73007 | M86071 | | Acute hematogenous osteomyelitis, right ankle and foot |
| Acute osteomyelitis, ankle and foot | 73007 | M86072 | | Acute hematogenous osteomyelitis, left ankle and foot |
| Acute osteomyelitis, ankle and foot | 73007 | M86079 | | Acute hematogenous osteomyelitis, unspecified ankle and foot |
| Acute osteomyelitis, ankle and foot | 73007 | M86171 | | Other acute osteomyelitis, right ankle and foot |
| Acute osteomyelitis, ankle and foot | 73007 | M86172 | | Other acute osteomyelitis, left ankle and foot |
| Acute osteomyelitis, ankle and foot | 73007 | M86179 | | Other acute osteomyelitis, unspecified ankle and foot |
| Acute osteomyelitis, ankle and foot | 73007 | M86271 | | Subacute osteomyelitis, right ankle and foot |
| Acute osteomyelitis, ankle and foot | 73007 | M86272 | | Subacute osteomyelitis, left ankle and foot |
| Acute osteomyelitis, ankle and foot | 73007 | M86279 | | Subacute osteomyelitis, unspecified ankle and foot |
| Acute osteomyelitis, other specified sites | 73008 | M8608 | | Acute hematogenous osteomyelitis, other sites |
| Acute osteomyelitis, other specified sites | 73008 | M8618 | | Other acute osteomyelitis, other site |
| Acute osteomyelitis, other specified sites | 73008 | M8628 | | Subacute osteomyelitis, other site |
| Acute osteomyelitis, multiple sites | 73009 | M8609 | | Acute hematogenous osteomyelitis, multiple sites |
| Acute osteomyelitis, multiple sites | 73009 | M8619 | | Other acute osteomyelitis, multiple sites |
| Acute osteomyelitis, multiple sites | 73009 | M8629 | | Other chronic osteomyelitis, unspecified humerus |
| Unspecified osteomyelitis | 73020 | M869 | | Osteomyelitis, unspecified |
| Unspecified osteomyelitis, other specified sites | 73028 | M4620 | | Osteomyelitis of vertebra, site unspecified |
| Unspecified osteomyelitis, other specified sites | 73028 | M4621 | | Osteomyelitis of vertebra, occipito-atlanto-axial region |
| Unspecified osteomyelitis, other specified sites | 73028 | M4622 | | Osteomyelitis of vertebra, cervical region |
| Unspecified osteomyelitis, other specified sites | 73028 | M4623 | | Osteomyelitis of vertebra, cervicothoracic region |
| Unspecified osteomyelitis, other specified sites | 73028 | M4624 | | Osteomyelitis of vertebra, thoracic region |
| Unspecified osteomyelitis, other specified sites | 73028 | M4625 | | Osteomyelitis of vertebra, thoracolumbar region |
| Unspecified osteomyelitis, other specified sites | 73028 | M4626 | | Osteomyelitis of vertebra, lumbar region |
| Unspecified osteomyelitis, other specified sites | 73028 | M4627 | | Osteomyelitis of vertebra, lumbosacral region |
| Unspecified osteomyelitis, other specified sites | 73028 | M4628 | | Osteomyelitis of vertebra, sacral and sacrococcygeal region |
| Other infections involving bone in diseases classified elsewhere | 73080 | M9080 | | Osteopathy in diseases classified elsewhere, unspecified site |
| Other infections involving bone in diseases, shoulder region | 73081 | M90811 | | Osteopathy in diseases classified elsewhere, right shoulder |
| **Appendix B Table 1-BJI: IDC-9 and ICD-10 Codes for Bone and Joint Infections cont.** | | | | |
| **ICD-9 Description** | **ICD-9_bji** | **ICD-10_bji** | | **ICD-10 Description** |
| Other infections involving bone in diseases, shoulder region | 73081 | M90812 | | Osteopathy in diseases classified elsewhere, left shoulder |
| Other infections involving bone in diseases, shoulder region | 73081 | M90819 | | Osteopathy in diseases classified elsewhere, unspecified shoulder |
| Other infections involving bone in diseases, upper arm | 73082 | M90821 | | Osteopathy in diseases classified elsewhere, right upper arm |
| Other infections involving bone in diseases, upper arm | 73082 | M90822 | | Osteopathy in diseases classified elsewhere, left upper arm |
| Other infections involving bone in diseases, upper arm | 73082 | M90829 | | Osteopathy in diseases classified elsewhere, unspecified upper arm |
| Other infections involving bone in diseases, forarm | 73083 | M90831 | | Osteopathy in diseases classified elsewhere, right forearm |
| Other infections involving bone in diseases, forarm | 73083 | M90832 | | Osteopathy in diseases classified elsewhere, left forearm |
| Other infections involving bone in diseases, forarm | 73083 | M90839 | | Osteopathy in diseases classified elsewhere, unspecified forearm |
| Other infections involving bone in diseases, hand | 73084 | M90841 | | Osteopathy in diseases classified elsewhere, right hand |
| Other infections involving bone in diseases, hand | 73084 | M90842 | | Osteopathy in diseases classified elsewhere, left hand |
| Other infections involving bone in diseases, hand | 73084 | M90849 | | Osteopathy in diseases classified elsewhere, unspecified hand |
| Other infections involving bone in diseases, pelvic region and thigh | 73085 | M90851 | | Osteopathy in diseases classified elsewhere, right thigh |
| Other infections involving bone in diseases, pelvic region and thigh | 73085 | M90852 | | Osteopathy in diseases classified elsewhere, left thigh |
| Other infections involving bone in diseases, pelvic region and thigh | 73085 | M90859 | | Osteopathy in diseases classified elsewhere, unspecified thigh |
| Other infections involving bone in diseases, lower leg | 73086 | M90861 | | Osteopathy in diseases classified elsewhere, right lower leg |
| Other infections involving bone in diseases, lower leg | 73086 | M90862 | | Osteopathy in diseases classified elsewhere, left lower leg |
| Other infections involving bone in diseases, lower leg | 73086 | M90869 | | Osteopathy in diseases classified elsewhere, unspecified lower leg |
| Other infections involving bone in diseases, ankle and foot | 73087 | M90871 | | Osteopathy in diseases classified elsewhere, right ankle and foot |
| Other infections involving bone in diseases, ankle and foot | 73087 | M90872 | | Osteopathy in diseases classified elsewhere, left ankle and foot |
| Other infections involving bone in diseases, ankle and foot | 73087 | M90879 | | Osteopathy in diseases classified elsewhere, unspecified ankle and foot |
| Other infections involving bone in diseases, other specified sites | 73088 | A1801 | | Tuberculosis of spine |
| Other infections involving bone in diseases, other specified sites | 73088 | A1803 | | Tuberculosis of other bones |
| Other infections involving bone in diseases, other specified sites | 73088 | M9088 | | Osteopathy in diseases classified elsewhere, other site |
| Other infections involving bone in diseases, multiple sites | 73089 | M9089 | | Osteopathy in diseases classified elsewhere, multiple sites |
| Unspecified infection of bone | 73090 | M869 | | Osteomyelitis, unspecified |
| Unspecified infection of bone, shoulder region | 73091 | M869 | | Osteomyelitis, unspecified |
| Unspecified infection of bone, upper arm | 73092 | M869 | | Osteomyelitis, unspecified |
| Unspecified infection of bone, forarm | 73093 | M869 | | Osteomyelitis, unspecified |
| Unspecified infection of bone, hand | 73094 | M869 | | Osteomyelitis, unspecified |
| Unspecified infection of bone, pelvic region and thigh | 73095 | M869 | | Osteomyelitis, unspecified |
| Unspecified infection of bone, lower leg | 73096 | M869 | | Osteomyelitis, unspecified |
| Unspecified infection of bone, ankle and foot | 73097 | M869 | | Osteomyelitis, unspecified |
| Unspecified infection of bone, other specified sites | 73098 | M4630 | | Infection of intervertebral disc (pyogenic), site unspecified |
| Unspecified infection of bone, other specified sites | 73098 | M4631 | | Infection of intervertebral disc (pyogenic), occipito-atlanto-axial region |
| Unspecified infection of bone, other specified sites | 73098 | M4632 | | Infection of intervertebral disc (pyogenic), cervical region |
| Unspecified infection of bone, other specified sites | 73098 | M4633 | | Infection of intervertebral disc (pyogenic), cervicothoracic region |
| Unspecified infection of bone, other specified sites | 73098 | M4634 | | Infection of intervertebral disc (pyogenic), thoracic region |
| Unspecified infection of bone, other specified sites | 73098 | M4635 | | Infection of intervertebral disc (pyogenic), thoracolumbar region |
| **Appendix B Table 1-BJI: IDC-9 and ICD-10 Codes for Bone and Joint Infections cont.** | | | | |
| **ICD-9 Description** | **ICD-9_bji** | **ICD-10_bji** | | **ICD-10 Description** |
| Unspecified infection of bone, other specified sites | 73098 | M4636 | | Infection of intervertebral disc (pyogenic), lumbar region |
| Unspecified infection of bone, other specified sites | 73098 | M4637 | | Infection of intervertebral disc (pyogenic), lumbosacral region |
| Unspecified infection of bone, other specified sites | 73098 | M4638 | | Infection of intervertebral disc (pyogenic), sacral and sacrococcygeal region |
| Unspecified infection of bone, other specified sites | 73098 | M4639 | | Infection of intervertebral disc (pyogenic), multiple sites in spine |
| Unspecified infection of bone, other specified sites | 73098 | M869 | | Osteomyelitis, unspecified |
| Unspecified infection of bone, multiple sites | 73099 | M869 | | Osteomyelitis, unspecified |
| Other complications due to internal joint prosthesis | 99677 | T8481XA | | Embolism due to internal orthopedic prosthetic devices, implants and grafts, initial encounter |
| Other complications due to internal joint prosthesis | 99677 | T8482XA | | Fibrosis due to internal orthopedic prosthetic devices, implants and grafts, initial encounter |
| Other complications due to internal joint prosthesis | 99677 | T8483XA | | Hemorrhage due to internal orthopedic prosthetic devices, implants and grafts, initial encounter |
| Other complications due to internal joint prosthesis | 99677 | T8484XA | | Pain due to internal orthopedic prosthetic devices, implants and grafts, initial encounter |
| Other complications due to internal joint prosthesis | 99677 | T8485XA | | Stenosis due to internal orthopedic prosthetic devices, implants and grafts, initial encounter |
| Other complications due to internal joint prosthesis | 99677 | T8486XA | | Thrombosis due to internal orthopedic prosthetic devices, implants and grafts, initial encounter |
| Other complications due to internal joint prosthesis | 99677 | T8489XA | | Other specified complication of internal orthopedic prosthetic devices, implants and grafts, initial encounter |
| Other complications due to internal joint prosthesis | 99677 | T849XXA | | Unspecified complication of internal orthopedic prosthetic device, implant and graft, initial encounter |

**Appendix B Table 2-BJI: Univariate Statistics 2011 and 2016**

| **Patient Characteristics** | **BJI - Group Comparisons 2011** | | | **BJI - Group Comparisons 2016** | | |
| --- | --- | --- | --- | --- | --- | --- |
|  | **Cases with Secondary Dx, LOS > 2 days** | **Cases with Principal DX** | **Matched Cases with No Dx** | **Cases with Secondary Dx, LOS > 2 days** | **Cases with Principal DX** | **Matched Cases with No Dx** |
|  |  |  |  |  |  |  |
|  |  |  |  |  |  |  |
| **n =** | 42,552 | 21,776 | 212,760 | 42,549 | 22,409 | 212,745 |
| **Variables:** |  |  |  |  |  |  |
| **Cost** |  |  |  |  |  |  |
| **mean** | $26,375 | $17,650 | $14,452 | $25,355 | $16,768 | $14,721 |
| **median** | $16,739 | $11,302 | $8,389 | $16,641 | $11,836 | $8,807 |
| **mode** | $11,192 | $1,068 | $78 | $18,916 | $3,148 | $3,148 |
| **range** | $195 - $1,172,737 | $145 - $724,544 | $30 - $1,377,091 | $42 - $1,683,604 | $46 - $514,430 | $24 - $1,606,412 |
| **LOS** |  |  |  |  |  |  |
| **mean** | 11.55 | 7.74 | 5.77 | 10.76 | 6.77 | 5.69 |
| **median** | 8 | 5 | 4 | 7 | 5 | 4 |
| **mode** | 3 | 3 | 2 | 3 | 3 | 2 |
| **range** | 3 - 365 | 1 - 254 | 1 - 355 | 3 - 363 | 1 - 326 | 1 - 360 |
| **Number of Diagnoses; mean (SD)** | 15.51 (6.33) | 10.65 (6.10) | 11.52 (6.07) | 17.69 (6.41) | 12.43 (6.52) | 13.80 (6.71) |
| **Number of Procedures; mean (SD)** | 3.37 (3.10) | 2.42 (2.27) | 1.72 (2.39) | 3.45 (3.12) | 2.54 (2.22) | 1.69 (2.42) |
| **age; mean (range)** | 61 (0 - 103) | 56 (0 -104) | 58 (0-114) | 60 (0 - 90) | 57 (0 -90) | 59 (0-90) |
| **APRDRG_Severity; mean (SD)** | 3.04 (0.73) | 2.37 (0.86) | 2.52 (0.92) | 2.97 (0.73) | 2.19 (0.85) | 2.51 (0.91) |
| **APRDRG_Risk_Mortality; mean (SD)** | 2.38 (0.98) | 1.72 (0.89) | 2.10 (1.02) | 2.37 (0.97) | 1.68 (0.82) | 2.20 (1.05) |
| **Wage Index; mean (SD)** | 1.0080 (0.155) | 0.9952 (0.155) | 1.0017 (0.159) | 1.0039 (0.191) | 1.0001 (0.190) | 1.0013 (0.196) |

**Appendix B Table 3-BJI: Results of Median Regression Models 2011 and 2016**

| **Median Regression Estimates for Cost of BJI as a Principal Diagnosis (n=21769) 2011** | | | | | | | **Median Regression Estimates for Cost of BJI as a Primary Diagnosis (n=22407) 2016** | | | | | | |
| --- | --- | --- | --- | --- | --- | --- | --- | --- | --- | --- | --- | --- | --- |
| **Parameter** | **Estimate** | **Standard Error** | **95% Confidence Limits** | | **t Value** | **Pr > \|t\|** | **Parameter** | **Estimate** | **Standard Error** | **95% Confidence Limits** | | **t Value** | **Pr > \|t\|** |
| **Intercept** | -8651.55 | 319.362 | -9277.518 | -8025.573 | -27.09 | <.0001 | **Intercept** | -7278.62 | 286.0456 | -7839.294 | -6717.955 | -25.45 | <.0001 |
| **LOS** | 1151.825 | 16.2874 | 1119.9005 | 1183.7496 | 70.72 | <.0001 | **LOS** | 1108.387 | 17.1203 | 1074.8302 | 1141.9441 | 64.74 | <.0001 |
| **NDX** | 53.5041 | 12.6016 | 28.8041 | 78.2042 | 4.25 | <.0001 | **NDX** | 4.7677 | 8.2901 | -11.4814 | 21.0168 | 0.58 | 0.5652 |
| **NPR** | 1918.271 | 39.744 | 1840.3701 | 1996.1725 | 48.27 | <.0001 | **NPR** | 1963.745 | 32.1826 | 1900.6652 | 2026.8256 | 61.02 | <.0001 |
| **URBAN_TEACH** | 108.3387 | 74.4866 | -37.6605 | 254.3379 | 1.45 | 0.1458 | **URBAN_TEACH** | 86.5112 | 72.3674 | -55.3341 | 228.3564 | 1.2 | 0.2319 |
| **URBAN_TEACH** | 0 | 0 | 0 | 0 | . | . | **URBAN_TEACH** | 0 | 0 | 0 | 0 | . | . |
| **SMALL_BEDSIZE** | -952.928 | 127.3678 | -1202.578 | -703.2774 | -7.48 | <.0001 | **SMALL_BEDSIZE** | -547.248 | 91.5466 | -726.686 | -367.8107 | -5.98 | <.0001 |
| **SMALL_BEDSIZE** | 0 | 0 | 0 | 0 | . | . | **SMALL_BEDSIZE** | 0 | 0 | 0 | 0 | . | . |
| **AGE** | -0.6464 | 2.1054 | -4.7732 | 3.4803 | -0.31 | 0.7588 | **AGE** | 5.3549 | 2.2063 | 1.0303 | 9.6795 | 2.43 | 0.0152 |
| **APRDRG_Severity** | 657.6537 | 82.3624 | 496.2175 | 819.0899 | 7.98 | <.0001 | **APRDRG_Severity** | 340.7198 | 74.454 | 194.7847 | 486.6549 | 4.58 | <.0001 |
| **APRDRG_Risk_Mortality** | -132.217 | 86.6704 | -302.0971 | 37.6634 | -1.53 | 0.1271 | **APRDRG_Risk_Mortality** | 2.8575 | 77.625 | -149.293 | 155.008 | 0.04 | 0.9706 |
| **WI_X** | 8924.141 | 297.0027 | 8341.9944 | 9506.2882 | 30.05 | <.0001 | **WI_X** | 8512.411 | 268.2197 | 7986.6817 | 9038.1406 | 31.74 | <.0001 |

**Specified Stage 2 Cost Simulation Models for BJI 2011 and 2016**

**Cost_bji_2011 = -8651.55 + 1151.825*7.74320353 + 53.5041*NDX + 1918.271*NPR + 108.3387*URBAN_TEACH + -952.928*SMALL_BEDSIZE +**

**-0.6464*AGE + 657.6537*APRDRG_Severity + -132.217*APRDRG_Risk_Mortality + 8924.141*Wage_Index**

**Cost_bji_2016 = -7278.62 + 1108.387*6.771208 + 4.7677*NDX + 1963.745*NPR + 86.5112*URBAN_TEACH + -547.248*SMALL_BEDSIZE +**

**5.3549*AGE + 340.7198*APRDRG_Severity + 2.8575*APRDRG_Risk_Mortality + 8512.411*Wage_Index**

**Primary Bloodstream Infections (BSI)**

| **Appendix B Table 1-BSI: IDC-9 and ICD-10 Codes for Primary Bloodstream Infections** | | |  |
| --- | --- | --- | --- |
| **ICD-9 Description** | **icd9_bsi** | **icd10_bsi** | **ICD-10 Description** |
| Bubonic | 0200 | A200 | Bubonic plague |
| Streptococcal septicemia | 0380 | A400 | Sepsis due to streptococcus, group A |
| Streptococcal septicemia | 0380 | A401 | Sepsis due to streptococcus, group B |
| Streptococcal septicemia | 0380 | A403 | Sepsis due to Streptococcus pneumoniae |
| Streptococcal septicemia | 0380 | A408 | Other streptococcal sepsis |
| Streptococcal septicemia | 0380 | A409 | Streptococcal sepsis, unspecified |
| Staphylcoccoal septicemia, unspecified | 03810 | A412 | Sepsis due to unspecified staphylococcus |
| Methicillin susceptible Staphylcoccus aureus septicemia | 03811 | A4101 | Sepsis due to Methicillin susceptible Staphylococcus aureus |
| Methicillin resistant Staphylococcus aureus septicemia | 03812 | A4102 | Sepsis due to Methicillin resistant Staphylococcus aureus |
| Other staphylococcal septicemia | 03819 | A411 | Sepsis due to other specified staphylococcus |
| Pneumococcal septicemia | 0382 | A403 | Sepsis due to Streptococcus pneumoniae |
| Septicemia due to anaerobes | 0383 | A414 | Sepsis due to anaerobes |
| Septicemia due to gram-negative organism, unspecified | 03840 | A4150 | Gram-negative sepsis, unspecified |
| Septicemia due to H. flu | 03841 | A413 | Sepsis due to Hemophilus influenzae |
| septicemia due to escherichia coli | 03842 | A4151 | Sepsis due to Escherichia coli [E. coli] |
| Septicemia due to pseudomonas | 03843 | A4152 | Sepsis due to Pseudomonas |
| Septicemia due to serratia | 03844 | A4153 | Sepsis due to Serratia |
| Other septicemia due to gram-negative organisms | 03849 | A4159 | Other Gram-negative sepsis |
| Other specified septicemias (actinomycotic, enterococcus, chromobacter…) | 0388 | A4181 | Sepsis due to Enterococcus |
| Other specified septicemias (actinomycotic, enterococcus, chromobacter…) | 0388 | A4189 | Other specified sepsis |
| Other specified septicemias (actinomycotic, enterococcus, chromobacter…) | 0388 | A427 | Actinomycotic sepsis |
| Unspecified septicemia | 0389 | A419 | Sepsis, unspecified organism |
| Disseminated candidiasis (including candidemia assocaited with an intravascular line, line related, or due to a central line) | 1125 | B377 | Candidal sepsis |
| Candidal endocarditis | 11281 | B376 | Candidal endocarditis |
| Other and unspecified mycoses (which includes fungemia) | 1179 | B483 | Geotrichosis |
| Other and unspecified mycoses (which includes fungemia) | 1179 | B488 | Other specified mycoses |
| Other and unspecified mycoses (which includes fungemia) | 1179 | B49 | Unspecified mycosis |
| Bacteremia | 7907 | R7881 | Bacteremia |
| Systemic inflammatory response syndrome, unspecified | 99590 | n/a |  |
| Sepsis | 99591 | A021 | Salmonella sepsis |
| Sepsis | 99591 | A227 | Anthrax sepsis |
| Sepsis | 99591 | A267 | Erysipelothrix sepsis |
| Sepsis | 99591 | A327 | Listerial sepsis |
| Sepsis | 99591 | A400 | Sepsis due to streptococcus, group A |
| **Appendix B Table 1-BSI: IDC-9 and ICD-10 Codes for Primary Bloodstream Infections cont.** | | | |
| **ICD-9 Description** | **icd9_bsi** | **icd10_bsi** | **ICD-10 Description** |
| Sepsis | 99591 | A401 | Sepsis due to streptococcus, group B |
| Sepsis | 99591 | A403 | Sepsis due to Streptococcus pneumoniae |
| Sepsis | 99591 | A408 | Other streptococcal sepsis |
| Sepsis | 99591 | A409 | Streptococcal sepsis, unspecified |
| Sepsis | 99591 | A4101 | Sepsis due to Methicillin susceptible Staphylococcus aureus |
| Sepsis | 99591 | A4102 | Sepsis due to Methicillin resistant Staphylococcus aureus |
| Sepsis | 99591 | A411 | Sepsis due to other specified staphylococcus |
| Sepsis | 99591 | A412 | Sepsis due to unspecified staphylococcus |
| Sepsis | 99591 | A413 | Sepsis due to Hemophilus influenzae |
| Sepsis | 99591 | A414 | Sepsis due to anaerobes |
| Sepsis | 99591 | A414 | Sepsis due to anaerobes |
| Sepsis | 99591 | A4150 | Gram-negative sepsis, unspecified |
| Sepsis | 99591 | A4150 | Gram-negative sepsis, unspecified |
| Sepsis | 99591 | A4150 | Gram-negative sepsis, unspecified |
| Sepsis | 99591 | A4151 | Sepsis due to Escherichia coli [E. coli] |
| Sepsis | 99591 | A4152 | Sepsis due to Pseudomonas |
| Sepsis | 99591 | A4159 | Other Gram-negative sepsis |
| Sepsis | 99591 | A4181 | Sepsis due to Enterococcus |
| Sepsis | 99591 | A4189 | Other specified sepsis |
| Sepsis | 99591 | A419 | Sepsis, unspecified organism |
| Sepsis | 99591 | A427 | Actinomycotic sepsis |
| Sepsis | 99591 | A4153 | Sepsis due to Serratia |
| Sepsis | 99591 | A5486 | Gonococcal sepsis |
| Sepsis | 99591 | B377 | Candida sepsis |
| Other and unspecified infection due to central venous catheter | 99931 | T80218A | Other infection due to central venous catheter, initial encounter |
| Other and unspecified infection due to central venous catheter | 99931 | T80219A | Unspecified infection due to central venous catheter, initial encounter |
| Bloodstream infection due to central venous catheter | 99932 | T80211A | Bloodstream infection due to central venous catheter, initial encounter |
| Other specified bacterial infections in conditions classified elsewhere and of unspecified site, other gram-negative organisms | 04185 | B9689 | Other specified bacterial agents as the cause of diseases classified elsewhere |
| Bacterial infection, unspecified, in conditions classified elsewhere and of unspecified site (includes gram positive bacteremia) | 0419 | A499 | Bacterial infection, unspecified |
| Bacterial infection, unspecified, in conditions classified elsewhere and of unspecified site (includes gram positive bacteremia) | 0419 | B9689 | Other specified bacterial agents as the cause of diseases classified elsewhere |

**Appendix B Table 2-BSI: Univariate Statistics 2011 and 2016**

| **Patient Characteristics** | **BSI - Group Comparisons 2011** | | | **BSI - Group Comparisons 2016** | | |
| --- | --- | --- | --- | --- | --- | --- |
|  | **Cases with Secondary Dx, LOS > 2 days** | **Cases with Principal DX** | **Matched Cases with No Dx** | **Cases with Secondary Dx, LOS > 2 days** | **Cases with Principal DX** | **Matched Cases with No Dx** |
|  |  |  |  |  |  |  |
|  |  |  |  |  |  |  |
| **n =** | 146,190 | 217,950 | 728,674 | 132,732 | 377,953 | 656,091 |
| **Variables:** |  |  |  |  |  |  |
| **Cost** |  |  |  |  |  |  |
| **mean** | $41,308 | $21,419 | $13,909 | $40,143 | $18,267 | $14,258 |
| **median** | $21,190 | $12,653 | $8,684 | $19,259 | $11,007 | $8,737 |
| **mode** | $5,928 | $5,535 | $64,032 | $9,445 | $6,297 | $3,148 |
| **range** | $34 - $1,756,572 | $34 - $1,258,04 | $33 - $1,353,867 | $34 - $2,850,000 | $22 - $1,636,440 | $19 - $2,831,796 |
| **LOS** |  |  |  |  |  |  |
| **mean** | 14.73 | 8.23 | 5.39 | 13.83 | 7.36 | 5.17 |
| **median** | 9 | 6 | 4 | 8 | 5 | 3 |
| **mode** | 3 | 3 | 2 | 3 | 3 | 2 |
| **range** | 3 - 365 | 1 -323 | 1 - 365 | 3 - 365 | 1 -340 | 1 - 36053 |
| **Number of Diagnoses; mean (SD)** | 16.76 (6.20) | 15.74 (6.02) | 10.52 (5.69) | 18.72 (6.38) | 16.89 (6.39) | 12.40 (6.44) |
| **Number of Procedures; mean (SD)** | 3.94 (3.94) | 2.22 (2.83) | 1.78 (2.29) | 3.76 (4.02) | 1.90 (2.72) | 1.77 (2.31) |
| **age; mean (range)** | 62 (0 -110) | 66 (0 -111) | 59 (0-121) | 61 (0 - 90) | 64 (0 -90) | 59 (0-90) |
| **APRDRG_Severity; mean (SD)** | 3.46 (0.68) | 3.32 (0.79) | 2.38 (0.89) | 3.35 (0.75) | 3.10 (0.83) | 2.31 (0.86) |
| **APRDRG_Risk_Mortality; mean (SD)** | 3.10 (0.96) | 3.12 (0.97) | 1.95 (0.93) | 3.08 (0.97) | 2.98 (1.00) | 1.97 (0.93) |
| **Wage Index; mean (SD)** | 1.0016 (0.155) | 1.0050 (0.161) | 1.0002 (0.157) | 1.0050 (0.20) | 1.0052 (0.201) | 1.0023 (0.194) |

**Appendix B Table 3-BSI: Results of Median Regression Models 2011 and 2016**

| **Median Regression Estimates for Cost of BSI as a Principal Diagnosis (n= 217934) 2011** | | | | | | | **Median Regression Estimates for Cost of BSI as a Principal Diagnosis (n=377895) 2016** | | | | | | |
| --- | --- | --- | --- | --- | --- | --- | --- | --- | --- | --- | --- | --- | --- |
| **Parameter** | **Estimate** | **Standard Error** | **95% Confidence Limits** | | **t Value** | **Pr > \|t\|** | **Parameter** | **Estimate** | **Standard Error** | **95% Confidence Limits** | | **t Value** | **Pr > \|t\|** |
| **Intercept** | -11794.4 | 107.2514 | -12004.65 | -11584.23 | -109.97 | <.0001 | **Intercept** | -8105.46 | 55.0966 | -8213.449 | -7997.474 | -147.11 | <.0001 |
| **LOS** | 1701.719 | 6.2587 | 1689.4519 | 1713.9858 | 271.89 | <.0001 | **LOS** | 1626.448 | 4.6842 | 1617.2675 | 1635.6294 | 347.22 | <.0001 |
| **NDX** | 63.3219 | 3.0736 | 57.2976 | 69.3461 | 20.6 | <.0001 | **NDX** | 33.7845 | 1.5535 | 30.7397 | 36.8293 | 21.75 | <.0001 |
| **NPR** | 1923.993 | 13.4348 | 1897.6612 | 1950.3251 | 143.21 | <.0001 | **NPR** | 1721.249 | 9.1925 | 1703.2318 | 1739.2659 | 187.24 | <.0001 |
| **URBAN_TEACH** | 1310.756 | 27.0462 | 1257.7465 | 1363.7663 | 48.46 | <.0001 | **URBAN_TEACH** | 722.6558 | 15.6399 | 692.0020 | 753.3096 | 46.21 | <.0001 |
| **URBAN_TEACH** | 0 | 0 | 0 | 0 | . | . | **URBAN_TEACH** | 0.0000 | 0.0000 | 0.0000 | 0.0000 | . | . |
| **SMALL_BEDSIZE** | -1145.68 | 41.2732 | -1226.572 | -1064.784 | -27.76 | <.0001 | **SMALL_BEDSIZE** | -539.963 | 18.2780 | -575.7873 | -504.1386 | -29.54 | <.0001 |
| **SMALL_BEDSIZE** | 0 | 0 | 0 | 0 | . | . | **SMALL_BEDSIZE** | 0.0000 | 0.0000 | 0.0000 | 0.0000 | . | . |
| **AGE** | -32.4587 | 0.7541 | -33.9367 | -30.9806 | -43.04 | <.0001 | **AGE** | -28.4938 | 0.4341 | -29.3447 | -27.6430 | -65.64 | <.0001 |
| **APRDRG_Severity** | 113.6498 | 25.4529 | 63.7627 | 163.5369 | 4.47 | <.0001 | **APRDRG_Severity** | 331.8231 | 16.0615 | 300.3431 | 363.3031 | 20.66 | <.0001 |
| **APRDRG_Risk_Mortality** | 715.1107 | 23.1445 | 669.7481 | 760.4733 | 30.9 | <.0001 | **APRDRG_Risk_Mortality** | 577.8419 | 12.8615 | 552.6338 | 603.0501 | 44.93 | <.0001 |
| **Wage_Index** | 11330 | 96.3002 | 11141.257 | 11518.749 | 117.65 | <.0001 | **Wage_Index** | 8052.545 | 42.0126 | 7970.2013 | 8134.8882 | 191.67 | <.0001 |

**Specified Stage 2 Cost Simulation Models for BSI 2011 and 2016**

**Cost_bsi_2011 = -11794.4 + 1701.719*8.22854324 + 63.3219*NDX + 1923.993*NPR + 1310.756*URBAN_TEACH + (-1145.68)*SMALL_BEDSIZE +**

**(-32.4587)*AGE + 113.6498*APRDRG_Severity + 715.1107*APRDRG_Risk_Mortality + 11330*Wage_Index**

**Cost_bsi_2016 = -8105.46+ 1626.448*7.35943887 + 33.7845*NDX + 1721.249*NPR + 722.6558*URBAN_TEACH + (-539.963)*SMALL_BEDSIZE +**

**(-28.4938)*AGE + 331.8231*APRDRG_Severity + 577.8419*APRDRG_Risk_Mortality + 8052.545* Wage_Index**

**Catheter-Associated Urinary Tract Infection (CAUTI)**

| **Appendix B Table 1-CAUTI: IDC-9 and ICD-10 Codes for Catheter-Associated Urinary Tract Infections** | | | | | |
| --- | --- | --- | --- | --- | --- |
| **ICD-9 Description** | **icd9_cauti** | **icd10_cauti** | **ICD-10 Description** | | |
| Candidiasis of urinary tract | 1122 | B3741 | Candidal cystitis and urethritis | | |
| Candidiasis of urinary tract | 1122 | B3742 | Candidal balanitis | | |
| Candidiasis of urinary tract | 1122 | B3749 | Other urogenital candidiasis | | |
| Urinary tract infection of newborn | 77182 | P393 | Neonatal urinary tract infection | | |
| Infections of genitourinary tract in pregnancy, unspecified as to episode of care or not applicable | 64660 | O2300 | Infections of kidney in pregnancy, unspecified trimester | | |
| Infections of genitourinary tract in pregnancy, unspecified as to episode of care or not applicable | 64660 | O2310 | Infections of bladder in pregnancy, unspecified trimester | | |
| Infections of genitourinary tract in pregnancy, unspecified as to episode of care or not applicable | 64660 | O2320 | Infections of urethra in pregnancy, unspecified trimester | | |
| Infections of genitourinary tract in pregnancy, unspecified as to episode of care or not applicable | 64660 | O2330 | Infections of other parts of urinary tract in pregnancy, unspecified trimester | | |
| Infections of genitourinary tract in pregnancy, unspecified as to episode of care or not applicable | 64660 | O2340 | Unspecified infection of urinary tract in pregnancy, unspecified trimester | | |
| Infections of genitourinary tract in pregnancy, unspecified as to episode of care or not applicable | 64660 | O23519 | Infections of cervix in pregnancy, unspecified trimester | | |
| Infections of genitourinary tract in pregnancy, unspecified as to episode of care or not applicable | 64660 | O23529 | Salpingo-oophoritis in pregnancy, unspecified trimester | | |
| Infections of genitourinary tract in pregnancy, unspecified as to episode of care or not applicable | 64660 | O23599 | Infection of other part of genital tract in pregnancy, unspecified trimester | | |
| Infections of genitourinary tract in pregnancy, unspecified as to episode of care or not applicable | 64660 | O2390 | Unspecified genitourinary tract infection in pregnancy, unspecified trimester | | |
| Infections of the genitourinary tract in pregnancy, antepartum condition or complication | 64663 | O2301 | Infections of kidney in pregnancy, first trimester | | |
| Infections of the genitourinary tract in pregnancy, antepartum condition or complication | 64663 | O2302 | Infections of kidney in pregnancy, second trimester | | |
| Infections of the genitourinary tract in pregnancy, antepartum condition or complication | 64663 | O2303 | Infections of kidney in pregnancy, third trimester | | |
| Infections of the genitourinary tract in pregnancy, antepartum condition or complication | 64663 | O2311 | Infections of bladder in pregnancy, first trimester | | |
| Infections of the genitourinary tract in pregnancy, antepartum condition or complication | 64663 | O2312 | Infections of bladder in pregnancy, second trimester | | |
| Infections of the genitourinary tract in pregnancy, antepartum condition or complication | 64663 | O2313 | Infections of bladder in pregnancy, third trimester | | |
| Infections of the genitourinary tract in pregnancy, antepartum condition or complication | 64663 | O2321 | Infections of urethra in pregnancy, first trimester | | |
| **Appendix B Table 1-CAUTI: IDC-9 and ICD-10 Codes for Catheter-Associated Urinary Tract Infections cont.** | | | |  |  |
| **ICD-9 Description** | **icd9_cauti** | **icd10_cauti** | **ICD-10 Description** | |  |
| Infections of the genitourinary tract in pregnancy, antepartum condition or complication | 64663 | O2322 | Infections of urethra in pregnancy, second trimester | | |
| Infections of the genitourinary tract in pregnancy, antepartum condition or complication | 64663 | O2323 | Infections of urethra in pregnancy, third trimester | | |
| Infections of the genitourinary tract in pregnancy, antepartum condition or complication | 64663 | O2331 | Infections of other parts of urinary tract in pregnancy, first trimester | | |
| Infections of the genitourinary tract in pregnancy, antepartum condition or complication | 64663 | O2332 | Infections of other parts of urinary tract in pregnancy, second trimester | | |
| Infections of the genitourinary tract in pregnancy, antepartum condition or complication | 64663 | O2333 | Infections of other parts of urinary tract in pregnancy, third trimester | | |
| Infections of the genitourinary tract in pregnancy, antepartum condition or complication | 64663 | O2341 | Unspecified infection of urinary tract in pregnancy, first trimester | | |
| Infections of the genitourinary tract in pregnancy, antepartum condition or complication | 64663 | O2342 | Unspecified infection of urinary tract in pregnancy, second trimester | | |
| Infections of the genitourinary tract in pregnancy, antepartum condition or complication | 64663 | O2343 | Unspecified infection of urinary tract in pregnancy, third trimester | | |
| Infections of the genitourinary tract in pregnancy, antepartum condition or complication | 64663 | O23511 | Infections of cervix in pregnancy, first trimester | | |
| Infections of the genitourinary tract in pregnancy, antepartum condition or complication | 64663 | O23512, | Infections of cervix in pregnancy, second trimester | | |
| Infections of the genitourinary tract in pregnancy, antepartum condition or complication | 64663 | O23513 | Infections of cervix in pregnancy, third trimester | | |
| Infections of the genitourinary tract in pregnancy, antepartum condition or complication | 64663 | O23522 | Salpingo-oophoritis in pregnancy, second trimester | | |
| Infections of the genitourinary tract in pregnancy, antepartum condition or complication | 64663 | O23523 | Salpingo-oophoritis in pregnancy, third trimester | | |
| Infections of the genitourinary tract in pregnancy, antepartum condition or complication | 64663 | O23591 | Infection of other part of genital tract in pregnancy, first trimester | | |
| Infections of the genitourinary tract in pregnancy, antepartum condition or complication | 64663 | O23592 | Infection of other part of genital tract in pregnancy, second trimester | | |
| Infections of the genitourinary tract in pregnancy, antepartum condition or complication | 64663 | O23593 | Infection of other part of genital tract in pregnancy, third trimester | | |
| Infections of the genitourinary tract in pregnancy, antepartum condition or complication | 64663 | O2390 | Unspecified genitourinary tract infection in pregnancy, unspecified trimester | | |
| Infections of the genitourinary tract in pregnancy, antepartum condition or complication | 64663 | O2391 | Unspecified genitourinary tract infection in pregnancy, first trimester | | |
| Infections of the genitourinary tract in pregnancy, antepartum condition or complication | 64663 | O2392 | Unspecified genitourinary tract infection in pregnancy, second trimester | | |
| **Appendix B Table 1-CAUTI: IDC-9 and ICD-10 Codes for Catheter-Associated Urinary Tract Infections cont.** | | | | | |
| **ICD-9 Description** | **icd9_cauti** | **icd10_cauti** | **ICD-10 Description** | | |
| Infections of the genitourinary tract in pregnancy, antepartum condition or complication | 64663 | O2393 | Unspecified genitourinary tract infection in pregnancy, third trimester | | |
| Infection and inflammatory reaction due to indwelling urinary catheter | 99664 | T83510A | Infection and inflammatory reaction due to cystostomy catheter, initial encounter | | |
| Infection and inflammatory reaction due to indwelling urinary catheter | 99664 | T83511A | Infection and inflammatory reaction due to indwelling urethral catheter, initial encounter | | |
| Infection and inflammatory reaction due to indwelling urinary catheter | 99664 | T83512A | Infection and inflammatory reaction due to nephrostomy catheter, initial encounter | | |
| Infection and inflammatory reaction due to indwelling urinary catheter | 99664 | T83518A | Infection and inflammatory reaction due to other urinary catheter, initial encounter | | |
| Acute pyelonephritis without lesion of renal medullary necrosis | 59010 | N10 | Acute pyelonephritis | | |
| Acute pyelonephritis with lesion of renal medullary necrosis | 59011 | N10 | Acute pyelonephritis | | |
| Renal and perinephric abscess | 5902 | N151 | Renal and perinephric abscess | | |
| Pyeloureteritis cystica | 5903 | N2884 | Pyelitis cystica | | |
| Pyeloureteritis cystica | 5903 | N2885 | Pyeloureteritis cystica | | |
| Pyeloureteritis cystica | 5903 | N2886 | Ureteritis cystica | | |
| Pyelonephritis, unspecified | 59080 | N119 | Chronic tubulo-interstitial nephritis, unspecified | | |
| Pyelonephritis, unspecified | 59080 | N12 | Tubulo-interstitial nephritis, not specified as acute or chronic | | |
| Pyelonephritis, unspecified | 59080 | N136 | Pyonephrosis | | |
| Pyelitis or pyelonephritis in diseases classified elsewhere | 59081 | N16 | Renal tubulo-interstitial disorders in diseases classified elsewhere | | |
| Infection of kidney, unspecified | 5909 | n/a |  | | |
| Acute cystitis | 5950 | N3000 | Acute cystitis without hematuria | | |
| Acute cystitis | 5950 | N3001 | Acute cystitis with hematuria | | |
| Cystitis in diseases classified elsewhere | 5954 | A5601 | Chlamydial cystitis and urethritis | | |
| Cystitis in diseases classified elsewhere | 5954 | N3080 | Other cystitis without hematuria | | |
| Cystitis in diseases classified elsewhere | 5954 | N3081 | Other cystitis with hematuria | | |
| Cystitis, unspecified | 5959 | N3090 | Cystitis, unspecified without hematuria | | |
| Cystitis, unspecified | 5959 | N3091 | Cystitis, unspecified with hematuria | | |
| Uretethral abscess | 5970 | N340 | Urethral abscess | | |
| Urethritis, unspecified | 59780 | N341 | Nonspecific urethritis | | |
| Urethritis, unspecified | 59780 | N342 | Other urethritis | | |
| Other urethritis | 59789 | N343 | Urethral syndrome, unspecified | | |
| Urethral stricture due to unspecified infection | 59800 | N35111 | Postinfective urethral stricture, not elsewhere classified, male, meatal | | |
| Urethral stricture due to unspecified infection | 59800 | N35112 | Postinfective bulbous urethral stricture, not elsewhere classified, male | | |
| Urethral stricture due to unspecified infection | 59800 | N35113 | Postinfective membranous urethral stricture, not elsewhere classified, male | | |
| **Appendix B Table 1-CAUTI: IDC-9 and ICD-10 Codes for Catheter-Associated Urinary Tract Infections cont.** | | | | | |
| **ICD-9 Description** | **icd9_cauti** | **icd10_cauti** | **ICD-10 Description** | | |
| Urethral stricture due to unspecified infection | 59800 | N35114 | Postinfective anterior urethral stricture, not elsewhere classified, male | | |
| Urethral stricture due to unspecified infection | 59800 | N35119 | Postinfective urethral stricture, not elsewhere classified, male, unspecified | | |
| Urethral stricture due to unspecified infection | 59800 | N3512 | Postinfective urethral stricture, not elsewhere classified, female | | |
| Urethral stricture due to infective disease classified elsewhere | 59801 | N37 | Urethral disorders in diseases classified elsewhere | | |
| Urinary tract infection, site not specified | 5990 | N390 | Urinary tract infection, site not specified | | |
| Urinary (tract) infection | V1302 | n/a |  | | |

**Appendix B Table 2-CAUTI: Univariate Statistics 2011 and 2016**

| **Patient Characteristics** | **CAUTI - Group Comparisons 2011** | | | **CAUTI - Group Comparisons 2016** | | |
| --- | --- | --- | --- | --- | --- | --- |
|  | **Cases with Secondary Dx, LOS > 2 days** | **Cases with Principal DX** | **Matched Cases with No Dx** | **Cases with Secondary Dx, LOS > 2 days** | **Cases with Principal DX** | **Matched Cases with No Dx** |
|  |  |  |  |  |  |  |
|  |  |  |  |  |  |  |
| **n =** | 9,064 | 2,230 | 26,320 | 6,253 | 1,163 | 17,253 |
| **Variables:** |  |  |  |  |  |  |
| **Cost** |  |  |  |  |  |  |
| **mean** | $23,879 | $9,852 | $18,756 | $24,168 | $9,601 | $20,145 |
| **median** | $14,689 | $7,312 | $11,820 | $14,784 | $7,327 | $2,962 |
| **mode** | $4,931 | $6,527 | $4,488 | $5,718 | n/a | $5988 |
| **range** | $776 -$1,087,189 | $893- $149,413 | $349 - $779,678 | $561 -$686,186 | $439- $173,095 | $22 - $940,249 |
| **LOS** |  |  |  |  |  |  |
| **mean** | 9.71 | 5.24 | 6.39 | 10.48 | 5.26 | 7.16 |
| **median** | 7 | 4 | 5 | 7 | 4 | 5 |
| **mode** | 3 | 3 | 3 | 4 | 3 | 3 |
| **range** | 3 - 280 | 1 - 67 | 1 - 297 | 3 - 250 | 1 - 68 | 1 -218 |
| **Number of Diagnoses; mean (SD)** | 16.91 (6.66) | 14.30 (5.47) | 13.55 (6.00) | 19.73 (5.92) | 15.85 (5.99) | 16.46 (6.86) |
| **Number of Procedures; mean (SD)** | 3.74 (3.15) | 2.04 (1.69) | 3.61 (2.91) | 3.88 (3.32) | 2.02 (1.75) | 3.91 (3.22) |
| **age; mean (range)** | 73 (0 - 104) | 74 (0 - 103) | 68 (0-111) | 72 (0 - 90) | 73 (0 -90) | 67 (0-90) |
| **APRDRG_Severity; mean (SD)** | 3.21 (0.72) | 2.79 (0.75) | 2.80 (0.92) | 3.10 (0.79) | 2.60 (0.76) | 2.80 (0.96) |
| **APRDRG_Risk_Mortality; mean (SD)** | 2.92 (0.91) | 2.45 (0.89) | 2.51 (1.05) | 3.00 (0.88) | 2.40 (0.87) | 2.64 (1.07) |
| **Wage Index; mean (SD)** | 1.0158 (0.157) | 1.0050 (0.165) | 1.0150 (0.154) | 1.0067 (0,204) | 0.9874 (0.199) | 1.0044 (0.200) |

**Appendix B Table 3-CAUTI: Results of Median Regression Models 2011 and 2016**

| **Median Regression Estimates for Cost of CAUTI as a Principal Diagnosis (n=2230) 2011** | | | | | | | **Median Regression Estimates for Cost of CAUTI as a Principal Diagnosis (n=1162) 2016** | | | | | | |
| --- | --- | --- | --- | --- | --- | --- | --- | --- | --- | --- | --- | --- | --- |
| **Parameter** | **Estimate** | **Standard Error** | **95% Confidence Limits** | | **t Value** | **Pr > \|t\|** | **Parameter** | **Estimate** | **Standard Error** | **95% Confidence Limits** | | **t Value** | **Pr > \|t\|** |
| **Intercept** | -7468.86 | 577.2494 | -8600.868 | -6336.858 | -12.94 | <.0001 | **Intercept** | -6337.99 | 791.2709 | -7890.479 | -4785.491 | -8.01 | <.0001 |
| **LOS** | 1213.853 | 46.9643 | 1121.7546 | 1305.9517 | 25.85 | <.0001 | **LOS** | 1070.616 | 51.7633 | 969.0555 | 1172.1774 | 20.68 | <.0001 |
| **NDX** | 64.7421 | 15.7678 | 33.8209 | 95.6632 | 4.11 | <.0001 | **NDX** | 69.3141 | 18.4953 | 33.0259 | 105.6024 | 3.75 | 0.0002 |
| **NPR** | 716.913 | 80.1914 | 559.655 | 874.171 | 8.94 | <.0001 | **NPR** | 1141.597 | 90.1571 | 964.7070 | 1318.4878 | 12.66 | <.0001 |
| **URBAN_TEACH** | 393.8521 | 137.8902 | 123.4447 | 664.2594 | 2.86 | 0.0043 | **URBAN_TEACH** | 1128.312 | 189.8859 | 755.7512 | 1500.8731 | 5.94 | <.0001 |
| **URBAN_TEACH** | 0 | 0 | 0 | 0 | . | . | **URBAN_TEACH** | 0.0000 | 0.0000 | 0.0000 | 0.0000 | . | . |
| **SMALL_BEDSIZE** | -1412.6 | 182.7209 | -1770.925 | -1054.281 | -7.73 | <.0001 | **SMALL_BEDSIZE** | -658.207 | 201.4032 | -1053.365 | -263.0488 | -3.27 | 0.0011 |
| **SMALL_BEDSIZE** | 0 | 0 | 0 | 0 | . | . | **SMALL_BEDSIZE** | 0.0000 | 0.0000 | 0.0000 | 0.0000 | . | . |
| **AGE** | -18.469 | 4.3093 | -26.9198 | -10.0182 | -4.29 | <.0001 | **AGE** | -13.2967 | 7.0636 | -27.1557 | 0.5624 | -1.88 | 0.0600 |
| **APRDRG_Severity** | 259.0951 | 149.41 | -33.9029 | 552.0931 | 1.73 | 0.083 | **APRDRG_Severity** | 447.2756 | 243.6736 | -30.8182 | 925.3694 | 1.84 | 0.0667 |
| **APRDRG_Risk_Mortality** | 388.0329 | 130.4068 | 132.3009 | 643.7649 | 2.98 | 0.003 | **APRDRG_Risk_Mortality** | -124.155 | 201.8185 | -520.1285 | 271.8177 | -0.62 | 0.5386 |
| **Wage_Index** | 8277.895 | 464.6543 | 7366.693 | 9189.0978 | 17.82 | <.0001 | **Wage_Index** | 6295.842 | 487.9433 | 5338.4849 | 7253.1990 | 12.90 | <.0001 |

**Specified Stage 2 Cost Simulation Models for CAUTI 2011 and 2016**

**Cost_cauti_2011 = -7468.86 + 1213.853 *5.23901345 + 64.7421*NDX + 1141.597*NPR + 1128.312*URBAN_TEACH + (-658.207)*SMALL_BEDSIZE +**

**(-13.2967)*AGE + 447.2756*APRDRG_Severity + -124.155*APRDRG_Risk_Mortality + 6295.842*Wage_Index**

**Cost_cauti_2016 = -6337.99 + 1070.616*5.25859107 + 69.3141*NDX + 1141.597*NPR + 1120.037*URBAN_TEACH + (-660.799)*SMALL_BEDSIZE +**

**(-15.6026)*AGE + 432.338*APRDRG_Severity + (-98.9864)*APRDRG_Risk_Mortality + 6269.582*Wage_Index**

***Clostridium difficile* Infections (CDI)**

| **Appendix B Table-CDI: IDC-9 and ICD-10 Codes for *Clostridium difficile* Infections** | | | |
| --- | --- | --- | --- |
| **ICD-9 Description** | **icd9_cdi** | **ICD10_cdi** | **ICD-10 Description** |
| Intestinal infection due to *Clostridium difficile* | 00845 | A047 | Enterocolitis due to *Clostridium difficile* |

**Appendix B Table 1-CDI: Univariate Statistics 2011 and 2016**

| **Patient Characteristics** | **CDI - Group Comparisons** | | | **CDI - Group Comparisons 2016** | | |
| --- | --- | --- | --- | --- | --- | --- |
|  | **Cases with Secondary Dx, LOS > 2 days** | **Cases with Principal DX** | **Matched Cases with No Dx** | **Cases with Secondary Dx, LOS > 2 days** | **Cases with Principal DX** | **Matched Cases with No Dx** |
|  |  |  |  |  |  |  |
|  |  |  |  |  |  |  |
| **n =** | 45,608 | 23,829 | 228,040 | 45,591 | 20,983 | 227,955 |
| **Variables:** |  |  |  |  |  |  |
| **Cost** |  |  |  |  |  |  |
| **mean** | $37,454 | $11,378 | $15,804 | $33,358 | $9,442 | $15,423 |
| **median** | $20,400 | $7,719 | $9,217 | $17,357 | $6,789 | $9,139 |
| **mode** | $11,069 | $4,597 | $78 | $9,445 | $9,445 | $3,148 |
| **range** | $58 - $1,558,337 | $254 - $525,106 | $31 - $1,077,017 | $51 - $2,009,951 | $53 - $557,110 | $22 - $2,180,084 |
| **LOS** |  |  |  |  |  |  |
| **mean** | 14.95 | 6.39 | 6.32 | 13.12 | 5.52 | 6.00 |
| **median** | 10 | 5 | 4 | 9 | 4 | 4 |
| **mode** | 5 | 3 | 3 | 4 | 3 | 3 |
| **range** | 3 - 363 | 1 - 188 | 1 - 365 | 3 - 360 | 1 - 179 | 1 - 334 |
| **Number of Diagnoses; mean (SD)** | 17.11 (6.31) | 12.53 (5.83) | 12.10 (6.18) | 19.02 (6.12) | 13.95 (6.24) | 14.10 (6.72) |
| **Number of Procedures; mean (SD)** | 3.26 (3.76) | 0.84 (1.53) | 1.80 (2.46) | 2.91 (3.55) | 0.62 (1.36) | 1.71 (2.47) |
| **age; mean (range)** | 68 (0 - 105) | 68 (0 - 104) | 62 (0-111) | 65 (0 - 90) | 64 (0 - 90) | 61 (0-90) |
| **APRDRG_Severity; mean (SD)** | 3.52 (0.62) | 2.71 (0.81) | 2.65 (0.95) | 3.39 (0.64) | 2.44 (0.77) | 2.59 (0.92) |
| **APRDRG_Risk_Mortality; mean (SD)** | 2.99 (0.96) | 2.28 (0.98) | 2.30 (1.07) | 2.93 (0.95) | 2.10 (0.88) | 2.35 (1.06) |
| **Wage Index; mean (SD)** | 1.0239 (0.162) | 1.0034 (0.156) | 1.0005 (0.160) | 1.0128 (0.197) | 0.9920 (0.188) | 1.0024 (0.196) |

**Appendix B Table 3-CDI: Results of Median Regression Models 2011 and 2016**

| **Median Regression Estimates for Cost of CDI as a Principal Diagnosis (n= 23825) 2011** | | | | | | | **Median Regression Estimates for Cost of CDI as a Principal Diagnosis (n= 20975) 2016** | | | | | | |
| --- | --- | --- | --- | --- | --- | --- | --- | --- | --- | --- | --- | --- | --- |
| **Parameter** | **Estimate** | **Standard Error** | **95% Confidence Limits** | | **t Value** | **Pr > \|t\|** | **Parameter** | **Estimate** | **Standard Error** | **95% Confidence Limits** | | **t Value** | **Pr > \|t\|** |
| **Intercept** | -8412.8 | 238.5321 | -8880.343 | -7945.267 | -35.27 | <.0001 | **Intercept** | -4736.41 | 169.6228 | -5068.885 | -4403.938 | -27.92 | <.0001 |
| **LOS** | 1202.687 | 12.4281 | 1178.3275 | 1227.0471 | 96.77 | <.0001 | **LOS** | 1101.976 | 11.7509 | 1078.9433 | 1125.0085 | 93.78 | <.0001 |
| **NDX** | 57.4629 | 6.2605 | 45.1918 | 69.7339 | 9.18 | <.0001 | **NDX** | 42.4056 | 4.7864 | 33.0239 | 51.7872 | 8.86 | <.0001 |
| **NPR** | 1425.42 | 39.8703 | 1347.272 | 1503.5685 | 35.75 | <.0001 | **NPR** | 1077.977 | 40.8273 | 997.9518 | 1158.0013 | 26.4 | <.0001 |
| **URBAN_TEACH** | 624.4567 | 45.288 | 535.6893 | 713.2241 | 13.79 | <.0001 | **URBAN_TEACH** | 613.5722 | 47.9813 | 519.5251 | 707.6192 | 12.79 | <.0001 |
| **URBAN_TEACH** | 0 | 0 | 0 | 0 | 0 | . | **URBAN_TEACH** | 0 | 0 | 0 | 0 | . | . |
| **SMALL_BEDSIZE** | -501.746 | 67.1356 | -633.3363 | -370.1562 | -7.47 | <.0001 | **SMALL_BEDSIZE** | -541.004 | 51.2706 | -641.4979 | -440.5092 | -10.55 | <.0001 |
| **SMALL_BEDSIZE** | 0 | 0 | 0 | 0 | 0 | . | **SMALL_BEDSIZE** | 0 | 0 | 0 | 0 | . | . |
| **AGE** | -19.0363 | 1.2423 | -21.4713 | -16.6013 | -15.32 | <.0001 | **AGE** | -18.0531 | 1.338 | -20.6757 | -15.4304 | -13.49 | <.0001 |
| **APRDRG_Severity** | 210.6645 | 54.9526 | 102.9539 | 318.375 | 3.83 | 0.0001 | **APRDRG_Severity** | 244.2869 | 41.8892 | 162.1808 | 326.393 | 5.83 | <.0001 |
| **APRDRG_Risk_Mortality** | 213.8718 | 43.942 | 127.7426 | 300.0009 | 4.87 | <.0001 | **APRDRG_Risk_Mortality** | 193.5017 | 46.9157 | 101.5433 | 285.4602 | 4.12 | <.0001 |
| **Wage_Index** | 9359.705 | 197.0235 | 8973.5261 | 9745.8832 | 47.51 | <.0001 | **Wage_Index** | 6412.483 | 148.2891 | 6121.8246 | 6703.1408 | 43.24 | <.0001 |

**Specified Stage 2 Cost Simulation Models for CDI 2011 and 2016**

**Cost_cdi_2011 = -8412.8 + 1202.687*6.39430945 + 57.4629*NDX + 1425.42*NPR + 624.4567*URBAN_TEACH + (-501.746)*SMALL_BEDSIZE +**

**(-19.0363)*AGE + 210.6645*APRDRG_Severity + 213.8718*APRDRG_Risk_Mortality + 9359.705*Wage_Index**

**Cost_cdi_2016 = -4736.41 + 1101.976*5.5157985 + 42.4056*NDX + 1077.977*NPR + 613.5722*URBAN_TEACH + (-541.004)*SMALL_BEDSIZE +**

**(-18.0531)*AGE + 244.2869*APRDRG_Severity + 193.5017*APRDRG_Risk_Mortality + 6412.483*Wage_Index**

**Central Nervous System Infections (CNSI)**

| **Appendix B Table 1-CNSI: IDC-9 and ICD-10 Codes for Central Nervous System Infections** | | | |
| --- | --- | --- | --- |
| **ICD-9 Description** | **icd9_cnsi** | **ICD10_cnsi** | **ICD-10 Description** |
| meningococcal meningitis | 0360 | A390 | Meningococcal meningitis |
| meningococcal encephalitis | 0361 | A3981 | Meningococcal encephalitis |
| meningococcemia (technically in the blood, although source likely CNS) | 0362 | A392 | Acute meningococcemia |
| meningococcemia (technically in the blood, although source likely CNS) | 0362 | A393 | Chronic meningococcemia |
| meningococcemia (technically in the blood, although source likely CNS) | 0362 | A394 | Meningococcemia, unspecified |
| Waterhouse-friderichsen syndrom, meningococcal (adrenal, although with meningitis) | 0363 | A391 | Waterhouse-Friderichsen syndrome |
| Meningococcal carditis, unspecified (technically heart, again with meningitis) | 03640 | A3950 | Meningococcal carditis, unspecified |
| Meningococal pericarditis | 03641 | A3953 | Meningococcal pericarditis |
| Meningococcal endocarditis | 03642 | A3951 | Meningococcal endocarditis |
| Meningococcal myocarditis | 03643 | A3952 | Meningococcal myocarditis |
| Meningococal optic neuritis (technically eye, with mengingitis) | 03681 | A3982 | Meningococcal retrobulbar neuritis |
| Meningococal arthorpathy (joint with meningitis) | 03682 | A3983 | Meningococcal arthritis |
| Meningococal arthorpathy (joint with meningitis) | 03682 | A3984 | Postmeningococcal arthritis |
| Other specified meningococal infections | 03689 | A3989 | Other meningococcal infections |
| Meningococcal infection, unspecified | 0369 | A399 | Meningococcal infection, unspecified |
| Unspecified slow virus infection of central nervous system | 0469 | A819 | Atypical virus infection of central nervous system, unspecified |
| Other specified viral meningitis | 0478 | A870 | Enteroviral meningitis |
| Other specified viral meningitis | 0478 | A878 | Other viral meningitis |
| Unspecified viral meningitis | 0479 | A879 | Viral meningitis, unspecified |
| Unspecified viral meningitis | 0479 | G032 | Benign recurrent meningitis [Mollaret] |
| Other enterovirus disease of central nervous system | 048 | A880 | Enteroviral exanthematous fever [Boston exanthem] |
| Other enterovirus disease of central nervous system | 048 | A888 | Other specified viral infections of central nervous system |
| Lymphocytic choriomeningitis | 0490 | A872 | Lymphocytic choriomeningitis |
| Meningitis due to adenovirus | 0491 | A871 | Adenoviral meningitis |
| Other human herpesvirus encephalitis | 05829 | B1009 | Other human herpesvirus encephalitis |
| candidal meningitis | 11283 | B375 | Candidal meningitis |
| hemophilus meningitis | 3200 | G000 | Hemophilus meningitis |
| **Appendix B Table 1-CNSI: IDC-9 and ICD-10 Codes for Central Nervous System Infections cont.** | | | |
| **ICD-9 Description** | **icd9_cnsi** | **ICD10_cnsi** | **ICD-10 Description** |
| Pneumococcal meningitis | 3201 | G001 | Pneumococcal meningitis |
| Streptococcal meningitis | 3202 | G002 | Streptococcal meningitis |
| Staphylcoccal meningitis | 3203 | G003 | Staphylococcal meningitis |
| meningitis in other bacterial diseases classified elsewhere | 3207 | G01 | Meningitis in bacterial diseases classified elsewhere |
| anaerobic meningitis | 32081 | G008 | Other bacterial meningitis |
| meningitis due to gram-negative bacteria, not classified elsewhere | 32082 | G009 | Bacterial meningitis, unspecified |
| meningitis due to other specified bacteria | 32089 | G008 | Other bacterial meningitis |
| Meningitis due to unspecified bacterium | 3209 | G009 | Bacterial meningitis, unspecified |
| Meningitis due to unspecified bacterium | 3209 | G042 | Bacterial meningoencephalitis and meningomyelitis, not elsewhere classified |
| meningitis due to fungal diseases | 3211 | G02 | Meningitis in other infectious and parasitic diseases classified elsewhere |
| meningitis due to viruses not elsewhere classified | 3212 | G02 | Meningitis in other infectious and parasitic diseases classified elsewhere |
| meningitis due to other nonbacterial organisms classified elsewhere | 3218 | G02 | Meningitis in other infectious and parasitic diseases classified elsewhere |
| meningitis due to other nonbacterial organisms classified elsewhere | 3218 | G030 | Nonpyogenic meningitis |
| nonpyogenic meningitis | 3220 | G030 | Nonpyogenic meningitis |
| meningits, unspecified (includes ventriculitis and many others) | 3229 | G039 | Meningitis, unspecified |
| Encephalitis and encephalomyelitis in viral diseases classified elsewhere | 32301 | G053 | Encephalitis and encephalomyelitis in diseases classified elsewhere |
| Myelitis in viral diseases classified elsewhere | 32302 | G054 | Myelitis in diseases classified elsewhere |
| Other encephalitis and encephalomyelitis due to toher infections classified elsewhere | 32341 | G053 | Encephalitis and encephalomyelitis in diseases classified elsewhere |
| Other myleitis due to other infections classified elsewhere | 32342 | G054 | Myelitis in diseases classified elsewhere |
| Encephalitis and encephalomyelitis following immunization procedures | 32351 | G0401 | Postinfectious acute disseminated encephalitis and encephalomyelitis (postinfectious ADEM) |
| Encephalitis and encephalomyelitis following immunization procedures | 32351 | G0402 | Postimmunization acute disseminated encephalitis, myelitis and encephalomyelitis |
| Encephalitis and encephalomyelitis following immunization procedures | 32351 | G0432 | Postimmunization acute necrotizing hemorrhagic encephalopathy |
| Other postinfectious encephalitis and encephalomyelitis | 32362 | G0430 | Acute necrotizing hemorrhagic encephalopathy, unspecified |
| Other postinfectious encephalitis and encephalomyelitis | 32362 | G0431 | Postinfectious acute necrotizing hemorrhagic encephalopathy |
| other causes of encephalitis and encephalomyelitis | 32381 | G0481 | Other encephalitis and encephalomyelitis |
| other causes of myelitis | 32382 | G0489 | Other myelitis |
| unspecified causes of encephalitis, myelitis, and encephalomyelitis | 3239 | G0490 | Encephalitis and encephalomyelitis, unspecified |
| **Appendix B Table 1-CNSI: IDC-9 and ICD-10 Codes for Central Nervous System Infections cont.** | | | |
| **ICD-9 Description** | **icd9_cnsi** | **ICD10_cnsi** | **ICD-10 Description** |
| unspecified causes of encephalitis, myelitis, and encephalomyelitis | 3239 | G0491 | Myelitis, unspecified |
| unspecified causes of encephalitis, myelitis, and encephalomyelitis | 3239 | G374 | Subacute necrotizing myelitis of central nervous system |
| intracranial abscess | 3240 | G060 | Intracranial abscess and granuloma |
| intraspinal abscess | 3241 | G061 | Intraspinal abscess and granuloma |
| intracranial and intraspinal abscess of unspecified site | 3249 | G062 | Extradural and subdural abscess, unspecified |
| intracranial and intraspinal abscess of unspecified site | 3249 | G07 | Intracranial and intraspinal abscess and granuloma in diseases classified elsewhere |
| phlebitis and thrombophlebitis of intracranial venous sinuses | 325 | G08 | Intracranial and intraspinal phlebitis and thrombophlebitis |
| Late effects of intracranial abscess or pyogenic infection | 326 | G09 | Sequelae of inflammatory diseases of central nervous system |
| Acute infective polyneuritis | 3570 | n/a |  |
| Infection and inflammatory reaction due to nervous system device, implant, and graft | 99663 | T85730A | Infection and inflammatory reaction due to ventricular intracranial (communicating) shunt, initial encounter |
| Infection and inflammatory reaction due to nervous system device, implant, and graft | 99663 | T85731A | Infection and inflammatory reaction due to implanted electronic neurostimulator of brain, electrode (lead), initial encounter |
| Infection and inflammatory reaction due to nervous system device, implant, and graft | 99663 | T85732A | Infection and inflammatory reaction due to implanted electronic neurostimulator of peripheral nerve, electrode (lead), initial encounter |
| Infection and inflammatory reaction due to nervous system device, implant, and graft | 99663 | T85733A | Infection and inflammatory reaction due to implanted electronic neurostimulator of spinal cord, electrode (lead), initial encounter |
| Infection and inflammatory reaction due to nervous system device, implant, and graft | 99663 | T85734A | Infection and inflammatory reaction due to implanted electronic neurostimulator, generator, initial encounter |
| Infection and inflammatory reaction due to nervous system device, implant, and graft | 99663 | T85735A | Infection and inflammatory reaction due to cranial or spinal infusion catheter, initial encounter |
| Infection and inflammatory reaction due to nervous system device, implant, and graft | 99663 | T85738A | Infection and inflammatory reaction due to other nervous system device, implant or graft, initial encounter |
| Other complications due to nervous system device, implant, and graft | 99675 | T85810A | Embolism due to nervous system prosthetic devices, implants and grafts, initial encounter |
| Other complications due to nervous system device, implant, and graft | 99675 | T85820A | Fibrosis due to nervous system prosthetic devices, implants and grafts, initial encounter |
| Other complications due to nervous system device, implant, and graft | 99675 | T85830A | Hemorrhage due to nervous system prosthetic devices, implants and grafts, initial encounter |
| Other complications due to nervous system device, implant, and graft | 99675 | T85840A | Pain due to nervous system prosthetic devices, implants and grafts, initial encounter |
| Other complications due to nervous system device, implant, and graft | 99675 | T85850A | Stenosis due to nervous system prosthetic devices, implants and grafts, initial encounter |
| **Appendix B Table 1-CNSI: IDC-9 and ICD-10 Codes for Central Nervous System Infections cont.** | | | |
| **ICD-9 Description** | **icd9_cnsi** | **ICD10_cnsi** | **ICD-10 Description** |
| Other complications due to nervous system device, implant, and graft | 99675 | T85860A | Thrombosis due to nervous system prosthetic devices, implants and grafts, initial encounter |
| Other complications due to nervous system device, implant, and graft | 99675 | T85890A | Other specified complication of nervous system prosthetic devices, implants and grafts, initial encounter |

**Appendix B Table 2-CNSI: Univariate Statistics 2011 and 2016**

| **Patient Characteristics** | **CNSI - Group Comparisons 2011** | | | **CNSI - Group Comparisons 2016** | | |
| --- | --- | --- | --- | --- | --- | --- |
|  | **Cases with Secondary Dx, LOS > 2 days** | **Cases with Principal DX** | **Matched Cases with No Dx** | **Cases with Secondary Dx, LOS > 2 days** | **Cases with Principal DX** | **Matched Cases with No Dx** |
|  |  |  |  |  |  |  |
|  |  |  |  |  |  |  |
| **n =** | 10,790 | 12,877 | 53,950 | 9,759 | 8,339 | 48,795 |
| **Variables:** |  |  |  |  |  |  |
| **Cost** |  |  |  |  |  |  |
| **mean** | $44,180 | $21,285 | $15,927 | $48,930 | $20,256 | $16,737 |
| **median** | $22,738 | $10,346 | $9,173 | $25,876 | $10,248 | $9,733 |
| **mode** | $7,742 | $2,426 | $78 | $13,062 | $3,148 | $3,148 |
| **range** | $1,177 - $1,403,145 | $344 - $1,250,769 | $31 - $1,258,043 | $71 - $1,957,156 | $413 - $1,190,381 | $39 - $1,336,654 |
| **LOS** |  |  |  |  |  |  |
| **mean** | 15.92 | 7.49 | 6.76 | 16.78 | 7.58 | 6.44 |
| **median** | 10 | 4 | 4 | 10 | 4 | 4 |
| **mode** | 3 | 2 | 2 | 5 | 2 | 2 |
| **range** | 3 - 360 | 1 - 310 | 1 - 365 | 3 - 355 | 1 - 268 | 1 -326 |
| **Number of Diagnoses; mean (SD)** | 14.46 (6.61) | 8.23 (6.12) | 11.20 (6.29) | 17.14 (6.84) | 10.07 (6.91) | 13.52 (7.03) |
| **Number of Procedures; mean (SD)** | 3.86 (4.06) | 2.23 (2.60) | 1.78 (2.39) | 4.17 (3.85) | 2.10 (2.46) | 1.83 (2.49) |
| **age; mean (range)** | 50 (0 - 98) | 40 (0 -101) | 57 (0-121) | 49 (0 - 90) | 40 (0 -90) | 57 (0-90) |
| **APRDRG_Severity; mean (SD)** | 3.32 (0.71) | 2.23 (0.97) | 2.51 (0.97) | 3.39 (0.69) | 2.23 (0.98) | 2.55 (0.96) |
| **APRDRG_Risk_Mortality; mean (SD)** | 2.76 (1.00) | 1.67 (1.00) | 2.10 (1.07) | 2.98 (0.94) | 1.78 (1.01) | 2.25 (1.10) |
|  | 1.0204 (0.159) | 1.0068 (0.152) | 1.0067 (0.161) | 1.0203 (0.195) | 1.0086 (0.193) | 1.0085 (0.197) |

**Appendix B Table 3-CNSI: Results of Median Regression Models 2011 and 2016**

| **Median Regression Estimates for Cost of CNSI as a Principal Diagnosis (n=12855) 2011** | | | | | | | **Median Regression Estimates for Cost of CNSI as a Principal Diagnosis (n=8338) 2016** | | | | | | |
| --- | --- | --- | --- | --- | --- | --- | --- | --- | --- | --- | --- | --- | --- |
| **Parameter** | **Estimate** | **Standard Error** | **95% Confidence Limits** | | **t Value** | **Pr > \|t\|** | **Parameter** | **Estimate** | **Standard Error** | **95% Confidence Limits** | | **t Value** | **Pr > \|t\|** |
| **Intercept** | -11349.1 | 395.6146 | -12124.61 | -10573.68 | -28.69 | <.0001 | **Intercept** | -10323.6 | 451.1375 | -11207.93 | -9439.246 | -22.88 | <.0001 |
| **LOS** | 1856.319 | 35.1502 | 1787.4197 | 1925.2188 | 52.81 | <.0001 | **LOS** | 1597.515 | 36.9814 | 1525.0219 | 1670.0073 | 43.20 | <.0001 |
| **NDX** | 29.6811 | 20.0386 | -9.5975 | 68.9597 | 1.48 | 0.1386 | **NDX** | 31.0207 | 21.0841 | -10.3095 | 72.3508 | 1.47 | 0.1413 |
| **NPR** | 2227.191 | 78.935 | 2072.4671 | 2381.9157 | 28.22 | <.0001 | **NPR** | 2240.332 | 81.2868 | 2080.9897 | 2399.6746 | 27.56 | <.0001 |
| **URBAN_TEACH** | 88.1918 | 93.2704 | -94.632 | 271.0156 | 0.95 | 0.3444 | **URBAN_TEACH** | -40.3790 | 138.3218 | -311.5241 | 230.7661 | -0.29 | 0.7704 |
| **URBAN_TEACH** | 0 | 0 | 0 | 0 | . | . | **URBAN_TEACH** | 0.0000 | 0.0000 | 0.0000 | 0.0000 | . | . |
| **SMALL_BEDSIZE** | -649.164 | 147.4262 | -938.1409 | -360.1864 | -4.4 | <.0001 | **SMALL_BEDSIZE** | -634.482 | 147.1508 | -922.9341 | -346.0299 | -4.31 | <.0001 |
| **SMALL_BEDSIZE** | 0 | 0 | 0 | 0 | . | . | **SMALL_BEDSIZE** | 0.0000 | 0.0000 | 0.0000 | 0.0000 | . | . |
| **AGE** | 11.7064 | 2.743 | 6.3296 | 17.0832 | 4.27 | <.0001 | **AGE** | -17.0603 | 3.5511 | -24.0213 | -10.0993 | -4.80 | <.0001 |
| **APRDRG_Severity** | 965.1056 | 113.3068 | 743.0075 | 1187.2038 | 8.52 | <.0001 | **APRDRG_Severity** | 811.3727 | 135.0265 | 546.6872 | 1076.0582 | 6.01 | <.0001 |
| **APRDRG_Risk_Mortality** | -32.9493 | 114.3408 | -257.0742 | 191.1756 | -0.29 | 0.7732 | **APRDRG_Risk_Mortality** | 949.5807 | 149.3892 | 656.7406 | 1242.4208 | 6.36 | <.0001 |
| **Wage_Index** | 8675.544 | 396.1837 | 7898.9653 | 9452.1233 | 21.9 | <.0001 | **Wage_Index** | 8437.237 | 422.8439 | 7608.3575 | 9266.1163 | 19.95 | <.0001 |

**Specified Stage 2 Cost Simulation Models for CNSI 2011 and 2016**

**Cost_cnsi_2011 = -11349.1 + 1856.319*7.49312728 + 29.6811*NDX + 2227.191*NPR + 88.1918*URBAN_TEACH + (-649.164)*SMALL_BEDSIZE +**

**11.7064*AGE + 965.1056*APRDRG_Severity + (-32.9493)*APRDRG_Risk_Mortality + 8675.544*Wage_Index**

**Cost_cnsi_2016 = Intercept = -10323.6+ 1597.515* 7.58472239 + (31.0207)*NDX + 2240.332*NPR + (-40.3790 )*URBAN_TEACH +**

**(-634.482)*SMALL_BEDSIZE + (-17.0603)*AGE + 811.3727*APRDRG_Severity + 949.5807*APRDRG_Risk_Mortality +**

**8437.237*Wage_Index**

**Cardiovascular System Infections (CSI)**

| **Appendix B Table 1-CSI: IDC-9 and ICD-10 Codes for Cardiovascular System Infections** | | | |  |  |  |
| --- | --- | --- | --- | --- | --- | --- |
| **ICD-9 Description** | **icd9_csi** | | **ICD10_csi** | **ICD-10 Description** |  |  |
| candidial endocarditis | 11281 | | B376 | candidial endocarditis |  |  |
| acute pericarditis in diseases classified elsewhere | 4200 | | I32 | Pericarditis in diseases classified elsewhere |  |  |
| acute pericarditis, unspecified | 42090 | | I301 | Infective pericarditis |  |  |
| other acute pericarditis | 42099 | | I309 | Acute pericarditis, unspecified |  |  |
| acute and subacute bacterial endocarditis (includes mycotic aneurysm) | 4210 | | I330 | Acute and subacute infective endocarditis |  |  |
| acute and subacute infective endocarditis in diseases classified elsewhere | 4211 | | I39 | Endocarditis and heart valve disorders in diseases classified elsewhere |  |  |
| Acute endocarditis, unspecified | 4219 | | I339 | Acute and subacute endocarditis, unspecified |  |  |
| acute myocarditis in diseases classified elsewhere | 4220 | | I41 | Myocarditis in diseases classified elsewhere |  |  |
| acute myocarditis, unspecified | 42290 | | I409 | Acute myocarditis, unspecified |  |  |
| septic myocarditis | 42292 | | I400 | Infective myocarditis |  |  |
| other acute myocarditis | 42299 | | I408 | Other acute myocarditis |  |  |
| Endocarditis, valve unspecified, unspecified cause | 42490 | I38 | | Endocarditis, valve unspecified, unspecified cause |  | |
| Endocarditis in diseases classified elsewhere | 42491 | | I39 | Endocarditis and heart valve disorders in diseases classified elsewhere |  |  |
| Other endocarditis, valve unspecified | 42499 | | I38 | Endocarditis, valve unspecified |  |  |
| Myocarditis, unspecified | 4290 | | I514 | Myocarditis, unspecified |  |  |
| Phlebitis and thrombophlebitis | 45189 | | I808 | Phlebitis and thrombophlebitis of other sites |  |  |
| Phlebitis and thrombophlebitis of unspecified site | 4519 | | I809 | Phlebitis and thrombophlebitis of unspecified site |  |  |
|  |  | |  |  |  |  |
|  |  | |  |  |  |  |
|  |  | |  |  |  |  |
|  |  | |  |  |  |  |

**Appendix B Table 2-CSI: Univariate Statistics 2011 and 2016**

| **Patient Characteristics** | **CSI - Group Comparisons 2011** | | | **CSI - Group Comparisons 2016** | | |
| --- | --- | --- | --- | --- | --- | --- |
|  | **Cases with Secondary Dx** | **Cases with Principal DX** | **Matched Cases with No Dx** | **Cases with Secondary Dx** | **Cases with Principal DX** | **Matched Cases with No Dx** |
|  |  |  |  |  |  |  |
|  |  |  |  |  |  |  |
| **n =** | 9,125 | 5,015 | 45,625 | 22,336 | 5,654 | 111,680 |
| **Variables:** |  |  |  |  |  |  |
| **Cost** |  |  |  |  |  |  |
| **mean** | $43,249 | $26,555 | $18,144 | $34,174 | $22,195 | $16,131 |
| **median** | $25,213 | $12,807 | $10,563 | $19,064 | $10,206 | $9,526 |
| **mode** | $4,949 | $3,461 | $78 | $9,445 | $4,883 | $3,148 |
| **range** | $68 - $1,452,246 | $417 - $656,113 | $31 - $1,756,572 | $71 - $2,371,529 | $187 - $931,326 | $22 - $1,910,435 |
| **LOS** |  |  |  |  |  |  |
| **mean** | 14.57 | 9.00 | 6.44 | 12.43 | 7.92 | 5.86 |
| **median** | 10 | 5 | 4 | 8 | 4 | 4 |
| **mode** | 6 | 1 | 3 | 3 | 1 | 2 |
| **range** | 3 - 355 | 1 - 158 | 1 - 308 | 3 - 355 | 1 - 214 | 1 - 340 |
| **Number of Diagnoses; mean (SD)** | 16.64 (6.50) | 12.10 (6.77) | 12.32 (6.26) | 18.66 (6.29) | 12.90 (7.13) | 13.94 (6.71) |
| **Number of Procedures; mean (SD)** | 4.45 (4.16) | 2.82 (3.33) | 2.22 (2.76) | 3.46 (3.68) | 2.31 (3.06) | 1.86 (2.54) |
| **age; mean (range)** | 58 (0 - 103) | 54 (0 - 98) | 62 (0-108) | 60 (0 - 90) | 49 (0 - 90) | 62 (0-90) |
| **APRDRG_Severity; mean (SD)** | 3.50 (0.70) | 2.74 (1.03) | 2.67 (0.97) | 3.24 (0.76) | 2.56 (1.03) | 2.55 (0.93) |
| **APRDRG_Risk_Mortality; mean (SD)** | 3.14 (0.91) | 2.35 (1.12) | 2.32 (1.09) | 2.93 (0.98) | 2.21 (1.11) | 2.30 (1.06) |
|  | 1.0174 (0.150) | 1.0221 (0.165) | 1.0024 (0.158) | 1.0038 (0.191) | 1.0218 (0.201) | 1.0027 (0.196) |

**Appendix B Table 3-CSI: Results of Median Regression Models 2011 and 2016**

| **Median Regression Estimates for Cost of CSI as a Principal Diagnosis (n= 5012) 2011** | | | | | | | **Median Regression Estimates for Cost of CSI as a Principal Diagnosis (n= 5653) 2016** | | | | | | |
| --- | --- | --- | --- | --- | --- | --- | --- | --- | --- | --- | --- | --- | --- |
| **Parameter** | **Estimate** | **Standard Error** | **95% Confidence Limits** | | **t Value** | **Pr > \|t\|** | **Parameter** | **Estimate** | **Standard Error** | **95% Confidence Limits** | | **t Value** | **Pr > \|t\|** |
| **Intercept** | -9875.05 | 881.0686 | -11602.33 | -8147.767 | -11.21 | <.0001 | **Intercept** | -6782.42 | 443.0136 | -7650.898 | -5913.944 | -15.31 | <.0001 |
| **LOS** | 1605.839 | 41.6725 | 1524.1429 | 1687.5358 | 38.53 | <.0001 | **LOS** | 1562.522 | 49.7109 | 1465.0699 | 1659.975 | 31.43 | <.0001 |
| **NDX** | 24.841 | 27.8475 | -29.7523 | 79.4343 | 0.89 | 0.3724 | **NDX** | 25.3155 | 21.7013 | -17.2275 | 67.8584 | 1.17 | 0.2434 |
| **NPR** | 2679.449 | 93.3593 | 2496.424 | 2862.4741 | 28.7 | <.0001 | **NPR** | 2438.37 | 95.7893 | 2250.5857 | 2626.1534 | 25.46 | <.0001 |
| **URBAN_TEACH** | 695.7214 | 215.1823 | 273.8698 | 1117.5729 | 3.23 | 0.0012 | **URBAN_TEACH** | -75.2345 | 161.8814 | -392.5843 | 242.1152 | -0.46 | 0.6421 |
| **URBAN_TEACH** | 0 | 0 | 0 | 0 | . | . | **URBAN_TEACH** | 0 | 0 | 0 | 0 | . | . |
| **SMALL_BEDSIZE** | -1009.98 | 336.1404 | -1668.959 | -350.9941 | -3 | 0.0027 | **SMALL_BEDSIZE** | -267.062 | 172.92 | -606.052 | 71.9273 | -1.54 | 0.1225 |
| **SMALL_BEDSIZE** | 0 | 0 | 0 | 0 | . | . | **SMALL_BEDSIZE** | 0 | 0 | 0 | 0 | . | . |
| **AGE** | -40.1626 | 7.1528 | -54.1852 | -26.1401 | -5.61 | <.0001 | **AGE** | -22.8534 | 4.8414 | -32.3444 | -13.3624 | -4.72 | <.0001 |
| **APRDRG_Severity** | -428.236 | 221.6577 | -862.7818 | 6.3107 | -1.93 | 0.0534 | **APRDRG_Severity** | 11.6151 | 145.7617 | -274.1338 | 297.3641 | 0.08 | 0.9365 |
| **APRDRG_Risk_Mortality** | 881.292 | 215.1791 | 459.4466 | 1303.1373 | 4.1 | <.0001 | **APRDRG_Risk_Mortality** | 573.6926 | 137.8632 | 303.4278 | 843.9575 | 4.16 | <.0001 |
| **Wage_Index** | 11568.16 | 703.2219 | 10189.535 | 12946.781 | 16.45 | <.0001 | **Wage_Index** | 7614.379 | 305.5018 | 7015.478 | 8213.2801 | 24.92 | <.0001 |

**Specified Stage 2 Cost Simulation Models for CSI 2011 and 2016**

**Cost_csi_2011 = -9875.05 + 1605.839*9.00418744 + 24.841*NDX + 2679.449*NPR + 695.7214*URBAN_TEACH + (-1009.98)*SMALL_BEDSIZE +**

**(-40.1626)*AGE + (-428.236)*APRDRG_Severity + 881.292*APRDRG_Risk_Mortality + 11568.16*Wage_Index**

**Cost_csi_2016 = -6782.42 + 1562.522*7.91934913 + 25.3155*NDX + 2438.37*NPR + (-75.2345)*URBAN_TEACH + (-267.062)*SMALL_BEDSIZE +**

**(-22.8534)*AGE + 11.6151*APRDRG_Severity + 573.6926*APRDRG_Risk_Mortality + 7614.379*Wage_Index**

**Eye, Ear, Nose, Throat, and Mouth Infections (EENTM)**

| **Appendix B Table 1-EENTM: IDC-9 and ICD-10 Codes for Eye, Ear, Nose, Throat, and Mouth Infections** | | | |
| --- | --- | --- | --- |
| **ICD-9 Description** | **icd9_eentm** | **icd10_eentm** | **ICD-10 Description** |
| Streptococcal sore throat | 0340 | J020 | Streptococcal pharyngitis |
| Streptococcal sore throat | 0340 | J0300 | Acute streptococcal tonsillitis, unspecified |
| Streptococcal sore throat | 0340 | J0301 | Acute recurrent streptococcal tonsillitis |
| candidal otitis externa | 11282 | B3784 | Candidal otitis externa |
| purulent endopthalmitis, unspecified | 36000 | H44001 | Unspecified purulent endophthalmitis, right eye |
| purulent endopthalmitis, unspecified | 36000 | H44002 | Unspecified purulent endophthalmitis, left eye |
| purulent endopthalmitis, unspecified | 36000 | H44003 | Unspecified purulent endophthalmitis, bilateral |
| purulent endopthalmitis, unspecified | 36000 | H44009 | Unspecified purulent endophthalmitis, unspecified eye |
| acute endopthalmitis | 36001 | H44001 | Unspecified purulent endophthalmitis, right eye |
| acute endopthalmitis | 36001 | H44002 | Unspecified purulent endophthalmitis, left eye |
| acute endopthalmitis | 36001 | H44003 | Unspecified purulent endophthalmitis, bilateral |
| acute endopthalmitis | 36001 | H44009 | Unspecified purulent endophthalmitis, unspecified eye |
| panopthalmitis | 36002 | H44011 | Panophthalmitis (acute), right eye |
| panopthalmitis | 36002 | H44012 | Panophthalmitis (acute), left eye |
| panopthalmitis | 36002 | H44013 | Panophthalmitis (acute), bilateral |
| panopthalmitis | 36002 | H44019 | Panophthalmitis (acute), unspecified eye |
| vitreous abscess | 36004 | H44021 | Vitreous abscess (chronic), right eye |
| vitreous abscess | 36004 | H44022 | Vitreous abscess (chronic), left eye |
| vitreous abscess | 36004 | H44023 | Vitreous abscess (chronic), bilateral |
| vitreous abscess | 36004 | H44029 | Vitreous abscess (chronic), unspecified eye |
| panuveitis | 36012 | H44111 | Panuveitis, right eye |
| panuveitis | 36012 | H44112 | Panuveitis, left eye |
| panuveitis | 36012 | H44113 | Panuveitis, bilateral |
| panuveitis | 36012 | H44119 | Panuveitis, unspecified eye |
| other endopthalmitis | 36019 | H4419 | Other endophthalmitis |
| superficial keratitis | 37020 | H16101 | Unspecified superficial keratitis, right eye |
| superficial keratitis | 37020 | H16102 | Unspecified superficial keratitis, left eye |
| **Appendix B Table 1-EENTM: IDC-9 and ICD-10 Codes for Eye, Ear, Nose, Throat, and Mouth Infections cont.** | | | |
| **ICD-9 Description** | **icd9_eentm** | **icd10_eentm** | **ICD-10 Description** |
| superficial keratitis | 37020 | H16103 | Unspecified superficial keratitis, bilateral |
| superficial keratitis | 37020 | H16109 | Unspecified superficial keratitis, unspecified eye |
| punctate keratitis | 37021 | H16141 | Punctate keratitis, right eye |
| punctate keratitis | 37021 | H16142 | Punctate keratitis, left eye |
| punctate keratitis | 37021 | H16143 | Punctate keratitis, bilateral |
| punctate keratitis | 37021 | H16149 | Punctate keratitis, unspecified eye |
| Phlyctenular keratoconjunctivitis | 37031 | A1852 | Tuberculous keratitis |
| Phlyctenular keratoconjunctivitis | 37031 | H16251 | Phlyctenular keratoconjunctivitis, right eye |
| Phlyctenular keratoconjunctivitis | 37031 | H16252 | Phlyctenular keratoconjunctivitis, left eye |
| Phlyctenular keratoconjunctivitis | 37031 | H16253 | Phlyctenular keratoconjunctivitis, bilateral |
| Phlyctenular keratoconjunctivitis | 37031 | H16259 | Phlyctenular keratoconjunctivitis, unspecified eye |
| keratoconjunctivitis, unspecified | 37040 | H16201 | Unspecified keratoconjunctivitis, right eye |
| keratoconjunctivitis, unspecified | 37040 | H16202 | Unspecified keratoconjunctivitis, left eye |
| keratoconjunctivitis, unspecified | 37040 | H16203 | Unspecified keratoconjunctivitis, bilateral |
| keratoconjunctivitis, unspecified | 37040 | H16209 | Unspecified keratoconjunctivitis, unspecified eye |
| keratitis or keratoconjunctivitis in exanthema | 37044 | B09 | Unspecified viral infection characterized by skin and mucous membrane lesions |
| keratitis or keratoconjunctivitis in exanthema | 37044 | H16291 | Other keratoconjunctivitis, right eye |
| keratitis or keratoconjunctivitis in exanthema | 37044 | H16292 | Other keratoconjunctivitis, left eye |
| keratitis or keratoconjunctivitis in exanthema | 37044 | H16293 | Other keratoconjunctivitis, bilateral |
| keratitis or keratoconjunctivitis in exanthema | 37044 | H16299 | Other keratoconjunctivitis, unspecified eye |
| Other keratoconjunctivitis | 37049 | H16291 | Other keratoconjunctivitis, right eye |
| Other keratoconjunctivitis | 37049 | H16292 | Other keratoconjunctivitis, left eye |
| Other keratoconjunctivitis | 37049 | H16293 | Other keratoconjunctivitis, bilateral |
| Other keratoconjunctivitis | 37049 | H16299 | Other keratoconjunctivitis, unspecified eye |
| Interstitial keratitis, unspecified | 37050 | H16301 | Unspecified interstitial keratitis, right eye |
| Interstitial keratitis, unspecified | 37050 | H16302 | Unspecified interstitial keratitis, left eye |
| Interstitial keratitis, unspecified | 37050 | H16303 | Unspecified interstitial keratitis, bilateral |
| Interstitial keratitis, unspecified | 37050 | H16309 | Unspecified interstitial keratitis, unspecified eye |
| diffuse Interstitial keratitis | 37052 | H16321 | Diffuse interstitial keratitis, right eye |
| diffuse Interstitial keratitis | 37052 | H16322 | Diffuse interstitial keratitis, left eye |
| **Appendix B Table 1-EENTM: IDC-9 and ICD-10 Codes for Eye, Ear, Nose, Throat, and Mouth Infections cont.** | | | |
| **ICD-9 Description** | **icd9_eentm** | **icd10_eentm** | **ICD-10 Description** |
| diffuse Interstitial keratitis | 37052 | H16323 | Diffuse interstitial keratitis, bilateral |
| diffuse Interstitial keratitis | 37052 | H16329 | Diffuse interstitial keratitis, unspecified eye |
| sclerosing keratitis | 37054 | H16331 | Sclerosing keratitis, right eye |
| sclerosing keratitis | 37054 | H16332 | Sclerosing keratitis, left eye |
| sclerosing keratitis | 37054 | H16333 | Sclerosing keratitis, bilateral |
| sclerosing keratitis | 37054 | H16339 | Sclerosing keratitis, unspecified eye |
| corneal abscess | 37055 | H16311 | Corneal abscess, right eye |
| corneal abscess | 37055 | H16312 | Corneal abscess, left eye |
| corneal abscess | 37055 | H16313 | Corneal abscess, bilateral |
| corneal abscess | 37055 | H16319 | Corneal abscess, unspecified eye |
| other interstitial and deep keratitis | 37059 | A1852 | Tuberculous keratitis |
| other interstitial and deep keratitis | 37059 | H16391 | Other interstitial and deep keratitis, right eye |
| other interstitial and deep keratitis | 37059 | H16392 | Other interstitial and deep keratitis, left eye |
| other interstitial and deep keratitis | 37059 | H16393 | Other interstitial and deep keratitis, bilateral |
| other interstitial and deep keratitis | 37059 | H16399 | Other interstitial and deep keratitis, unspecified eye |
| acute conjunctivitis | 37200 | H1030 | Unspecified acute conjunctivitis, unspecified eye |
| acute conjunctivitis | 37200 | H1031 | Unspecified acute conjunctivitis, right eye |
| acute conjunctivitis | 37200 | H1032 | Unspecified acute conjunctivitis, left eye |
| acute conjunctivitis | 37200 | H1033 | Unspecified acute conjunctivitis, bilateral |
| serous conjunctivitis | 37201 | H10231 | Serous conjunctivitis, except viral, right eye |
| serous conjunctivitis | 37201 | H10232 | Serous conjunctivitis, except viral, left eye |
| serous conjunctivitis | 37201 | H10233 | Serous conjunctivitis, except viral, bilateral |
| serous conjunctivitis | 37201 | H10239 | Serous conjunctivitis, except viral, unspecified eye |
| acute follicular conjunctivitis | 37202 | H10011 | Acute follicular conjunctivitis, right eye |
| acute follicular conjunctivitis | 37202 | H10012 | Acute follicular conjunctivitis, left eye |
| acute follicular conjunctivitis | 37202 | H10013 | Acute follicular conjunctivitis, bilateral |
| acute follicular conjunctivitis | 37202 | H10019 | Acute follicular conjunctivitis, unspecified eye |
| other mucopurulent conjunctivitis | 37203 | H10021 | Other mucopurulent conjunctivitis, right eye |
| other mucopurulent conjunctivitis | 37203 | H10022 | Other mucopurulent conjunctivitis, left eye |
| other mucopurulent conjunctivitis | 37203 | H10023 | Other mucopurulent conjunctivitis, bilateral |
| **Appendix B Table 1-EENTM: IDC-9 and ICD-10 Codes for Eye, Ear, Nose, Throat, and Mouth Infections cont.** | | | |
| **ICD-9 Description** | **icd9_eentm** | **icd10_eentm** | **ICD-10 Description** |
| other mucopurulent conjunctivitis | 37203 | H10029 | Other mucopurulent conjunctivitis, unspecified eye |
| pseudomembranous conjunctivitis | 37204 | H10221 | Pseudomembranous conjunctivitis, right eye |
| pseudomembranous conjunctivitis | 37204 | H10222 | Pseudomembranous conjunctivitis, left eye |
| pseudomembranous conjunctivitis | 37204 | H10223 | Pseudomembranous conjunctivitis, bilateral |
| pseudomembranous conjunctivitis | 37204 | H10229 | Pseudomembranous conjunctivitis, unspecified eye |
| conjunctivitis, unspecified | 37230 | H109 | Unspecified conjunctivitis |
| conjunctivitis in mucocutaneous disease | 37233 | B552 | Mucocutaneous leishmaniasis |
| conjunctivitis in mucocutaneous disease | 37233 | H1089 | Other conjunctivitis |
| other conjunctivitis | 37239 | H10511 | Ligneous conjunctivitis, right eye |
| other conjunctivitis | 37239 | H10512 | Ligneous conjunctivitis, left eye |
| other conjunctivitis | 37239 | H10513 | Ligneous conjunctivitis, bilateral |
| other conjunctivitis | 37239 | H10519 | Ligneous conjunctivitis, unspecified eye |
| other conjunctivitis | 37239 | H1089 | Other conjunctivitis |
| other conjunctivitis | 37239 | H109 | Unspecified conjunctivitis |
| abscess of eyelid | 37313 | H00031 | Abscess of right upper eyelid |
| abscess of eyelid | 37313 | H00032 | Abscess of right lower eyelid |
| abscess of eyelid | 37313 | H00033 | Abscess of eyelid right eye, unspecified eyelid |
| abscess of eyelid | 37313 | H00034 | Abscess of left upper eyelid |
| abscess of eyelid | 37313 | H00035 | Abscess of left lower eyelid |
| abscess of eyelid | 37313 | H00036 | Abscess of eyelid left eye, unspecified eyelid |
| abscess of eyelid | 37313 | H00039 | Abscess of eyelid unspecified eye, unspecified eyelid |
| infective dermatitis of eyelid of types resulting in deformity | 3734 | H018 | Other specified inflammations of eyelid |
| other infective dermatitis of eyelid | 3735 | H018 | Other specified inflammations of eyelid |
| other inflammations of eyelids | 3738 | H018 | Other specified inflammations of eyelid |
| unspecified inflammation of eyelid | 3739 | H019 | Unspecified inflammation of eyelid |
| phlegmonous dacrocystitis | 37533 | H04311 | Phlegmonous dacryocystitis of right lacrimal passage |
| phlegmonous dacrocystitis | 37533 | H04312 | Phlegmonous dacryocystitis of left lacrimal passage |
| phlegmonous dacrocystitis | 37533 | H04313 | Phlegmonous dacryocystitis of bilateral lacrimal passages |
| phlegmonous dacrocystitis | 37533 | H04319 | Phlegmonous dacryocystitis of unspecified lacrimal passage |
| acute inflammation of orbit, unspecified | 37600 | H0500 | Unspecified acute inflammation of orbit |
| orbital cellulitis | 37601 | H05011 | Cellulitis of right orbit |
| **Appendix B Table 1-EENTM: IDC-9 and ICD-10 Codes for Eye, Ear, Nose, Throat, and Mouth Infections cont.** | | | |
| **ICD-9 Description** | **icd9_eentm** | **icd10_eentm** | **ICD-10 Description** |
| orbital cellulitis | 37601 | H05012 | Cellulitis of left orbit |
| orbital cellulitis | 37601 | H05013 | Cellulitis of bilateral orbits |
| orbital cellulitis | 37601 | H05019 | Cellulitis of unspecified orbit |
| orbital periostitis | 37602 | H05031 | Periostitis of right orbit |
| orbital periostitis | 37602 | H05032 | Periostitis of left orbit |
| orbital periostitis | 37602 | H05033 | Periostitis of bilateral orbits |
| orbital periostitis | 37602 | H05039 | Periostitis of unspecified orbit |
| orbital osteomyelitis | 37603 | H05021 | Osteomyelitis of right orbit |
| orbital osteomyelitis | 37603 | H05022 | Osteomyelitis of left orbit |
| orbital osteomyelitis | 37603 | H05023 | Osteomyelitis of bilateral orbits |
| orbital osteomyelitis | 37603 | H05029 | Osteomyelitis of unspecified orbit |
| orbital tenonitis | 37604 | H05041 | Tenonitis of right orbit |
| orbital tenonitis | 37604 | H05042 | Tenonitis of left orbit |
| orbital tenonitis | 37604 | H05043 | Tenonitis of bilateral orbits |
| orbital tenonitis | 37604 | H05049 | Tenonitis of unspecified orbit |
| infective otitis externa, unspecified | 38010 | H6000 | Abscess of external ear, unspecified ear |
| infective otitis externa, unspecified | 38010 | H6001 | Abscess of right external ear |
| infective otitis externa, unspecified | 38010 | H6002 | Abscess of left external ear |
| infective otitis externa, unspecified | 38010 | H6003 | Abscess of external ear, bilateral |
| infective otitis externa, unspecified | 38010 | H6010 | Cellulitis of external ear, unspecified ear |
| infective otitis externa, unspecified | 38010 | H6011 | Cellulitis of right external ear |
| infective otitis externa, unspecified | 38010 | H6012 | Cellulitis of left external ear |
| infective otitis externa, unspecified | 38010 | H6013 | Cellulitis of external ear, bilateral |
| infective otitis externa, unspecified | 38010 | H60311 | Diffuse otitis externa, right ear |
| infective otitis externa, unspecified | 38010 | H60312 | Diffuse otitis externa, left ear |
| infective otitis externa, unspecified | 38010 | H60313 | Diffuse otitis externa, bilateral |
| infective otitis externa, unspecified | 38010 | H60319 | Diffuse otitis externa, unspecified ear |
| infective otitis externa, unspecified | 38010 | H60321 | Hemorrhagic otitis externa, right ear |
| infective otitis externa, unspecified | 38010 | H60322 | Hemorrhagic otitis externa, left ear |
| infective otitis externa, unspecified | 38010 | H60323 | Hemorrhagic otitis externa, bilateral |
| **Appendix B Table 1-EENTM: IDC-9 and ICD-10 Codes for Eye, Ear, Nose, Throat, and Mouth Infections cont.** | | | |
| **ICD-9 Description** | **icd9_eentm** | **icd10_eentm** | **ICD-10 Description** |
| infective otitis externa, unspecified | 38010 | H60329 | Hemorrhagic otitis externa, unspecified ear |
| infective otitis externa, unspecified | 38010 | H60391 | Other infective otitis externa, right ear |
| infective otitis externa, unspecified | 38010 | H60392 | Other infective otitis externa, left ear |
| infective otitis externa, unspecified | 38010 | H60393 | Other infective otitis externa, bilateral |
| infective otitis externa, unspecified | 38010 | H60399 | Other infective otitis externa, unspecified ear |
| Disorder of external ear, unspecified, bilateral | 38011 | H6190 | Disorder of external ear, unspecified |
| Disorder of external ear, unspecified, bilateral | 38011 | H6191 | Disorder of right external ear, unspecified |
| Disorder of external ear, unspecified, bilateral | 38011 | H6192 | Disorder of left external ear, unspecified |
| Disorder of external ear, unspecified, bilateral | 38011 | H6193 | Disorder of external ear, bilateral |
| Acute infection of pinna | 38012 | H60331 | Swimmer's ear, right ear |
| Acute infection of pinna | 38012 | H60332 | Swimmer's ear, left ear |
| Acute infection of pinna | 38012 | H60333 | Swimmer's ear, bilateral |
| Acute infection of pinna | 38012 | H60339 | Swimmer's ear, unspecified ear |
| Other acute otitis externa | 38022 | H60501 | Unspecified acute noninfective otitis externa, right ear |
| Other acute otitis externa | 38022 | H60502 | Unspecified acute noninfective otitis externa, left ear |
| Other acute otitis externa | 38022 | H60503 | Unspecified acute noninfective otitis externa, bilateral |
| Other acute otitis externa | 38022 | H60509 | Unspecified acute noninfective otitis externa, unspecified ear |
| Other acute otitis externa | 38022 | H60511 | Acute actinic otitis externa, right ear |
| Other acute otitis externa | 38022 | H60512 | Acute actinic otitis externa, left ear |
| Other acute otitis externa | 38022 | H60513 | Acute actinic otitis externa, bilateral |
| Other acute otitis externa | 38022 | H60519 | Acute actinic otitis externa, unspecified ear |
| Other acute otitis externa | 38022 | H60521 | Acute chemical otitis externa, right ear |
| Other acute otitis externa | 38022 | H60522 | Acute chemical otitis externa, left ear |
| Other acute otitis externa | 38022 | H60523 | Acute chemical otitis externa, bilateral |
| Other acute otitis externa | 38022 | H60529 | Acute chemical otitis externa, unspecified ear |
| Other acute otitis externa | 38022 | H60531 | Acute contact otitis externa, right ear |
| Other acute otitis externa | 38022 | H60532 | Acute contact otitis externa, left ear |
| Other acute otitis externa | 38022 | H60533 | Acute contact otitis externa, bilateral |
| Other acute otitis externa | 38022 | H60539 | Acute contact otitis externa, unspecified ear |
| Other acute otitis externa | 38022 | H60541 | Acute eczematoid otitis externa, right ear |
| **Appendix B Table 1-EENTM: IDC-9 and ICD-10 Codes for Eye, Ear, Nose, Throat, and Mouth Infections cont.** | | | |
| **ICD-9 Description** | **icd9_eentm** | **icd10_eentm** | **ICD-10 Description** |
| Other acute otitis externa | 38022 | H60542 | Acute eczematoid otitis externa, left ear |
| Other acute otitis externa | 38022 | H60543 | Acute eczematoid otitis externa, bilateral |
| Other acute otitis externa | 38022 | H60549 | Acute eczematoid otitis externa, unspecified ear |
| Other acute otitis externa | 38022 | H60551 | Acute reactive otitis externa, right ear |
| Other acute otitis externa | 38022 | H60552 | Acute reactive otitis externa, left ear |
| Other acute otitis externa | 38022 | H60553 | Acute reactive otitis externa, bilateral |
| Other acute otitis externa | 38022 | H60559 | Acute reactive otitis externa, unspecified ear |
| Other acute otitis externa | 38022 | H60591 | Other noninfective acute otitis externa, right ear |
| Other acute otitis externa | 38022 | H60592 | Other noninfective acute otitis externa, left ear |
| Other acute otitis externa | 38022 | H60593 | Other noninfective acute otitis externa, bilateral |
| Other acute otitis externa | 38022 | H60599 | Other noninfective acute otitis externa, unspecified ear |
| nonsuppartive otitis media, not specified as acute or chronic | 3814 | H6590 | Unspecified nonsuppurative otitis media, unspecified ear |
| nonsuppartive otitis media, not specified as acute or chronic | 3814 | H6591 | Unspecified nonsuppurative otitis media, right ear |
| nonsuppartive otitis media, not specified as acute or chronic | 3814 | H6592 | Unspecified nonsuppurative otitis media, left ear |
| nonsuppartive otitis media, not specified as acute or chronic | 3814 | H6593 | Unspecified nonsuppurative otitis media, bilateral |
| Eustachian salpingitis, unspecified | 38150 | H68001 | Unspecified Eustachian salpingitis, right ear |
| Eustachian salpingitis, unspecified | 38150 | H68002 | Unspecified Eustachian salpingitis, left ear |
| Eustachian salpingitis, unspecified | 38150 | H68003 | Unspecified Eustachian salpingitis, bilateral |
| Eustachian salpingitis, unspecified | 38150 | H68009 | Unspecified Eustachian salpingitis, unspecified ear |
| acute Eustachian salpingitis | 38151 | H68011 | Acute Eustachian salpingitis, right ear |
| acute Eustachian salpingitis | 38151 | H68012 | Acute Eustachian salpingitis, left ear |
| acute Eustachian salpingitis | 38151 | H68013 | Acute Eustachian salpingitis, bilateral |
| acute Eustachian salpingitis | 38151 | H68019 | Acute Eustachian salpingitis, unspecified ear |
| other disorders of Eustachain tube (including post-infectious) | 38189 | H6980 | Other specified disorders of Eustachian tube, unspecified ear |
| other disorders of Eustachain tube (including post-infectious) | 38189 | H6981 | Other specified disorders of Eustachian tube, right ear |
| other disorders of Eustachain tube (including post-infectious) | 38189 | H6982 | Other specified disorders of Eustachian tube, left ear |
| other disorders of Eustachain tube (including post-infectious) | 38189 | H6983 | Other specified disorders of Eustachian tube, bilateral |
| Acute suppurative otitis media without spontaneous rupture of eardrum | 38200 | H66001 | Acute suppurative otitis media without spontaneous rupture of ear drum, right ear |
| Acute suppurative otitis media without spontaneous rupture of eardrum | 38200 | H66002 | Acute suppurative otitis media without spontaneous rupture of ear drum, left ear |
| **Appendix B Table 1-EENTM: IDC-9 and ICD-10 Codes for Eye, Ear, Nose, Throat, and Mouth Infections cont.** | | | |
| **ICD-9 Description** | **icd9_eentm** | **icd10_eentm** | **ICD-10 Description** |
| Acute suppurative otitis media without spontaneous rupture of eardrum | 38200 | H66003 | Acute suppurative otitis media without spontaneous rupture of ear drum, bilateral |
| Acute suppurative otitis media without spontaneous rupture of eardrum | 38200 | H66004 | Acute suppurative otitis media without spontaneous rupture of ear drum, recurrent, right ear |
| Acute suppurative otitis media without spontaneous rupture of eardrum | 38200 | H66005 | Acute suppurative otitis media without spontaneous rupture of ear drum, recurrent, left ear |
| Acute suppurative otitis media without spontaneous rupture of eardrum | 38200 | H66006 | Acute suppurative otitis media without spontaneous rupture of ear drum, recurrent, bilateral |
| Acute suppurative otitis media with spontaneous rupture of eardrum | 38201 | H6601 | Acute suppurative otitis media with spontaneous rupture of ear drum |
| Acute suppurative otitis media with spontaneous rupture of eardrum | 38201 | H66012 | Acute suppurative otitis media with spontaneous rupture of ear drum, left ear |
| Acute suppurative otitis media with spontaneous rupture of eardrum | 38201 | H66013 | Acute suppurative otitis media with spontaneous rupture of ear drum, bilateral |
| Acute suppurative otitis media with spontaneous rupture of eardrum | 38201 | H66014 | Acute suppurative otitis media with spontaneous rupture of ear drum, recurrent, right ear |
| Acute suppurative otitis media with spontaneous rupture of eardrum | 38201 | H66015 | Acute suppurative otitis media with spontaneous rupture of ear drum, recurrent, left ear |
| Acute suppurative otitis media with spontaneous rupture of eardrum | 38201 | H66016 | Acute suppurative otitis media with spontaneous rupture of ear drum, recurrent, bilateral |
| Acute suppurative otitis media with spontaneous rupture of eardrum | 38201 | H66017 | Acute suppurative otitis media with spontaneous rupture of ear drum, recurrent, unspecified ear |
| Acute suppurative otitis media with spontaneous rupture of eardrum | 38201 | H66019 | Acute suppurative otitis media with spontaneous rupture of ear drum, unspecified ear |
| acute suppurative otitis media in diseases classified elsewhere | 38202 | H671 | Otitis media in diseases classified elsewhere, right ear |
| acute suppurative otitis media in diseases classified elsewhere | 38202 | H672 | Otitis media in diseases classified elsewhere, left ear |
| acute suppurative otitis media in diseases classified elsewhere | 38202 | H673 | Otitis media in diseases classified elsewhere, bilateral |
| acute suppurative otitis media in diseases classified elsewhere | 38202 | H679 | Otitis media in diseases classified elsewhere, unspecified ear |
| unspecified suppuriative otitis media | 3824 | H6640 | Suppurative otitis media, unspecified, unspecified ear |
| unspecified suppuriative otitis media | 3824 | H6641 | Suppurative otitis media, unspecified, right ear |
| unspecified suppuriative otitis media | 3824 | H6642 | Suppurative otitis media, unspecified, left ear |
| unspecified suppuriative otitis media | 3824 | H6643 | Suppurative otitis media, unspecified, bilateral |
| unspecified otitis media | 3829 | H6690 | Otitis media, unspecified, unspecified ear |
| unspecified otitis media | 3829 | H6691 | Otitis media, unspecified, right ear |
| **Appendix B Table 1-EENTM: IDC-9 and ICD-10 Codes for Eye, Ear, Nose, Throat, and Mouth Infections cont.** | | | |
| **ICD-9 Description** | **icd9_eentm** | **icd10_eentm** | **ICD-10 Description** |
| unspecified otitis media | 3829 | H6692 | Otitis media, unspecified, left ear |
| unspecified otitis media | 3829 | H6693 | Otitis media, unspecified, bilateral |
| acute mastoiditis without complications | 38300 | H70001 | Acute mastoiditis without complications, right ear |
| acute mastoiditis without complications | 38300 | H70002 | Acute mastoiditis without complications, left ear |
| acute mastoiditis without complications | 38300 | H70003 | Acute mastoiditis without complications, bilateral |
| acute mastoiditis without complications | 38300 | H70009 | Acute mastoiditis without complications, unspecified ear |
| subperiosteal abscess of mastoid | 38301 | H70011 | Subperiosteal abscess of mastoid, right ear |
| subperiosteal abscess of mastoid | 38301 | H70012 | Subperiosteal abscess of mastoid, left ear |
| subperiosteal abscess of mastoid | 38301 | H70013 | Subperiosteal abscess of mastoid, bilateral |
| subperiosteal abscess of mastoid | 38301 | H70019 | Subperiosteal abscess of mastoid, unspecified ear |
| acute mastoiditis with other complications | 38302 | H70091 | Acute mastoiditis with other complications, right ear |
| acute mastoiditis with other complications | 38302 | H70092 | Acute mastoiditis with other complications, left ear |
| acute mastoiditis with other complications | 38302 | H70093 | Acute mastoiditis with other complications, bilateral |
| acute mastoiditis with other complications | 38302 | H70099 | Acute mastoiditis with other complications, unspecified ear |
| Petrositis, unspecified | 38320 | H70201 | Unspecified petrositis, right ear |
| Petrositis, unspecified | 38320 | H70202 | Unspecified petrositis, left ear |
| Petrositis, unspecified | 38320 | H70203 | Unspecified petrositis, bilateral |
| Petrositis, unspecified | 38320 | H70209 | Unspecified petrositis, unspecified ear |
| acute petrositis | 38321 | H70211 | Acute petrositis, right ear |
| acute petrositis | 38321 | H70212 | Acute petrositis, left ear |
| acute petrositis | 38321 | H70213 | Acute petrositis, bilateral |
| acute petrositis | 38321 | H70219 | Acute petrositis, unspecified ear |
| unspecified mastoiditis | 3839 | H7090 | Unspecified mastoiditis, unspecified ear |
| unspecified mastoiditis | 3839 | H7091 | Unspecified mastoiditis, right ear |
| unspecified mastoiditis | 3839 | H7092 | Unspecified mastoiditis, left ear |
| unspecified mastoiditis | 3839 | H7093 | Unspecified mastoiditis, bilateral |
| acute myringitis, unspecified | 38400 | H73001 | Acute myringitis, right ear |
| acute myringitis, unspecified | 38400 | H73002 | Acute myringitis, left ear |
| acute myringitis, unspecified | 38400 | H73003 | Acute myringitis, bilateral |
| acute myringitis, unspecified | 38400 | H73009 | Acute myringitis, unspecified ear |
| **Appendix B Table 1-EENTM: IDC-9 and ICD-10 Codes for Eye, Ear, Nose, Throat, and Mouth Infections cont.** | | | |
| **ICD-9 Description** | **icd9_eentm** | **icd10_eentm** | **ICD-10 Description** |
| acute myringitis, unspecified | 38400 | H7320 | Unspecified myringitis, unspecified ear |
| acute myringitis, unspecified | 38400 | H7321 | Unspecified myringitis, right ear |
| acute myringitis, unspecified | 38400 | H7323 | Unspecified myringitis, bilateral |
| bullous myringitis | 38401 | H73011 | Bullous myringitis, right ear |
| bullous myringitis | 38401 | H73012 | Bullous myringitis, left ear |
| bullous myringitis | 38401 | H73013 | Bullous myringitis, bilateral |
| bullous myringitis | 38401 | H73019 | Bullous myringitis, unspecified ear |
| other acute myringitis without mention of otitis media | 38409 | H73091 | Other acute myringitis, right ear |
| other acute myringitis without mention of otitis media | 38409 | H73092 | Other acute myringitis, left ear |
| other acute myringitis without mention of otitis media | 38409 | H73093 | Other acute myringitis, bilateral |
| other acute myringitis without mention of otitis media | 38409 | H73099 | Other acute myringitis, unspecified ear |
| acute maxillary sinusitis | 4610 | J0100 | Acute maxillary sinusitis, unspecified |
| acute maxillary sinusitis | 4610 | J0101 | Acute recurrent maxillary sinusitis |
| acute frontal sinusitis | 4611 | J0110 | Acute frontal sinusitis, unspecified |
| acute frontal sinusitis | 4611 | J0111 | Acute recurrent frontal sinusitis |
| acute sphenoidal sinusitis | 4613 | J0130 | Acute sphenoidal sinusitis, unspecified |
| acute sphenoidal sinusitis | 4613 | J0131 | Acute recurrent sphenoidal sinusitis |
| other acute sinusitis | 4618 | J0140 | Acute pansinusitis, unspecified |
| other acute sinusitis | 4618 | J0141 | Acute recurrent pansinusitis |
| other acute sinusitis | 4618 | J0180 | Other acute sinusitis |
| other acute sinusitis | 4618 | J0181 | Other acute recurrent sinusitis |
| acute sinusitis, unspecified | 4619 | J0190 | Acute sinusitis, unspecified |
| acute sinusitis, unspecified | 4619 | J0191 | Acute recurrent sinusitis, unspecified |
| acute pharyngitis | 462 | J028 | Acute pharyngitis due to other specified organisms |
| acute pharyngitis | 462 | J029 | Acute pharyngitis, unspecified |
| acute laryngitis without mention of obstruction | 46400 | J040 | Acute laryngitis |
| acute laryngitis with obstruction | 46401 | J050 | Acute obstructive laryngitis [croup] |
| acute tracheitis without mention of obstruction | 46410 | J0410 | Acute tracheitis without obstruction |
| acute traceitis with obstruction | 46411 | J0411 | Acute tracheitis with obstruction |
| Acute laryngotraceheitis without mention of obstruction | 46420 | J042 | Acute laryngotracheitis |
| **Appendix B Table 1-EENTM: IDC-9 and ICD-10 Codes for Eye, Ear, Nose, Throat, and Mouth Infections cont.** | | | |
| **ICD-9 Description** | **icd9_eentm** | **icd10_eentm** | **ICD-10 Description** |
| acute laryngotracheitis with obstruction | 46421 | J050 | Acute obstructive laryngitis [croup] |
| acute epiglottitis without mention of obstruction | 46430 | J0510 | Acute epiglottitis without obstruction |
| acute epiglotittis with obstruction | 46431 | J0511 | Acute epiglottitis with obstruction |
| croup | 4644 | J050 | Acute obstructive laryngitis [croup] |
| supraglottitis unspecified, without obstruction | 46450 | J0430 | Supraglottitis, unspecified, without obstruction |
| supraglottitis unspecified, with obstruction | 46451 | J0431 | Supraglottitis, unspecified, with obstruction |
| Acute laryngopharyngitis | 4650 | J060 | Acute laryngopharyngitis |
| acute upper respiratory infections of other multiple sites | 4658 | J069 | Acute upper respiratory infection, unspecified |
| acute upper respiratory infections of unspecified sites | 4659 | J069 | Acute upper respiratory infection, unspecified |
| peritonsilar abscess | 475 | J36 | Peritonsillar abscess |
| other disease of nasal cavity and sinuses (including cellulitis of nose, internal) | 47819 | J340 | Abscess, furuncle and carbuncle of nose |
| other disease of nasal cavity and sinuses (including cellulitis of nose, internal) | 47819 | J341 | Cyst and mucocele of nose and nasal sinus |
| other disease of nasal cavity and sinuses (including cellulitis of nose, internal) | 47819 | J3489 | Other specified disorders of nose and nasal sinuses |
| other disease of nasal cavity and sinuses (including cellulitis of nose, internal) | 47819 | J349 | Unspecified disorder of nose and nasal sinuses |
| other disease of nasal cavity and sinuses (including cellulitis of nose, internal) | 47819 | R0981 | Nasal congestion |
| cellulitis of the pharynx or nasopharynx | 47821 | J391 | Other abscess of pharynx |
| pulpitis | 5220 | K0401 | Reversible pulpitis |
| pulpitis | 5220 | K0402 | Irreversible pulpitis |
| Acute apical periodontitis of pulpal origin | 5224 | K044 | Acute apical periodontitis of pulpal origin |
| periapical abscess without sinus | 5225 | K047 | Periapical abscess without sinus |
| periapical abscess with sinus | 5227 | K046 | Periapical abscess with sinus |
| inflammatory conditions of the jaw (includes infection, abscess) | 5264 | M272 | Inflammatory conditions of jaws |
| abscess of salivary gland | 5273 | K113 | Abscess of salivary gland |
| Cellulitis and abscess or oral soft tissues | 5283 | K122 | Cellulitis and abscess of mouth |
| diseases of lips (includes abscess) | 5285 | K130 | Diseases of lips |
| other and unspecified disease of the oral soft tissue (includes infectious diseases) | 5289 | E08638 | Diabetes mellitus due to underlying condition with other oral complications |
|  |  |  |  |
|  |  |  |  |
|  |  |  |  |
| **Appendix B Table 1-EENTM: IDC-9 and ICD-10 Codes for Eye, Ear, Nose, Throat, and Mouth Infections cont.** | | | |
| **ICD-9 Description** | **icd9_eentm** | **icd10_eentm** | **ICD-10 Description** |
| other and unspecified disease of the oral soft tissue (includes infectious diseases) | 5289 | K136 | Irritative hyperplasia of oral mucosa |
| other and unspecified disease of the oral soft tissue (includes infectious diseases) | 5289 | K1370 | Unspecified lesions of oral mucosa |
| other and unspecified disease of the oral soft tissue (includes infectious diseases) | 5289 | K1379 | Other lesions of oral mucosa |
| other and unspecified disease of the oral soft tissue (includes infectious diseases) | 5289 | E09638 | Drug or chemical induced diabetes mellitus with other oral complications |
| other and unspecified disease of the oral soft tissue (includes infectious diseases) | 5289 | K131 | Cheek and lip biting |
| other and unspecified disease of the oral soft tissue (includes infectious diseases) | 5289 | K134 | Granuloma and granuloma-like lesions of oral mucosa |

**Appendix B Table 2-EENTM: Univariate Statistics 2011 and 2016**

| **Patient Characteristics** | **EENTM - Group Comparisons 2011** | | | **EENTM - Group Comparisons 2016** | | |
| --- | --- | --- | --- | --- | --- | --- |
|  | **Cases with Secondary Dx, LOS > 2 days** | **Cases with Principal DX** | **Matched Cases with No Dx** | **Cases with Secondary Dx, LOS > 2 days** | **Cases with Principal DX** | **Matched Cases with No Dx** |
|  |  |  |  |  |  |  |
|  |  |  |  |  |  |  |
| **n =** | 69,880 | 25,409 | 349,395 | 70,703 | 22,131 | 353,349 |
| **Variables:** |  |  |  |  |  |  |
| **Cost** |  |  |  |  |  |  |
| mean | $17,616 | $5,601 | $12,127 | $21,168 | $6,899 | $13,028 |
| median | $9,210 | $3,744 | $6,858 | $10,154 | $4,470 | $7,422 |
| mode | $9,221 | $78 | $78 | $9,445 | $1,465 | $3,148 |
| range | $81 - $1,620,419 | $31 - $651,016 | $31 - $1,220,386 | $31 - $2,371,529 | $44 - $638,305 | $16 - $2,372,384 |
| **LOS** |  |  |  |  |  |  |
| mean | 8.34 | 2.77 | 5.35 | 9.00 | 2.97 | 5.47 |
| median | 5 | 2 | 3 | 5 | 2 | 4 |
| mode | 3 | 1 | 2 | 3 | 1 | 2 |
| range | 3 - 365 | 1-210 | 1 - 365 | 3 - 358 | 1 -165 | 1 - 363 |
| **Number of Diagnoses; mean (SD)** | 12.01 (6.23) | 6.01 (4.55) | 9.88 (5.91) | 14.41 (6.83) | 7.56 (5.54) | 12.10 (6.76) |
| **Number of Procedures; mean (SD)** | 1.63 (2.57) | 0.71 (1.32) | 1.31 (2.09) | 1.71 (2.73) | 0.88 (1.56) | 1.34 (2.18) |
| **age; mean (range)** | 48 (0 - 121) | 31 (0 - 106) | 55 (0-123) | 48 (0 - 90) | 33 (0 - 90) | 55 (0-90) |
| **APRDRG_Severity; mean (SD)** | 2.43 (0.91) | 1.75 (0.79) | 2.29 (0.92) | 2.49 (0.90) | 1.81 (0.80) | 2.33 (0.92) |
| **APRDRG_Risk_Mortality; mean (SD)** | 1.90 (0.98) | 1.26 (0.58) | 1.87 (0.97) | 2.04 (1.01) | 1.32 (0.64) | 2.01 (1.01) |
| **Wi_X; mean (SD)** | 0.9936 (0.154) | 0.9956 (0.157) | 1.0007 (0.160) | 1.0051 (0.191) | 1.0223 (0.198) | 1.0020 (0.195) |

**Appendix B Table 3-EENTM: Results of Median Regression Models 2011 and 2016**

| **Median Regression Estimates for Cost of EENTM as a Principal Diagnosis (n=25355) 2011** | | | | | | | **Median Regression Estimates for Cost of EENTM as a Principal Diagnosis (n=22087) 2016** | | | | | | |
| --- | --- | --- | --- | --- | --- | --- | --- | --- | --- | --- | --- | --- | --- |
| **Parameter** | **Estimate** | **Standard Error** | **95% Confidence Limits** | | **t Value** | **Pr > \|t\|** | **Parameter** | **Estimate** | **Standard Error** | **95% Confidence Limits** | | **t Value** | **Pr > \|t\|** |
| **Intercept** | -5039.22 | 125.9295 | -5286.046 | -4792.387 | -40.02 | <.0001 | **Intercept** | -4031.27 | 100.5586 | -4228.371 | -3834.167 | -40.09 | <.0001 |
| **LOS** | 1315.817 | 19.9445 | 1276.7246 | 1354.9093 | 65.97 | <.0001 | **LOS** | 1445.403 | 23.0083 | 1400.3052 | 1490.5009 | 62.82 | <.0001 |
| **NDX** | 59.4397 | 6.1055 | 47.4725 | 71.4068 | 9.74 | <.0001 | **NDX** | 48.9485 | 5.7890 | 37.6017 | 60.2953 | 8.46 | <.0001 |
| **NPR** | 982.6232 | 29.4307 | 924.9373 | 1040.3091 | 33.39 | <.0001 | **NPR** | 1159.855 | 30.6852 | 1099.7099 | 1220.0004 | 37.80 | <.0001 |
| **URBAN_TEACH** | 338.5096 | 28.3699 | 282.9029 | 394.1162 | 11.93 | <.0001 | **URBAN_TEACH** | 167.7029 | 38.4388 | 92.3600 | 243.0457 | 4.36 | <.0001 |
| **URBAN_TEACH** | 0.0000 | 0.0000 | 0.0000 | 0.0000 | . | . | **URBAN_TEACH** | 0.0000 | 0.0000 | 0.0000 | 0.0000 | . | . |
| **SMALL_BEDSIZE** | -428.407 | 47.7117 | -521.9242 | -334.8888 | -8.98 | <.0001 | **SMALL_BEDSIZE** | -500.326 | 40.1790 | -579.0798 | -421.5725 | -12.45 | <.0001 |
| **SMALL_BEDSIZE** | 0.0000 | 0.0000 | 0.0000 | 0.0000 | . | . | **SMALL_BEDSIZE** | 0.0000 | 0.0000 | 0.0000 | 0.0000 | . | . |
| **AGE** | 2.9597 | 0.6594 | 1.6673 | 4.2521 | 4.49 | <.0001 | **AGE** | -2.3133 | 0.7722 | -3.8269 | -0.7996 | -3.00 | 0.0027 |
| **APRDRG_Severity** | 262.2263 | 27.2284 | 208.8570 | 315.5956 | 9.63 | <.0001 | **APRDRG_Severity** | 391.2379 | 32.0755 | 328.3676 | 454.1083 | 12.20 | <.0001 |
| **APRDRG_Risk_Mortality** | 310.0439 | 48.3846 | 215.2073 | 404.8805 | 6.41 | <.0001 | **APRDRG_Risk_Mortality** | 315.6229 | 52.5628 | 212.5961 | 418.6497 | 6.00 | <.0001 |
| **Wage_Index** | 4641.978 | 105.6163 | 4434.9641 | 4848.9920 | 43.95 | <.0001 | **Wage_Index** | 3703.944 | 81.1959 | 3544.7946 | 3863.0940 | 45.62 | <.0001 |

**Specified Stage 2 Cost Simulation Models for EENTM 2011 and 2016**

**Cost_eentm_2011 = -5039.22 + 1315.817*2.76775946 + 59.4397*NDX + 982.6232*NPR + 338.5096*URBAN_TEACH + (-428.407)*SMALL_BEDSIZE +**

**2.9597*AGE + 262.2263*APRDRG_Severity + 310.0439*APRDRG_Risk_Mortality + 4641.978*Wage_Index**

**Cost_eentm_2016 = -4031.27 + 1445.403*2.96624644 + 48.9485*NDX + 1159.855*NPR + 167.7029*URBAN_TEACH + (-500.326)*SMALL_BEDSIZE +**

**(-2.3133)*AGE + 391.2379*APRDRG_Severity + 315.6229*APRDRG_Risk_Mortality + 3703.944*Wage_Index**

**Gastrointestinal Infections**

| **Appendix B Table 1-GI: IDC-9 and ICD-10 Codes for Gastrointestinal Infections** | | | |
| --- | --- | --- | --- |
| **ICD-9 Description** | **icd9_gi** | **icd10_gi** | **ICD-10 Description** |
| Appendicitis, unqualified | 541 | K37 | Unspecified appendicitis |
| Other appendicitis | 542 | K36 | Other appendicitis |
| Other and unspecified diseases of Appendix B | 5439 | K381 | Appendicular concretions |
| Other and unspecified diseases of Appendix B | 5439 | K382 | Diverticulum of Appendix B |
| Other and unspecified diseases of Appendix B | 5439 | K383 | Fistula of Appendix B |
| Other and unspecified diseases of Appendix B | 5439 | K388 | Other specified diseases of Appendix B |
| Other and unspecified diseases of Appendix B | 5439 | K389 | Disease of Appendix B, unspecified |
| Peritonitis in infectious diseases classified elsewhere | 5670 | K67 | Disorders of peritoneum in infectious diseases classified elsewhere |
| Pneumococcal peritonitis | 5671 | K658 | Other peritonitis |
| Peritonitis (acute) generalized | 56721 | K650 | Generalized (acute) peritonitis |
| Peritoneal abscess | 56722 | K651 | Peritoneal abscess |
| Spontaneous bacterial peritonitis | 56723 | K652 | Spontaneous bacterial peritonitis |
| Other suppurative peritonitis | 56729 | K650 | Generalized (acute) peritonitis |
| Psoas muscle abscess | 56731 | K6812 | Psoas muscle abscess |
| Other retroperitoneal abscess | 56738 | K6819 | Other retroperitoneal abscess |
| Other retroperitoneal infections | 56739 | K689 | Other disorders of retroperitoneum |
| Choleperitonitis | 56781 | K653 | Choleperitonitis |
| Sclerosing meserteritis | 56782 | K654 | Sclerosing mesenteritis |
| Other retroperitoneal infections | 56789 | K658 | Other peritonitis |
| Unspecified peritonitis | 5679 | K659 | Peritonitis, unspecified |
| Abscess of intestine | 5695 | K50014 | Crohn's disease of small intestine with abscess |
| Abscess of intestine | 5695 | K50114 | Crohn's disease of large intestine with abscess |
| Abscess of intestine | 5695 | K50814 | Crohn's disease of both small and large intestine with abscess |
| Abscess of intestine | 5695 | K50914 | Crohn's disease, unspecified, with abscess |
| Abscess of intestine | 5695 | K51014 | Ulcerative (chronic) pancolitis with abscess |
| Abscess of intestine | 5695 | K51214 | Ulcerative (chronic) proctitis with abscess |
| Abscess of intestine | 5695 | K51314 | Ulcerative (chronic) rectosigmoiditis with abscess |
| **Appendix B Table 1-GI: IDC-9 and ICD-10 Codes for Gastrointestinal Infections cont.** | | | |
| **ICD-9 Description** | **icd9_gi** | **icd10_gi** | **ICD-10 Description** |
| Abscess of intestine | 5695 | K51414 | Inflammatory polyps of colon with abscess |
| Abscess of intestine | 5695 | K51514 | Left sided colitis with abscess |
| Abscess of intestine | 5695 | K51814 | Other ulcerative colitis with abscess |
| Abscess of intestine | 5695 | K51914 | Ulcerative colitis, unspecified with abscess |
| Abscess of intestine | 5695 | K5700 | Diverticulitis of small intestine with perforation and abscess without bleeding |
| Abscess of intestine | 5695 | K5701 | Diverticulitis of small intestine with perforation and abscess with bleeding |
| Abscess of intestine | 5695 | K5720 | Diverticulitis of large intestine with perforation and abscess without bleeding |
| Abscess of intestine | 5695 | K5721 | Diverticulitis of large intestine with perforation and abscess with bleeding |
| Abscess of intestine | 5695 | K5740 | Diverticulitis of both small and large intestine with perforation and abscess without bleeding |
| Abscess of intestine | 5695 | K5741 | Diverticulitis of both small and large intestine with perforation and abscess with bleeding |
| Abscess of intestine | 5695 | K5780 | Diverticulitis of intestine, part unspecified, with perforation and abscess without bleeding |
| Abscess of intestine | 5695 | K5781 | Diverticulitis of intestine, part unspecified, with perforation and abscess with bleeding |
| Abscess of intestine | 5695 | K630 | Abscess of intestine |
| Infection of colostomy and enterostomy | 56961, | K9402 | Colostomy infection |
| Infection of colostomy and enterostomy | 56961, | K9412 | Enterostomy infection |
| Pouchitis | 56971, | K91850 | Pouchitis |
| Perforation of intestine | 56983, | K631 | Perforation of intestine (nontraumatic) |
| Abscess of liver | 5720 | K750 | Abscess of liver |
| Portal pyemia | 5721 | K751 | Phlebitis of portal vein |
| Calculus of gallbladder with acute cholecystitis with obstruction | 57400 | K8000 | Calculus of gallbladder with acute cholecystitis without obstruction |
| Calculus of gallbladder with acute cholecystitis with obstruction | 57400 | K8012 | Calculus of gallbladder with acute and chronic cholecystitis without obstruction |
| Calculus of gallbladder with acute cholecystitis with obstruction | 57401 | K8001 | Calculus of gallbladder with acute cholecystitis with obstruction |
| Calculus of gallbladder with acute cholecystitis with obstruction | 57401 | K8013 | Calculus of gallbladder with acute and chronic cholecystitis with obstruction |
| Calculus of gallbladder with other cholecystitis with obstruction | 57411 | K8011 | Calculus of gallbladder with chronic cholecystitis with obstruction |
| Calculus of gallbladder with other cholecystitis with obstruction | 57411 | K8013 | Calculus of gallbladder with acute and chronic cholecystitis with obstruction |
| **Appendix B Table 1-GI: IDC-9 and ICD-10 Codes for Gastrointestinal Infections cont.** | | | |
| **ICD-9 Description** | **icd9_gi** | **icd10_gi** | **ICD-10 Description** |
| Calculus of gallbladder with other cholecystitis with obstruction | 57411 | K8019 | Calculus of gallbladder with other cholecystitis with obstruction |
| Calculus of gallbladder without cholecystitis without obstruction | 57420 | K8020 | Calculus of gallbladder without cholecystitis without obstruction |
| Calculus of gallbladder without cholecystitis without obstruction | 57420 | K8080 | Other cholelithiasis without obstruction |
| Calculus of gallbladder without cholecystitis with obstruction | 57421 | K8021 | Calculus of gallbladder without cholecystitis with obstruction |
| Calculus of bile duct with acute cholecystitis without obstruction | 57430 | K8042 | Calculus of bile duct with acute cholecystitis without obstruction |
| Calculus of bile duct with acute cholecystitis without obstruction | 57430 | K8046 | Calculus of bile duct with acute and chronic cholecystitis without obstruction |
| Calculus of bile duct with acute cholecystitis with obstruction | 57431 | K8043 | Calculus of bile duct with acute cholecystitis with obstruction |
| Calculus of bile duct with acute cholecystitis with obstruction | 57431 | K8047 | Calculus of bile duct with acute and chronic cholecystitis with obstruction |
| Calculus of bile duct with other cholecystitis without obstruction | 57440 | K8040 | Calculus of bile duct with cholecystitis, unspecified, without obstruction |
| Calculus of bile duct with other cholecystitis without obstruction | 57440 | K8044 | Calculus of bile duct with chronic cholecystitis without obstruction |
| Calculus of bile duct with other cholecystitis without obstruction | 57440 | K8046 | Calculus of bile duct with acute and chronic cholecystitis without obstruction |
| Calculus of bile duct with other cholecystitis with obstruction | 57441 | K8041 | Calculus of bile duct with cholecystitis, unspecified, with obstruction |
| Calculus of bile duct with other cholecystitis with obstruction | 57441 | K8045 | Calculus of bile duct with chronic cholecystitis with obstruction |
| Calculus of bile duct with other cholecystitis with obstruction | 57441 | K8047 | Calculus of bile duct with acute and chronic cholecystitis with obstruction |
| Calculus of bile duct with other cholecystitis with obstruction | 57441 | K8081 | Other cholelithiasis with obstruction |
| Calculus of bile duct without cholecystitis without obstruction | 57450 | K8030 | Calculus of bile duct with cholangitis, unspecified, without obstruction |
| Calculus of bile duct without cholecystitis without obstruction | 57450 | K8032 | Calculus of bile duct with acute cholangitis without obstruction |
| Calculus of bile duct without cholecystitis without obstruction | 57450 | K8034 | Calculus of bile duct with chronic cholangitis without obstruction |
| Calculus of bile duct without cholecystitis without obstruction | 57450 | K8036 | Calculus of bile duct with acute and chronic cholangitis without obstruction |
| Calculus of bile duct without cholecystitis without obstruction | 57450 | K8050 | Calculus of bile duct without cholangitis or cholecystitis without obstruction |
| Calculus of bile duct without cholecystitis with obstruction | 57451 | K8031 | Calculus of bile duct with cholangitis, unspecified, with obstruction |
| Calculus of bile duct without cholecystitis with obstruction | 57451 | K8033 | Calculus of bile duct with acute cholangitis with obstruction |
| Calculus of bile duct without cholecystitis with obstruction | 57451 | K8035 | Calculus of bile duct with chronic cholangitis with obstruction |
| Calculus of bile duct without cholecystitis with obstruction | 57451 | K8037 | Calculus of bile duct with acute and chronic cholangitis with obstruction |
| Calculus of bile duct without cholecystitis with obstruction | 57451 | K8051 | Calculus of bile duct without cholangitis or cholecystitis with obstruction |
|  |  |  |  |
|  |  |  |  |
| **Appendix B Table 1-GI: IDC-9 and ICD-10 Codes for Gastrointestinal Infections cont.** | | | |
| **ICD-9 Description** | **ICD-9** | **ICD-10** | **ICD-10 Description** |
| Calculus of gallbladder and bile duct with other cholecystitis without obstruction | 57470 | K8060 | Calculus of gallbladder and bile duct with cholecystitis, unspecified, without obstruction |
| Calculus of gallbladder and bile duct with other cholecystitis with obstruction | 57471 | K8061 | Calculus of gallbladder and bile duct with cholecystitis, unspecified, with obstruction |
| Calculus of gallbladder and bile duct with other cholecystitis without obstruction | 57470 | K8064 | Calculus of gallbladder and bile duct with chronic cholecystitis without obstruction |
| Calculus of gallbladder and bile duct with other cholecystitis with obstruction | 57471 | K8065 | Calculus of gallbladder and bile duct with chronic cholecystitis with obstruction |
| Calculus of gallbladder and bile duct with acute and chronic cholecystitis without obstruction | 57480 | K8066 | Calculus of gallbladder and bile duct with acute and chronic cholecystitis without obstruction |
| Calculus of gallbladder and bile duct with acute and chronic cholecystitis with obstruction | 57481 | K8067 | Calculus of gallbladder and bile duct with acute and chronic cholecystitis with obstruction |
| Calculus of gallbladder and bile duct without cholecystitis without obstruction | 57490 | K8070 | Calculus of gallbladder and bile duct without cholecystitis without obstruction |
| Calculus of gallbladder and bile duct without cholecystitis with obstruction | 57491 | K8071 | Calculus of gallbladder and bile duct without cholecystitis with obstruction |
| Acute cholecystitis | 5750 | K810 | Acute cholecystitis |
| Cholecystitis unspecified | 57510 | K819 | Cholecystitis, unspecified |
| Chronic cholecystitis | 57511 | K811 | Chronic cholecystitis |
| Acute and chronic cholecystitis | 57512 | K812 | Acute cholecystitis with chronic cholecystitis |
| Obstruction of gallbladder | 5752 | K820 | Obstruction of gallbladder |
| Hydrops of gallbladder | 5753 | K821 | Hydrops of gallbladder |
| Perforation of gallbladder | 5754 | K822 | Perforation of gallbladder |
| Fistula of gallbladder | 5755 | K823 | Fistula of gallbladder |
| Cholesterolosis of gallbladder | 5756 | K824 | Cholesterolosis of gallbladder |
| Other specified disorders of gallbladder | 5758 | K828 | Other specified diseases of gallbladder |
| Unspecified disorder of gallbladder | 5759 | K829 | Disease of gallbladder, unspecified |
| Postcholecystectomy syndrome | 5760 | K915 | Postcholecystectomy syndrome |
| Cholangitis | 5761 | K8030 | Calculus of bile duct with cholangitis, unspecified, without obstruction |
| Cholangitis | 5761 | K8031 | Calculus of bile duct with cholangitis, unspecified, with obstruction |
| Cholangitis | 5761 | K8032 | Calculus of bile duct with acute cholangitis without obstruction |
| Cholangitis | 5761 | K8033 | Calculus of bile duct with acute cholangitis with obstruction |
| Cholangitis | 5761 | K8034 | Calculus of bile duct with chronic cholangitis without obstruction |
| **Appendix B Table 1-GI: IDC-9 and ICD-10 Codes for Gastrointestinal Infections cont.** | | | |
| **ICD-9 Description** | **ICD-9** | **ICD-10** | **ICD-10 Description** |
| Cholangitis | 5761 | K8035 | Calculus of bile duct with chronic cholangitis with obstruction |
| Cholangitis | 5761 | K8036 | Calculus of bile duct with acute and chronic cholangitis without obstruction |
| Cholangitis | 5761 | K8037 | Calculus of bile duct with acute and chronic cholangitis with obstruction |
| Cholangitis | 5761 | K830 | Cholangitis |
| Obstruction of bile duct | 5762 | K831 | Obstruction of bile duct |
| Perforation of bile duct | 5763 | K832 | Perforation of bile duct |
| Fistula of bile duct | 5764 | K833 | Fistula of bile duct |
| Spasm of sphincter of oddi | 5765 | K834 | Spasm of sphincter of Oddi |
| Other specified disorders of biliary tract | 5768 | K835 | Biliary cyst |
| Other specified disorders of biliary tract | 5768 | K838 | Other specified diseases of biliary tract |
| Other specified disorders of biliary tract | 5768 | K87 | Disorders of gallbladder, biliary tract and pancreas in diseases classified elsewhere |
| Unspecified disorder of biliary tract | 5769 | K839 | Disease of biliary tract, unspecified |

**Appendix B Table 2-GI: Univariate Statistics 2011 and 2016**

| **Patient Characteristics** | **GI - Group Comparisons 2011** | | | **GI - Group Comparisons 2016** | | |
| --- | --- | --- | --- | --- | --- | --- |
|  | **Cases with Secondary Dx** | **Cases with Principal DX** | **Matched Cases with No Dx** | **Cases with Secondary Dx** | **Cases with Principal DX** | **Matched Cases with No Dx** |
|  |  |  |  |  |  |  |
|  |  |  |  |  |  |  |
| **n =** | 116,457 | 101,290 | 553,289 | 113,501 | 94,606 | 556,692 |
| **Variables:** |  |  |  |  |  |  |
| **Cost** |  |  |  |  |  |  |
| **mean** | $28,311 | $13,226 | $13,606 | $28,039 | $13,237 | $13,980 |
| **median** | $15,409 | $10,084 | $8,027 | $15,175 | $10,241 | $8,370 |
| **mode** | $4,151 | $2,767 | $78 | $9,445 | $7,567 | $3,148 |
| **range** | $68 - $1,452,246 | $195 - $884,259 | $30- $1,558,337 | $53 - $2,372,384 | $27- $1,974,915 | $20- $2,831,796 |
| **LOS** |  |  |  |  |  |  |
| **mean** | 10.28 | 4.33 | 5.44 | 10.13 | 4.63 | 5.48 |
| **median** | 7 | 3 | 4 | 6 | 3 | 4 |
| **mode** | 3 | 2 | 2 | 3 | 2 | 2 |
| **range** | 3 - 362 | 1- 267 | 1 - 365 | 3 - 363 | 1- 346 | 1 - 363 |
| **Number of Diagnoses; mean (SD)** | 13.97 (6.69) | 7.99 (5.61) | 10.66 (6.03) | 16.47 (6.87) | 9.35 (6.22) | 13.04 (6.79) |
| **Number of Procedures; mean (SD)** | 3.22 (3.49) | 2.08 (1.83) | 1.69 (2.26) | 3.22 (3.47) | 1.96 (1.94) | 1.71 (2.35) |
| **age; mean (range)** | 61 (0 -110) | 54 (0 - 108) | 59 (0-111) | 60 (0 -90) | 56 (0 - 90) | 58 (0-90) |
| **APRDRG_Severity; mean (SD)** | 3.00 (0.83) | 2.02 (0.88) | 2.38 (0.96) | 3.01 (0.81) | 1.96 (0.84) | 2.43 (0.95) |
| **APRDRG_Risk_Mortality; mean (SD)** | 2.45 (1.08) | 1.56 (0.85) | 2.00 (1.03) | 2.59 (1.06) | 1.59 (0.85) | 2.15 (1.07) |
| **Wage Index; mean (SD)** | 1.0067 (0.155) | 1.0095 (0.162) | 1.0015 (0.158) | 1.0167 (0.199) | 1.0147 (0.200) | 1.0033 (0.196) |

**Appendix B Table 3-GI: Results of Median Regression Models 2011 and 2016**

| **Median Regression Estimates for Cost of GI as a Principal Diagnosis (n=101198 ) 2011** | | | | | | | **Median Regression Estimates for Cost of GI as a Principal Diagnosis (n=94597) 2016** | | | | | | |
| --- | --- | --- | --- | --- | --- | --- | --- | --- | --- | --- | --- | --- | --- |
| **Parameter** | **Estimate** | **Standard Error** | **95% Confidence Limits** | | **t Value** | **Pr > \|t\|** | **Parameter** | **Estimate** | **Standard Error** | **95% Confidence Limits** | | **t Value** | **Pr > \|t\|** |
| **Intercept** | -5112.48 | 121.1984 | -5350.026 | -4874.932 | -42.18 | <.0001 | **Intercept** | -3631.49 | 102.3393 | -3832.078 | -3430.911 | -35.48 | <.0001 |
| **LOS** | 1490.666 | 11.4008 | 1468.3210 | 1513.0118 | 130.75 | <.0001 | **LOS** | 1333.642 | 10.6793 | 1312.7111 | 1354.5736 | 124.88 | <.0001 |
| **NDX** | 63.1412 | 5.3725 | 52.6111 | 73.6713 | 11.75 | <.0001 | **NDX** | 4.6365 | 3.8298 | -2.8697 | 12.1428 | 1.21 | 0.2260 |
| **NPR** | 1423.577 | 16.0217 | 1392.1749 | 1454.9794 | 88.85 | <.0001 | **NPR** | 1802.287 | 13.6084 | 1775.6151 | 1828.9596 | 132.44 | <.0001 |
| **URBAN_TEACH** | 1209.181 | 29.4626 | 1151.4348 | 1266.9273 | 41.04 | <.0001 | **URBAN_TEACH** | 972.0372 | 31.2677 | 910.7528 | 1033.3216 | 31.09 | <.0001 |
| **URBAN_TEACH** | 0.0000 | 0.0000 | 0.0000 | 0.0000 | . | . | **URBAN_TEACH** | 0.0000 | 0.0000 | 0.0000 | 0.0000 | . | . |
| **SMALL_BEDSIZE** | -1205.40 | 44.9726 | -1293.550 | -1117.258 | -26.80 | <.0001 | **SMALL_BEDSIZE** | -757.289 | 36.6843 | -829.1897 | -685.3882 | -20.64 | <.0001 |
| **SMALL_BEDSIZE** | 0.0000 | 0.0000 | 0.0000 | 0.0000 | . | . | **SMALL_BEDSIZE** | 0.0000 | 0.0000 | 0.0000 | 0.0000 | . | . |
| **AGE** | -8.6833 | 0.8787 | -10.4056 | -6.9610 | -9.88 | <.0001 | **AGE** | -9.9230 | 0.9590 | -11.8027 | -8.0433 | -10.35 | <.0001 |
| **APRDRG_Severity** | -79.8087 | 31.7433 | -142.0253 | -17.5922 | -2.51 | 0.0119 | **APRDRG_Severity** | 225.0201 | 30.3747 | 165.4859 | 284.5542 | 7.41 | <.0001 |
| **APRDRG_Risk_Mortality** | -34.0554 | 35.9262 | -104.4704 | 36.3596 | -0.95 | 0.3432 | **APRDRG_Risk_Mortality** | 95.3908 | 30.6388 | 35.3390 | 155.4425 | 3.11 | 0.0019 |
| **Wage_Index** | 8236.952 | 106.1588 | 8028.8815 | 8445.0215 | 77.59 | <.0001 | **Wage_Index** | 6244.817 | 83.0939 | 6081.9535 | 6407.6797 | 75.15 | <.0001 |

**Specified Stage 2 Cost Simulation Models for GI 2011 and 2016**

**Cost_gi_2011 = -5112.48 + 1490.666*4.33228354 + 63.1412*NDX + 1423.577*NPR + 1209.181*URBAN_TEACH + (-1205.40)*SMALL_BEDSIZE +**

**(-8.6833)*AGE + (-79.8087)*APRDRG_Severity + (-34.0554)*APRDRG_Risk_Mortality + 8236.952*Wage_Index**

**Cost_gi_2016 = -3631.49 + 1333.642*4.62510834 + (4.6365 )*NDX + 1802.287*NPR + 972.0372*URBAN_TEACH + (-757.289)*SMALL_BEDSIZE +**

**-9.9230*AGE + 225.0201*APRDRG_Severity + 95.3908*APRDRG_Risk_Mortality + 6244.817*Wage_Index**

**Pneumonia (PNEU)**

| **Appendix B Table 1-PNEU: IDC-9 and ICD-10 Codes for Pneumonia** | | | |
| --- | --- | --- | --- |
| **ICD-9 Description** | **icd9_pneu** | **icd10_pneu** | **ICD-10 Description** |
| Adenoviral Pneumonia | 4800 | J120 | Adenoviral pneumonia |
| Pneumonia due to respiratory syncytial virus (RSV) | 4801 | J121 | Respiratory syncytial virus pneumonia |
| Pneumonia due to parainfluenza virus | 4802 | J122 | Parainfluenza virus pneumonia |
| Pneumonia due to SARS-associated coronavirus | 4803 | J1281 | Pneumonia due to SARS-associated coronavirus |
| Pneumonia due to other virus not elsewhere classified | 4808 | J123 | Human metapneumovirus pneumonia |
| Pneumonia due to other virus not elsewhere classified | 4808 | J1289 | Other viral pneumonia |
| Viral pneumonia, unspecified | 4809 | J129 | Viral pneumonia, unspecified |
| Pneumococcal pneumonia; Streptococcus pneumoniae (pneumococcal disease) | 481 | J13 | Pneumonia due to Streptococcus pneumoniae |
| Pneumococcal pneumonia; Streptococcus pneumoniae (pneumococcal disease) | 481 | J181 | Lobar pneumonia, unspecified organism |
| Pneumonia due to Klebsiella pneumoniae | 4820 | J15 | Bacterial pneumonia, not elsewhere classified |
| Pneumonia due to Pseudomonas | 4821 | J151 | Pneumonia due to Pseudomonas |
| Pneumonia due to Haemophilus influenzae | 4822 | J14 | Pneumonia due to Hemophilus influenzae |
| Pneumonia due to Streptococcus (unspecified) | 48230 | J154 | Pneumonia due to other streptococci |
| Pneumonia Streptococcus A | 48231 | J154 | Pneumonia due to other streptococci |
| Pneumonia Streptococcus B | 48232 | J153 | Pneumonia due to streptococcus, group B |
| Pneumonia Other Strep | 48239 | J154 | Pneumonia due to other streptococci |
| Staphylococcal Pneumonia Nos | 48240 | J1520 | Pneumonia due to staphylococcus, unspecified |
| Staph Aureus Pneumonia | 48241 | J15211 | Pneumonia due to Methicillin susceptible Staphylococcus aureus |
| Methicillin resistant pneumonia due to Staphylococcus aureus | 48242 | J15212 | Pneumonia due to Methicillin resistant Staphylococcus aureus |
| Staph Pneumonia Nec | 48249 | J1529 | Pneumonia due to other staphylococcus |
| Pneumonia Anaerobes | 48281 | J158 | Pneumonia due to other specified bacteria |
| Pneumonia due to E. coli | 48282 | J155 | Pneumonia due to Escherichia coli |
| Pneumonia due to other gram-negative bacteria | 48283 | J156 | Pneumonia due to other Gram-negative bacteria |
| Legionnaires Disease | 48284 | A481 | Legionnaires' disease |
| Pneumonia due to other specified bacteria | 48289 | J158 | Pneumonia due to other specified bacteria |
| Bacterial pneumonia, unspecified | 4829 | J159 | Unspecified bacterial pneumonia |
| **Appendix B Table 1-PNEU: IDC-9 and ICD-10 Codes for Pneumonia cont.** | | | |
| **ICD-9 Description** | **icd9_pneu** | **icd10_pneu** | **ICD-10 Description** |
| Mycoplasma pneumonia | 4830 | J157 | Pneumonia due to Mycoplasma pneumoniae |
| Pneumonia Due To Chlamydia | 4831 | J160 | Chlamydial pneumonia |
| Pneumon Oth Spec Orgnsm | 4838 | J168 | Pneumonia due to other specified infectious organisms |
| Pneum In Infect Dis Nec | 4848 | B7781 | Ascariasis pneumonia |
| Unspecified infectious and parasitic diseases | 136.9 | B999 | Unspecified infectious disease |
| Bronchopneumonia, organism unspecified | 485 | J180 | Bronchopneumonia, unspecified organism |
| Pneumonia; community-acquired pneumonia (CAP); nosocomial pneumonia (hospital-acquired) | 486 | J188 | Other pneumonia, unspecified organism |
| Pneumonia; community-acquired pneumonia (CAP); nosocomial pneumonia (hospital-acquired) | 486 | J189 | Pneumonia, unspecified organism |

**Appendix B Table 2-PNEU: Univariate Statistics 2011 and 2016**

| **Patient Characteristics** | **PNEU - Group Comparisons 2011** | | | **PNEU - Group Comparisons 2016** | | |
| --- | --- | --- | --- | --- | --- | --- |
|  | **Cases with Secondary Dx, LOS > 2 days** | **Cases with Principal DX** | **Matched Cases with No Dx** | **Cases with Secondary Dx, LOS > 2 days** | **Cases with Principal DX** | **Matched Cases with No Dx** |
|  |  |  |  |  |  |  |
|  |  |  |  |  |  |  |
|  |  |  |  |  |  |  |
| **n =** | 276,384 | 211,745 | 1,158,662 | 285,522 | 156,343 | 1,122,370 |
| **Variables:** |  |  |  |  |  |  |
| **Cost** |  |  |  |  |  |  |
| **mean** | $29,712 | $10,424 | $13,876 | $27,139 | $9,887 | $14,186 |
| **median** | $15,914 | $6,930 | $8,327 | $14,449 | $6,935 | $8,585 |
| **mode** | $4,151 | $64,032 | $78 | $9,445 | $3,148 | $3,148 |
| **range** | $34 - $1,756,572 | $42 - $1,023,780 | $29 - $1,317,792 | $36 - $2,009,951 | $31 - $1,067,309 | $22 - $2,850,000 |
| **LOS** |  |  |  |  |  |  |
| **mean** | 11.04 | 5.07 | 5.47 | 10.10 | 4.73 | 5.50 |
| **median** | 7 | 4 | 4 | 7 | 4 | 4 |
| **mode** | 3 | 3 | 2 | 3 | 3 | 2 |
| **range** | 3 - 365 | 1-321 | 1 - 365 | 3 - 364 | 1 - 233 | 1 - 365 |
| **Number of Diagnoses; mean (SD)** | 15.51 (6.16) | 10.72 (5.72) | 11.34 (5.90) | 17.77 (6.20) | 12.67 (6.32) | 13.82 (6.55) |
| **Number of Procedures; mean (SD)** | 2.75 (3.53) | 0.70 (1.49) | 1.64 (2.36) | 2.47 (3.35) | 0.61 (1.42) | 1.56 (2.33) |
| **age; mean (range)** | 67 (0 - 111) | 61 (0 - 111) | 62 (0-121) | 66 (0 - 90) | 62 (0 - 90) | 62 (0 - 90) |
| **APRDRG_Severity; mean (SD)** | 3.35 (0.69) | 2.52 (0.86) | 2.47 (0.93) | 3.33 (0.68) | 2.52 (0.82) | 2.52 (0.89) |
| **APRDRG_Risk_Mortality; mean (SD)** | 3.10 (0.87) | 2.12 (0.95) | 2.13 (1.00) | 3.17 (0.84) | 2.26 (0.96) | 2.27 (1.02) |
| **Wage Index; mean (SD)** | 0.9961 (0.158) | 0.9792 (0.157) | 0.9975 (0.158) | 0.9902 (0.194) | 0.9736 (0.190) | 0.9995 (0.195) |

**Appendix B Table 2-PNEU: Results of Median Regression Models 2011 and 2016**

| **Median Regression Estimates for Cost of PNEU as a Principal Diagnosis (n=211596) 2011** | | | | | | | **Median Regression Estimates for Cost of PNEU as a Principal Diagnosis (n=156247) 2016** | | | | | | |
| --- | --- | --- | --- | --- | --- | --- | --- | --- | --- | --- | --- | --- | --- |
| **Parameter** | **Estimate** | **Standard Error** | **95% Confidence Limits** | | **t Value** | **Pr > \|t\|** | **Parameter** | **Estimate** | **Standard Error** | **95% Confidence Limits** | | **t Value** | **Pr > \|t\|** |
| **Intercept** | -6093.17 | 54.4183 | -6199.826 | -5986.508 | -111.97 | <.0001 | **Intercept** | -4477.65 | 62.442 | -4600.036 | -4355.266 | -71.71 | <.0001 |
| **LOS** | 1439.671 | 5.021 | 1429.8305 | 1449.5124 | 286.73 | <.0001 | **LOS** | 1420.994 | 5.8856 | 1409.4584 | 1432.5299 | 241.43 | <.0001 |
| **NDX** | 82.3438 | 2.084 | 78.2593 | 86.4283 | 39.51 | <.0001 | **NDX** | 47.7424 | 1.9247 | 43.97 | 51.5149 | 24.8 | <.0001 |
| **NPR** | 1396.829 | 15.8469 | 1365.7693 | 1427.8884 | 88.15 | <.0001 | **NPR** | 1166.507 | 16.1786 | 1134.7973 | 1198.2168 | 72.1 | <.0001 |
| **URBAN_TEACH** | 714.1943 | 15.136 | 684.5281 | 743.8606 | 47.19 | <.0001 | **URBAN_TEACH** | 794.5371 | 16.1066 | 762.9685 | 826.1057 | 49.33 | <.0001 |
| **URBAN_TEACH** | 0 | 0 | 0 | 0 | . | . | **URBAN_TEACH** | 0 | 0 | 0 | 0 | . | . |
| **SMALL_BEDSIZE** | -653.982 | 18.6294 | -690.4947 | -617.4684 | -35.1 | <.0001 | **SMALL_BEDSIZE** | -827.17 | 19.381 | -865.1562 | -789.1837 | -42.68 | <.0001 |
| **SMALL_BEDSIZE** | 0 | 0 | 0 | 0 | . | . | **SMALL_BEDSIZE** | 0 | 0 | 0 | 0 | . | . |
| **AGE** | -6.7812 | 0.2861 | -7.3421 | -6.2204 | -23.7 | <.0001 | **AGE** | -6.8097 | 0.3739 | -7.5425 | -6.0769 | -18.21 | <.0001 |
| **APRDRG_Severity** | 271.3773 | 14.5198 | 242.9189 | 299.8358 | 18.69 | <.0001 | **APRDRG_Severity** | 349.7959 | 18.2871 | 313.9535 | 385.6382 | 19.13 | <.0001 |
| **APRDRG_Risk_Mortality** | 141.5945 | 14.01 | 114.1352 | 169.0538 | 10.11 | <.0001 | **APRDRG_Risk_Mortality** | 114.5624 | 15.4982 | 84.1862 | 144.9386 | 7.39 | <.0001 |
| **Wage_Index** | 5973.446 | 51.6541 | 5872.2052 | 6074.6865 | 115.64 | <.0001 | **Wage_Index** | 5032.6 | 54.6301 | 4925.5263 | 5139.6741 | 92.12 | <.0001 |

**Specified Stage 2 Cost Simulation Models for PNEU 2011 and 2016**

**Cost_pneu_2011 = -6093.17 + 1439.671*5.07369714 + 82.3438*NDX + 1396.829*NPR + 714.1943*urban_teach + (-653.982)*small_bedsize +**

**(-6.7812)*AGE + 271.3773*APRDRG_Severity + 141.5945*APRDRG_Risk_Mortality + 5973.446*Wage_Index**

**Cost_pneu_2016 = -4477.65 + 1420.994*4.73024056 + 47.7424*NDX + 1166.507*NPR + 794.5371*URBAN_TEACH + (-827.17)*SMALL_BEDSIZE +**

**(-6.8097)*AGE + 349.7959*APRDRG_Severity + 114.5624*APRDRG_Risk_Mortality + 5032.60*Wage_Index**

**Reproductive Tract Infections (RTI)**

| **Appendix B Table 1-RTI: IDC-9 and ICD-10 Codes for Reproductive Tract Infections** | | | |
| --- | --- | --- | --- |
| **ICD-9 Description** | **icd9_rti** | **icd10_rti** | **ICD-10 Description** |
| Other inflammatory disorders of the penis (including abscess, boil, cellulitis) | 6072 | N4821 | Abscess of corpus cavernosum and penis |
| Other inflammatory disorders of the penis (including abscess, boil, cellulitis) | 6072 | N4822 | Cellulitis of corpus cavernosum and penis |
| Other inflammatory disorders of the penis (including abscess, boil, cellulitis) | 6072 | N4829 | Other inflammatory disorders of penis |
| Other inflammatory disorders of the male genital organs (including abscess, boil, cellulitis… of penis) | 6084 | N491 | Inflammatory disorders of spermatic cord, tunica vaginalis and vas deferens |
| Other inflammatory disorders of the male genital organs (including abscess, boil, cellulitis… of penis) | 6084 | N492 | Inflammatory disorders of scrotum |
| Other inflammatory disorders of the male genital organs (including abscess, boil, cellulitis… of penis) | 6084 | N493 | Fournier gangrene |
| Other inflammatory disorders of the male genital organs (including abscess, boil, cellulitis… of penis) | 6084 | N499 | Inflammatory disorder of unspecified male genital organ |
| unspecified inflammatory diseases of uterus, except cervix | 6159 | N719 | Inflammatory disease of uterus, unspecified |
| cervicitis and endocervicitis | 6160 | N72 | Inflammatory disease of cervix uteri |
| vaginitis and vulvovaginitis, in diseases classified elsewhere | 61611 | A5602 | Chlamydial vulvovaginitis |
| vaginitis and vulvovaginitis, in diseases classified elsewhere | 61611 | N771 | Vaginitis, vulvitis and vulvovaginitis in diseases classified elsewhere |
| abscess of bartholin's gland | 6163 | N751 | Abscess of Bartholin's gland |
| other abscess of vulva | 6164 | N764 | Abscess of vulva |
| ulceration of vulva, unspecified | 61650 | N766 | Ulceration of vulva |
| ulceration of vulva in diseases classified elsewhere | 61651 | N770 | Ulceration of vulva in diseases classified elsewhere |
| mucositis (ulcerative) of cervix, vagina and vulva | 61681 | N7681 | Mucositis (ulcerative) of vagina and vulva |
| Unspecified infection or infestation of mother, antepartum condition or complication | 63793 | O98911 | Unspecified maternal infectious and parasitic disease complicating pregnancy, first trimester |
| Unspecified infection or infestation of mother, antepartum condition or complication | 63793 | O98912 | Unspecified maternal infectious and parasitic disease complicating pregnancy, second trimester |
| **Appendix B Table 1-RTI: IDC-9 and ICD-10 Codes for Reproductive Tract Infections cont.** | | | |
| **ICD-9 Description** | **icd9_rti** | **icd10_rti** | **ICD-10 Description** |
| Unspecified infection or infestation of mother, antepartum condition or complication | 63793 | O98913 | Unspecified maternal infectious and parasitic disease complicating pregnancy, third trimester |
| Unspecified infection or infestation of mother, antepartum condition or complication | 63793 | O98919 | Unspecified maternal infectious and parasitic disease complicating pregnancy, unspecified trimester |
| Infections of the genitourinary tract in pregnancy, antepartum condition or complication | 64663 | O2301 | Infections of kidney in pregnancy, first trimester |
| Infections of the genitourinary tract in pregnancy, antepartum condition or complication | 64663 | O2302 | Infections of kidney in pregnancy, second trimester |
| Infections of the genitourinary tract in pregnancy, antepartum condition or complication | 64663 | O2303 | Infections of kidney in pregnancy, third trimester |
| Infections of the genitourinary tract in pregnancy, antepartum condition or complication | 64663 | O2311 | Infections of bladder in pregnancy, first trimester |
| Infections of the genitourinary tract in pregnancy, antepartum condition or complication | 64663 | O2312 | Infections of bladder in pregnancy, second trimester |
| Infections of the genitourinary tract in pregnancy, antepartum condition or complication | 64663 | O2313 | Infections of bladder in pregnancy, third trimester |
| Infections of the genitourinary tract in pregnancy, antepartum condition or complication | 64663 | O2321 | Infections of urethra in pregnancy, first trimester |
| Infections of the genitourinary tract in pregnancy, antepartum condition or complication | 64663 | O2322 | Infections of urethra in pregnancy, second trimester |
| Infections of the genitourinary tract in pregnancy, antepartum condition or complication | 64663 | O2323 | Infections of urethra in pregnancy, third trimester |
| Infections of the genitourinary tract in pregnancy, antepartum condition or complication | 64663 | O2331 | Infections of other parts of urinary tract in pregnancy, first trimester |
| Infections of the genitourinary tract in pregnancy, antepartum condition or complication | 64663 | O2332 | Infections of other parts of urinary tract in pregnancy, second trimester |
| Infections of the genitourinary tract in pregnancy, antepartum condition or complication | 64663 | O2333 | Infections of other parts of urinary tract in pregnancy, third trimester |
| **Appendix B Table 1-RTI: IDC-9 and ICD-10 Codes for Reproductive Tract Infections cont.** | | | |
| **ICD-9 Description** | **icd9_rti** | **icd10_rti** | **ICD-10 Description** |
| Infections of the genitourinary tract in pregnancy, antepartum condition or complication | 64663 | O2341 | Unspecified infection of urinary tract in pregnancy, first trimester |
| Infections of the genitourinary tract in pregnancy, antepartum condition or complication | 64663 | O2342 | Unspecified infection of urinary tract in pregnancy, second trimester |
| Infections of the genitourinary tract in pregnancy, antepartum condition or complication | 64663 | O2343 | Unspecified infection of urinary tract in pregnancy, third trimester |
| Infections of the genitourinary tract in pregnancy, antepartum condition or complication | 64663 | O23511 | Infections of cervix in pregnancy, first trimester |
| Infections of the genitourinary tract in pregnancy, antepartum condition or complication | 64663 | O23512 | Infections of cervix in pregnancy, second trimester |
| Infections of the genitourinary tract in pregnancy, antepartum condition or complication | 64663 | O23513 | Infections of cervix in pregnancy, third trimester |
| Infections of the genitourinary tract in pregnancy, antepartum condition or complication | 64663 | O23521 | Salpingo-oophoritis in pregnancy, first trimester |
| Infections of the genitourinary tract in pregnancy, antepartum condition or complication | 64663 | O23522 | Salpingo-oophoritis in pregnancy, second trimester |
| Infections of the genitourinary tract in pregnancy, antepartum condition or complication | 64663 | O23523 | Salpingo-oophoritis in pregnancy, third trimester |
| Infections of the genitourinary tract in pregnancy, antepartum condition or complication | 64663 | O23591 | Infection of other part of genital tract in pregnancy, first trimester |
| Infections of the genitourinary tract in pregnancy, antepartum condition or complication | 64663 | O23592 | Infection of other part of genital tract in pregnancy, second trimester |
| Infections of the genitourinary tract in pregnancy, antepartum condition or complication | 64663 | O23593 | Infection of other part of genital tract in pregnancy, third trimester |
| Infections of the genitourinary tract in pregnancy, antepartum condition or complication | 64663 | O2390 | Unspecified genitourinary tract infection in pregnancy, unspecified trimester |
| Infections of the genitourinary tract in pregnancy, antepartum condition or complication | 64663 | O2391 | Unspecified genitourinary tract infection in pregnancy, first trimester |
| **Appendix B Table 1-RTI: IDC-9 and ICD-10 Codes for Reproductive Tract Infections cont.** | | | |
| **ICD-9 Description** | **icd9_rti** | **icd10_rti** | **ICD-10 Description** |
| Infections of the genitourinary tract in pregnancy, antepartum condition or complication | 64663 | O2392 | Unspecified genitourinary tract infection in pregnancy, second trimester |
| Infections of the genitourinary tract in pregnancy, antepartum condition or complication | 64663 | O2393 | Unspecified genitourinary tract infection in pregnancy, third trimester |
| Unspecified infection or infestation of mother, unspecified as to episode of care or not applicable | 6479 | O98919 | Unspecified maternal infectious and parasitic disease complicating pregnancy, unspecified trimester |
| Infection of amniotic cavity, unspecified as to episode of care or not appicable | 6584 | O411090 | Infection of amniotic sac and membranes, unspecified, unspecified trimester, not applicable or unspecified |
| Infection of amniotic cavity, unspecified as to episode of care or not appicable | 6584 | O411091 | Infection of amniotic sac and membranes, unspecified, unspecified trimester, fetus 1 |
| Infection of amniotic cavity, unspecified as to episode of care or not appicable | 6584 | O411092 | Infection of amniotic sac and membranes, unspecified, unspecified trimester, fetus 2 |
| Infection of amniotic cavity, unspecified as to episode of care or not appicable | 6584 | O411093 | Infection of amniotic sac and membranes, unspecified, unspecified trimester, fetus 3 |
| Infection of amniotic cavity, unspecified as to episode of care or not appicable | 6584 | O411094 | Infection of amniotic sac and membranes, unspecified, unspecified trimester, fetus 4 |
| Infection of amniotic cavity, unspecified as to episode of care or not appicable | 6584 | O411095 | Infection of amniotic sac and membranes, unspecified, unspecified trimester, fetus 5 |
| Infection of amniotic cavity, unspecified as to episode of care or not appicable | 6584 | O411099 | Infection of amniotic sac and membranes, unspecified, unspecified trimester, other fetus |
| Infection of amniotic cavity, unspecified as to episode of care or not appicable | 6584 | O411290 | Chorioamnionitis, unspecified trimester, not applicable or unspecified |
| Infection of amniotic cavity, unspecified as to episode of care or not appicable | 6584 | O411291 | Chorioamnionitis, unspecified trimester, fetus 1 |
| Infection of amniotic cavity, unspecified as to episode of care or not appicable | 6584 | O411292 | Chorioamnionitis, unspecified trimester, fetus 2 |
| Infection of amniotic cavity, unspecified as to episode of care or not appicable | 6584 | O411293 | Chorioamnionitis, unspecified trimester, fetus 3 |
| **Appendix B Table 1-RTI: IDC-9 and ICD-10 Codes for Reproductive Tract Infections cont.** | | | |
| **ICD-9 Description** | **icd9_rti** | **icd10_rti** | **ICD-10 Description** |
| Infection of amniotic cavity, unspecified as to episode of care or not appicable | 6584 | O411294 | Chorioamnionitis, unspecified trimester, fetus 4 |
| Infection of amniotic cavity, unspecified as to episode of care or not appicable | 6584 | O411295 | Chorioamnionitis, unspecified trimester, fetus 5 |
| Infection of amniotic cavity, unspecified as to episode of care or not appicable | 6584 | O411299 | Chorioamnionitis, unspecified trimester, other fetus |
| Infection of amniotic cavity, unspecified as to episode of care or not appicable | 6584 | O411490 | Placentitis, unspecified trimester, not applicable or unspecified |
| Infection of amniotic cavity, unspecified as to episode of care or not appicable | 6584 | O411491 | Placentitis, unspecified trimester, fetus 1 |
| Infection of amniotic cavity, unspecified as to episode of care or not appicable | 6584 | O411492 | Placentitis, unspecified trimester, fetus 2 |
| Infection of amniotic cavity, unspecified as to episode of care or not appicable | 6584 | O411493 | Placentitis, unspecified trimester, fetus 3 |
| Infection of amniotic cavity, unspecified as to episode of care or not appicable | 6584 | O411494 | Placentitis, unspecified trimester, fetus 4 |
| Infection of amniotic cavity, unspecified as to episode of care or not appicable | 6584 | O411495 | Placentitis, unspecified trimester, fetus 5 |
| Infection of amniotic cavity, unspecified as to episode of care or not appicable | 6584 | O411499 | Placentitis, unspecified trimester, other fetus |
| Puerperal sepsis (postpartum sepsis) | 6702 | O85 | Puerperal sepsis |

**Appendix B Table 2-RTI: Univariate Statistics 2011 and 2016**

| **Patient Characteristics** | **RTI - Group Comparisons 2011** | | | **RTI - Group Comparisons 2016** | | |
| --- | --- | --- | --- | --- | --- | --- |
|  | **Cases with Secondary Dx, LOS > 2 days** | **Cases with Principal DX** | **Matched Cases with No Dx** | **Cases with Secondary Dx, LOS > 2 days** | **Cases with Principal DX** | **Matched Cases with No Dx** |
|  |  |  |  |  |  |  |
|  |  |  |  |  |  |  |
| **n =** | 8,825 | 9,495 | 44,125 | 9,799 | 8,578 | 48,995 |
| **Variables:** |  |  |  |  |  |  |
| **Cost** |  |  |  |  |  |  |
| **mean** | $14,045 | $5,599 | $9,012 | $16,733 | $7,008 | $9,830 |
| **median** | $8,531 | $3,829 | $5,354 | $9,558 | $4,465 | $5,701 |
| **mode** | $4,151 | $175 | $2,834 | $9,969 | $6,646 | $3,148 |
| **range** | $235 - $636,044 | $78 - $280,975 | $73 - $492,370 | $223 - $685,282 | $250 - $328,965 | $31 - $881,125 |
| **LOS** |  |  |  |  |  |  |
| **mean** | 7.27 | 3.19 | 4.03 | 8.13 | 3.61 | 4.39 |
| **median** | 5 | 2 | 3 | 5 | 3 | 3 |
| **mode** | 3 | 2 | 2 | 3 | 2 | 2 |
| **range** | 3 - 350 | 1 - 74 | 1 - 238 | 3 - 361 | 1 -118 | 1 - 289 |
| **Number of Diagnoses; mean (SD)** | 11.01 (5.84) | 6.10 (4.29) | 7.64 (5.34) | 13.80 (6.49) | 7.97 (5.41) | 10.25 (6.43) |
| **Number of Procedures; mean (SD)** | 2.04 (2.39) | 0.67 (1.13) | 1.91 (1.86) | 2.37 (2.76) | 0.91 (1.61) | 1.91 (2.05) |
| **age; mean (range)** | 39 (0 -106) | 32 (0 -98) | 42 (0-103) | 41 (0-90) | 34 (0 -90) | 44 (0-90) |
| **APRDRG_Severity; mean (SD)** | 2.49 (0.82) | 1.77 (0.75) | 1.93 (0.90) | 2.58 (0.84) | 1.86 (0.76) | 2.09 (0.93) |
| **APRDRG_Risk_Mortality; mean (SD)** | 1.56 (0.91) | 1.18 (0.51) | 1.44 (0.85) | 1.79 (1.03) | 1.28 (0.65) | 1.64 (1.00) |
|  | 1.0099 (0.155) | 1.0001 (0.161) | 1.0102 (0.162) | 1.0141 (0.202) | 1.0044 (0.199) | 1.0102 (0.198) |

**Appendix B Table 3-RTI: Results of Median Regression Models 2011 and 2016**

| **Median Regression Estimates for Cost of RTI as a Principal Diagnosis (n=9484) 2011** | | | | | | | **Median Regression Estimates for Cost of RTI as a Principal Diagnosis (n=8578) 2016** | | | | | | |
| --- | --- | --- | --- | --- | --- | --- | --- | --- | --- | --- | --- | --- | --- |
| **Parameter** | **Estimate** | **Standard Error** | **95% Confidence Limits** | | **t Value** | **Pr > \|t\|** | **Parameter** | **Estimate** | **Standard Error** | **95% Confidence Limits** | | **t Value** | **Pr > \|t\|** |
| **Intercept** | -4811.57 | 236.6579 | -5275.474 | -4347.673 | -20.33 | <.0001 | **Intercept** | -4808.49 | 208.6256 | -5217.448 | -4399.536 | -23.05 | <.0001 |
| **LOS** | 1077.952 | 26.1932 | 1026.6075 | 1129.2962 | 41.15 | <.0001 | **LOS** | 1224.267 | 27.0428 | 1171.2571 | 1277.2778 | 45.27 | <.0001 |
| **NDX** | 66.4368 | 11.122 | 44.6354 | 88.2382 | 5.97 | <.0001 | **NDX** | 17.0887 | 9.9657 | -2.4466 | 36.6239 | 1.71 | 0.0864 |
| **NPR** | 1046.362 | 39.8371 | 968.2729 | 1124.4515 | 26.27 | <.0001 | **NPR** | 1162.286 | 46.6451 | 1070.8501 | 1253.7215 | 24.92 | <.0001 |
| **URBAN_TEACH** | 425.2636 | 41.4684 | 343.9766 | 506.5505 | 10.26 | <.0001 | **URBAN_TEACH** | 361.3846 | 61.0509 | 241.7102 | 481.059 | 5.92 | <.0001 |
| **URBAN_TEACH** | 0 | 0 | 0 | 0 | . | . | **URBAN_TEACH** | 0 | 0 | 0 | 0 | . | . |
| **SMALL_BEDSIZE** | -671.411 | 92.382 | -852.4992 | -490.3223 | -7.27 | <.0001 | **SMALL_BEDSIZE** | -529.542 | 77.2353 | -680.9419 | -378.1422 | -6.86 | <.0001 |
| **SMALL_BEDSIZE** | 0 | 0 | 0 | 0 | . | . | **SMALL_BEDSIZE** | 0 | 0 | 0 | 0 | . | . |
| **AGE** | 7.3295 | 2.0086 | 3.3922 | 11.2668 | 3.65 | 0.0003 | **AGE** | 7.4211 | 2.4414 | 2.6353 | 12.2068 | 3.04 | 0.0024 |
| **APRDRG_Severity** | 84.8594 | 43.9366 | -1.2657 | 170.9846 | 1.93 | 0.0535 | **APRDRG_Severity** | 285.1055 | 59.1316 | 169.1934 | 401.0177 | 4.82 | <.0001 |
| **APRDRG_Risk_Mortality** | 438.8791 | 102.69 | 237.5847 | 640.1734 | 4.27 | <.0001 | **APRDRG_Risk_Mortality** | 869.2903 | 108.4997 | 656.6048 | 1081.9758 | 8.01 | <.0001 |
| **Wage_Index** | 4714.327 | 202.0228 | 4318.3193 | 5110.3353 | 23.34 | <.0001 | **Wage_Index** | 3910.726 | 181.0106 | 3555.902 | 4265.5506 | 21.6 | <.0001 |

**Specified Stage 2 Cost Simulation Models for RTI 2011 and 2016**

**Cost_rti_2011 = -4811.57 + 1077.952*3.19473407 + 66.4368*NDX + 1046.362*NPR + 425.2636*URBAN_TEACH + (-671.411)*SMALL_BEDSIZE +**

**7.3295*AGE + 84.8594*APRDRG_Severity + 438.8791*APRDRG_Risk_Mortality + 4714.327*Wage_Index**

**Cost_rti_2016 = -4808.49 + 1224.267*3.61331313 + 17.0887*NDX + 1162.286*NPR + 361.3846*URBAN_TEACH + (-529.542)*SMALL_BEDSIZE +**

**7.4211*AGE + 285.1055*APRDRG_Severity + 869.2903*APRDRG_Risk_Mortality + 3910.726*Wage_Index**

**Surgical Site Infections (SSI)**

| **Appendix B Table-SSI: IDC-9 and ICD-10 Codes for Surgical Site Infections** | | | |
| --- | --- | --- | --- |
| **ICD-9 Description** | **ICD9_ssi** | **ICD10_ssi** | **ICD-10 Description** |
| infection of tracheostomy | 51901 | J9502 | Infection of tracheostomy stoma |
| mediastinitis | 5192 | J9851 | Mediastinitis |
| infection of esophagostomy | 53086 | K9432 | Esophagostomy infection |
| Infection of gastrostomy | 53641 | K9422 | Gastrostomy infection |
| Inflammation (infection) or postprocedural bleb, unspecified | 37960 | H5940 | Inflammation (infection) of postprocedural bleb, unspecified |
| Inflammation (infection) or postprocedural bleb, stage 1 | 37961 | H5941 | Inflammation (infection) of postprocedural bleb, stage 1 |
| Inflammation (infection) or postprocedural bleb, stage 2 | 37962 | H5942 | Inflammation (infection) of postprocedural bleb, stage 2 |
| Inflammation (infection) or postprocedural bleb, stage 3 | 37963 | H5943 | Inflammation (infection) of postprocedural bleb, stage 3 |
| infection due to other bariatric procedure | 53981 | K9581 | Infection due to other bariatric procedure |
| Postmastoidectomy complication, unspecified | 38330 | H95111 | Chronic inflammation of postmastoidectomy cavity, right ear |
| Infection and inflammatory reaction due to other internal prosthetic device, implant, graft | 99660 | T8579XA | Infection and inflammatory reaction due to other internal prosthetic devices, implants and grafts, initial encounter |
| Infection and inflammatory reaction due to cardiac device, implant, and graft | 99661 | T826XXA | Infection and inflammatory reaction due to cardiac valve prosthesis, initial encounter |
| Infection and inflammatory reaction due to cardiac device, implant, and graft | 99661 | T827XXA | Infection and inflammatory reaction due to other cardiac and vascular devices, implants and grafts, initial encounter |
| Infection and inflammatory reaction due to other vascular device, implant, and graft | 99662 | T827XXA | Infection and inflammatory reaction due to other cardiac and vascular devices, implants and grafts, initial encounter |
| Infection and inflammatory reaction due to other vascular device, implant, and graft | 99665 | T83590A | Infection and inflammatory reaction due to implanted urinary neurostimulation device, initial encounter |
| Infection and inflammatory reaction due to other vascular device, implant, and graft | 99665 | T83591A | Infection and inflammatory reaction due to implanted urinary sphincter, initial encounter |
| Infection and inflammatory reaction due to other vascular device, implant, and graft | 99665 | T83592A | Infection and inflammatory reaction due to indwelling ureteral stent, initial encounter |
| Infection and inflammatory reaction due to other vascular device, implant, and graft | 99665 | T83593A | Infection and inflammatory reaction due to other urinary stents, initial encounter |
| Infection and inflammatory reaction due to other vascular device, implant, and graft | 99665 | T83598A | Infection and inflammatory reaction due to other prosthetic device, implant and graft in urinary system, initial encounter |
| Infection and inflammatory reaction due to other vascular device, implant, and graft | 99665 | T8361XA | Infection and inflammatory reaction due to implanted penile prosthesis, initial encounter |

| **Appendix B Table-SSI: IDC-9 and ICD-10 Codes for Surgical Site Infections** | | | |
| --- | --- | --- | --- |
| **ICD-9 Description** | **ICD9_ssi** | **ICD10_ssi** | **ICD-10 Description** |
| Infection and inflammatory reaction due to other vascular device, implant, and graft | 99665 | T8362XA | Infection and inflammatory reaction due to implanted testicular prosthesis, initial encounter |
| Infection and inflammatory reaction due to other vascular device, implant, and graft | 99665 | T8369XA | Infection and inflammatory reaction due to other prosthetic device, implant and graft in genital tract, initial encounter |
| Infection and inflammatory reaction due to joint prosthesis -- in bone and joint section | 99666 | T8450XA | Infection and inflammatory reaction due to unspecified internal joint prosthesis, initial encounter |
| Infection and inflammatory reaction due to other internal orthopedic device, implant, and graft | 99667 | T8460XA | Infection and inflammatory reaction due to internal fixation device of unspecified site, initial encounter |
| Infection and inflammatory reaction due to other internal orthopedic device, implant, and graft | 99667 | T847XXA | Infection and inflammatory reaction due to other internal orthopedic prosthetic devices, implants and grafts, initial encounter |
| Infection and inflammatory reaction due to other internal prosthetic device, implant, graft **not orthopedic | 99669 | T8579XA | Infection and inflammatory reaction due to other internal prosthetic devices, implants and grafts, initial encounter |
| Disruption of wound, unspecified | 99830 | T8130XA | Disruption of wound, unspecified, initial encounter |
| Disruption of internal operation (surgical) wound | 99831 | T8132XA | Disruption of internal operation (surgical) wound, not elsewhere classified, initial encounter |
| Disruption of external operation (surgical) wound | 99832 | T8131XA | Disruption of external operation (surgical) wound, not elsewhere classified, initial encounter |
| Disruption of traumatic injury wound repair | 99833 | T8133XA | Disruption of traumatic injury wound repair, initial encounter |
| Infected postoperative seroma | 99851 | n/a |  |
| Other postoperative infection | 99859 | K6811 | Postprocedural retroperitoneal abscess |
| Infection of colostomy or enterostomy | 56961 | K9402 | Colostomy infection |
| Infection of colostomy or enterostomy | 56961 | K9412 | Enterostomy infection |
|  |  |  |  |
|  |  |  |  |
|  |  |  |  |
|  |  |  |  |
|  |  |  |  |
|  |  |  |  |
|  |  |  |  |
|  |  |  |  |

**Appendix B Table 2-SSI: Univariate Statistics 2011 and 2016**

| **Patient Characteristics** | **SSI - Group Comparisons 2011** | | | **SSI - Group Comparisons 2016** | | |
| --- | --- | --- | --- | --- | --- | --- |
|  | **Cases with Secondary Dx, LOS > 2 days** | **Cases with Principal DX** | **Matched Cases with No Dx** | **Cases with Secondary Dx, LOS > 2 days** | **Cases with Principal DX** | **Matched Cases with No Dx** |
|  |  |  |  |  |  |  |
|  |  |  |  |  |  |  |
| **n =** | 34,975 | 64,052 | 174,875 | 16,337 | 19,873 | 81,685 |
| **Variables:** |  |  |  |  |  |  |
| **Cost** |  |  |  |  |  |  |
| mean | $49,746 | $18,877 | $15,789 | $51,902 | $22,733 | $16,528 |
| median | $26,615 | $11,441 | $9,705 | $24,994 | $13,435 | $9,956 |
| mode | $3,751 | $6,601 | $78 | $8,978 | $22,039 | $3,148 |
| range | $34 - $1,756,572 | $51 - $1,063,794 | $31 - $1,296,286 | $31 - $2,072,496 | $54 - $713,909 | $47 - $1,648,340 |
| **LOS** |  |  |  |  |  |  |
| mean | 17.44 | 7.68 | 5.99 | 17.59 | 8.41 | 5.83 |
| median | 11 | 5 | 4 | 11 | 6 | 4 |
| mode | 4 | 3 | 2 | 4 | 3 | 2 |
| range | 3 - 365 | 1- 362 | 1 - 355 | 3 - 363 | 1- 215 | 1 - 358 |
| **Number of Diagnoses; mean (SD)** | 16.06 (6.67) | 11.46 (6.20) | 10.80 (6.06) | 18.30 (6.67) | 15.21 (6.90) | 13.06 (6.77) |
| **Number of Procedures; mean (SD)** | 4.88 (4.53) | 2.59 (2.57) | 2.04 (2.465) | 5.04 (4.35) | 3.29 (3.04) | 2.10 (2.56) |
| **age; mean (range)** | 60 (0 - 106) | 58 (0 - 103) | 60 (0-112) | 58 (0 -90) | 59 (0 - 90) | 59 (0-90) |
| **APRDRG_Severity; mean (SD)** | 3.16 (0.81) | 2.55 (0.91) | 2.40 (0.96) | 3.13 (0.80) | 2.77 (0.89) | 2.43 (0.93) |
| **APRDRG_Risk_Mortality; mean (SD)** | 2.63 (1.07) | 2.02 (1.02) | 2.02 (1.01) | 2.71 (1.03) | 2.40 (1.05) | 2.12 (1.03) |
| **Wage Index; mean (SD)** | 1.0093 (0.156) | 1.0011 (0.150) | 1.0036 (0.159) | 1.0079 (0.192) | 1.0087 (0.193) | 1.0056 (0.194) |

**Appendix B Table 3-SSI: Results of Median Regression Models 2011 and 2016**

| **Median Regression Estimates for Cost of SSI as a Principal Diagnosis (n=64040) 2011** | | | | | | | **Median Regression Estimates for Cost of SSI as a Principal Diagnosis (n=19870) 2016** | | | | | | |
| --- | --- | --- | --- | --- | --- | --- | --- | --- | --- | --- | --- | --- | --- |
| **Parameter** | **Estimate** | **Standard Error** | **95% Confidence Limits** | | **t Value** | **Pr > \|t\|** | **Parameter** | **Estimate** | **Standard Error** | **95% Confidence Limits** | | **t Value** | **Pr > \|t\|** |
| **Intercept** | -11073.9 | 229.7128 | -11524.12 | -10623.65 | -48.21 | <.0001 | **Intercept** | -10774.9 | 307.5955 | -11377.85 | -10172.02 | -35.03 | <.0001 |
| **LOS** | 1437.191 | 12.2775 | 1413.1269 | 1461.2546 | 117.06 | <.0001 | **LOS** | 1728.372 | 22.2199 | 1684.8187 | 1771.9246 | 77.78 | <.0001 |
| **NDX** | 48.1119 | 6.2082 | 35.9437 | 60.2800 | 7.75 | <.0001 | **NDX** | 5.7985 | 11.0851 | -15.9292 | 27.5262 | 0.52 | 0.6009 |
| **NPR** | 2047.908 | 23.0889 | 2002.6537 | 2093.1624 | 88.70 | <.0001 | **NPR** | 1818.593 | 40.6912 | 1738.8350 | 1898.3513 | 44.69 | <.0001 |
| **URBAN_TEACH** | 461.5828 | 44.4764 | 374.4090 | 548.7566 | 10.38 | <.0001 | **URBAN_TEACH** | -148.774 | 99.2938 | -343.3984 | 45.8498 | -1.50 | 0.1341 |
| **URBAN_TEACH** | 0.0000 | 0.0000 | 0.0000 | 0.0000 | . | . | **URBAN_TEACH** | 0.0000 | 0.0000 | 0.0000 | 0.0000 | . | . |
| **SMALL_BEDSIZE** | -720.862 | 69.7916 | -857.6532 | -584.0702 | -10.33 | <.0001 | **SMALL_BEDSIZE** | 47.8525 | 126.7527 | -200.5934 | 296.2984 | 0.38 | 0.7058 |
| **SMALL_BEDSIZE** | 0.0000 | 0.0000 | 0.0000 | 0.0000 | . | . | **SMALL_BEDSIZE** | 0.0000 | 0.0000 | 0.0000 | 0.0000 | . | . |
| **AGE** | -13.0971 | 1.1015 | -15.2560 | -10.9381 | -11.89 | <.0001 | **AGE** | -19.9862 | 2.5054 | -24.8970 | -15.0754 | -7.98 | <.0001 |
| **APRDRG_Severity** | 240.5360 | 45.3626 | 151.6252 | 329.4468 | 5.30 | <.0001 | **APRDRG_Severity** | -222.361 | 88.2437 | -395.3265 | -49.3965 | -2.52 | 0.0117 |
| **APRDRG_Risk_Mortality** | 331.0729 | 41.9590 | 248.8333 | 413.3125 | 7.89 | <.0001 | **APRDRG_Risk_Mortality** | 535.0581 | 86.1788 | 366.1404 | 703.9758 | 6.21 | <.0001 |
| **Wage_Index** | 10614.84 | 205.5821 | 10211.903 | 11017.785 | 51.63 | <.0001 | **Wage_index** | 10549.75 | 245.9032 | 10067.764 | 11031.746 | 42.90 | <.0001 |

**Specified Stage 2 Cost Simulation Models for SSI 2011 and 2016**

**Cost_ssi_2011 = -11073.9+ 1437.191*7.67960407+ 48.1119*NDX + 2047.908*NPR + 461.5828*URBAN_TEACH + (-720.862)*SMALL_BEDSIZE +**

**(-13.0971)*AGE + 240.5360*APRDRG_Severity + 331.0729*APRDRG_Risk_Mortality + 10614.84*Wage_Index**

**Cost_ssi_2016 = -10774.9+ 1728.372*8.41503548 + 5.7985*NDX + 1818.593*NPR + (-148.774)*URBAN_TEACH + 47.8525*SMALL_BEDSIZE +**

**-19.9862*AGE + (-222.361)*APRDRG_Severity + 535.0581*APRDRG_Risk_Mortality + 10549.75*Wage_Index**

**Skin and Soft Tissue Infections (SSTI)**

| **Appendix B Table-SSTI: IDC-9 and ICD-10 Codes for Skin and Soft Tissue Infections** | | |  |
| --- | --- | --- | --- |
| **ICD-9 Description** | **icd9_ssti** | **icd10_ssti** | **ICD-10 Description** |
| Erysipelas | 035 | A46 | Erysipelas |
| Gas gangrene | 400 | A480 | Gas gangrene |
| inflammatory disease of breast (including cellulitis) | 6110 | N610 | Mastitis without abscess |
| inflammatory disease of breast (including cellulitis) | 6110 | N611 | Abscess of the breast and nipple |
| Infections of nipple associated with childbirth, unspecified as to episode of care or not applicable | 67500 | O91019 | Infection of nipple associated with pregnancy, unspecified trimester |
| Infections of nipple associated with childbirth, delivered, with or without mention of antepartum condition | 67501 | O91011 | Infection of nipple associated with pregnancy, first trimester |
| Infections of nipple associated with childbirth, delivered, with or without mention of antepartum condition | 67501 | O91012 | Infection of nipple associated with pregnancy, second trimester |
| Infections of nipple associated with childbirth, delivered, with or without mention of antepartum condition | 67501 | O91013 | Infection of nipple associated with pregnancy, third trimester |
| Infections of nipple associated with childbirth, delivered, with or without mention of antepartum condition | 67501 | O91019 | Infection of nipple associated with pregnancy, unspecified trimester |
| Infections of nipple associated with childbirth, delivered, with mention of postpartum condition | 67502 | O9102 | Infection of nipple associated with the puerperium |
| Infections of nipple associated with childbirth, antepartum condition or complications | 67503 | O91011 | Infection of nipple associated with pregnancy, first trimester |
| Infections of nipple associated with childbirth, antepartum condition or complications | 67503 | O91012 | Infection of nipple associated with pregnancy, second trimester |
| Infections of nipple associated with childbirth, antepartum condition or complications | 67503 | O91013 | Infection of nipple associated with pregnancy, third trimester |
| Infections of nipple associated with childbirth, antepartum condition or complications | 67503 | O91019 | Infection of nipple associated with pregnancy, unspecified trimester |
| Infections of nipple associated with childbirth, postpartum condition or complication | 67504 | O9102 | Infection of nipple associated with the puerperium |
| Infections of nipple associated with childbirth, postpartum condition or complication | 67504 | O9103 | Infection of nipple associated with lactation |
| **Appendix B Table-SSTI: IDC-9 and ICD-10 Codes for Skin and Soft Tissue Infections cont.** | | | |
| **ICD-9 Description** | **icd9_ssti** | **icd10_ssti** | **ICD-10 Description** |
| Abscess of breast associated with childbirth, unspecified as to episode of care or not applicable | 67510 | O91119 | Abscess of breast associated with pregnancy, unspecified trimester |
| Abcess of breast associated with childbirth, delivered, with or without mention of antepartum condition | 67511 | O91111 | Abscess of breast associated with pregnancy, first trimester |
| Abcess of breast associated with childbirth, delivered, with or without mention of antepartum condition | 67511 | O91112 | Abscess of breast associated with pregnancy, second trimester |
| Abcess of breast associated with childbirth, delivered, with or without mention of antepartum condition | 67511 | O91113 | Abscess of breast associated with pregnancy, third trimester |
| Abcess of breast associated with childbirth, delivered, with or without mention of antepartum condition | 67511 | O91119 | Abscess of breast associated with pregnancy, unspecified trimester |
| Abscess of breast associated with childbirth, delivered, with mention of postpartum condition | 67512 | O9112 | Abscess of breast associated with the puerperium |
| Abscess of breast associated with childbirth, antepartum condition or complications | 67513 | O91111 | Abscess of breast associated with pregnancy, first trimester |
| Abscess of breast associated with childbirth, antepartum condition or complications | 67513 | O91112 | Abscess of breast associated with pregnancy, second trimester |
| Abscess of breast associated with childbirth, antepartum condition or complications | 67513 | O91113 | Abscess of breast associated with pregnancy, third trimester |
| Abscess of breast associated with childbirth, antepartum condition or complications | 67513 | O91119 | Abscess of breast associated with pregnancy, unspecified trimester |
| Abcess of breast associated with childbirth, postpartum condition or complication | 67514 | O9112 | Abscess of breast associated with the puerperium |
| Abcess of breast associated with childbirth, postpartum condition or complication | 67514 | O9113 | Abscess of breast associated with lactation |
| Other specified infections of the breast and nipple associated with childbirth, delivered, with or without mention of antepartum condition | 67581 | O9123 | Nonpurulent mastitis associated with lactation |
| Other specified infections of the breast and nipple associated with childbirth, postpartum condition or complication | 67584 | O9122 | Nonpurulent mastitis associated with the puerperium |
| Carbuncle and furuncle of trunk | 6802 | L02221 | Furuncle of abdominal wall |
| **Appendix B Table-SSTI: IDC-9 and ICD-10 Codes for Skin and Soft Tissue Infections cont.** | | | |
| **ICD-9 Description** | **icd9_ssti** | **icd10_ssti** | **ICD-10 Description** |
| Carbuncle and furuncle of trunk | 6802 | L02222 | Furuncle of back [any part, except buttock] |
| Carbuncle and furuncle of trunk | 6802 | L02223 | Furuncle of chest wall |
| Carbuncle and furuncle of trunk | 6802 | L02224 | Furuncle of groin |
| Carbuncle and furuncle of trunk | 6802 | L02225 | Furuncle of perineum |
| Carbuncle and furuncle of trunk | 6802 | L02226 | Furuncle of umbilicus |
| Carbuncle and furuncle of trunk | 6802 | L02229 | Furuncle of trunk, unspecified |
| Carbuncle and furuncle of trunk | 6802 | L02231 | Carbuncle of abdominal wall |
| Carbuncle and furuncle of trunk | 6802 | L02232 | Carbuncle of back [any part, except buttock] |
| Carbuncle and furuncle of trunk | 6802 | L02233 | Carbuncle of chest wall |
| Carbuncle and furuncle of trunk | 6802 | L02234 | Carbuncle of groin |
| Carbuncle and furuncle of trunk | 6802 | L02235 | Carbuncle of perineum |
| Carbuncle and furuncle of trunk | 6802 | L02236 | Carbuncle of umbilicus |
| Carbuncle and furuncle of trunk | 6802 | L02239 | Carbuncle of trunk, unspecified |
| Carbuncle and furuncle of upper arm and forearm | 6803 | L02421 | Furuncle of right axilla |
| Carbuncle and furuncle of upper arm and forearm | 6803 | L02422 | Furuncle of left axilla |
| Carbuncle and furuncle of upper arm and forearm | 6803 | L02423 | Furuncle of right upper limb |
| Carbuncle and furuncle of upper arm and forearm | 6803 | L02424 | Furuncle of left upper limb |
| Carbuncle and furuncle of upper arm and forearm | 6803 | L02429 | Furuncle of limb, unspecified |
| Carbuncle and furuncle of upper arm and forearm | 6803 | L02431 | Carbuncle of right axilla |
| Carbuncle and furuncle of upper arm and forearm | 6803 | L02432 | Carbuncle of left axilla |
| Carbuncle and furuncle of upper arm and forearm | 6803 | L02433 | Carbuncle of right upper limb |
| Carbuncle and furuncle of upper arm and forearm | 6803 | L02434 | Carbuncle of left upper limb |
| Carbuncle and furuncle of upper arm and forearm | 6803 | L02439 | Carbuncle of limb, unspecified |
| Carbuncle and furuncle of hand | 6804 | L02521 | Furuncle right hand |
| Carbuncle and furuncle of hand | 6804 | L02522 | Furuncle left hand |
| Carbuncle and furuncle of hand | 6804 | L02529 | Furuncle unspecified hand |
| Carbuncle and furuncle of hand | 6804 | L02531 | Carbuncle of right hand |
| Carbuncle and furuncle of hand | 6804 | L02532 | Carbuncle of left hand |
| Carbuncle and furuncle of hand | 6804 | L02539 | Carbuncle of unspecified hand |
| Carbuncle and furuncle of buttock | 6805 | L0232 | Furuncle of buttock |
| **Appendix B Table-SSTI: IDC-9 and ICD-10 Codes for Skin and Soft Tissue Infections cont.** | | | |
| **ICD-9 Description** | **icd9_ssti** | **icd10_ssti** | **ICD-10 Description** |
| Carbuncle and furuncle of buttock | 6805 | L0233 | Carbuncle of buttock |
| Carbuncle and furuncle of foot | 6807 | L02621 | Furuncle of right foot |
| Carbuncle and furuncle of foot | 6807 | L02622 | Furuncle of left foot |
| Carbuncle and furuncle of foot | 6807 | L02629 | Furuncle of unspecified foot |
| Carbuncle and furuncle of foot | 6807 | L02631 | Carbuncle of right foot |
| Carbuncle and furuncle of foot | 6807 | L02632 | Carbuncle of left foot |
| Carbuncle and furuncle of foot | 6807 | L02639 | Carbuncle of unspecified foot |
| Carbuncle and furuncle of other specified sites | 6808 | L02821 | Furuncle of head [any part, except face] |
| Carbuncle and furuncle of other specified sites | 6808 | L02828 | Furuncle of other sites |
| Carbuncle and furuncle of other specified sites | 6808 | L02831 | Carbuncle of head [any part, except face] |
| Carbuncle and furuncle of other specified sites | 6808 | L02838 | Carbuncle of other sites |
| Carbuncle and furuncle of unspecified site | 6809 | L0292 | Furuncle, unspecified |
| Carbuncle and furuncle of unspecified site | 6809 | L0293 | Carbuncle, unspecified |
| celluitis and abscess of finger, unspecified | 68100 | L02511 | Cutaneous abscess of right hand |
| celluitis and abscess of finger, unspecified | 68100 | L02512 | Cutaneous abscess of left hand |
| celluitis and abscess of finger, unspecified | 68100 | L02519 | Cutaneous abscess of unspecified hand |
| celluitis and abscess of finger, unspecified | 68100 | L03011 | Cellulitis of right finger |
| celluitis and abscess of finger, unspecified | 68100 | L03012 | Cellulitis of left finger |
| celluitis and abscess of finger, unspecified | 68100 | L03019 | Cellulitis of unspecified finger |
| celluitis and abscess of finger, unspecified | 68100 | L03021 | Acute lymphangitis of right finger |
| celluitis and abscess of finger, unspecified | 68100 | L03022 | Acute lymphangitis of left finger |
| celluitis and abscess of finger, unspecified | 68100 | L03029 | Acute lymphangitis of unspecified finger |
| felon | 68101 | L03011 | Cellulitis of right finger |
| felon | 68101 | L03012 | Cellulitis of left finger |
| felon | 68101 | L03019 | Cellulitis of unspecified finger |
| onychia and paronychia of finger | 68102 | L03011 | Cellulitis of right finger |
| onychia and paronychia of finger | 68102 | L03012 | Cellulitis of left finger |
| onychia and paronychia of finger | 68102 | L03019 | Cellulitis of unspecified finger |
| celluitis and abscess of toe, unspecified | 68110 | L02611 | Cutaneous abscess of right foot |
| celluitis and abscess of toe, unspecified | 68110 | L02612 | Cutaneous abscess of left foot |
| **Appendix B Table-SSTI: IDC-9 and ICD-10 Codes for Skin and Soft Tissue Infections cont.** | | | |
| **ICD-9 Description** | **icd9_ssti** | **icd10_ssti** | **ICD-10 Description** |
| celluitis and abscess of toe, unspecified | 68110 | L02619 | Cutaneous abscess of unspecified foot |
| celluitis and abscess of toe, unspecified | 68110 | L03031 | Cellulitis of right toe |
| celluitis and abscess of toe, unspecified | 68110 | L03032 | Cellulitis of left toe |
| celluitis and abscess of toe, unspecified | 68110 | L03039 | Cellulitis of unspecified toe |
| celluitis and abscess of toe, unspecified | 68110 | L03041 | Acute lymphangitis of right toe |
| celluitis and abscess of toe, unspecified | 68110 | L03042 | Acute lymphangitis of left toe |
| celluitis and abscess of toe, unspecified | 68110 | L03049 | Acute lymphangitis of unspecified toe |
| onychia and paronychia of toe | 68111 | L03031 | Cellulitis of right toe |
| onychia and paronychia of toe | 68111 | L03032 | Cellulitis of left toe |
| onychia and paronychia of toe | 68111 | L03039 | Cellulitis of unspecified toe |
| Cellullitis and abscess of unspecified digit | 6819 | L03019 | Cellulitis of unspecified finger |
| Cellullitis and abscess of unspecified digit | 6819 | L03029 | Acute lymphangitis of unspecified finger |
| Cellullitis and abscess of unspecified digit | 6819 | L03039 | Cellulitis of unspecified toe |
| Cellullitis and abscess of unspecified digit | 6819 | L03049 | Acute lymphangitis of unspecified toe |
| other cellulitis and abscess of face | 6820 | K122 | Cellulitis and abscess of mouth |
| other cellulitis and abscess of face | 6820 | L0201 | Cutaneous abscess of face |
| other cellulitis and abscess of face | 6820 | L03211 | Cellulitis of face |
| other cellulitis and abscess of face | 6820 | L03212 | Acute lymphangitis of face |
| other cellulitis and abscess of face | 6820 | L03213 | Periorbital cellulitis |
| Cellulitis and abscess of neck | 6821 | L0211 | Cutaneous abscess of neck |
| Cellulitis and abscess of neck | 6821 | L03221 | Cellulitis of neck |
| Cellulitis and abscess of neck | 6821 | L03222 | Acute lymphangitis of neck |
| Cellulitis and abscess of trunk | 6822 | L02211 | Cutaneous abscess of abdominal wall |
| Cellulitis and abscess of trunk | 6822 | L02212 | Cutaneous abscess of back [any part, except buttock] |
| Cellulitis and abscess of trunk | 6822 | L02213 | Cutaneous abscess of chest wall |
| Cellulitis and abscess of trunk | 6822 | L02214 | Cutaneous abscess of groin |
| Cellulitis and abscess of trunk | 6822 | L02215 | Cutaneous abscess of perineum |
| Cellulitis and abscess of trunk | 6822 | L02216 | Cutaneous abscess of umbilicus |
| Cellulitis and abscess of trunk | 6822 | L02219 | Cutaneous abscess of trunk, unspecified |
| Cellulitis and abscess of trunk | 6822 | L03311 | Cellulitis of abdominal wall |
| **Appendix B Table-SSTI: IDC-9 and ICD-10 Codes for Skin and Soft Tissue Infections cont.** | | | |
| **ICD-9 Description** | **icd9_ssti** | **icd10_ssti** | **ICD-10 Description** |
| Cellulitis and abscess of trunk | 6822 | L03312 | Cellulitis of back [any part except buttock] |
| Cellulitis and abscess of trunk | 6822 | L03313 | Cellulitis of chest wall |
| Cellulitis and abscess of trunk | 6822 | L03314 | Cellulitis of groin |
| Cellulitis and abscess of trunk | 6822 | L03315 | Cellulitis of perineum |
| Cellulitis and abscess of trunk | 6822 | L03316 | Cellulitis of umbilicus |
| Cellulitis and abscess of trunk | 6822 | L03319 | Cellulitis of trunk, unspecified |
| Cellulitis and abscess of trunk | 6822 | L03321 | Acute lymphangitis of abdominal wall |
| Cellulitis and abscess of trunk | 6822 | L03322 | Acute lymphangitis of back [any part except buttock] |
| Cellulitis and abscess of trunk | 6822 | L03323 | Acute lymphangitis of chest wall |
| Cellulitis and abscess of trunk | 6822 | L03324 | Acute lymphangitis of groin |
| Cellulitis and abscess of trunk | 6822 | L03325 | Acute lymphangitis of perineum |
| Cellulitis and abscess of trunk | 6822 | L03326 | Acute lymphangitis of umbilicus |
| Cellulitis and abscess of trunk | 6822 | L03329 | Acute lymphangitis of trunk, unspecified |
| cellulitis and abscess of upper arm and forearm | 6823 | L02411 | Cutaneous abscess of right axilla |
| cellulitis and abscess of upper arm and forearm | 6823 | L02412 | Cutaneous abscess of left axilla |
| cellulitis and abscess of upper arm and forearm | 6823 | L02413 | Cutaneous abscess of right upper limb |
| cellulitis and abscess of upper arm and forearm | 6823 | L02414 | Cutaneous abscess of left upper limb |
| cellulitis and abscess of upper arm and forearm | 6823 | L02419 | Cutaneous abscess of limb, unspecified |
| cellulitis and abscess of upper arm and forearm | 6823 | L03111 | Cellulitis of right axilla |
| cellulitis and abscess of upper arm and forearm | 6823 | L03112 | Cellulitis of left axilla |
| cellulitis and abscess of upper arm and forearm | 6823 | L03113 | Cellulitis of right upper limb |
| cellulitis and abscess of upper arm and forearm | 6823 | L03114 | Cellulitis of left upper limb |
| cellulitis and abscess of upper arm and forearm | 6823 | L03119 | Cellulitis of unspecified part of limb |
| cellulitis and abscess of upper arm and forearm | 6823 | L03121 | Acute lymphangitis of right axilla |
| cellulitis and abscess of upper arm and forearm | 6823 | L03122 | Acute lymphangitis of left axilla |
| cellulitis and abscess of upper arm and forearm | 6823 | L03123 | Acute lymphangitis of right upper limb |
| cellulitis and abscess of upper arm and forearm | 6823 | L03124 | Acute lymphangitis of left upper limb |
| cellulitis and abscess of upper arm and forearm | 6823 | L03129 | Acute lymphangitis of unspecified part of limb |
| cellulitis and abscess of hand, except tfingers and thing | 6824 | L02511 | Cutaneous abscess of right hand |
| cellulitis and abscess of hand, except tfingers and thing | 6824 | L02512 | Cutaneous abscess of left hand |
| **Appendix B Table-SSTI: IDC-9 and ICD-10 Codes for Skin and Soft Tissue Infections cont.** | | | |
| **ICD-9 Description** | **icd9_ssti** | **icd10_ssti** | **ICD-10 Description** |
| cellulitis and abscess of hand, except tfingers and thing | 6824 | L02519 | Cutaneous abscess of unspecified hand |
| cellulitis and abscess of hand, except tfingers and thing | 6824 | L03113 | Cellulitis of right upper limb |
| cellulitis and abscess of hand, except tfingers and thing | 6824 | L03114 | Cellulitis of left upper limb |
| cellulitis and abscess of hand, except tfingers and thing | 6824 | L03119 | Cellulitis of unspecified part of limb |
| cellulitis and abscess of hand, except tfingers and thing | 6824 | L03123 | Acute lymphangitis of right upper limb |
| cellulitis and abscess of hand, except tfingers and thing | 6824 | L03124 | Acute lymphangitis of left upper limb |
| cellulitis and abscess of hand, except tfingers and thing | 6824 | L03129 | Acute lymphangitis of unspecified part of limb |
| cellulitis and abscess of buttock | 6825 | L0231 | Cutaneous abscess of buttock |
| cellulitis and abscess of buttock | 6825 | L03317 | Cellulitis of buttock |
| cellulitis and abscess of buttock | 6825 | L03327 | Acute lymphangitis of buttock |
| cellulitis and abscess of leg, except foot | 6826 | L02415 | Cutaneous abscess of right lower limb |
| cellulitis and abscess of leg, except foot | 6826 | L02416 | Cutaneous abscess of left lower limb |
| cellulitis and abscess of leg, except foot | 6826 | L02419 | Cutaneous abscess of limb, unspecified |
| cellulitis and abscess of leg, except foot | 6826 | L03115 | Cellulitis of right lower limb |
| cellulitis and abscess of leg, except foot | 6826 | L03116 | Cellulitis of left lower limb |
| cellulitis and abscess of leg, except foot | 6826 | L03119 | Cellulitis of unspecified part of limb |
| cellulitis and abscess of leg, except foot | 6826 | L03125 | Acute lymphangitis of right lower limb |
| cellulitis and abscess of leg, except foot | 6826 | L03126 | Acute lymphangitis of left lower limb |
| cellulitis and abscess of leg, except foot | 6826 | L03129 | Acute lymphangitis of unspecified part of limb |
| cellulitis and abscess of foot, except toes | 6827 | L02611 | Cutaneous abscess of right foot |
| cellulitis and abscess of foot, except toes | 6827 | L02612 | Cutaneous abscess of left foot |
| Cellulitis and abscess of other specified sites | 6828 | L02811 | Cutaneous abscess of head [any part, except face] |
| Cellulitis and abscess of other specified sites | 6828 | L02818 | Cutaneous abscess of other sites |
| Cellulitis and abscess of other specified sites | 6828 | L03811 | Cellulitis of head [any part, except face] |
| Cellulitis and abscess of other specified sites | 6828 | L03891 | Acute lymphangitis of head [any part, except face] |
| Cellulitis and abscess of other specified sites | 6828 | L03898 | Acute lymphangitis of other sites |
| cellulitis and abscess of unspecified sites | 6829 | L0291 | Cutaneous abscess, unspecified |
| cellulitis and abscess of unspecified sites | 6829 | L0390 | Cellulitis, unspecified |
| cellulitis and abscess of unspecified sites | 6829 | L0391 | Acute lymphangitis, unspecified |
| cellulitis and abscess of unspecified sites | 6829 | L983 | Eosinophilic cellulitis [Wells] |
| **Appendix B Table-SSTI: IDC-9 and ICD-10 Codes for Skin and Soft Tissue Infections cont.** | | | |
| **ICD-9 Description** | **icd9_ssti** | **icd10_ssti** | **ICD-10 Description** |
| acute lymphadenitis | 683 | L040 | Acute lymphadenitis of face, head and neck |
| acute lymphadenitis | 683 | L041 | Acute lymphadenitis of trunk |
| acute lymphadenitis | 683 | L042 | Acute lymphadenitis of upper limb |
| acute lymphadenitis | 683 | L043 | Acute lymphadenitis of lower limb |
| acute lymphadenitis | 683 | L048 | Acute lymphadenitis of other sites |
| acute lymphadenitis | 683 | L049 | Acute lymphadenitis, unspecified |
| Impetigo | 684 | L0100 | Impetigo, unspecified |
| Impetigo | 684 | L0101 | Non-bullous impetigo |
| Impetigo | 684 | L0102 | Bockhart's impetigo |
| Impetigo | 684 | L0103 | Bullous impetigo |
| Impetigo | 684 | L0109 | Other impetigo |
| Impetigo | 684 | L011 | Impetiginization of other dermatoses |
| Pilonidal cyst with abscess | 6850 | L0501 | Pilonidal cyst with abscess |
| Pilonidal cyst with abscess | 6850 | L0502 | Pilonidal sinus with abscess |
| Pilonidal cyst without abscess | 6851 | L0591 | Pilonidal cyst without abscess |
| Pilonidal cyst without abscess | 6851 | L0592 | Pilonidal sinus without abscess |
| Pyoderma, unspecified | 68600 | L080 | Pyoderma |
| Pyoderma gangrenosum | 68601 | L88 | Pyoderma gangrenosum |
| Other pyoderma | 68609 | L0881 | Pyoderma vegetans |
| Other pyoderma | 68609 | L0889 | Other specified local infections of the skin and subcutaneous tissue |
| Pyogenic granuloma of skin and subcutaneous tissue | 6861 | L928 | Other granulomatous disorders of the skin and subcutaneous tissue |
| Pyogenic granuloma of skin and subcutaneous tissue | 6861 | L980 | Pyogenic granuloma |
| Other specified local infections of skin and subcutaneous tissue | 6868 | B781 | Cutaneous strongyloidiasis |
| Other specified local infections of skin and subcutaneous tissue | 6868 | E832 | Disorders of zinc metabolism |
| Other specified local infections of skin and subcutaneous tissue | 6868 | L0882 | Omphalitis not of newborn |
| Other specified local infections of skin and subcutaneous tissue | 6868 | L0889 | Other specified local infections of the skin and subcutaneous tissue |
| **Appendix B Table-SSTI: IDC-9 and ICD-10 Codes for Skin and Soft Tissue Infections cont.** | | | |
| **ICD-9 Description** | **icd9_ssti** | **icd10_ssti** | **ICD-10 Description** |
| Unspecified local infection of skin and subcutaneous tissue | 6869 | L089 | Local infection of the skin and subcutaneous tissue, unspecified |
| Ulcer of lower limb, unspecified convert | 70710 | L97901 | Non-pressure chronic ulcer of unspecified part of unspecified lower leg limited to breakdown of skin |
| Ulcer of lower limb, unspecified convert | 70710 | L97902 | Non-pressure chronic ulcer of unspecified part of unspecified lower leg with fat layer exposed |
| Ulcer of lower limb, unspecified convert | 70710 | L97903 | Non-pressure chronic ulcer of unspecified part of unspecified lower leg with necrosis of muscle |
| Ulcer of lower limb, unspecified convert | 70710 | L97904 | Non-pressure chronic ulcer of unspecified part of unspecified lower leg with necrosis of bone |
| Ulcer of lower limb, unspecified convert | 70710 | L97905 | Non-pressure chronic ulcer of unspecified part of unspecified lower leg with muscle involvement without evidence of necrosis |
| Ulcer of lower limb, unspecified convert | 70710 | L97906 | Non-pressure chronic ulcer of unspecified part of unspecified lower leg with bone involvement without evidence of necrosis |
| Ulcer of lower limb, unspecified convert | 70710 | L97908 | Non-pressure chronic ulcer of unspecified part of unspecified lower leg with other specified severity |
| Ulcer of lower limb, unspecified convert | 70710 | L97909 | Non-pressure chronic ulcer of unspecified part of unspecified lower leg with unspecified severity |
| Ulcer of lower limb, unspecified convert | 70710 | L97911 | Non-pressure chronic ulcer of unspecified part of right lower leg limited to breakdown of skin |
| Ulcer of lower limb, unspecified convert | 70710 | L97912 | Non-pressure chronic ulcer of unspecified part of right lower leg with fat layer exposed |
| Ulcer of lower limb, unspecified convert | 70710 | L97913 | Non-pressure chronic ulcer of unspecified part of right lower leg with necrosis of muscle |
| Ulcer of lower limb, unspecified convert | 70710 | L97914 | Non-pressure chronic ulcer of unspecified part of right lower leg with necrosis of bone |
| **Appendix B Table-SSTI: IDC-9 and ICD-10 Codes for Skin and Soft Tissue Infections cont.** | | | |
| **ICD-9 Description** | **icd9_ssti** | **icd10_ssti** | **ICD-10 Description** |
| Ulcer of lower limb, unspecified convert | 70710 | L97915 | Non-pressure chronic ulcer of unspecified part of right lower leg with muscle involvement without evidence of necrosis |
| Ulcer of lower limb, unspecified convert | 70710 | L97916 | Non-pressure chronic ulcer of unspecified part of right lower leg with bone involvement without evidence of necrosis |
| Ulcer of lower limb, unspecified convert | 70710 | L97918 | Non-pressure chronic ulcer of unspecified part of right lower leg with other specified severity |
| Ulcer of lower limb, unspecified convert | 70710 | L97919 | Non-pressure chronic ulcer of unspecified part of right lower leg with unspecified severity |
| Ulcer of lower limb, unspecified convert | 70710 | L97921 | Non-pressure chronic ulcer of unspecified part of left lower leg limited to breakdown of skin |
| Ulcer of lower limb, unspecified convert | 70710 | L97922 | Non-pressure chronic ulcer of unspecified part of left lower leg with fat layer exposed |
| Ulcer of lower limb, unspecified convert | 70710 | L97923 | Non-pressure chronic ulcer of unspecified part of left lower leg with necrosis of muscle |
| Ulcer of lower limb, unspecified convert | 70710 | L97924 | Non-pressure chronic ulcer of unspecified part of left lower leg with necrosis of bone |
| Ulcer of lower limb, unspecified convert | 70710 | L97925 | Non-pressure chronic ulcer of unspecified part of left lower leg with muscle involvement without evidence of necrosis |
| Ulcer of lower limb, unspecified convert | 70710 | L97926 | Non-pressure chronic ulcer of unspecified part of left lower leg with bone involvement without evidence of necrosis |
| Ulcer of lower limb, unspecified convert | 70710 | L97928 | Non-pressure chronic ulcer of unspecified part of left lower leg with other specified severity |
| Ulcer of lower limb, unspecified convert | 70710 | L97929 | Non-pressure chronic ulcer of unspecified part of left lower leg with unspecified severity |
| Ulcer of thigh convert | 70711 | I70231 | Atherosclerosis of native arteries of right leg with ulceration of thigh |
| **Appendix B Table-SSTI: IDC-9 and ICD-10 Codes for Skin and Soft Tissue Infections cont.** | | | |
| **ICD-9 Description** | **icd9_ssti** | **icd10_ssti** | **ICD-10 Description** |
| Ulcer of thigh convert | 70711 | I70241 | Atherosclerosis of native arteries of left leg with ulceration of thigh |
| Ulcer of thigh convert | 70711 | I70331 | Atherosclerosis of unspecified type of bypass graft(s) of the right leg with ulceration of thigh |
| Ulcer of thigh convert | 70711 | I70341 | Atherosclerosis of unspecified type of bypass graft(s) of the left leg with ulceration of thigh |
| Ulcer of thigh convert | 70711 | I70431 | Atherosclerosis of autologous vein bypass graft(s) of the right leg with ulceration of thigh |
| Ulcer of thigh convert | 70711 | I70441 | Atherosclerosis of autologous vein bypass graft(s) of the left leg with ulceration of thigh |
| Ulcer of thigh convert | 70711 | I70531 | Atherosclerosis of nonautologous biological bypass graft(s) of the right leg with ulceration of thigh |
| Ulcer of thigh convert | 70711 | I70541 | Atherosclerosis of nonautologous biological bypass graft(s) of the left leg with ulceration of thigh |
| Ulcer of thigh convert | 70711 | I70631 | Atherosclerosis of nonbiological bypass graft(s) of the right leg with ulceration of thigh |
| Ulcer of thigh convert | 70711 | I70641 | Atherosclerosis of nonbiological bypass graft(s) of the left leg with ulceration of thigh |
| Ulcer of thigh convert | 70711 | I70731 | Atherosclerosis of other type of bypass graft(s) of the right leg with ulceration of thigh |
| Ulcer of thigh convert | 70711 | I70741 | Atherosclerosis of other type of bypass graft(s) of the left leg with ulceration of thigh |
| Ulcer of thigh convert | 70711 | L97101 | Non-pressure chronic ulcer of unspecified thigh limited to breakdown of skin |
| Ulcer of thigh convert | 70711 | L97102 | Non-pressure chronic ulcer of unspecified thigh with fat layer exposed |
| Ulcer of thigh convert | 70711 | L97103 | Non-pressure chronic ulcer of unspecified thigh with necrosis of muscle |
| **Appendix B Table-SSTI: IDC-9 and ICD-10 Codes for Skin and Soft Tissue Infections cont.** | | | |
| **ICD-9 Description** | **icd9_ssti** | **icd10_ssti** | **ICD-10 Description** |
| Ulcer of thigh convert | 70711 | L97104 | Non-pressure chronic ulcer of unspecified thigh with necrosis of bone |
| Ulcer of thigh convert | 70711 | L97105 | Non-pressure chronic ulcer of unspecified thigh with muscle involvement without evidence of necrosis |
| Ulcer of thigh convert | 70711 | L97106 | Non-pressure chronic ulcer of unspecified thigh with bone involvement without evidence of necrosis |
| Ulcer of thigh convert | 70711 | L97108 | Non-pressure chronic ulcer of unspecified thigh with other specified severity |
| Ulcer of thigh convert | 70711 | L97109 | Non-pressure chronic ulcer of unspecified thigh with unspecified severity |
| Ulcer of thigh convert | 70711 | L97111 | Non-pressure chronic ulcer of right thigh limited to breakdown of skin |
| Ulcer of thigh convert | 70711 | L97112 | Non-pressure chronic ulcer of right thigh with fat layer exposed |
| Ulcer of thigh convert | 70711 | L97113 | Non-pressure chronic ulcer of right thigh with necrosis of muscle |
| Ulcer of thigh convert | 70711 | L97114 | Non-pressure chronic ulcer of right thigh with necrosis of bone |
| Ulcer of thigh convert | 70711 | L97115 | Non-pressure chronic ulcer of right thigh with muscle involvement without evidence of necrosis |
| Ulcer of thigh convert | 70711 | L97116 | Non-pressure chronic ulcer of right thigh with bone involvement without evidence of necrosis |
| Ulcer of thigh convert | 70711 | L97118 | Non-pressure chronic ulcer of right thigh with other specified severity |
| Ulcer of thigh convert | 70711 | L97119 | Non-pressure chronic ulcer of right thigh with unspecified severity |
| Ulcer of thigh convert | 70711 | L97121 | Non-pressure chronic ulcer of left thigh limited to breakdown of skin |
| **Appendix B Table-SSTI: IDC-9 and ICD-10 Codes for Skin and Soft Tissue Infections cont.** | | | |
| **ICD-9 Description** | **icd9_ssti** | **icd10_ssti** | **ICD-10 Description** |
| Ulcer of thigh convert | 70711 | L97122 | Non-pressure chronic ulcer of left thigh with fat layer exposed |
| Ulcer of thigh convert | 70711 | L97123 | Non-pressure chronic ulcer of left thigh with necrosis of muscle |
| Ulcer of thigh convert | 70711 | L97124 | Non-pressure chronic ulcer of left thigh with necrosis of bone |
| Ulcer of thigh convert | 70711 | L97125 | Non-pressure chronic ulcer of left thigh with muscle involvement without evidence of necrosis |
| Ulcer of thigh convert | 70711 | L97126 | Non-pressure chronic ulcer of left thigh with bone involvement without evidence of necrosis |
| Ulcer of thigh convert | 70711 | L97126 | Non-pressure chronic ulcer of left thigh with bone involvement without evidence of necrosis |
| Ulcer of thigh convert | 70711 | L97128 | Non-pressure chronic ulcer of left thigh with other specified severity |
| Ulcer of thigh convert | 70711 | L97129 | Non-pressure chronic ulcer of left thigh with unspecified severity |
| Ulcer of calf convert | 70712 | I70232 | Atherosclerosis of native arteries of right leg with ulceration of calf |
| Ulcer of calf convert | 70712 | I70242 | Atherosclerosis of native arteries of left leg with ulceration of calf |
| Ulcer of calf convert | 70712 | I70332 | Atherosclerosis of unspecified type of bypass graft(s) of the right leg with ulceration of calf |
| Ulcer of calf convert | 70712 | I70432 | Atherosclerosis of autologous vein bypass graft(s) of the right leg with ulceration of calf |
| Ulcer of calf convert | 70712 | I70442 | Atherosclerosis of autologous vein bypass graft(s) of the left leg with ulceration of calf |
| Ulcer of calf convert | 70712 | I70532 | Atherosclerosis of nonautologous biological bypass graft(s) of the right leg with ulceration of calf |
| **Appendix B Table-SSTI: IDC-9 and ICD-10 Codes for Skin and Soft Tissue Infections cont.** | | | |
| **ICD-9 Description** | **icd9_ssti** | **icd10_ssti** | **ICD-10 Description** |
| Ulcer of calf convert | 70712 | I70542 | Atherosclerosis of nonautologous biological bypass graft(s) of the left leg with ulceration of calf |
| Ulcer of calf convert | 70712 | I70632 | Atherosclerosis of nonbiological bypass graft(s) of the right leg with ulceration of calf |
| Ulcer of calf convert | 70712 | I70642 | Atherosclerosis of nonbiological bypass graft(s) of the left leg with ulceration of calf |
| Ulcer of calf convert | 70712 | I70732 | Atherosclerosis of other type of bypass graft(s) of the right leg with ulceration of calf |
| Ulcer of calf convert | 70712 | I70742 | Atherosclerosis of other type of bypass graft(s) of the left leg with ulceration of calf |
| Ulcer of calf convert | 70712 | L97201 | Non-pressure chronic ulcer of unspecified calf limited to breakdown of skin |
| Ulcer of calf convert | 70712 | L97202 | Non-pressure chronic ulcer of unspecified calf with fat layer exposed |
| Ulcer of calf convert | 70712 | L97203 | Non-pressure chronic ulcer of unspecified calf with necrosis of muscle |
| Ulcer of calf convert | 70712 | L97204 | Non-pressure chronic ulcer of unspecified calf with necrosis of bone |
| Ulcer of calf convert | 70712 | L97205 | Non-pressure chronic ulcer of unspecified calf with muscle involvement without evidence of necrosis |
| Ulcer of calf convert | 70712 | L97206 | Non-pressure chronic ulcer of unspecified calf with bone involvement without evidence of necrosis |
| Ulcer of calf convert | 70712 | L97208 | Non-pressure chronic ulcer of unspecified calf with other specified severity |
| Ulcer of calf convert | 70712 | L97209 | Non-pressure chronic ulcer of unspecified calf with unspecified severity |
| Ulcer of calf convert | 70712 | L97211 | Non-pressure chronic ulcer of right calf limited to breakdown of skin |
| **Appendix B Table-SSTI: IDC-9 and ICD-10 Codes for Skin and Soft Tissue Infections cont.** | | | |
| **ICD-9 Description** | **icd9_ssti** | **icd10_ssti** | **ICD-10 Description** |
| Ulcer of calf convert | 70712 | L97212 | Non-pressure chronic ulcer of right calf with fat layer exposed |
| Ulcer of calf convert | 70712 | L97213 | Non-pressure chronic ulcer of right calf with necrosis of muscle |
| Ulcer of calf convert | 70712 | L97214 | Non-pressure chronic ulcer of right calf with necrosis of bone |
| Ulcer of calf convert | 70712 | L97215 | Non-pressure chronic ulcer of right calf with muscle involvement without evidence of necrosis |
| Ulcer of calf convert | 70712 | L97216 | Non-pressure chronic ulcer of right calf with bone involvement without evidence of necrosis |
| Ulcer of calf convert | 70712 | L97218 | Non-pressure chronic ulcer of right calf with other specified severity |
| Ulcer of calf convert | 70712 | L97219 | Non-pressure chronic ulcer of right calf with unspecified severity |
| Ulcer of calf convert | 70712 | L97221 | Non-pressure chronic ulcer of left calf limited to breakdown of skin |
| Ulcer of calf convert | 70712 | L97222 | Non-pressure chronic ulcer of left calf with fat layer exposed |
| Ulcer of calf convert | 70712 | L97223 | Non-pressure chronic ulcer of left calf with necrosis of muscle |
| Ulcer of calf convert | 70712 | L97224 | Non-pressure chronic ulcer of left calf with necrosis of bone |
| Ulcer of calf convert | 70712 | L97225 | Non-pressure chronic ulcer of left calf with muscle involvement without evidence of necrosis |
| Ulcer of calf convert | 70712 | L97226 | Non-pressure chronic ulcer of left calf with bone involvement without evidence of necrosis |
| Ulcer of calf convert | 70712 | L97228 | Non-pressure chronic ulcer of left calf with other specified severity |
| **Appendix B Table-SSTI: IDC-9 and ICD-10 Codes for Skin and Soft Tissue Infections cont.** | | | |
| **ICD-9 Description** | **icd9_ssti** | **icd10_ssti** | **ICD-10 Description** |
| Ulcer of calf convert | 70712 | L97229 | Non-pressure chronic ulcer of left calf with unspecified severity |
| Ulcer of ankle convert | 70713 | I70233 | Atherosclerosis of native arteries of right leg with ulceration of ankle |
| Ulcer of ankle convert | 70713 | I70243 | Atherosclerosis of native arteries of left leg with ulceration of ankle |
| Ulcer of ankle convert | 70713 | I70333 | Atherosclerosis of unspecified type of bypass graft(s) of the right leg with ulceration of ankle |
| Ulcer of ankle convert | 70713 | I70343 | Atherosclerosis of unspecified type of bypass graft(s) of the left leg with ulceration of ankle |
| Ulcer of ankle convert | 70713 | I70433 | Atherosclerosis of autologous vein bypass graft(s) of the right leg with ulceration of ankle |
| Ulcer of ankle convert | 70713 | I70443 | Atherosclerosis of autologous vein bypass graft(s) of the left leg with ulceration of ankle |
| Ulcer of ankle convert | 70713 | I70533 | Atherosclerosis of nonautologous biological bypass graft(s) of the right leg with ulceration of ankle |
| Ulcer of ankle convert | 70713 | I70543 | Atherosclerosis of nonautologous biological bypass graft(s) of the left leg with ulceration of ankle |
| Ulcer of ankle convert | 70713 | I70633 | Atherosclerosis of nonbiological bypass graft(s) of the right leg with ulceration of ankle |
| Ulcer of ankle convert | 70713 | I70643 | Atherosclerosis of nonbiological bypass graft(s) of the left leg with ulceration of ankle |
| Ulcer of ankle convert | 70713 | I70733 | Atherosclerosis of other type of bypass graft(s) of the right leg with ulceration of ankle |
| Ulcer of ankle convert | 70713 | I70743 | Atherosclerosis of other type of bypass graft(s) of the left leg with ulceration of ankle |
|  | | | |
| **Appendix B Table-SSTI: IDC-9 and ICD-10 Codes for Skin and Soft Tissue Infections cont.** | | | |
| **ICD-9 Description** | **icd9_ssti** | **icd10_ssti** | **ICD-10 Description** |
| Ulcer of ankle convert | 70713 | L97301 | Non-pressure chronic ulcer of unspecified ankle limited to breakdown of skin |
| Ulcer of ankle convert | 70713 | L97302 | Non-pressure chronic ulcer of unspecified ankle with fat layer exposed |
| Ulcer of ankle convert | 70713 | L97303 | Non-pressure chronic ulcer of unspecified ankle with necrosis of muscle |
| Ulcer of ankle convert | 70713 | L97304 | Non-pressure chronic ulcer of unspecified ankle with necrosis of bone |
| Ulcer of ankle convert | 70713 | L97305 | Non-pressure chronic ulcer of unspecified ankle with muscle involvement without evidence of necrosis |
| Ulcer of ankle convert | 70713 | L97306 | Non-pressure chronic ulcer of unspecified ankle with bone involvement without evidence of necrosis |
| Ulcer of ankle convert | 70713 | L97308 | Non-pressure chronic ulcer of unspecified ankle with other specified severity |
| Ulcer of ankle convert | 70713 | L97309 | Non-pressure chronic ulcer of unspecified ankle with unspecified severity |
| Ulcer of ankle convert | 70713 | L97311 | Non-pressure chronic ulcer of right ankle limited to breakdown of skin |
| Ulcer of ankle convert | 70713 | L97312 | Non-pressure chronic ulcer of right ankle with fat layer exposed |
| Ulcer of ankle convert | 70713 | L97313 | Non-pressure chronic ulcer of right ankle with necrosis of muscle |
| Ulcer of ankle convert | 70713 | I70233 | Atherosclerosis of native arteries of right leg with ulceration of ankle |
| Ulcer of ankle convert | 70713 | I70243 | Atherosclerosis of native arteries of left leg with ulceration of ankle |
| Ulcer of ankle convert | 70713 | I70443 | Atherosclerosis of autologous vein bypass graft(s) of the left leg with ulceration of ankle |
| **Appendix B Table-SSTI: IDC-9 and ICD-10 Codes for Skin and Soft Tissue Infections cont.** | | | |
| **ICD-9 Description** | **icd9_ssti** | **icd10_ssti** | **ICD-10 Description** |
| Ulcer of ankle convert | 70713 | I70533 | Atherosclerosis of nonautologous biological bypass graft(s) of the right leg with ulceration of ankle |
| Ulcer of ankle convert | 70713 | I70543 | Atherosclerosis of nonautologous biological bypass graft(s) of the left leg with ulceration of ankle |
| Ulcer of ankle convert | 70713 | I70633 | Atherosclerosis of nonbiological bypass graft(s) of the right leg with ulceration of ankle |
| Ulcer of ankle convert | 70713 | I70643 | Atherosclerosis of nonbiological bypass graft(s) of the left leg with ulceration of ankle |
| Ulcer of ankle convert | 70713 | I70733 | Atherosclerosis of other type of bypass graft(s) of the right leg with ulceration of ankle |
| Ulcer of ankle convert | 70713 | I70743 | Atherosclerosis of other type of bypass graft(s) of the left leg with ulceration of ankle |
| Ulcer of ankle convert | 70713 | L97301 | Non-pressure chronic ulcer of unspecified ankle limited to breakdown of skin |
| Ulcer of ankle convert | 70713 | L97302 | Non-pressure chronic ulcer of unspecified ankle with fat layer exposed |
| Ulcer of ankle convert | 70713 | L97303 | Non-pressure chronic ulcer of unspecified ankle with necrosis of muscle |
| Ulcer of ankle convert | 70713 | L97304 | Non-pressure chronic ulcer of unspecified ankle with necrosis of bone |
| Ulcer of ankle convert | 70713 | L97305 | Non-pressure chronic ulcer of unspecified ankle with muscle involvement without evidence of necrosis |
| Ulcer of ankle convert | 70713 | L97306 | Non-pressure chronic ulcer of unspecified ankle with bone involvement without evidence of necrosis |
| Ulcer of ankle convert | 70713 | L97308 | Non-pressure chronic ulcer of unspecified ankle with other specified severity |
| Ulcer of ankle convert | 70713 | L97309 | Non-pressure chronic ulcer of unspecified ankle with unspecified severity |
| **Appendix B Table-SSTI: IDC-9 and ICD-10 Codes for Skin and Soft Tissue Infections cont.** | | | |
| **ICD-9 Description** | **icd9_ssti** | **icd10_ssti** | **ICD-10 Description** |
| Ulcer of ankle convert | 70713 | L97311 | Non-pressure chronic ulcer of right ankle limited to breakdown of skin |
| Ulcer of ankle convert | 70713 | L97312 | Non-pressure chronic ulcer of right ankle with fat layer exposed |
| Ulcer of ankle convert | 70713 | L97313 | Non-pressure chronic ulcer of right ankle with necrosis of muscle |
| Ulcer of ankle convert | 70713 | L97314 | Non-pressure chronic ulcer of right ankle with necrosis of bone |
| Ulcer of ankle convert | 70713 | L97315 | Non-pressure chronic ulcer of right ankle with muscle involvement without evidence of necrosis |
| Ulcer of ankle convert | 70713 | L97316 | Non-pressure chronic ulcer of right ankle with bone involvement without evidence of necrosis |
| Ulcer of ankle convert | 70713 | L97318 | Non-pressure chronic ulcer of right ankle with other specified severity |
| Ulcer of ankle convert | 70713 | L97319 | Non-pressure chronic ulcer of right ankle with unspecified severity |
| Ulcer of ankle convert | 70713 | L97321 | Non-pressure chronic ulcer of left ankle limited to breakdown of skin |
| Ulcer of ankle convert | 70713 | L97322 | Non-pressure chronic ulcer of left ankle with fat layer exposed |
| Ulcer of ankle convert | 70713 | L97323 | Non-pressure chronic ulcer of left ankle with necrosis of muscle |
| Ulcer of ankle convert | 70713 | L97324 | Non-pressure chronic ulcer of left ankle with necrosis of bone |
| Ulcer of ankle convert | 70713 | L97325 | Non-pressure chronic ulcer of left ankle with muscle involvement without evidence of necrosis |
| Ulcer of ankle convert | 70713 | L97326 | Non-pressure chronic ulcer of left ankle with bone involvement without evidence of necrosis |
| **Appendix B Table-SSTI: IDC-9 and ICD-10 Codes for Skin and Soft Tissue Infections cont.** | | | |
| **ICD-9 Description** | **icd9_ssti** | **icd10_ssti** | **ICD-10 Description** |
| Ulcer of ankle convert | 70713 | L97328 | Non-pressure chronic ulcer of left ankle with other specified severity |
| Ulcer of ankle convert | 70713 | L97329 | Non-pressure chronic ulcer of left ankle with unspecified severity |
| Ulcer of other part of foot convert | 70715 | I70235 | Atherosclerosis of native arteries of right leg with ulceration of other part of foot |
| Ulcer of other part of foot convert | 70715 | I70245 | Atherosclerosis of native arteries of left leg with ulceration of other part of foot |
| Ulcer of other part of foot convert | 70715 | I70335 | Atherosclerosis of unspecified type of bypass graft(s) of the right leg with ulceration of other part of foot |
| Ulcer of other part of foot convert | 70715 | I70345 | Atherosclerosis of unspecified type of bypass graft(s) of the left leg with ulceration of other part of foot |
| Ulcer of other part of foot convert | 70715 | I70435 | Atherosclerosis of autologous vein bypass graft(s) of the right leg with ulceration of other part of foot |
| Ulcer of other part of foot convert | 70715 | I70445 | Atherosclerosis of autologous vein bypass graft(s) of the left leg with ulceration of other part of foot |
| Ulcer of other part of foot convert | 70715 | I70535 | Atherosclerosis of nonautologous biological bypass graft(s) of the right leg with ulceration of other part of foot |
| Ulcer of other part of foot convert | 70715 | I70545 | Atherosclerosis of nonautologous biological bypass graft(s) of the left leg with ulceration of other part of foot |
| Ulcer of other part of foot convert | 70715 | I70635 | Atherosclerosis of nonbiological bypass graft(s) of the right leg with ulceration of other part of foot |
| Ulcer of other part of foot convert | 70715 | I70645 | Atherosclerosis of nonbiological bypass graft(s) of the left leg with ulceration of other part of foot |
| Ulcer of other part of foot convert | 70715 | I70735 | Atherosclerosis of other type of bypass graft(s) of the right leg with ulceration of other part of foot |
| **Appendix B Table-SSTI: IDC-9 and ICD-10 Codes for Skin and Soft Tissue Infections cont.** | | | |
| **ICD-9 Description** | **icd9_ssti** | **icd10_ssti** | **ICD-10 Description** |
| Ulcer of other part of foot convert | 70715 | I70745 | Atherosclerosis of other type of bypass graft(s) of the left leg with ulceration of other part of foot |
| Ulcer of other part of foot convert | 70715 | L97501 | Non-pressure chronic ulcer of other part of unspecified foot limited to breakdown of skin |
| Ulcer of other part of foot convert | 70715 | L97502 | Non-pressure chronic ulcer of other part of unspecified foot with fat layer exposed |
| Ulcer of other part of foot convert | 70715 | L97503 | Non-pressure chronic ulcer of other part of unspecified foot with necrosis of muscle |
| Ulcer of other part of foot convert | 70715 | L97504 | Non-pressure chronic ulcer of other part of unspecified foot with necrosis of bone |
| Ulcer of other part of foot convert | 70715 | L97505 | Non-pressure chronic ulcer of other part of unspecified foot with muscle involvement without evidence of necrosis |
| Ulcer of other part of foot convert | 70715 | L97506 | Non-pressure chronic ulcer of other part of unspecified foot with bone involvement without evidence of necrosis |
| Ulcer of other part of foot convert | 70715 | L97508 | Non-pressure chronic ulcer of other part of unspecified foot with other specified severity |
| Ulcer of other part of foot convert | 70715 | L97509 | Non-pressure chronic ulcer of other part of unspecified foot with unspecified severity |
| Ulcer of other part of foot convert | 70715 | L97511 | Non-pressure chronic ulcer of other part of right foot limited to breakdown of skin |
| Ulcer of other part of foot convert | 70715 | L97512 | Non-pressure chronic ulcer of other part of right foot with fat layer exposed |
| Ulcer of other part of foot convert | 70715 | L97513 | Non-pressure chronic ulcer of other part of right foot with necrosis of muscle |
| Ulcer of other part of foot convert | 70715 | L97514 | Non-pressure chronic ulcer of other part of right foot with necrosis of bone |
| **Appendix B Table-SSTI: IDC-9 and ICD-10 Codes for Skin and Soft Tissue Infections cont.** | | | |
| **ICD-9 Description** | **icd9_ssti** | **icd10_ssti** | **ICD-10 Description** |
| Ulcer of other part of foot convert | 70715 | L97515 | Non-pressure chronic ulcer of other part of right foot with muscle involvement without evidence of necrosis |
| Ulcer of other part of foot convert | 70715 | L97516 | Non-pressure chronic ulcer of other part of right foot with bone involvement without evidence of necrosis |
| Ulcer of other part of foot convert | 70715 | L97518 | Non-pressure chronic ulcer of other part of right foot with other specified severity |
| Ulcer of other part of foot convert | 70715 | L97519 | Non-pressure chronic ulcer of other part of right foot with unspecified severity |
| Ulcer of other part of foot convert | 70715 | L97521 | Non-pressure chronic ulcer of other part of left foot limited to breakdown of skin |
| Ulcer of other part of foot convert | 70715 | L97522 | Non-pressure chronic ulcer of other part of left foot with fat layer exposed |
| Ulcer of other part of foot convert | 70715 | L97523 | Non-pressure chronic ulcer of other part of left foot with necrosis of muscle |
| Ulcer of other part of foot convert | 70715 | L97524 | Non-pressure chronic ulcer of other part of left foot with necrosis of bone |
| Ulcer of other part of foot convert | 70715 | L97525 | Non-pressure chronic ulcer of other part of left foot with muscle involvement without evidence of necrosis |
| Ulcer of other part of foot convert | 70715 | L97526 | Non-pressure chronic ulcer of other part of left foot with bone involvement without evidence of necrosis |
| Ulcer of other part of foot convert | 70715 | L97528 | Non-pressure chronic ulcer of other part of left foot with other specified severity |
| Ulcer of other part of foot convert | 70715 | L97529 | Non-pressure chronic ulcer of other part of left foot with unspecified severity |
| Ulcer of other part of lower limb convert | 70719 | I70238 | Atherosclerosis of native arteries of right leg with ulceration of other part of lower right leg |
| Ulcer of other part of lower limb convert | 70719 | I70239 | Atherosclerosis of native arteries of right leg with ulceration of unspecified site |
| **Appendix B Table-SSTI: IDC-9 and ICD-10 Codes for Skin and Soft Tissue Infections cont.** | | | |
| **ICD-9 Description** | **icd9_ssti** | **icd10_ssti** | **ICD-10 Description** |
| Ulcer of other part of lower limb convert | 70719 | I70248 | Atherosclerosis of native arteries of left leg with ulceration of other part of lower left leg |
| Ulcer of other part of lower limb convert | 70719 | I70249 | Atherosclerosis of native arteries of left leg with ulceration of unspecified site |
| Ulcer of other part of lower limb convert | 70719 | I70338 | Atherosclerosis of unspecified type of bypass graft(s) of the right leg with ulceration of other part of lower leg |
| Ulcer of other part of lower limb convert | 70719 | I70339 | Atherosclerosis of unspecified type of bypass graft(s) of the right leg with ulceration of unspecified site |
| Ulcer of other part of lower limb convert | 70719 | I70348 | Atherosclerosis of unspecified type of bypass graft(s) of the left leg with ulceration of other part of lower leg |
| Ulcer of other part of lower limb convert | 70719 | I70349 | Atherosclerosis of unspecified type of bypass graft(s) of the left leg with ulceration of unspecified site |
| Ulcer of other part of lower limb convert | 70719 | I70438 | Atherosclerosis of autologous vein bypass graft(s) of the right leg with ulceration of other part of lower leg |
| Ulcer of other part of lower limb convert | 70719 | I70439 | Atherosclerosis of autologous vein bypass graft(s) of the right leg with ulceration of unspecified site |
| Ulcer of other part of lower limb convert | 70719 | I70448 | Atherosclerosis of autologous vein bypass graft(s) of the left leg with ulceration of other part of lower leg |
| Ulcer of other part of lower limb convert | 70719 | I70449 | Atherosclerosis of autologous vein bypass graft(s) of the left leg with ulceration of unspecified site |
| Ulcer of other part of lower limb convert | 70719 | I70538 | Atherosclerosis of nonautologous biological bypass graft(s) of the right leg with ulceration of other part of lower leg |
| Ulcer of other part of lower limb convert | 70719 | I70539 | Atherosclerosis of nonautologous biological bypass graft(s) of the right leg with ulceration of unspecified site |
| Ulcer of other part of lower limb convert | 70719 | I70548 | Atherosclerosis of nonautologous biological bypass graft(s) of the left leg with ulceration of other part of lower leg |
| **Appendix B Table-SSTI: IDC-9 and ICD-10 Codes for Skin and Soft Tissue Infections cont.** | | | |
| **ICD-9 Description** | **icd9_ssti** | **icd10_ssti** | **ICD-10 Description** |
| Ulcer of other part of lower limb convert | 70719 | I70549 | Atherosclerosis of nonautologous biological bypass graft(s) of the left leg with ulceration of unspecified site |
| Ulcer of other part of lower limb convert | 70719 | I70638 | Atherosclerosis of nonbiological bypass graft(s) of the right leg with ulceration of other part of lower leg |
| Ulcer of other part of lower limb convert | 70719 | I70639 | Atherosclerosis of nonbiological bypass graft(s) of the right leg with ulceration of unspecified site |
| Ulcer of other part of lower limb convert | 70719 | I70648 | Atherosclerosis of nonbiological bypass graft(s) of the left leg with ulceration of other part of lower leg |
| Ulcer of other part of lower limb convert | 70719 | I70738 | Atherosclerosis of other type of bypass graft(s) of the right leg with ulceration of other part of lower leg |
| Ulcer of other part of lower limb convert | 70719 | I70739 | Atherosclerosis of other type of bypass graft(s) of the right leg with ulceration of unspecified site |
| Ulcer of other part of lower limb convert | 70719 | I70748 | Atherosclerosis of other type of bypass graft(s) of the left leg with ulceration of other part of lower leg |
| Ulcer of other part of lower limb convert | 70719 | I70749 | Atherosclerosis of other type of bypass graft(s) of the left leg with ulceration of unspecified site |
| Ulcer of other part of lower limb convert | 70719 | L97801 | Non-pressure chronic ulcer of other part of unspecified lower leg limited to breakdown of skin |
| Ulcer of other part of lower limb convert | 70719 | L97802 | Non-pressure chronic ulcer of other part of unspecified lower leg with fat layer exposed |
| Ulcer of other part of lower limb convert | 70719 | L97803 | Non-pressure chronic ulcer of other part of unspecified lower leg with necrosis of muscle |
| Ulcer of other part of lower limb convert | 70719 | L97804 | Non-pressure chronic ulcer of other part of unspecified lower leg with necrosis of bone |
| Ulcer of other part of lower limb convert | 70719 | L97805 | Non-pressure chronic ulcer of other part of unspecified lower leg with muscle involvement without evidence of necrosis |
|  | | | |
| **Appendix B Table-SSTI: IDC-9 and ICD-10 Codes for Skin and Soft Tissue Infections cont.** | | | |
| **ICD-9 Description** | **icd9_ssti** | **icd10_ssti** | **ICD-10 Description** |
| Ulcer of other part of lower limb convert | 70719 | L97806 | Non-pressure chronic ulcer of other part of unspecified lower leg with bone involvement without evidence of necrosis |
| Ulcer of other part of lower limb convert | 70719 | L97808 | Non-pressure chronic ulcer of other part of unspecified lower leg with other specified severity |
| Ulcer of other part of lower limb convert | 70719 | L97809 | Non-pressure chronic ulcer of other part of unspecified lower leg with unspecified severity |
| Ulcer of other part of lower limb convert | 70719 | L97811 | Non-pressure chronic ulcer of other part of right lower leg limited to breakdown of skin |
| Ulcer of other part of lower limb convert | 70719 | L97812 | Non-pressure chronic ulcer of other part of right lower leg with fat layer exposed |
| Ulcer of other part of lower limb convert | 70719 | L97813 | Non-pressure chronic ulcer of other part of right lower leg with necrosis of muscle |
| Ulcer of other part of lower limb convert | 70719 | L97814 | Non-pressure chronic ulcer of other part of right lower leg with necrosis of bone |
| Ulcer of other part of lower limb convert | 70719 | L97815 | Non-pressure chronic ulcer of other part of right lower leg with muscle involvement without evidence of necrosis |
| Ulcer of other part of lower limb convert | 70719 | L97816 | Non-pressure chronic ulcer of other part of right lower leg with bone involvement without evidence of necrosis |
| Ulcer of other part of lower limb convert | 70719 | L97818 | Non-pressure chronic ulcer of other part of right lower leg with other specified severity |
| Ulcer of other part of lower limb convert | 70719 | L97819 | Non-pressure chronic ulcer of other part of right lower leg with unspecified severity |
| Ulcer of other part of lower limb convert | 70719 | L97821 | Non-pressure chronic ulcer of other part of left lower leg limited to breakdown of skin |
| Ulcer of other part of lower limb convert | 70719 | L97822 | Non-pressure chronic ulcer of other part of left lower leg with fat layer exposed |
| **Appendix B Table-SSTI: IDC-9 and ICD-10 Codes for Skin and Soft Tissue Infections cont.** | | | |
| **ICD-9 Description** | **icd9_ssti** | **icd10_ssti** | **ICD-10 Description** |
| Ulcer of other part of lower limb convert | 70719 | L97823 | Non-pressure chronic ulcer of other part of left lower leg with necrosis of muscle |
| Ulcer of other part of lower limb convert | 70719 | L97824 | Non-pressure chronic ulcer of other part of left lower leg with necrosis of bone |
| Ulcer of other part of lower limb convert | 70719 | L97825 | Non-pressure chronic ulcer of other part of left lower leg with muscle involvement without evidence of necrosis |
| Ulcer of other part of lower limb convert | 70719 | L97826 | Non-pressure chronic ulcer of other part of left lower leg with bone involvement without evidence of necrosis |
| Ulcer of other part of lower limb convert | 70719 | L97828 | Non-pressure chronic ulcer of other part of left lower leg with other specified severity |
| Ulcer of other part of lower limb convert | 70719 | L97829 | Non-pressure chronic ulcer of other part of left lower leg with unspecified severity |
| Panniculitis specified as affecting the neck | 7236 | M5400 | Panniculitis affecting regions of neck and back, site unspecified |
| Panniculitis specified as affecting the neck | 7236 | M5401 | Panniculitis affecting regions of neck and back, occipito-atlanto-axial region |
| Panniculitis specified as affecting the neck | 7236 | M5402 | Panniculitis affecting regions of neck and back, cervical region |
| Infective myositis | 7280 | M60000 | Infective myositis, unspecified right arm |
| Infective myositis | 7280 | M60001 | Infective myositis, unspecified left arm |
| Infective myositis | 7280 | M60002 | Infective myositis, unspecified arm |
| Infective myositis | 7280 | M60003 | Infective myositis, unspecified right leg |
| Infective myositis | 7280 | M60004 | Infective myositis, unspecified left leg |
| Infective myositis | 7280 | M60005 | Infective myositis, unspecified leg |
| Infective myositis | 7280 | M60009 | Infective myositis, unspecified site |
| Infective myositis | 7280 | M60011 | Infective myositis, right shoulder |
| Infective myositis | 7280 | M60012 | Infective myositis, left shoulder |
| Infective myositis | 7280 | M60019 | Infective myositis, unspecified shoulder |
| Infective myositis | 7280 | M60021 | Infective myositis, right upper arm |
| **Appendix B Table-SSTI: IDC-9 and ICD-10 Codes for Skin and Soft Tissue Infections cont.** | | | |
| **ICD-9 Description** | **icd9_ssti** | **icd10_ssti** | **ICD-10 Description** |
| Infective myositis | 7280 | M60022 | Infective myositis, left upper arm |
| Infective myositis | 7280 | M60029 | Infective myositis, unspecified upper arm |
| Infective myositis | 7280 | M60031 | Infective myositis, right forearm |
| Infective myositis | 7280 | M60032 | Infective myositis, left forearm |
| Infective myositis | 7280 | M60039 | Infective myositis, unspecified forearm |
| Infective myositis | 7280 | M60041 | Infective myositis, right hand |
| Infective myositis | 7280 | M60042 | Infective myositis, left hand |
| Infective myositis | 7280 | M60043 | Infective myositis, unspecified hand |
| Infective myositis | 7280 | M60044 | Infective myositis, right finger(s) |
| Infective myositis | 7280 | M60045 | ective myositis, left finger(s) |
| Infective myositis | 7280 | M60046 | Infective myositis, unspecified finger(s) |
| Infective myositis | 7280 | M60051 | Infective myositis, right thigh |
| Infective myositis | 7280 | M60052 | Infective myositis, left thigh |
| Infective myositis | 7280 | M60059 | Infective myositis, unspecified thigh |
| Infective myositis | 7280 | M60061 | Infective myositis, right lower leg |
| Infective myositis | 7280 | M60062 | Infective myositis, left lower leg |
| Infective myositis | 7280 | M60069 | Infective myositis, unspecified lower leg |
| Infective myositis | 7280 | M60070 | Infective myositis, right ankle |
| Infective myositis | 7280 | M60071 | Infective myositis, left ankle |
| Infective myositis | 7280 | M60072 | Infective myositis, unspecified ankle |
| Infective myositis | 7280 | M60073 | Infective myositis, right foot |
| Infective myositis | 7280 | M60074 | Infective myositis, left foot |
| Infective myositis | 7280 | M60075 | Infective myositis, unspecified foot |
| Infective myositis | 7280 | M60076 | Infective myositis, right toe(s) |
| Infective myositis | 7280 | M60077 | Infective myositis, left toe(s) |
| Infective myositis | 7280 | M60078 | Infective myositis, unspecified toe(s) |
| Infective myositis | 7280 | M6008 | Infective myositis, other site |
| Infective myositis | 7280 | M6009 | Infective myositis, multiple sites |
| necrotizing fasciitis | 72886 | M726 | Necrotizing fasciitis |
| Panniculitis, unspecified site | 72930 | M356 | Relapsing panniculitis [Weber-Christian] |
| Panniculitis, unspecified site | 72930 | M793 | Panniculitis, unspecified |
| **Appendix B Table-SSTI: IDC-9 and ICD-10 Codes for Skin and Soft Tissue Infections cont.** | | | |
| **ICD-9 Description** | **icd9_ssti** | **icd10_ssti** | **ICD-10 Description** |
| gangrene | 7854 | E0852 | Diabetes mellitus due to underlying condition with diabetic peripheral angiopathy with gangrene |
| gangrene | 7854 | E0952 | Drug or chemical induced diabetes mellitus with diabetic peripheral angiopathy with gangrene |
| gangrene | 7854 | E1052 | Type 1 diabetes mellitus with diabetic peripheral angiopathy with gangrene |
| gangrene | 7854 | E1152 | Type 2 diabetes mellitus with diabetic peripheral angiopathy with gangrene |
| gangrene | 7854 | E1352 | Other specified diabetes mellitus with diabetic peripheral angiopathy with gangrene |
| gangrene | 7854 | I70361 | Atherosclerosis of unspecified type of bypass graft(s) of the extremities with gangrene, right leg |
| gangrene | 7854 | I70362 | Atherosclerosis of unspecified type of bypass graft(s) of the extremities with gangrene, left leg |
| gangrene | 7854 | I70363 | Atherosclerosis of unspecified type of bypass graft(s) of the extremities with gangrene, bilateral legs |
| gangrene | 7854 | I70368 | Atherosclerosis of unspecified type of bypass graft(s) of the extremities with gangrene, other extremity |
| gangrene | 7854 | I70369 | Atherosclerosis of unspecified type of bypass graft(s) of the extremities with gangrene, unspecified extremity |
| gangrene | 7854 | I70461 | Atherosclerosis of autologous vein bypass graft(s) of the extremities with gangrene, right leg |
| gangrene | 7854 | I70462 | Atherosclerosis of autologous vein bypass graft(s) of the extremities with gangrene, left leg |
| gangrene | 7854 | I70463 | Atherosclerosis of autologous vein bypass graft(s) of the extremities with gangrene, bilateral legs |
| gangrene | 7854 | I70468 | Atherosclerosis of autologous vein bypass graft(s) of the extremities with gangrene, other extremity |
| **Appendix B Table-SSTI: IDC-9 and ICD-10 Codes for Skin and Soft Tissue Infections cont.** | | | |
| **ICD-9 Description** | **icd9_ssti** | **icd10_ssti** | **ICD-10 Description** |
| gangrene | 7854 | I70469 | Atherosclerosis of autologous vein bypass graft(s) of the extremities with gangrene, unspecified extremity |
| gangrene | 7854 | I70561 | Atherosclerosis of nonautologous biological bypass graft(s) of the extremities with gangrene, right leg |
| gangrene | 7854 | I70562 | Atherosclerosis of nonautologous biological bypass graft(s) of the extremities with gangrene, left leg |
| gangrene | 7854 | I70563 | Atherosclerosis of nonautologous biological bypass graft(s) of the extremities with gangrene, bilateral legs |
| gangrene | 7854 | I70568 | Atherosclerosis of nonautologous biological bypass graft(s) of the extremities with gangrene, other extremity |
| gangrene | 7854 | I70569 | Atherosclerosis of nonautologous biological bypass graft(s) of the extremities with gangrene, unspecified extremity |
| gangrene | 7854 | I70661 | Atherosclerosis of nonbiological bypass graft(s) of the extremities with gangrene, right leg |
| gangrene | 7854 | I70662 | Atherosclerosis of nonbiological bypass graft(s) of the extremities with gangrene, left leg |
| gangrene | 7854 | I70663 | Atherosclerosis of nonbiological bypass graft(s) of the extremities with gangrene, bilateral legs |
| gangrene | 7854 | I70668 | Atherosclerosis of nonbiological bypass graft(s) of the extremities with gangrene, other extremity |
| gangrene | 7854 | I70669 | Atherosclerosis of nonbiological bypass graft(s) of the extremities with gangrene, unspecified extremity |
| gangrene | 7854 | I70761 | Atherosclerosis of other type of bypass graft(s) of the extremities with gangrene, right leg |
| gangrene | 7854 | I70762 | Atherosclerosis of other type of bypass graft(s) of the extremities with gangrene, left leg |
| **Appendix B Table-SSTI: IDC-9 and ICD-10 Codes for Skin and Soft Tissue Infections cont.** | | | |
| **ICD-9 Description** | **icd9_ssti** | **icd10_ssti** | **ICD-10 Description** |
| gangrene | 7854 | I70763 | Atherosclerosis of other type of bypass graft(s) of the extremities with gangrene, bilateral legs |
| gangrene | 7854 | I70768 | Atherosclerosis of other type of bypass graft(s) of the extremities with gangrene, other extremity |
| gangrene | 7854 | I70769 | Atherosclerosis of other type of bypass graft(s) of the extremities with gangrene, unspecified extremity |
| gangrene | 7854 | I7301 | Raynaud's syndrome with gangrene |
| gangrene | 7854 | I96 | Gangrene, not elsewhere classified |

**Appendix B Table 2-SSTI: Univariate Statistics 2011 and 2016**

| **Patient Characteristics** | **SSTI - Group Comparisons 2011** | | | **SSTI - Group Comparisons 2016** | | |
| --- | --- | --- | --- | --- | --- | --- |
|  | **Cases with Secondary Dx** | **Cases with Principal DX** | **Matched Cases with No Dx** | **Cases with Secondary Dx** | **Cases with Principal DX** | **Matched Cases with No Dx** |
|  |  |  |  |  |  |  |
|  |  |  |  |  |  |  |
| **n =** | 191,787 | 134,747 | 859,036 | 198,195 | 131,301 | 915,776 |
| **Variables:** |  |  |  |  |  |  |
| **Cost** |  |  |  |  |  |  |
| mean | $21,929 | $7,675 | $14,443 | $21,519 | $8,763 | $14,483 |
| median | $12,889 | $5,304 | $8,368 | $12,580 | $5,883 | $8,548 |
| mode | $8,302 | $78 | $5,535 | $9,445 | $3,148 | $3,148 |
| range | $34 - $1,174,598 | $31 - $457,612 | $31 - $1,410,243 | $26 - $2,850,000 | $23 - $866,185 | $21 - $2,180,084 |
| **LOS** |  |  |  |  |  |  |
| mean | 9.82 | 4.40 | 5.78 | 9.43 | 4.61 | 5.64 |
| median | 7 | 3 | 4 | 6 | 3 | 4 |
| mode | 3 | 3 | 2 | 3 | 3 | 2 |
| range | 3 - 365 | 1- 311 | 1 - 365 | 3 - 363 | 1- 350 | 1 - 363 |
| **Number of Diagnoses; mean (SD)** | 14.86 (6.32) | 8.77 (5.51) | 11.20 (6.06) | 17.14 (6.54) | 11.24 (6.44) | 13.56 (6.75) |
| **Number of Procedures; mean (SD)** | 2.52 (2.97) | 0.88 (1.33) | 1.73 (2.39) | 2.49 (3.01) | 1.06 (1.78) | 1.64 (2.40) |
| **age; mean (range)** | 62 (0 - 107) | 52 (0 - 107) | 60 (0-114) | 60 (0 - 90) | 54 (0 - 90) | 60 (0-90) |
| **APRDRG_Severity; mean (SD)** | 2.87 (0.81) | 1.99 (0.84) | 2.48 (0.95) | 2.80 (0.78) | 2.01 (0.79) | 2.52 (0.93) |
| **APRDRG_Risk_Mortality; mean (SD)** | 2.36 (0.99) | 1.52 (0.76) | 2.11 (1.04) | 2.38 (0.98) | 1.60 (0.78) | 2.26 (1.07) |
| **Wage Index; mean (SD)** | 1.0053 (0.159) | 0.9991 (0.163) | 1.0011 (0.159) | 1.0026 (0.194) | 0.9995 (0.197) | 1.0017 (0.196) |

**Appendix B Table 3-SSTI: Results of Median Regression Models 2011 and 2016**

| **Median Regression Estimates for Cost of SSTI as a Principal Diagnosis (n=134662) 2011** | | | | | | | **Median Regression Estimates for Cost of SSTI as a Principal Diagnosis (n=131293) 2016** | | | | | | |
| --- | --- | --- | --- | --- | --- | --- | --- | --- | --- | --- | --- | --- | --- |
| **Parameter** | **Estimate** | **Standard Error** | **95% Confidence Limits** | | **t Value** | **Pr > \|t\|** | **Parameter** | **Estimate** | **Standard Error** | **95% Confidence Limits** | | **t Value** | **Pr > \|t\|** |
| **Intercept** | -5637.82 | 57.5840 | -5750.682 | -5524.955 | -97.91 | <.0001 | **Intercept** | -4328.99 | 44.6056 | -4416.417 | -4241.565 | -97.05 | <.0001 |
| **LOS** | 1178.226 | 5.2186 | 1167.9977 | 1188.4543 | 225.78 | <.0001 | **LOS** | 1154.976 | 7.9266 | 1139.4395 | 1170.5116 | 145.71 | <.0001 |
| **NDX** | 64.3486 | 2.5346 | 59.3807 | 69.3164 | 25.39 | <.0001 | **NDX** | 32.5963 | 1.9801 | 28.7154 | 36.4772 | 16.46 | <.0001 |
| **NPR** | 1074.838 | 12.0418 | 1051.2365 | 1098.4401 | 89.26 | <.0001 | **NPR** | 1506.030 | 13.4728 | 1479.6240 | 1532.4369 | 111.78 | <.0001 |
| **URBAN_TEACH** | 356.9400 | 13.8756 | 329.7441 | 384.1358 | 25.72 | <.0001 | **URBAN_TEACH** | 391.6719 | 15.4470 | 361.3961 | 421.9477 | 25.36 | <.0001 |
| **URBAN_TEACH** | 0.0000 | 0.0000 | 0.0000 | 0.0000 | . | . | **URBAN_TEACH** | 0.0000 | 0.0000 | 0.0000 | 0.0000 | . | . |
| **SMALL_BEDSIZE** | -589.443 | 21.0434 | -630.6876 | -548.1983 | -28.01 | <.0001 | **SMALL_BEDSIZE** | -477.405 | 16.4310 | -509.6097 | -445.2009 | -29.06 | <.0001 |
| **SMALL_BEDSIZE** | 0.0000 | 0.0000 | 0.0000 | 0.0000 | . | . | **SMALL_BEDSIZE** | 0.0000 | 0.0000 | 0.0000 | 0.0000 | . | . |
| **AGE** | -8.7239 | 0.3170 | -9.3451 | -8.1026 | -27.52 | <.0001 | **AGE** | -7.4655 | 0.4217 | -8.2921 | -6.6389 | -17.70 | <.0001 |
| **APRDRG_Severity** | 267.8497 | 13.1224 | 242.1301 | 293.5692 | 20.41 | <.0001 | **APRDRG_Severity** | 309.3366 | 15.1687 | 279.6063 | 339.0669 | 20.39 | <.0001 |
| **APRDRG_Risk_Mortality** | 151.7022 | 15.6628 | 121.0035 | 182.4009 | 9.69 | <.0001 | **APRDRG_Risk_Mortality** | 245.9449 | 17.6499 | 211.3514 | 280.5384 | 13.93 | <.0001 |
| **Wage_Index** | 5890.943 | 54.0631 | 5784.9806 | 5996.9058 | 108.96 | <.0001 | **Wage_Index** | 4633.705 | 41.8611 | 4551.6581 | 4715.7520 | 110.69 | <.0001 |

**Specified Stage 2 Cost Simulation Models for SSTI 2011 and 2016**

**Cost_ssti_2011 = -5637.82 + 1178.226*4.40113695 + 64.3486*NDX + 1074.838*NPR + 356.9400*URBAN_TEACH + (-589.443)*SMALL_BEDSIZE +**

**(-8.7239)*AGE + 267.8497*APRDRG_Severity + 151.7022*APRDRG_Risk_Mortality + 5890.943*Wage_Index**

**Cost_ssti_2016 = -4328.99+ 1154.976*4.60539524 + 32.5963*NDX + 1506.030*NPR + 391.6719*URBAN_TEACH + (-477.405)*SMALL_BEDSIZE +**

**(-7.4655)*AGE + 309.3366*APRDRG_Severity + 245.9449*APRDRG_Risk_Mortality + 4633.705*Wage_Index**

**Systemic Infections (SYSI)**

| **Appendix B Table-SysI: IDC-9 and ICD-10 Codes for Systemic Infections** | |  |  |
| --- | --- | --- | --- |
| **ICD-9 Description** | **icd9_sysi** | **icd10_sysi** | **ICD-10 Description** |
| Toxic shock syndrome | 04082 | A483 | Toxic shock syndrome |
| Other specified bacterial diseases | 04089 | A482 | Nonpneumonic Legionnaires' disease [Pontiac fever] |
| Other specified bacterial diseases | 04089 | A484 | Brazilian purpuric fever |
| Other specified bacterial diseases | 04089 | A488 | Other specified bacterial diseases |
| Streptococcus infection in conditions classified elsewhere and of unspecified site, Streptococcus, unspecified | 04100 | A491 | Streptococcal infection, unspecified site |
| Streptococcus infection in conditions classified elsewhere and of unspecified site, Streptococcus, unspecified | 04100 | B955 | Unspecified streptococcus as the cause of diseases classified  elsewhere |
| Streptococcus infection in conditions classified elsewhere and of unspecified site, Streptococcus, unspecified | 04100 | J202 | Acute bronchitis due to streptococcus |
| Streptococcus infection in conditions classified elsewhere and of unspecified site, Streptococcus group b (including sepsis of newborn) | 04102 | B951 | Streptococcus, group B, as the cause of diseases classified  elsewhere |
| Streptococcus infection in conditions classified elsewhere and of unspecified site, streptococcus, group C | 04103 | B954 | Other streptococcus as the cause of diseases classified elsewhere |
| Streptococcus infection in conditions classified elsewhere and of unspecified site, streptococcus, group d (enterococcus) | 04104 | B952 | Enterococcus as the cause of diseases classified elsewhere |
| Streptococcus infection in conditions classified elsewhere and of unspecified site, streptococcus, group G | 04105 | B954 | Other streptococcus as the cause of diseases classified elsewhere |
| Streptococcus infection in conditions classified elsewhere and of unspecified site, other streptococcus | 04109 | B953 | Streptococcus pneumoniae as the cause of diseases classified elsewhere |
| Streptococcus infection in conditions classified elsewhere and of unspecified site, other streptococcus | 04109 | B954 | Other streptococcus as the cause of diseases classified elsewhere |
| Streptococcus infection in conditions classified elsewhere and of unspecified site, other streptococcus | 04109 | M0020 | Other streptococcal arthritis, unspecified joint |
| Streptococcus infection in conditions classified elsewhere and of unspecified site, other streptococcus | 04109 | M00211 | Other streptococcal arthritis, right shoulder |
| Streptococcus infection in conditions classified elsewhere and of unspecified site, other streptococcus | 04109 | M00212 | Other streptococcal arthritis, left shoulder |
| **Appendix B Table-SysI: IDC-9 and ICD-10 Codes for Systemic Infections cont.** | | | |
| **ICD-9 Description** | **icd9_sysi** | **icd10_sysi** | **ICD-10 Description** |
| Streptococcus infection in conditions classified elsewhere and of unspecified site, other streptococcus | 04109 | M00219 | Other streptococcal arthritis, unspecified shoulder |
| Streptococcus infection in conditions classified elsewhere and of unspecified site, other streptococcus | 04109 | M00221 | Other streptococcal arthritis, right elbow |
| Streptococcus infection in conditions classified elsewhere and of unspecified site, other streptococcus | 04109 | M00222 | Other streptococcal arthritis, left elbow |
| Streptococcus infection in conditions classified elsewhere and of unspecified site, other streptococcus | 04109 | M00229 | Other streptococcal arthritis, unspecified elbow |
| Streptococcus infection in conditions classified elsewhere and of unspecified site, other streptococcus | 04109 | M00231 | Other streptococcal arthritis, right wrist |
| Streptococcus infection in conditions classified elsewhere and of unspecified site, other streptococcus | 04109 | M00232 | Other streptococcal arthritis, left wrist |
| Streptococcus infection in conditions classified elsewhere and of unspecified site, other streptococcus | 04109 | M00239 | Other streptococcal arthritis, unspecified wrist |
| Streptococcus infection in conditions classified elsewhere and of unspecified site, other streptococcus | 04109 | M00241 | Other streptococcal arthritis, right hand |
| Streptococcus infection in conditions classified elsewhere and of unspecified site, other streptococcus | 04109 | M00242 | Other streptococcal arthritis, left hand |
| Streptococcus infection in conditions classified elsewhere and of unspecified site, other streptococcus | 04109 | M00249 | Other streptococcal arthritis, unspecified hand |
| Streptococcus infection in conditions classified elsewhere and of unspecified site, other streptococcus | 04109 | M00251 | Other streptococcal arthritis, right hip |
| Streptococcus infection in conditions classified elsewhere and of unspecified site, other streptococcus | 04109 | M00252 | Other streptococcal arthritis, left hip |
| Streptococcus infection in conditions classified elsewhere and of unspecified site, other streptococcus | 04109 | M00259 | Other streptococcal arthritis, unspecified hip |
| Streptococcus infection in conditions classified elsewhere and of unspecified site, other streptococcus | 04109 | M00261 | Other streptococcal arthritis, right knee |
| Streptococcus infection in conditions classified elsewhere and of unspecified site, other streptococcus | 04109 | M00262 | Other streptococcal arthritis, left knee |

| **Appendix B Table-SysI: IDC-9 and ICD-10 Codes for Systemic Infections cont.** | | | |
| --- | --- | --- | --- |
| **ICD-9 Description** | **icd9_sysi** | **icd10_sysi** | **ICD-10 Description** |
| Streptococcus infection in conditions classified elsewhere and of unspecified site, other streptococcus | 04109 | M00269 | Other streptococcal arthritis, unspecified knee |
| Streptococcus infection in conditions classified elsewhere and of unspecified site, other streptococcus | 04109 | M00271 | Other streptococcal arthritis, right ankle and foot |
| Streptococcus infection in conditions classified elsewhere and of unspecified site, other streptococcus | 04109 | M00272 | Other streptococcal arthritis, left ankle and foot |
| Streptococcus infection in conditions classified elsewhere and of unspecified site, other streptococcus | 04109 | M00279 | Other streptococcal arthritis, unspecified ankle and foot |
| Streptococcus infection in conditions classified elsewhere and of unspecified site, other streptococcus | 04109 | M0028 | Other streptococcal arthritis, vertebrae |
| Streptococcus infection in conditions classified elsewhere and of unspecified site, other streptococcus | 04109 | M0029 | Other streptococcal polyarthritis |
| Staphylcoccus infection in conditions classified elsewhere and of unspecified site, staphylcococus, unspecified | 04110 | B958 | Unspecified staphylococcus as the cause of diseases classified elsewhere |
| Staphylcoccus infection in conditions classified elsewhere and of unspecified site, staphylcococus, unspecified | 04110 | M0000 | Staphylococcal arthritis, unspecified joint |
| Staphylcoccus infection in conditions classified elsewhere and of unspecified site, staphylcococus, unspecified | 04110 | M00011 | Staphylococcal arthritis, right shoulder |
| Staphylcoccus infection in conditions classified elsewhere and of unspecified site, staphylcococus, unspecified | 04110 | M00012 | Staphylococcal arthritis, left shoulder |
| Staphylcoccus infection in conditions classified elsewhere and of unspecified site, staphylcococus, unspecified | 04110 | M00019 | Staphylococcal arthritis, unspecified shoulder |
| Staphylcoccus infection in conditions classified elsewhere and of unspecified site, staphylcococus, unspecified | 04110 | M00021 | Staphylococcal arthritis, right elbow |
| Staphylcoccus infection in conditions classified elsewhere and of unspecified site, staphylcococus, unspecified | 04110 | M00022 | Staphylococcal arthritis, left elbow |
| Staphylcoccus infection in conditions classified elsewhere and of unspecified site, staphylcococus, unspecified | 04110 | M00029 | Staphylococcal arthritis, unspecified elbow |
| Staphylcoccus infection in conditions classified elsewhere and of unspecified site, staphylcococus, unspecified | 04110 | M00031 | Staphylococcal arthritis, right wrist |
| **Appendix B Table-SysI: IDC-9 and ICD-10 Codes for Systemic Infections cont.** | | | |
| **ICD-9 Description** | **icd9_sysi** | **icd10_sysi** | **ICD-10 Description** |
| Staphylcoccus infection in conditions classified elsewhere and of unspecified site, staphylcococus, unspecified | 04110 | M00032 | Staphylococcal arthritis, left wrist |
| Staphylcoccus infection in conditions classified elsewhere and of unspecified site, staphylcococus, unspecified | 04110 | M00039 | Staphylococcal arthritis, unspecified wrist |
| Staphylcoccus infection in conditions classified elsewhere and of unspecified site, staphylcococus, unspecified | 04110 | M00041 | Staphylococcal arthritis, right hand |
| Staphylcoccus infection in conditions classified elsewhere and of unspecified site, staphylcococus, unspecified | 04110 | M00042 | Staphylococcal arthritis, left hand |
| Staphylcoccus infection in conditions classified elsewhere and of unspecified site, staphylcococus, unspecified | 04110 | M00049 | Staphylococcal arthritis, unspecified hand |
| Staphylcoccus infection in conditions classified elsewhere and of unspecified site, staphylcococus, unspecified | 04110 | M00051 | Staphylococcal arthritis, right hip |
| Staphylcoccus infection in conditions classified elsewhere and of unspecified site, staphylcococus, unspecified | 04110 | M00052 | Staphylococcal arthritis, left hip |
| Staphylcoccus infection in conditions classified elsewhere and of unspecified site, staphylcococus, unspecified | 04110 | M00059 | Staphylococcal arthritis, unspecified hip |
| Staphylcoccus infection in conditions classified elsewhere and of unspecified site, staphylcococus, unspecified | 04110 | M00061 | Staphylococcal arthritis, right knee |
| Staphylcoccus infection in conditions classified elsewhere and of unspecified site, staphylcococus, unspecified | 04110 | M00062 | Staphylococcal arthritis, left knee |
| Staphylcoccus infection in conditions classified elsewhere and of unspecified site, staphylcococus, unspecified | 04110 | M00069 | Staphylococcal arthritis, unspecified knee |
| Staphylcoccus infection in conditions classified elsewhere and of unspecified site, staphylcococus, unspecified | 04110 | M00071 | Staphylococcal arthritis, right ankle and foot |
| Staphylcoccus infection in conditions classified elsewhere and of unspecified site, staphylcococus, unspecified | 04110 | M00072 | Staphylococcal arthritis, left ankle and foot |
| Staphylcoccus infection in conditions classified elsewhere and of unspecified site, staphylcococus, unspecified | 04110 | M00079 | Staphylococcal arthritis, unspecified ankle and foot |
| Staphylcoccus infection in conditions classified elsewhere and of unspecified site, staphylcococus, unspecified | 04110 | M0008 | Staphylococcal arthritis, vertebrae |
| **Appendix B Table-SysI: IDC-9 and ICD-10 Codes for Systemic Infections cont.** | | | |
| **ICD-9 Description** | **icd9_sysi** | **icd10_sysi** | **ICD-10 Description** |
| Staphylcoccus infection in conditions classified elsewhere and of unspecified site, staphylcococus, unspecified | 04110 | M0009 | Staphylococcal polyarthritis |
| Methicillin susceptible Staphylcoccus aureus in conditions classified elsewhere and of unspecified sites | 4111 | A4901 | Methicillin susceptible Staphylococcus aureus infection, unspecified site |
| Methicillin susceptible Staphylcoccus aureus in conditions classified elsewhere and of unspecified sites | 04111 | B9561 | Methicillin susceptible Staphylococcus aureus infection as the cause of diseases classified elsewhere |
| Staphylococcus infection in conditions classified elsewhere and of unspecified site, other staphylococcus (includes Staph toxic shock syndrome) | 04119 | B957 | Other staphylococcus as the cause of diseases classified elsewhere |
| Pneumococcus infection in conditions classified elsewhere and of unspecified site | 0412 | B953 | Streptococcus pneumoniae as the cause of diseases classified elsewhere |
| Pneumococcus infection in conditions classified elsewhere and of unspecified site | 0412 | M0010 | Pneumococcal arthritis, unspecified joint |
| Pneumococcus infection in conditions classified elsewhere and of unspecified site | 0412 | M00111 | Pneumococcal arthritis, right shoulder |
| Pneumococcus infection in conditions classified elsewhere and of unspecified site | 0412 | M00112 | Pneumococcal arthritis, left shoulder |
| Pneumococcus infection in conditions classified elsewhere and of unspecified site | 0412 | M00119 | Pneumococcal arthritis, unspecified shoulder |
| Pneumococcus infection in conditions classified elsewhere and of unspecified site | 0412 | M00121 | Pneumococcal arthritis, right elbow |
| Pneumococcus infection in conditions classified elsewhere and of unspecified site | 0412 | M00122 | Pneumococcal arthritis, left elbow |
| Pneumococcus infection in conditions classified elsewhere and of unspecified site | 0412 | M00129 | Pneumococcal arthritis, unspecified elbow |
| Pneumococcus infection in conditions classified elsewhere and of unspecified site | 0412 | M00131 | Pneumococcal arthritis, right wrist |
| Pneumococcus infection in conditions classified elsewhere and of unspecified site | 0412 | M00132 | Pneumococcal arthritis, left wrist |
| Pneumococcus infection in conditions classified elsewhere and of unspecified site | 0412 | M00139 | Pneumococcal arthritis, unspecified wrist |

| **Appendix B Table-SysI: IDC-9 and ICD-10 Codes for Systemic Infections cont.** | | | |
| --- | --- | --- | --- |
| **ICD-9 Description** | **icd9_sysi** | **icd10_sysi** | **ICD-10 Description** |
| Pneumococcus infection in conditions classified elsewhere and of unspecified site | 0412 | M00141 | Pneumococcal arthritis, right hand |
| Pneumococcus infection in conditions classified elsewhere and of unspecified site | 0412 | M00142 | Pneumococcal arthritis, left hand |
| Pneumococcus infection in conditions classified elsewhere and of unspecified site | 0412 | M00149 | Pneumococcal arthritis, unspecified hand |
| Pneumococcus infection in conditions classified elsewhere and of unspecified site | 0412 | M00151 | Pneumococcal arthritis, right hip |
| Pneumococcus infection in conditions classified elsewhere and of unspecified site | 0412 | M00152 | Pneumococcal arthritis, left hip |
| Pneumococcus infection in conditions classified elsewhere and of unspecified site | 0412 | M00159 | Pneumococcal arthritis, unspecified hip |
| Pneumococcus infection in conditions classified elsewhere and of unspecified site | 0412 | M00161 | Pneumococcal arthritis, right knee |
| Pneumococcus infection in conditions classified elsewhere and of unspecified site | 0412 | M00162 | Pneumococcal arthritis, left knee |
| Pneumococcus infection in conditions classified elsewhere and of unspecified site | 0412 | M00169 | Pneumococcal arthritis, unspecified knee |
| Pneumococcus infection in conditions classified elsewhere and of unspecified site | 0412 | M00171 | Pneumococcal arthritis, right ankle and foot |
| Pneumococcus infection in conditions classified elsewhere and of unspecified site | 0412 | M00172 | Pneumococcal arthritis, left ankle and foot |
| Pneumococcus infection in conditions classified elsewhere and of unspecified site | 0412 | M00179 | Pneumococcal arthritis, unspecified ankle and foot |
| Pneumococcus infection in conditions classified elsewhere and of unspecified site | 0412 | M0018 | Pneumococcal arthritis, vertebrae |
| Pneumococcus infection in conditions classified elsewhere and of unspecified site | 0412 | M0019 | Pneumococcal polyarthritis |
| Friedlander's bacillus infection in conditions classified elsewhere and of unspecified sites | 0413 | B961 | Klebsiella pneumoniae [K. pneumoniae] as the cause of diseases classified elsewhere |
| **Appendix B Table-SysI: IDC-9 and ICD-10 Codes for Systemic Infections cont.** | | | |
| **ICD-9 Description** | **icd9_sysi** | **icd10_sysi** | **ICD-10 Description** |
| Other and undspecified Escherichia coli | 04149 | B9629 | Other Escherichia coli [E. coli] as the cause of diseases classified elsewhere |
| Other and undspecified Escherichia coli | 04149 | B9620 | Unspecified Escherichia coli [E. coli] as the cause of diseases classified elsewhere |
| H.influenzae infection in conditions classified elsewhere and of unspecified site | 0415 | A492 | Hemophilus influenzae infection, unspecified site |
| H.influenzae infection in conditions classified elsewhere and of unspecified site | 0415 | B963 | Hemophilus influenzae [H. influenzae] as the cause of diseases classified elsewhere |
| H.influenzae infection in conditions classified elsewhere and of unspecified site | 0415 | J201 | Acute bronchitis due to Hemophilus influenzae |
| Proteus mirabilis/morganii infection in conditions classified elsewhere and of unspecified site | 0416 | B964 | Proteus (mirabilis) (morganii) as the cause of diseases classified elsewhere |
| Pseudomonas infection in conditions classified elewhere and of unspecified sites | 0417 | B965 | Pseudomonas (aeruginosa) (mallei) (pseudomallei) as the cause of diseases classified elsewhere |
| Other specified bacterial infections in conditions classified elsewhere and of unspecified site, mycoplasma | 04181 | A493 | Mycoplasma infection, unspecified site |
| Other specified bacterial infections in conditions classified elsewhere and of unspecified site, mycoplasma | 04181 | B960 | Mycoplasma pneumoniae [M. pneumoniae] as the cause of diseases classified elsewhere |
| Other specified bacterial infections in conditions classified elsewhere and of unspecified site, mycoplasma | 04181 | J200 | Acute bronchitis due to Mycoplasma pneumoniae |
| Bacteroides fragilis | 04182 | B966 | Bacteroides fragilis [B. fragilis] as the cause of diseases classified elsewhere |
| Other specified bacterial infections in conditions classified elsewhere and of unspecified site, Clostridium perfringens | 04183 | B967 | Clostridium perfringens [C. perfringens] as the cause of diseases classified elsewhere |
| Other specified bacterial infections in conditions classified elsewhere and of unspecified site, other anaerobes | 04184 | B9682 | Vibrio vulnificus as the cause of diseases classified elsewhere |
| Other specified bacterial infections in conditions classified elsewhere and of unspecified site, other anaerobes | 04184 | M9689 | Other intraoperative and postprocedural complications and disorders of the musculoskeletal system |
| Other specified bacterial infections in conditions classified elsewhere and of unspecified site, other gram-negative organisms | 04185 | B9689 | Other specified bacterial agents as the cause of diseases classified elsewhere |

| **Appendix B Table-SysI: IDC-9 and ICD-10 Codes for Systemic Infections cont.** | | | | | | |  |
| --- | --- | --- | --- | --- | --- | --- | --- |
| **ICD-9 Description** | **icd9_sysi** | | **icd10_sysi** | **ICD-10 Description** | | |  |
| Other specified bacterial infections in conditions classified elsewhere and of unspecified site, other other specified bacteria | 04189 | | A498 | Other bacterial infections of unspecified site | | |  |
| Other specified bacterial infections in conditions classified elsewhere and of unspecified site, other other specified bacteria | 04189 | | B9689 | Other specified bacterial agents as the cause of diseases classified elsewhere | | |  |
| Other specified bacterial infections in conditions classified elsewhere and of unspecified site, other other specified bacteria | 04189 | | M0080 | Staphylococcal arthritis, vertebrae | | |  |
| Other specified bacterial infections in conditions classified elsewhere and of unspecified site, other other specified bacteria | 04189 | | M00811 | Arthritis due to other bacteria, right shoulder | | |  |
| Other specified bacterial infections in conditions classified elsewhere and of unspecified site, other other specified bacteria | 04189 | | M00812 | Arthritis due to other bacteria, left shoulder | | |  |
| Other specified bacterial infections in conditions classified elsewhere and of unspecified site, other other specified bacteria | 04189 | | M00819 | Arthritis due to other bacteria, unspecified shoulder | | |  |
| Other specified bacterial infections in conditions classified elsewhere and of unspecified site, other other specified bacteria | 04189 | | M00821 | Arthritis due to other bacteria, right elbow | | |  |
| Other specified bacterial infections in conditions classified elsewhere and of unspecified site, other other specified bacteria | 04189 | | M00822 | Arthritis due to other bacteria, left elbow | | |  |
| Other specified bacterial infections in conditions classified elsewhere and of unspecified site, other other specified bacteria | 04189 | | M00829 | Arthritis due to other bacteria, unspecified elbow | | |  |
| Other specified bacterial infections in conditions classified elsewhere and of unspecified site, other other specified bacteria | 04189 | | M00831 | Arthritis due to other bacteria, right wrist | | |  |
| Other specified bacterial infections in conditions classified elsewhere and of unspecified site, other other specified bacteria | 04189 | | M00832 | Arthritis due to other bacteria, left wrist | | |  |
| Other specified bacterial infections in conditions classified elsewhere and of unspecified site, other other specified bacteria | 04189 | | M00839 | Arthritis due to other bacteria, unspecified wrist | | |  |
| Other specified bacterial infections in conditions classified elsewhere and of unspecified site, other other specified bacteria | 04189 | | M00841 | Arthritis due to other bacteria, right hand | | |  |
| Other specified bacterial infections in conditions classified elsewhere and of unspecified site, other other specified bacteria | 04189 | | M00842 | Arthritis due to other bacteria, left hand | | |  |
| Other specified bacterial infections in conditions classified elsewhere and of unspecified site, other other specified bacteria | 04189 | | M00849 | Arthritis due to other bacteria, unspecified hand | | |  |
| **Appendix B Table-SysI: IDC-9 and ICD-10 Codes for Systemic Infections cont.** | | | | | | |  |
| **ICD-9 Description** | **icd9_sysi** | | **icd10_sysi** | **ICD-10 Description** | | |  |
| Other specified bacterial infections in conditions classified elsewhere and of unspecified site, other other specified bacteria | 04189 | | M00851 | Arthritis due to other bacteria, right hip | | |  |
| Other specified bacterial infections in conditions classified elsewhere and of unspecified site, other other specified bacteria | 04189 | | M00852 | Arthritis due to other bacteria, left hip | | |  |
| Other specified bacterial infections in conditions classified elsewhere and of unspecified site, other other specified bacteria | 04189 | | M00859 | Arthritis due to other bacteria, unspecified hip | | |  |
| Other specified bacterial infections in conditions classified elsewhere and of unspecified site, other other specified bacteria | 04189 | | M00861 | Arthritis due to other bacteria, right knee | | |  |
| Other specified bacterial infections in conditions classified elsewhere and of unspecified site, other other specified bacteria | 04189 | | M00862 | Arthritis due to other bacteria, left knee | | |  |
| Other specified bacterial infections in conditions classified elsewhere and of unspecified site, other other specified bacteria | 04189 | | M00869 | Arthritis due to other bacteria, unspecified knee | | |  |
| Other specified bacterial infections in conditions classified elsewhere and of unspecified site, other other specified bacteria | 04189 | | M0088 | Arthritis due to other bacteria, vertebrae | | |  |
| Other specified bacterial infections in conditions classified elsewhere and of unspecified site, other other specified bacteria | 04189 | | M0089 | Polyarthritis due to other bacteria | | |  |
| Bacterial infection, unspecified, in conditions classified elsewhere and of unspecified site | 0419 | | A499 | Bacterial infection, unspecified | | |  |
| Bacterial infection, unspecified, in conditions classified elsewhere and of unspecified site | 0419 | | B9689 | Other specified bacterial agents as the cause of diseases classified elsewhere | | |  |
| Adenovirus infection | 0790 | | B340 | Adenovirus infection, unspecified | | |  |
| Adenovirus infection | 0790 | | B970 | Adenovirus as the cause of diseases classified elsewhere | | |  |
| Rhinovirus infection in condition classified elsewhere and of unspecified site | 0793 | | B9789 | Other viral agents as the cause of diseases classified elsewhere | | |  |
| Rhinovirus infection in condition classified elsewhere and of unspecified site | 0793 | | J206 | Acute bronchitis due to rhinovirus | | |  |
| Other specified viral infection | 07989 | | B338 | Other specified viral diseases | | |  |
| Other specified viral infection | 07989 | | B341 | Enterovirus infection, unspecified | | |  |
| Other specified viral infection | 07989 | | B342 | Coronavirus infection, unspecified | | |  |
| Other specified viral infection | 07989 | | B344 | Papovavirus infection, unspecified | | |  |
| Other specified viral infection | 07989 | | B348 | Other viral infections of unspecified site | | |  |
| **Appendix B Table-SysI: IDC-9 and ICD-10 Codes for Systemic Infections cont.** | | | | | | |  |
| **ICD-9 Description** | **icd9_sysi** | | **icd10_sysi** | **ICD-10 Description** | | |  |
| Other specified viral infection | 07989 | | B9719 | Other enterovirus as the cause of diseases classified elsewhere | | |  |
| Other specified viral infection | 07989 | | B9729 | Other coronavirus as the cause of diseases classified elsewhere | | |  |
| Other specified viral infection | 07989 | | B975 | Reovirus as the cause of diseases classified elsewhere | | |  |
| Other specified viral infection | 07989 | | B976 | Parvovirus as the cause of diseases classified elsewhere | | |  |
| Other specified viral infection | 07989 | | B9781 | Human metapneumovirus as the cause of diseases  elsewhere | | |  |
| Other specified viral infection | 07989 | | B9789 | Other viral agents as the cause of diseases classified elsewhere | | |  |
| Other specified viral infection | 07989 | | J204 | Acute bronchitis due to parainfluenza virus | | |  |
| Unspecified viral infection | 07999 | | B9710 | Unspecified enterovirus as the cause of diseases classified  elsewhere | | |  |
| Unspecified viral infection | 07999 | | B9789 | Other viral agents as the cause of diseases classified elsewhere | | |  |
| Other specified infectious and parasitic diseases | 1368 | | B608 | Other specified protozoal diseases | | |  |
| Other specified infectious and parasitic diseases | 1368 | | B998 | Other infectious disease | | |  |
| Other specified infecitous and parasitic diseases of mother, delivered, with or without mention of antepartum condition | 64781 | | O98611 | Protozoal diseases complicating pregnancy, first trimester | | |  |
| Other specified infecitous and parasitic diseases of mother, delivered, with or without mention of antepartum condition | 64781 | | O98612 | Protozoal diseases complicating pregnancy, second trimester | | |  |
| Other specified infecitous and parasitic diseases of mother, delivered, with or without mention of antepartum condition | 64781 | | O98613 | Protozoal diseases complicating pregnancy, third trimester | | |  |
| Other specified infecitous and parasitic diseases of mother, delivered, with or without mention of antepartum condition | 64781 | | O98811 | Other maternal infectious and parasitic diseases complicating pregnancy, first trimester | | |  |
| Other specified infecitous and parasitic diseases of mother, delivered, with or without mention of antepartum condition | 64781 | | O98812 | Other maternal infectious and parasitic diseases complicating pregnancy, second trimester | | |  |
| Other specified infecitous and parasitic diseases of mother, delivered, with or without mention of antepartum condition | 64781 | | O98813 | Other maternal infectious and parasitic diseases complicating pregnancy, third trimester | | |  |
| Other specified infecitous and parasitic diseases of mother, delivered, with or without mention of antepartum condition | 64781 | | O9882 | Other maternal infectious and parasitic diseases complicating childbirth | | |  |
| Other specified infecitous and parasitic diseases of mother, delivered, with or without mention of antepartum condition | 64781 | | O99830 | Other infection carrier state complicating pregnancy | | |  |
| Other specified infecitous and parasitic diseases of mother, delivered, with or without mention of antepartum condition | 64781 | | O99834 | Other infection carrier state complicating childbirth | | |  |
| **Appendix B Table-SysI: IDC-9 and ICD-10 Codes for Systemic Infections cont.** | | | | | | |  |
| **ICD-9 Description** | **icd9_sysi** | | **icd10_sysi** | **ICD-10 Description** | | |  |
| Shock, unspecified | 78550 | | R579 | Shock, unspecified | | |  |
| Septic shock | 78552 | | R6521 | Severe sepsis with septic shock | | |  |
| Sepsis with acute organ dysfunction, sepsis with multiple organ dysfunction, severe sepsis | 99592 | | R6520 | Severe sepsis without septic shock | | |  |
| Sepsis with acute organ dysfunction, sepsis with multiple organ dysfunction, severe sepsis | 99592 | | R6521 | Severe sepsis with septic shock | | |  |
| Postoperative shock, unspecified | 99800 | | T8110XA | Postprocedural shock unspecified, initial encounter | | |  |
| postoperative shock, septic | 99802 | | T8112XA | Postprocedural septic shock, initial encounter | | |  |
| Postoperative shock, other | 99809 | | T8119XA | Other postprocedural shock, initial encounter | | |  |
| Infection following infusion, injection, transfusion, or vaccination (including sepsis) | 99939 | | N980 | Infection associated with artificial insemination | | |  |
| Infection following infusion, injection, transfusion, or vaccination (including sepsis) | 99939 | | T8029XA | Infection following other infusion, transfusion and therapeutic injection, initial encounter | | |  |
| Infection following infusion, injection, transfusion, or vaccination (including sepsis) | 99939 | | T880XXA | Infection following immunization, initial encounter | | |  |
|  | |  | | |  |  | |

**Appendix B Table 2-SYSI: Univariate Statistics 2011 and 2016**

| **Patient Characteristics** | **SYSI - Group Comparisons 2011** | | | **SYSI - Group Comparisons 2016** | | |
| --- | --- | --- | --- | --- | --- | --- |
|  | **Cases with Secondary Dx, LOS > 2 days** | **Cases with Principal DX** | **Matched Cases with No Dx** | **Cases with Secondary Dx, LOS > 2 days** | **Cases with Principal DX** | **Matched Cases with No Dx** |
|  |  |  |  |  |  |  |
|  |  |  |  |  |  |  |
| **n =** | 447,047 | 8,478 | 1,590,801 | 456,813 | 11,746 | 1,497,653 |
| **Variables:** |  |  |  |  |  |  |
| **Cost** |  |  |  |  |  |  |
| **mean** | $25,888 | $6,718 | $12,679 | $26,446 | $14,989 | $12,787 |
| **median** | $13,438 | $4,385 | $7,740 | $13,888 | $8,675 | $7,953 |
| **mode** | $4,151 | $1,216 | $78 | $12,594 | $21,532 | $3,148 |
| **range** | $34 - $1,756,572 | $78 - $242,122 | $29 - $1,736,670 | $30 - $2,850,000 | $57 - $910,613 | $19 - $2,241,437 |
| **LOS** |  |  |  |  |  |  |
| **mean** | 10.27 | 3.21 | 5.18 | 10.09 | 6.01 | 5.06 |
| **median** | 7 | 2 | 3 | 6 | 4 | 3 |
| **mode** | 3 | 2 | 2 | 3 | 2 | 2 |
| **range** | 3 - 365 | 1 - 86 | 1 - 365 | 3 - 365 | 1 - 326 | 1 - 361 |
| **Number of Diagnoses; mean (SD)** | 15.27 (6.41) | 7.65 (5.34) | 10.33 (5.73) | 17.76 (6.42) | 12.47 (7.12) | 12.40 (6.49) |
| **Number of Procedures; mean (SD)** | 2.65 (3.34) | 0.76 (1.38) | 1.53 (2.14) | 2.65 (3.35) | 2.07 (2.47) | 1.48 (2.14) |
| **age; mean (range)** | 63 (0-111) | 36 (0-102) | 59 (0-123) | 63 (0-90) | 46 (0-90) | 59 (0-90) |
| **APRDRG_Severity; mean (SD)** | 3.07 (0.84) | 2.02 (0.86) | 2.35 (0.91) | 3.03 (0.87) | 2.58 (0.95) | 2.33 (0.88) |
| **APRDRG_Risk_Mortality; mean (SD)** | 2.64 (1.10) | 1.54 (0.83) | 1.95 (0.95) | 2.80 (1.06) | 2.14 (1.15) | 2.02 (0.97) |
| **Wage Index: mean (SD)** | 1.0038 (0.157) | 1.0094 (0.159) | 0.9996 (0.159) | 1.0057 (0.199) | 0.9970 (0.191) | 1.0001 (0.195) |

**Appendix B Table 3-SYSI: Results of Median Regression Models 2011 and 2016**

| **Median Regression Estimates for Cost of SYSI as a Principal Diagnosis (n=8465) 2011** | | | | | | | **Median Regression Estimates for Cost of SYSI as a Principal Diagnosis (n=11736) 2016** | | | | | | |
| --- | --- | --- | --- | --- | --- | --- | --- | --- | --- | --- | --- | --- | --- |
| **Parameter** | **Estimate** | **Standard Error** | **95% Confidence Limits** | | **t Value** | **Pr > \|t\|** | **Parameter** | **Estimate** | **Standard Error** | **95% Confidence Limits** | | **t Value** | **Pr > \|t\|** |
| **Intercept** | -6420.16 | 238.6383 | -6887.945 | -5952.366 | -26.9 | <.0001 | **Intercept** | -8093.58 | 284.9179 | -8652.066 | -7535.093 | -28.41 | <.0001 |
| **LOS** | 1386.768 | 33.0689 | 1321.9449 | 1451.591 | 41.94 | <.0001 | **LOS** | 1624.579 | 31.7802 | 1562.2842 | 1686.8732 | 51.12 | <.0001 |
| **NDX** | 89.4483 | 11.176 | 67.5407 | 111.356 | 8 | <.0001 | **NDX** | 55.0768 | 11.2717 | 32.9825 | 77.1711 | 4.89 | <.0001 |
| **NPR** | 784.196 | 48.5973 | 688.9334 | 879.4586 | 16.14 | <.0001 | **NPR** | 1281.069 | 46.5966 | 1189.7316 | 1372.4058 | 27.49 | <.0001 |
| **URBAN_TEACH** | 210.2487 | 55.2726 | 101.9008 | 318.5965 | 3.8 | 0.0001 | **URBAN_TEACH** | 297.7106 | 98.157 | 105.3065 | 490.1147 | 3.03 | 0.0024 |
| **URBAN_TEACH** | 0 | 0 | 0 | 0 | . | . | **URBAN_TEACH** | 0 | 0 | 0 | 0 | . | . |
| **SMALL_BEDSIZE** | -85.0408 | 101.8306 | -284.6537 | 114.572 | -0.84 | 0.4037 | **SMALL_BEDSIZE** | -492.398 | 105.6834 | -699.5552 | -285.2411 | -4.66 | <.0001 |
| **SMALL_BEDSIZE** | 0 | 0 | 0 | 0 | . | . | **SMALL_BEDSIZE** | 0 | 0 | 0 | 0 | . | . |
| **AGE** | 6.0392 | 1.3285 | 3.4351 | 8.6433 | 4.55 | <.0001 | **AGE** | -16.6443 | 2.0736 | -20.7088 | -12.5798 | -8.03 | <.0001 |
| **APRDRG_Severity** | 165.4499 | 58.3545 | 51.0608 | 279.8391 | 2.84 | 0.0046 | **APRDRG_Severity** | -135.934 | 88.2733 | -308.9648 | 37.0959 | -1.54 | 0.1236 |
| **APRDRG_Risk_Mortality** | 202.9854 | 66.3874 | 72.8499 | 333.1208 | 3.06 | 0.0022 | **APRDRG_Risk_Mortality** | 1493.057 | 79.2579 | 1337.698 | 1648.4153 | 18.84 | <.0001 |
| **Wage_Index** | 5696.747 | 199.5139 | 5305.6506 | 6087.8427 | 28.55 | <.0001 | **Wage_Index** | 6543.421 | 237.6205 | 6077.6454 | 7009.1966 | 27.54 | <.0001 |

**Specified Stage 2 Cost Simulation Models for SYSI 2011 and 2016**

**Cost_sysi_2011 = -6420.16 + 1386.768*3.21137061 + 89.4483*NDX + 784.196*NPR + 210.2487*URBAN_TEACH + (-85.0408)*SMALL_BEDSIZE +**

**6.0392*AGE + 165.4499*APRDRG_Severity + 202.9854*APRDRG_Risk_Mortality + 5696.747*Wage_Index**

**Cost_sysi_2016 = -8093.58 + 1624.579*6.00647029 + 55.0768*NDX + 1281.069*NPR + 297.7106*URBAN_TEACH + (-492.398)*SMALL_BEDSIZE +**

**(-16.6443)*AGE + (-135.934)*APRDRG_Severity + 1493.057*APRDRG_Risk_Mortality + 6543.421*Wage_Index**

**Urinary Tract Infections (UTI)**

| **Appendix B Table 1-UTI: IDC-9 and ICD-10 Codes for Urinary Tract Infections** | | | |
| --- | --- | --- | --- |
| **ICD-9 Definition** | **icd9_uti** | **icd10_uti** | **ICD-10 Definition** |
| Candidiasis of urinary tract | 1122 | B3741 | Candidal cystitis and urethritis |
| Candidiasis of urinary tract | 1122 | B3742 | Candidal balanitis |
| Candidiasis of urinary tract | 1122 | B3749 | Other urogenital candidiasis |
| Urinary tract infection of newborn | 77182 | P393 | Neonatal urinary tract infection |
| Infections of genitourinary tract in pregnancy, unspecified as to episode of care or not applicable | 64660 | O2300 | Infections of kidney in pregnancy, unspecified trimester |
| Infections of genitourinary tract in pregnancy, unspecified as to episode of care or not applicable | 64660 | 2310 | Infections of bladder in pregnancy, unspecified trimester |
| Infections of genitourinary tract in pregnancy, unspecified as to episode of care or not applicable | 64660 | O2320 | Infections of urethra in pregnancy, unspecified trimester |
| Infections of genitourinary tract in pregnancy, unspecified as to episode of care or not applicable | 64660 | O2330 | Infections of other parts of urinary tract in pregnancy, unspecified trimester |
| Infections of genitourinary tract in pregnancy, unspecified as to episode of care or not applicable | 64660 | O2340 | Unspecified infection of urinary tract in pregnancy, unspecified trimester |
| Infections of genitourinary tract in pregnancy, unspecified as to episode of care or not applicable | 64660 | O23519 | Infections of cervix in pregnancy, unspecified trimester |
| Infections of genitourinary tract in pregnancy, unspecified as to episode of care or not applicable | 64660 | O23529 | Salpingo-oophoritis in pregnancy, unspecified trimester |
| Infections of genitourinary tract in pregnancy, unspecified as to episode of care or not applicable | 64660 | O23599 | Infection of other part of genital tract in pregnancy, unspecified trimester |
| Infections of genitourinary tract in pregnancy, unspecified as to episode of care or not applicable | 64660 | O2390 | Unspecified genitourinary tract infection in pregnancy, unspecified trimester |
| Infections of the genitourinary tract in pregnancy, antepartum condition or complication | 64663 | O2301 | Infections of kidney in pregnancy, first trimester |
| Infections of the genitourinary tract in pregnancy, antepartum condition or complication | 64663 | O2302 | Infections of kidney in pregnancy, second trimester |
| **Appendix B Table 1-UTI: IDC-9 and ICD-10 Codes for Urinary Tract Infections cont.** | | | |
| **ICD-9 Definition** | **icd9_uti** | **icd10_uti** | **ICD-10 Definition** |
| Infections of the genitourinary tract in pregnancy, antepartum condition or complication | 64663 | O2303 | Infections of kidney in pregnancy, third trimester |
| Infections of the genitourinary tract in pregnancy, antepartum condition or complication | 64663 | O2311 | Infections of bladder in pregnancy, first trimester |
| Infections of the genitourinary tract in pregnancy, antepartum condition or complication | 64663 | O2312 | Infections of bladder in pregnancy, second trimester |
| Infections of the genitourinary tract in pregnancy, antepartum condition or complication | 64663 | O2313 | Infections of bladder in pregnancy, third trimester |
| Infections of the genitourinary tract in pregnancy, antepartum condition or complication | 64663 | O2321 | Infections of urethra in pregnancy, first trimester |
| Infections of the genitourinary tract in pregnancy, antepartum condition or complication | 64663 | O2322 | Infections of urethra in pregnancy, second trimester |
| Infections of the genitourinary tract in pregnancy, antepartum condition or complication | 64663 | O2323 | Infections of urethra in pregnancy, third trimester |
| Infections of the genitourinary tract in pregnancy, antepartum condition or complication | 64663 | O2331 | Infections of other parts of urinary tract in pregnancy, first trimester |
| Infections of the genitourinary tract in pregnancy, antepartum condition or complication | 64663 | O2332 | Infections of other parts of urinary tract in pregnancy, second trimester |
| Infections of the genitourinary tract in pregnancy, antepartum condition or complication | 64663 | O2333 | Infections of other parts of urinary tract in pregnancy, third trimester |
| Infections of the genitourinary tract in pregnancy, antepartum condition or complication | 64663 | O2341 | Unspecified infection of urinary tract in pregnancy, first trimester |
| Infections of the genitourinary tract in pregnancy, antepartum condition or complication | 64663 | O2342 | Unspecified infection of urinary tract in pregnancy, second trimester |
| Infections of the genitourinary tract in pregnancy, antepartum condition or complication | 64663 | O2343 | Unspecified infection of urinary tract in pregnancy, third trimester |
| Infections of the genitourinary tract in pregnancy, antepartum condition or complication | 64663 | O23511 | Infections of cervix in pregnancy, first trimester |
| **Appendix B Table 1-UTI: IDC-9 and ICD-10 Codes for Urinary Tract Infections cont.** | | | |
| **ICD-9 Definition** | **icd9_uti** | **icd10_uti** | **ICD-10 Definition** |
| Infections of the genitourinary tract in pregnancy, antepartum condition or complication | 64663 | O23512 | Infections of cervix in pregnancy, second trimester |
| Infections of the genitourinary tract in pregnancy, antepartum condition or complication | 64663 | O23513 | Infections of cervix in pregnancy, third trimester |
| Infections of the genitourinary tract in pregnancy, antepartum condition or complication | 64663 | O23522 | Salpingo-oophoritis in pregnancy, second trimester |
| Infections of the genitourinary tract in pregnancy, antepartum condition or complication | 64663 | O23523 | Salpingo-oophoritis in pregnancy, third trimester |
| Infections of the genitourinary tract in pregnancy, antepartum condition or complication | 64663 | O23591 | Infection of other part of genital tract in pregnancy, first trimester |
| Infections of the genitourinary tract in pregnancy, antepartum condition or complication | 64663 | O23592 | Infection of other part of genital tract in pregnancy, second trimester |
| Infections of the genitourinary tract in pregnancy, antepartum condition or complication | 64663 | O23593 | Infection of other part of genital tract in pregnancy, third trimester |
| Infections of the genitourinary tract in pregnancy, antepartum condition or complication | 64663 | O2390 | Unspecified genitourinary tract infection in pregnancy, unspecified trimester |
| Infections of the genitourinary tract in pregnancy, antepartum condition or complication | 64663 | O2391 | Unspecified genitourinary tract infection in pregnancy, first trimester |
| Infections of the genitourinary tract in pregnancy, antepartum condition or complication | 64663 | O2392 | Unspecified genitourinary tract infection in pregnancy, second trimester |
| Infections of the genitourinary tract in pregnancy, antepartum condition or complication | 64663 | O2393 | Unspecified genitourinary tract infection in pregnancy, third trimester |
| Acute pyelonephritis with lesion of renal medullary necrosis | 59011 | N10 | Acute pyelonephritis |
| Renal and perinephric abscess | 5902 | N151 | Renal and perinephric abscess |
| Pyeloureteritis cystica | 5903 | N2884 | Pyelitis cystica |
| Pyeloureteritis cystica | 5903 | N2885 | Pyeloureteritis cystica |
| Pyeloureteritis cystica | 5903 | N2886 | Ureteritis cystica |
| Pyelonephritis, unspecified | 59080 | N119 | Chronic tubulo-interstitial nephritis, unspecified |
| **Appendix B Table 1-UTI: IDC-9 and ICD-10 Codes for Urinary Tract Infections cont.** | | | |
| **ICD-9 Definition** | **icd9_uti** | **icd10_uti** | **ICD-10 Definition** |
| Pyelonephritis, unspecified | 59080 | N12 | Tubulo-interstitial nephritis, not specified as acute or chronic |
| Pyelonephritis, unspecified | 59080 | N136 | Pyonephrosis |
| Pyelitis or pyelonephritis in diseases classified elsewhere | 59081 | N16 | Renal tubulo-interstitial disorders in diseases classified elsewhere |
| Infection of kidney, unspecified | 5909 | N159 | Renal tubulo-interstitial disease, unspecified |
| Acute cystitis | 5950 | N3000 | Acute cystitis without hematuria |
| Acute cystitis | 5950 | N3001 | Acute cystitis with hematuria |
| Cystitis in diseases classified elsewhere | 5954 | A5601 | Chlamydial cystitis and urethritis |
| Cystitis in diseases classified elsewhere | 5954 | N3080 | Other cystitis without hematuria |
| Cystitis in diseases classified elsewhere | 5954 | N3081 | Other cystitis with hematuria |
| Cystitis, unspecified | 5959 | N3090 | Cystitis, unspecified without hematuria |
| Cystitis, unspecified | 5959 | N3091 | Cystitis, unspecified with hematuria |
| Uretethral abscess | 5970 | N340 | Urethral abscess |
| Urethritis, unspecified | 59780 | N341 | Nonspecific urethritis |
| Urethritis, unspecified | 59780 | N342 | Other urethritis |
| Other urethritis | 59789 | N343 | Urethral syndrome, unspecified |
| Urethral stricture due to unspecified infection | 59800 | N35111 | Postinfective urethral stricture, not elsewhere classified, male, meatal |
| Urethral stricture due to unspecified infection | 59800 | N35112 | Postinfective bulbous urethral stricture, not elsewhere classified, male |
| Urethral stricture due to unspecified infection | 59800 | N35113 | Postinfective membranous urethral stricture, not elsewhere classified, male |
| Urethral stricture due to unspecified infection | 59800 | N35114 | Postinfective anterior urethral stricture, not elsewhere classified, male |
| Urethral stricture due to unspecified infection | 59800 | N35119 | Postinfective urethral stricture, not elsewhere classified, male, unspecified |
| Urethral stricture due to unspecified infection | 59800 | N3512 | Postinfective urethral stricture, not elsewhere classified, female |
| **Appendix B Table 1-UTI: IDC-9 and ICD-10 Codes for Urinary Tract Infections cont.** | | | |
| **ICD-9 Definition** | **icd9_uti** | **icd10_uti** | **ICD-10 Definition** |
| Urethral stricture due to infective disease classified elsewhere | 59801 | N37 | Urethral disorders in diseases classified elsewhere |
| Urinary tract infection, site not specified | 5990 | N390 | Urinary tract infection, site not specified |
| Urinary (tract) infection | V1302 | Z87440 | Personal history of urinary (tract) infections |

**Appendix B Table 2-UTI: Univariate Statistics 2011 and 2016**

| **Patient Characteristics** | **UTI - Group Comparisons 2011** | | | **UTI - Group Comparisons 2016** | | |
| --- | --- | --- | --- | --- | --- | --- |
|  | **Cases with Secondary Dx** | **Cases with Principal DX** | **Matched Cases with No Dx** | **Cases with Secondary Dx** | **Cases with Principal DX** | **Matched Cases with No Dx** |
|  |  |  |  |  |  |  |
|  |  |  |  |  |  |  |
| **n =** | 465,732 | 130,217 | 1,961,233 | 452,591 | 103,923 | 1,868,330 |
| **Variables:** |  |  |  |  |  |  |
| **Cost** |  |  |  |  |  |  |
| **mean** | $21,202 | $7,259 | $13,270 | $19,467 | $6,888 | $13,937 |
| **median** | $12,072 | $5,411 | $8,091 | $11,304 | $5,416 | $8,573 |
| **mode** | $4,151 | $175 | $78 | $12,594 | $3,148 | $3,148 |
| **range** | $34 -$1,620,419 | $65 - $650,410 | $26 - $1,756,572 | $30 -$2,009,951 | $51 - $369,721 | $19 - $2,850,000 |
| **LOS** |  |  |  |  |  |  |
| **mean** | 9.44 | 4.14 | 5.30 | 8.81 | 3.89 | 5.37 |
| **median** | 6 | 3 | 3 | 6 | 3 | 4 |
| **mode** | 3 | 3 | 2 | 3 | 3 | 2 |
| **range** | 1 - 365 | 1- 342 | 1 - 365 | 3 - 365 | 1- 320 | 1 - 365 |
| **Number of Diagnoses; mean (SD)** | 15.00 (5.98) | 10.87 (5.57) | 10.39 (5.74) | 17.21 (6.19) | 12.42 (6.16) | 12.65 (6.55) |
| **Number of Procedures; mean (SD)** | 2.00 (2.81) | 0.50 (1.09) | 1.61 (2.24) | 1.81 (2.69) | 0.44 (1.04) | 1.61 (2.30) |
| **age; mean (range)** | 69 (0 - 111) | 64 (0 - 112) | 60 (0-123) | 68 (0 - 90) | 64 (0 - 90) | 60 (0-90) |
| **APRDRG_Severity; mean (SD)** | 2.98 (0.76) | 2.42 (0.81) | 2.34 (0.92) | 2.81 (0.84) | 2.25 (0.76) | 2.36 (0.91) |
| **APRDRG_Risk_Mortality; mean (SD)** | 2.58 (0.99) | 2.02 (0.92) | 1.96 (0.98) | 2.62 (0.97) | 1.99 (0.86) | 2.07 (1.02) |
| **Wage Index; mean (SD)** | 0.9950 (0.156) | 0.9906 (0.158) | 1.0015 (0.160) | 0.9956 (0.197) | 0.9891 (0.196) | 1.0015 (0.195) |

**Appendix B Table 3-UTI: Results of Median Regression Models 2011 and 2016**

| **Median Regression Estimates for Cost of UTI as a Principal Diagnosis (n=130185) 2011** | | | | | | | **Median Regression Estimates for Cost of UTI as a Principal Diagnosis (n= 103905) 2016** | | | | | | |
| --- | --- | --- | --- | --- | --- | --- | --- | --- | --- | --- | --- | --- | --- |
| **Parameter** | **Estimate** | **Standard Error** | **95% Confidence Limits** | | **t Value** | **Pr > \|t\|** | **Parameter** | **Estimate** | **Standard Error** | **95% Confidence Limits** | | **t Value** | **Pr > \|t\|** |
| **Intercept** | -5707.90 | 62.4815 | -5830.368 | -5585.442 | -91.35 | <.0001 | **Intercept** | -3237.23 | 52.8839 | -3340.886 | -3133.582 | -61.21 | <.0001 |
| **LOS** | 1043.199 | 6.4325 | 1030.5916 | 1055.8069 | 162.18 | <.0001 | **LOS** | 959.7469 | 6.9666 | 946.0924 | 973.4014 | 137.76 | <.0001 |
| **NDX** | 77.5170 | 2.3302 | 72.9498 | 82.0842 | 33.27 | <.0001 | **NDX** | 54.0325 | 1.7140 | 50.6731 | 57.3919 | 31.52 | <.0001 |
| **NPR** | 995.4099 | 16.4685 | 963.1319 | 1027.6879 | 60.44 | <.0001 | **NPR** | 1105.307 | 16.7174 | 1072.5412 | 1138.0729 | 66.12 | <.0001 |
| **URBAN_TEACH** | 468.5850 | 14.8344 | 439.5099 | 497.6601 | 31.59 | <.0001 | **URBAN_TEACH** | 462.5805 | 15.8915 | 431.4333 | 493.7276 | 29.11 | <.0001 |
| **URBAN_TEACH** | 0.0000 | 0.0000 | 0.0000 | 0.0000 | . | . | **URBAN_TEACH** | 0.0000 | 0.0000 | 0.0000 | 0.0000 | . | . |
| **SMALL_BEDSIZE** | -391.677 | 19.8209 | -430.5257 | -352.8284 | -19.76 | <.0001 | **SMALL_BEDSIZE** | -558.486 | 19.2813 | -596.2767 | -520.6943 | -28.97 | <.0001 |
| **SMALL_BEDSIZE** | 0.0000 | 0.0000 | 0.0000 | 0.0000 | . | . | **SMALL_BEDSIZE** | 0.0000 | 0.0000 | 0.0000 | 0.0000 | . | . |
| **AGE** | -2.4499 | 0.3129 | -3.0632 | -1.8366 | -7.83 | <.0001 | **AGE** | -1.2311 | 0.3840 | -1.9838 | -0.4784 | -3.21 | 0.0013 |
| **APRDRG_Severity** | 288.0838 | 15.8608 | 256.9969 | 319.1707 | 18.16 | <.0001 | **APRDRG_Severity** | 286.4116 | 17.9674 | 251.1958 | 321.6275 | 15.94 | <.0001 |
| **APRDRG_Risk_Mortality** | 1.9330 | 15.2336 | -27.9246 | 31.7907 | 0.13 | 0.8990 | **APRDRG_Risk_Mortality** | -21.3095 | 15.2215 | -51.1435 | 8.5246 | -1.40 | 0.1615 |
| **Wage_Index** | 6205.817 | 59.5922 | 6089.0174 | 6322.6167 | 104.14 | <.0001 | **Wage_Index** | 4438.609 | 44.9768 | 4350.4553 | 4526.7633 | 98.69 | <.0001 |

**Specified Stage 2 Cost Simulation Models for UTI 2011 and 2016**

**Cost_uti_2011 = -5707.90+ 1043.1990*4.13826152 + 77.5170*NDX + 995.4099*NPR + 468.5850*URBAN_TEACH + (-391.677)*SMALL_BEDSIZE +**

**(-2.4499)*AGE + 288.0838*APRDRG_Severity + 1.933*APRDRG_Risk_Mortality + 6205.817*Wage_Index**

**Cost_uti_2016 = -3237.23+ 959.7469*3.88754174+ 54.0325*NDX + 1105.307*NPR + 462.5805*URBAN_TEACH + (-558.486)*SMALL_BEDSIZE +**

**(-1.2311)*AGE + 286.4116*APRDRG_Severity + (-21.3095)*APRDRG_Risk_Mortality + 4438.609*Wage_Index**

**Ventilator-Associated Pneumonia (VAP)**

| **Appendix B Table 1-VAP: IDC-9 and ICD-10 Codes for Ventilator-Associated Pneumonia** | | | |
| --- | --- | --- | --- |
| **ICD-9 Description** | **icd9_vap** | **icd10_vap** | **ICD-10 Description** |
| Adenoviral Pneumonia | 4800 | J120 | Adenoviral pneumonia |
| Pneumonia due to respiratory syncytial virus (RSV) | 4801 | J121 | Respiratory syncytial virus pneumonia |
| Pneumonia due to parainfluenza virus | 4802 | J122 | Parainfluenza virus pneumonia |
| Pneumonia due to SARS-associated coronavirus | 4803 | J1281 | Pneumonia due to SARS-associated coronavirus |
| Pneumonia due to other virus not elsewhere classified | 4808 | J123 | Human metapneumovirus pneumonia |
| Pneumonia due to other virus not elsewhere classified | 4808 | J1289 | Other viral pneumonia |
| Viral pneumonia, unspecified | 4809 | J129 | Viral pneumonia, unspecified |
| Pneumococcal pneumonia; Streptococcus pneumoniae (pneumococcal disease) | 481 | J13 | Pneumonia due to Streptococcus pneumoniae |
| Pneumococcal pneumonia; Streptococcus pneumoniae (pneumococcal disease) | 481 | J181 | Lobar pneumonia, unspecified organism |
| Pneumonia due to Klebsiella pneumoniae | 4820 | J15 | Bacterial pneumonia, not elsewhere classified |
| Pneumonia due to Pseudomonas | 4821 | J151 | Pneumonia due to Pseudomonas |
| Pneumonia due to Haemophilus influenzae | 4822 | J14 | Pneumonia due to Hemophilus influenzae |
| Pneumonia due to Streptococcus (unspecified) | 48230 | J154 | Pneumonia due to other streptococci |
| Pneumonia Strptococcus A | 48231 | J154 | Pneumonia due to other streptococci |
| Pneumonia Strptococcus B | 48232 | J153 | Pneumonia due to streptococcus, group B |
| Pneumonia Oth Strep | 48239 | J154 | Pneumonia due to other streptococci |
| Staphylococcal Pneu Nos | 48240 | J1520 | Pneumonia due to staphylococcus, unspecified |
| Staph Aureus Pneumonia | 48241 | J15211 | Pneumonia due to Methicillin susceptible Staphylococcus aureus |
| Methicillin resistant pneumonia due to Staphylococcus aureus | 48242 | J15212 | Pneumonia due to Methicillin resistant Staphylococcus aureus |
| Staph Pneumonia Nec | 48249 | J1529 | Pneumonia due to other staphylococcus |
| Pneumonia Anaerobes | 48281 | J158 | Pneumonia due to other specified bacteria |
| Pneumonia due to E. coli | 48282 | J155 | Pneumonia due to Escherichia coli |
| Pneumonia due to other gram-negative bacteria | 48283 | J156 | Pneumonia due to other Gram-negative bacteria |
| Legionnaires Disease | 48284 | A481 | Legionnaires' disease |
| Pneumonia due to other specified bacteria | 48289 | J158 | Pneumonia due to other specified bacteria |
| **Appendix B Table 1-VAP: IDC-9 and ICD-10 Codes for Ventilator-Associated Pneumonia** | | | |
| **ICD-9 Description** | **icd9_vap** | **icd10_vap** | **ICD-10 Description** |
| Bacterial pneumonia, unspecified | 4829 | J159 | Unspecified bacterial pneumonia |
| Mycoplasma pneumonia | 4830 | J157 | Pneumonia due to Mycoplasma pneumoniae |
| Pneumonia Due To Chlamydia | 4831 | J160 | Chlamydial pneumonia |
| Pneumon Oth Spec Orgnsm | 4838 | J168 | Pneumonia due to other specified infectious organisms |
| Pneum In Infect Dis Nec | 4848 | B7781 | Ascariasis pneumonia |
| Unspecified infectious and parasitic diseases | 1369 | B999 | Unspecified infectious disease |
| Bronchopneumonia, organism unspecified | 485 | J180 | Bronchopneumonia, unspecified organism |
| Pneumonia; community-acquired pneumonia (CAP); nosocomial pneumonia (hospital-acquired) | 486 | J188 | Other pneumonia, unspecified organism |
| Pneumonia; community-acquired pneumonia (CAP); nosocomial pneumonia (hospital-acquired) | 486 | J189 | Pneumonia, unspecified organism |
| Ventilator associated pneumonia | 99731 | J95851 | Ventilator associated pneumonia |

**Appendix B Table 2-VAP: Univariate Statistics 2011 and 2016**

| **Patient Characteristics** | **VAP - Group Comparisons 2011** | | | **VAP - Group Comparisons 2016** | | |
| --- | --- | --- | --- | --- | --- | --- |
|  | **Cases with Secondary Dx, LOS > 2 days** | **Cases with Principal DX** | **Matched Cases with No Dx** | **Cases with Secondary Dx, LOS > 2 days** | **Cases with Principal DX** | **Matched Cases with No Dx** |
|  |  |  |  |  |  |  |
|  |  |  |  |  |  |  |
| **n =** | 57,311 | 7,189 | 128,400 | 54,664 | 4,570 | 132,297 |
| **Variables:** |  |  |  |  |  |  |
| **Cost** |  |  |  |  |  |  |
| **mean** | $68,967 | $43,211 | $41,913 | $66,083 | $38,786 | $41,871 |
| **median** | $45,865 | $30,239 | $25,784 | $41,593 | $25,961 | $8,846 |
| **mode** | $16,833 | $4,143 | $541 | $65 | $21,231 | $3,148 |
| **range** | $113 - $1,756,572 | $1,243 - $1,023,780 | $39 - $1,620,419 | $53 - $2,850,000 | $196 - $1,267,532 | $22 - $2,850,000 |
| **LOS** |  |  |  |  |  |  |
| **mean** | 20.01 | 13.92 | 11.55 | 18.80 | 12.47 | 11.28 |
| **median** | 14 | 11 | 7 | 13 | 9 | 7 |
| **mode** | 9 | 11 | 1 | 7 | 7 | 1 |
| **range** | 3 - 360 | 1- 321 | 1 - 357 | 3 - 364 | 1- 190 | 1 - 30 |
| **Number of Diagnoses; mean (SD)** | 18.83 (6.22) | 16.54 (6.20) | 15.36 (6.57) | 21.11 (5.55) | 18.60 (6.09) | 18.08 (6.55) |
| **Number of Procedures; mean (SD)** | 6.76 (4.30) | 4.95 (3.14) | 5.64 (3.81) | 6.61 (3.99) | 4.63 (3.12) | 1.36 (2.06) |
| **age; mean (range)** | 62 (0 - 111) | 62 (0 - 101) | 58 (0-123) | 61 (0 - 90) | 60 (0 - 90) | 58 (0 -90) |
| **APRDRG_Severity; mean (SD)** | 3.93 (0.28) | 3.75 (0.55) | 3.67 (0.60) | 3.93 (0.27) | 3.74 (0.56) | 3.70 (0.53) |
| **APRDRG_Risk_Mortality; mean (SD)** | 3.69 (0.55) | 3.42 (0.74) | 3.46 (0.78) | 3.74 (0.51) | 3.43 (0.70) | 3.59 (0.69) |
| **Wage Index; mean (SD)** | 1.0102 (0.155) | 1.0022 (0.157) | 1.0076 (0.155) | 1.0066 (0.196) | 1.0019 (0.198) | 1.0062 (0.193) |

**Appendix B Table 3-VAP: Results of Median Regression Models 2011 and 2016**

| **Median Regression Estimates for Cost of VAP as a Principal Diagnosis (n= 7179) 2011** | | | | | | | **Median Regression Estimates for Cost of VAP as a Principal Diagnosis (n= 4554) 2016** | | | | | | |
| --- | --- | --- | --- | --- | --- | --- | --- | --- | --- | --- | --- | --- | --- |
| **Parameter** | **Estimate** | **Standard Error** | **95% Confidence Limits** | | **t Value** | **Pr > \|t\|** | **Parameter** | **Estimate** | **Standard Error** | **95% Confidence Limits** | | **t Value** | **Pr > \|t\|** |
| **Intercept** | -17624.8 | 1580.454 | -20722.98 | -14526.67 | -11.15 | <.0001 | **Intercept** | -15338.1 | 1840.17 | -18945.69 | -11730.43 | -8.34 | <.0001 |
| **LOS** | 2277.057 | 30.1457 | 2217.9626 | 2336.1514 | 75.54 | <.0001 | **LOS** | 2346.396 | 45.0501 | 2258.0758 | 2434.7161 | 52.08 | <.0001 |
| **NDX** | 55.5434 | 23.9361 | 8.6216 | 102.4651 | 2.32 | 0.0203 | **NDX** | 89.564 | 32.0557 | 26.7194 | 152.4087 | 2.79 | 0.0052 |
| **NPR** | 1568.727 | 80.8507 | 1410.2358 | 1727.2184 | 19.40 | <.0001 | **NPR** | 1223.852 | 111.3454 | 1005.5609 | 1442.1432 | 10.99 | <.0001 |
| **URBAN_TEACH** | 2507.630 | 283.7512 | 1951.3942 | 3063.8663 | 8.84 | <.0001 | **URBAN_TEACH** | 1138.663 | 335.8536 | 480.2263 | 1797.0991 | 3.39 | 0.0007 |
| **URBAN_TEACH** | 0.0000 | 0.0000 | 0.0000 | 0.0000 | . | . | **URBAN_TEACH** | 0 | 0 | 0 | 0 | . | . |
| **SMALL_BEDSIZE** | -2613.43 | 459.2768 | -3513.743 | -1713.107 | -5.69 | <.0001 | **SMALL_BEDSIZE** | -973.117 | 428.4183 | -1813.025 | -133.2084 | -2.27 | 0.0232 |
| **SMALL_BEDSIZE** | 0.0000 | 0.0000 | 0.0000 | 0.0000 | . | . | **SMALL_BEDSIZE** | 0 | 0 | 0 | 0 | . | . |
| **AGE** | -86.5569 | 6.3352 | -98.9757 | -74.1382 | -13.66 | <.0001 | **AGE** | -96.1349 | 9.4193 | -114.6013 | -77.6684 | -10.21 | <.0001 |
| **APRDRG_Severity** | -378.558 | 308.6955 | -983.6922 | 226.5763 | -1.23 | 0.2201 | **APRDRG_Severity** | -5.3799 | 445.3311 | -878.4454 | 867.6857 | -0.01 | 0.9904 |
| **APRDRG_Risk_Mortality** | 1089.290 | 279.0087 | 542.3503 | 1636.2291 | 3.90 | <.0001 | **APRDRG_Risk_Mortality** | 1103.289 | 344.4458 | 428.0077 | 1778.5702 | 3.2 | 0.0014 |
| **Wage_Index** | 20323.37 | 1203.063 | 17965.008 | 22681.724 | 16.89 | <.0001 | **Wage_Index** | 16152.84 | 978.6369 | 14234.233 | 18071.441 | 16.51 | <.0001 |

**Specified Stage 2 Cost Simulation Models for VAP 2011 and 2016**

**Cost_vap_2011 = -17624.8+ 2277.057*13.9241897+ 55.5434*NDX + 1568.727*NPR + 2507.630*URBAN_TEACH + (-2613.43)*SMALL_BEDSIZE +**

**(-86.5569)*AGE + (-378.558)*APRDRG_Severity + 1089.290*APRDRG_Risk_Mortality + 20323.37*Wage_Index**

**Cost_vap_2016 = -15338.1 + 2346.396*12.4743982 + 89.564*NDX + 1223.852*NPR + 1138.663*URBAN_TEACH * (-973.117)*SMALL_BEDSIZE +**

**(-96.1349) + (-5.3799)*APRDRG_Severity + 1103.289*APRDRG_Risk_Mortality + 16152.84*Wage_Index**

**References**

1. Agency for Healthcare Research and Quality (AHRQ). Healthcare Cost Utilization Project (HCUP). National (Nationwide) Inpatient Sample (NIS) Database Documentation. <https://www.hcup-us.ahrq.gov/db/nation/nis/nisdbdocumentation.jsp>.
2. CMS. Wage Index. Available at: <https://www.cms.gov/Medicare/Medicare-Fee-for-Service-Payment/AcuteInpatientPPS/wageindex>
3. White H. A Heteroskedasticity-Consistent Covariance Matrix Estimator and a Direct Test for Heteroskedasticity. *Econometrica.* 1980; 48 (4): 817–838.
4. Koop G. Introduction to Econometrics. John Wiley & Sons, Ltd. West Sussex, England. 2008.
5. Bastani H, Goh J, Bayati M. Evidence of upcoding in pay-for-performance programs. *Management Science.* 2018;65(3):1042–1060. <https://doi.org/10.1287/mnsc.2017.2996>.
6. Silverman E, Skinner J. Medicare upcoding and hospital ownership. *J Health Econ*. 2004;23(2):369-89.
7. Steinbusch PJ, Oostenbrink JB, Zuurbier JJ, Schaepkens FJ. The risk of upcoding in casemix systems: a comparative study. *Health Policy.* 2007; 81(2-3):289-99.
8. Spika SB, Zweifel P. Buying efficiency: optimal hospital payment in the presence of double upcoding. *Health Econ Rev.* 2019; 28;9(1):38.
9. Meddings J, Saint S, McMahon LF Jr. Hospital-acquired catheter-associated urinary tract infection: documentation and coding issues may reduce financial impact of Medicare's new payment policy*. Infect Control Hosp Epidemiol.* 2010;31(6):627-33.
10. Roberts RR, Scott RD 2nd, Hota B, Kampe LM, Abbasi F, Schabowski S, Ahmad I, Ciavarella GG, Cordell R, Solomon SL, Hagtvedt R, Weinstein RA. Costs attributable to healthcare-acquired infection in hospitalized adults and a comparison of economic methods. *Med Care.* 2010;48(11):1026-35.
11. McGreevy KM, Lipsitz SR, Linder JA, Rimm E, Hoel DG. Using median regression to obtain adjusted estimates of central tendency for skewed laboratory and epidemiologic data. *Clin Chem.* 2009;55(1):165-169.
12. Roy S, Banerjee T. A flexible model for generalized linear regression with measurement error. *AISM.* 2006: 58: 153–169.
13. Lee AH, Fung, WK. Confirmation of multiple outliers in generalized linear and nonlinear regressions. ***CSDA.*** 1997;25: 55-65.
14. Butler, JRG. Hospital Cost Analysis. Kluwer Academic Publishers. Dordrecht, The Netherlands. 1995.
